# Prediction of Reduction Potentials of Copper Proteins with Continuum Electrostatics and Density Functional Theory

**DOI:** 10.1002/chem.201702901

**Published:** 2017-09-21

**Authors:** Nicholas J. Fowler, Christopher F. Blanford, Jim Warwicker, Sam P. de Visser

**Affiliations:** ^1^ Manchester Institute of Biotechnology and School of Chemistry The University of Manchester 131 Princess Street Manchester M1 7DN United Kingdom; ^2^ Manchester Institute of Biotechnology and School of Materials The University of Manchester 131 Princess Street Manchester M1 7DN United Kingdom; ^3^ Manchester Institute of Biotechnology and School of Chemical Engineering and Analytical Science The University of Manchester 131 Princess Street Manchester M1 7DN United Kingdom

**Keywords:** azurin, copper, density functional theory, protein electrostatics, redox

## Abstract

Blue copper proteins, such as azurin, show dramatic changes in Cu^2+^/Cu^+^ reduction potential upon mutation over the full physiological range. Hence, they have important functions in electron transfer and oxidation chemistry and have applications in industrial biotechnology. The details of what determines these reduction potential changes upon mutation are still unclear. Moreover, it has been difficult to model and predict the reduction potential of azurin mutants and currently no unique procedure or workflow pattern exists. Furthermore, high‐level computational methods can be accurate but are too time consuming for practical use. In this work, a novel approach for calculating reduction potentials of azurin mutants is shown, based on a combination of continuum electrostatics, density functional theory and empirical hydrophobicity factors. Our method accurately reproduces experimental reduction potential changes of 30 mutants with respect to wildtype within experimental error and highlights the factors contributing to the reduction potential change. Finally, reduction potentials are predicted for a series of 124 new mutants that have not yet been investigated experimentally. Several mutants are identified that are located well over 10 Å from the copper center that change the reduction potential by more than 85 mV. The work shows that secondary coordination sphere mutations mostly lead to long‐range electrostatic changes and hence can be modeled accurately with continuum electrostatics.

## Introduction

Copper is a relatively abundant transition metal in nature and as such is used in many proteins and enzymes. Its most common biochemical functions relate to electron transfer but it also is involved in oxygen transport, oxygen activation, photosynthesis and respiration.^1^ Of importance are the blue copper proteins that have high reduction potentials as a consequence of their unusual coordination geometry and work as electron transfer agents between two biochemical sites.^2^

**Figure 1:**
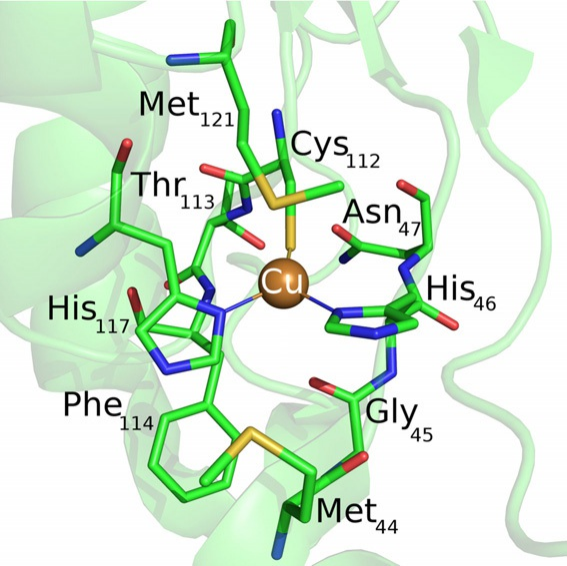
Active site structure of azurin as drawn from the 4AZU pdb file.

Structurally, the blue copper proteins have a conserved primary coordination sphere consisting of a copper(II) ion coordinated by one cysteine and two histidine amino acid side chains and, in addition, have one or two weakly coordinating axial ligands, for example, a methionine and peptide bond carboxyl group, normal to the 2‐His/1‐Cys ligands. Figure 1 shows the active site structure of azurin (*P. aeruginosa*), a typical blue copper protein, as taken from the crystal structure coordinates as reported in the 4AZU protein databank (pdb) file.^3^, ^4^ Despite similarity in individual blue copper protein structures their wildtype (WT) reduction potentials have been shown to spread over a large range: 184–680 mV relative to standard hydrogen electrode (SHE) at pH 7.^5^ Interestingly, an analogous effect has been seen in multicopper oxidases such as laccase.^6^ Many studies have been performed to elucidate the factors that determine the reduction potential of these proteins. The main components contributing to the magnitude of the reduction potential include metal–ligand interactions,^7^ desolvation and/or increased hydrophobicity of the copper center,5a, 8 hydrogen bonding interactions to the thiolate group of copper‐cysteinate interactions,^9^ electrostatics7g,7h, 10 and protein constraint (entatic state) in the enzyme.1a, 11 These copper redox proteins have potential for general use in biotechnology, in particular, if the reduction potential can be fine‐tuned through site‐directed mutagenesis. For instance, this could be used in applications such as enzyme‐catalyzed fuel cells and lignocellulose valorization.^12^, ^13^

Recently, azurin mutants were synthesized with reduction potentials that span the entire physiological range (−954 to +970 mV vs. SHE).^14^ Although this is a major achievement for a redox protein, actually little is known on what determines the reduction potential and the factors that contribute to its magnitude. There are several reported computational studies on reproducing and/or predicting individual reduction potentials of copper proteins; however, a large systematic study that predicts well over a dozen mutants successfully has never been reported and is discussed here. Thus, we describe a novel computational model for predicting reduction potentials of azurin mutants. The set is calibrated and benchmarked against a test set of experimental data from the literature. Azurin is an ideal test system for calibrating and benchmarking reduction potentials because a large number of mutants have been studied potentiometrically and data is known with reasonable accuracy and precision.

Previously reported methods for calculating the reduction potential of copper proteins include continuum electrostatics methods,^15^ molecular dynamics (MD) simulations,^16^ density functional theory (DFT) modeling1d, 17 as well as hybrid quantum/classical methods.^18^ However, in each case only a few mutants were computed. In this work, we investigated a large dataset of reduction potentials and searched for a computationally efficient and reliable method to predict azurin reduction potentials.

Methods which incorporate protein dynamics have been shown to compute the reduction potentials of some copper protein mutants with good agreement with experiment.18b,18g Unfortunately, these methods incur a significant computational cost especially because they need to sample a significant number of protein conformations, which limit the number of mutants that can be investigated. One way in which copper proteins allow for rapid electron transfer is by minimizing the reorganization energy upon reduction and, as a result, have rigid copper coordination centers.^19^ Indeed, crystal structure coordinates of azurin mutants show very little change in structure upon mutation, see the Supporting Information, which confirms the protein is very rigid and probably will not change dramatically during the reduction process.7e, 9d, 11b, 20, 21 In this paper, we exploit the knowledge of the rigidity of the protein by using computational methods that do not involve dynamics but still obtain reasonable estimates of the reduction potential. In particular, we present results of a detailed benchmarking study of two such methods, namely continuum electrostatics and DFT, in which continuum electrostatics is applied due to its low computational cost and DFT for its good accuracy. Ultimately, our methods couple low computational cost with reasonable predictive accuracy within ±25 mV and give a good estimate of expected reduction potentials. Subsequently, we calculate the reduction potential of 124 mutants and identify novel structures that show a large reduction potential change with respect to wildtype may thus have biotechnological applications.

## Results and Discussion

In this work, we present models for calculating reduction potential changes (ΔΔ*E*°′) from WT to mutant structures of azurin through a Born‐Haber cycle. To this end, the reduction potential for the Cu^II^/Cu^I^ couple, that is, Cu^2+^+e^−^⇄Cu^+^, is calculated for WT (Δ*E*°′_WT_) as the difference in energy between the oxidized and reduced states. The same procedure is followed for the mutant structures (Δ*E*°′_M_). As computationally determined individual reduction potentials tend to have a large systematic error with respect to experiment, we will focus on relative reduction potentials (ΔΔ*E*°′) only here. The reduction potential change is then calculated according to Equation (1).

(1) $$\Delta \Delta E{}^{\circ }{}^{'}=\Delta E{}^{\circ }{}^{'}{}_{M}-\Delta E{}^{\circ }{}^{'}{}_{\mathrm{WT}}$$

This paper is organized as follows. First, we discuss calculations of reduction potentials using continuum electrostatics and identify the type of mutations for which this method is most suitable and where it fails. For those in the latter category, we then present computed reduction potentials using DFT. An empirical factor is introduced to overcome an issue both methods have with computing reduction potentials of mutants with hydrophobic axial ligands. Finally, several azurin mutants (never reported on experimentally) are proposed following computation of 124 prospective mutants using continuum electrostatics.

### Continuum electrostatics calculations

Crystal structures suggest7e, 9d, 11b, 20, 21b that point mutations of azurin lead to minor structural changes and hence the main contributor to the reduction potential beyond the primary sphere is most likely electrostatics. As an example, Figure S2 in the Supporting Information shows that the crystal structures of seven mutant azurins have a similar fold to the WT enzyme. Therefore, the work was initiated with a detailed continuum electrostatics study into the reduction potential of 24 azurin mutants for which the reduction potentials were measured previously at pH 7.0 (Supplementary Information, Table S1 for full data).^22^ Our test set only contains reduction potentials measured at pH 7 because at lower pH ligands may dissociate upon reduction. Continuum electrostatics takes a chemical structure and transfers it onto a 3 D grid with point charges on the grid points. The electrostatic potential of each point charge is calculated by solving the finite difference Poisson–Boltzmann equation, which, when multiplied by the partial charge in that grid point, gives an electrostatic energy. The sums of these electrostatic energies were calculated for the oxidized and reduced states to give the corresponding reduction potential.

**Figure 2:**
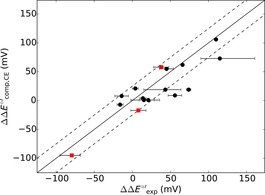
Calculated reduction potential changes (ΔΔ*E*°′) of azurin mutants relative to WT using continuum electrostatics as compared to experiment.^19^ Circles denote mutations made in the secondary coordination sphere of the copper ion and do not alter hydrogen bonds involving ligands. Red squares denote non‐hydrophobic axial ligand mutants. Error bars correspond to the experimental range in reduction potentials for that mutation as taken from the literature.

Figure 2 correlates experimentally determined and continuum electrostatics calculated reduction potential changes between WT azurin and mutants. The data shown in Figure 2 represent a selection of sixteen single mutants with the exception that they do not include variants with hydrophobic axial ligands or ones in which a hydrogen bond is either deleted or inserted toward a primary copper ligand. The remaining mutants will be discussed later in this work. As can be seen, continuum electrostatics methods predict reduction potential changes very well and all except three mutants are found within a typical experimental standard deviation (approximately 25 mV,^23^ dashed line in Figure 2) from experiment. The continuum electrostatics method appears to be most successful at calculating reduction potential changes of mutations made in the secondary coordination sphere, in which we define the second coordination sphere as the region outside the primary coordination sphere where the contributions to the reduction potential only come from dispersion and long‐range Coulombic interactions. Particularly well reproduced are those that do not add or delete hydrogen bonds to copper ligands. Most probably, this is due to only minor changes to the copper binding site structure. Moreover, it is evident that electrostatic interactions are a predominant determinant of the reduction potential shift.

The data in Figure 2 also includes a range of experimental reduction potentials of non‐hydrophobic axial ligand mutations, namely M121X, where X=K, N or Q (identified with red squares in Figure 2), which match well with continuum electrostatics calculations. The axial ligands are located perpendicular to the redox‐active singly occupied molecular orbital (SOMO), so the most significant contribution to the reduction potential change of these mutants could be electrostatics. A subsequent DFT calculation on the M121Q mutant, however, shows a small amount of spin density (ρ_Gln_=0.02) accumulating on the axial ligand indicating a small charge‐transfer contribution of the axial amino acid residue (Supporting Information, Table S7).

In addition to the mutants shown in Figure 2, we calculated reduction potentials of azurin structures with five other hydrophobic axial ligands (M121X mutants with X=G, A, L, V or I). The reduction potential changes of these mutants spanned a large range from +7 mV for M121G to +138 mV for M121I, which have been correlated with the hydrophobicity of the axial ligand,7d, 24 suggesting desolvation effects are key. As a consequence, our continuum electrostatics calculations on these mutants do not agree with experimental values. Other mutations resulting in an increased hydrophobicity of the copper binding site have also been shown to significantly elevate the reduction potential, such as M44F.^25^ This again is not fully captured with continuum electrostatics methods and hence quantum mechanics may be needed to reproduce these experimental results and therefore a DFT study was carried out.

**Scheme 1:**
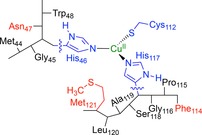
DFT models investigated in this work. Residues highlighted in red were mutated. Wiggly lines show where the active site model was cut from the surrounding protein.

Further complications with the continuum electrostatics method are seen for mutants that either add or delete hydrogen bonds to ligands of copper. For example, in both the F114P and N47P mutants, a hydrogen bond between Cys_112_ sulfur and a backbone amide group is deleted. Spectroscopic and DFT studies have revealed the large covalency of the S(Cys_112_)−Cu bond in the redox‐active SOMO (see below) and how it is altered as hydrogen bonds to the Cys_112_ are deleted.17a A DFT study of iron‐sulfur proteins revealed a linear correlation between number of hydrogen bonds to a thiolate group of the cysteine ligand and the reduction potential.^26^ It is suggested that these hydrogen bonds draw electron density away from the metal, stabilizing the reduced state and increasing the reduction potential as also seen in thiolate ligated heme structures, such as cytochrome P450 Compound I.^27^ Another example is the F114N mutant (Scheme 1), in which a hydrogen bond partner is introduced near to the Gly_45_ and His_117_ amino acids. A recent computational study suggested that hydrogen bonds are formed between the sidechain of Asn_114_ and the backbone amide of His_117_ and the carbonyl group of Gly_45_.18b The formation of such a hydrogen bond could perturb the coordination geometry, leading to a change in reduction potential which cannot be captured by continuum electrostatics.

In summary, continuum electrostatics reproduces experimental reduction potential changes very well, but it is restricted to sites in which the mutations are made in the secondary coordination sphere that do not add or delete hydrogen bonds to copper ligands or alter the solvation of the copper binding site. For azurin, many mutations, therefore, can be predicted with good accuracy using the continuum electrostatics method.

### DFT calculations

For azurin mutants for which the reduction potential is affected by quantum chemical as well as electrostatic effects, we performed a DFT study on model complexes because continuum electrostatics on its own is insufficient. The work specifically focused on mutants that disrupt the hydrogen bonding network near the copper binding site and those with hydrophobic axial ligands (residue 121 in Scheme 1). Amino acid residues that participate in hydrogen bonding interactions to the Cys_112_ thiolate are the amide groups of Asn_47_ and Phe_114_ (highlighted in red in Scheme 1). For example, the N47P, F114N and F114P mutations alter the hydrogen bonding interactions in the copper binding site. Three additional mutants were also investigated for comparison with continuum electrostatics calculations, namely M121N, M121Q and N47S. Models were built from the azurin 4AZU structure^4^ and geometries were optimized for the oxidized and reduced states with DFT at the B3LYP/BS2//B3LYP/BS1 level of theory.

**Figure 3:**
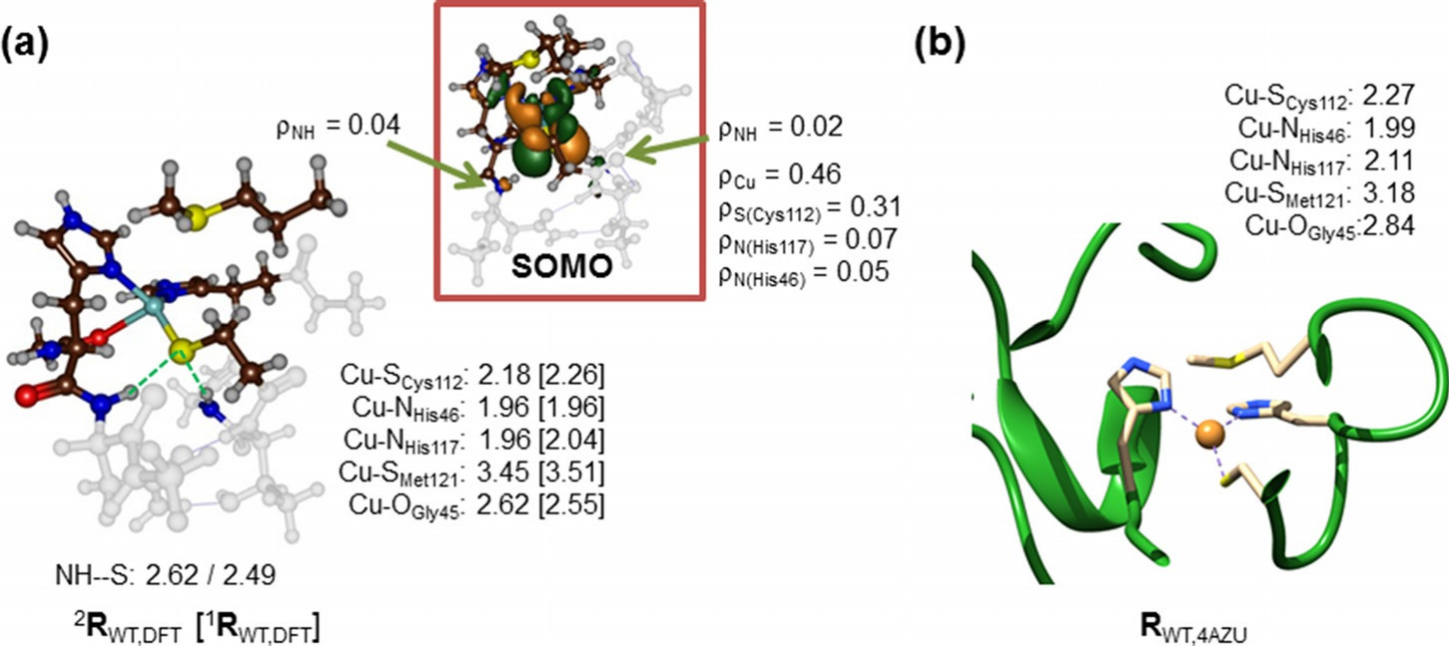
Optimized geometries with distances in Å of the WT oxidized and reduced (in square brackets) reactant complexes (**R**_WT_). a) DFT optimization at B3LYP/BS1 with a polarized continuum model included mimicking water. Also given are the SOMO and group spin densities (ρ). b) Crystal structure coordinates (4AZU structure).

Before we discuss the calculated reduction potential changes between WT and mutants as obtained with DFT, we will focus on the doublet spin ground state reactant complex (^2^ **R**_WT_) as calculated with DFT and how it compares to the crystal structure coordinates. Figure 3 displays DFT‐optimized geometries of the copper(II) complex with respect to the experimentally reported crystal structure. The Cu‐His distances are 1.99 and 2.11 Å, which are typical for metal–histidine ligations seen in analogous non‐heme metalloenzymes.^28^ In our gas‐phase model complex, both histidine residues are located at the same distance from copper; however, due to perturbations in the actual protein that are not present in the model, those distances are different by 0.12 Å. The copper(II)‐Cys_112_ distance is well reproduced with the small model complex to within 0.10 Å. This is surprising because in heme enzymes, it has been found difficult to reproduce the experimentally obtained Fe‐Cys distance.^29^ Nevertheless, overall the small model complex is in good agreement with the experimental structure and therefore should be suitable for reduction potential calculations.

In the Cu^II^ state the system is in a doublet spin state with orbital occupation σ*_z2_^2^ σ*_x2‐y2_^2^ π*_xz_^2^ π*_yz_^2^ π*_xy_^1^, in which the *z*‐axis is defined along the copper‐axial ligand axis. The singly occupied molecular orbital (SOMO) is depicted in Figure 3 a and represents the π* antibonding interaction between copper and Cys_112_ thiolate. This antibonding interaction puts dominant spin density on Cu (0.46) and S (0.31) (Figure 3), which are values in good agreement with previous reports on azurin and plastocyanin.^30^ However, small but significant spin density is also seen on the NH groups of the protein that donate a hydrogen bond to the thiolate of Cys_112_. Consequently, mutations that affect this hydrogen bonding network or the position of these backbone groups will influence the reduction potential dramatically. Indeed that is what is observed here and simple continuum electrostatics modeling cannot capture the effect.

Upon reduction, the electronic state of the copper complex changes to a closed‐shell singlet spin state with orbital occupation: σ*_z2_^2^ σ*_x2‐y2_^2^ π*_xz_^2^ π*_yz_^2^ π*_xy_^2^. Geometrically, reduction of the copper(II) complex leads to minor elongation of the Cu−S_Cys112_ bond by 0.08 Å as a result of double occupation of the antibonding orbital along that axis. At the same time, the axial carboxyl group of the Gly_45_ residue distance to the copper shortens by 0.07 Å.

To find out whether DFT modeling can reproduce reduction potential changes of mutants in which the hydrogen bonding network is disrupted, we evaluated several systems that had either Asn_47_ or Phe_114_ replaced. Table 1 gives calculated values of reduction potential changes upon mutation of either the Asn_47_ or Phe_114_ residues, which are the residues that provide the peptide NH groups for hydrogen‐bonding interactions to the cysteinate of Cys_112_. We used optimization conditions in the gas phase (*ϵ*=1) as well as the addition of a dielectric constant representing chlorobenzene (*ϵ*=5.7) and water (*ϵ*=78.4). Chlorobenzene was chosen as a model solvent as its dielectric constant is close to that found for a protein structure.^31^ Overall, the computed values are in reasonable agreement with experiment suggesting that DFT is a suitable method for predicting the reduction potential of such mutants. The best agreement with experiment is obtained when a dielectric constant mimicking water is applied as the implicit solvent model. Using *ϵ*=78.4, all calculated reduction potentials are within 25 mV from the experimental border regions. Interestingly, the computed reduction potential for the N47S mutant is similar to that obtained with the continuum electrostatics method. A population analysis reveals little difference in charge and spin densities between the redox‐active SOMO of the N47S mutant as compared to WT. This implies that both continuum electrostatics and DFT methods capture the electrostatic modulation of the reduction potential. It has been proposed that the deletion of a hydrogen bond reduces the rigidity of the copper site, which may indirectly lead to a change in reduction potential.^20^ However, this was contradicted by a molecular dynamics study that predicted a reduction potential of 61 mV for the N47S mutant.16b Hence, the effect of increased flexibility of the protein due to reorganization of the hydrogen bonds can be ruled out. Another study on a double mutant that contained the N47S replacement suggested that an increased reduction potential results from interactions between the two ligand‐containing loops.18b

**Table 1:** DFT calculated reduction potential changes (ΔΔ*E*°′) with respect to WT as calculated with solvent models with varying dielectric constant (*ϵ*).^[a]^

Mutation	*ϵ*=1	*ϵ*=5.7	*ϵ*=78.4	Experiment^[b]^
F114N	236	202	170	84 to 145
N47S	88	74	65	90 to 161
N47P	−66	−81	−81	−68 to −27
F114P	−231	−171	−80	−111 to −56

[a] In mV. [b] Experimental range taken from refs. [20, 22b,c,f].

**Figure 4:**
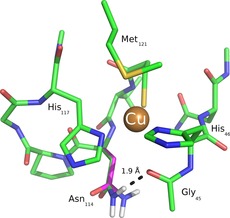
Optimized geometries (B3LYP/BS1) of the F114N mutant in the oxidized (purple) and reduced (green) states. Only the sidechain of N114 is shown for the oxidized state. Apart from hydrogen atoms involved in hydrogen‐bonding interactions, all hydrogens have been removed to aid visualization. In the reduced state, the amine group of Asn_114_ forms a hydrogen bond with the backbone carbonyl oxygen of Gly_45_.

A change in molecular geometry upon reduction is seen in the F114N mutant (Figure 4), in which a hydrogen bond is formed upon reduction. Thus, the position of the Asn_114_ sidechain varies between the two oxidation states, in which its terminal amine group forms a hydrogen bond with the backbone carbonyl of Gly_45_, an interaction that is not seen in the Cu^II^ state. Clearly, DFT has an advantage over continuum electrostatics for this situation and approaches the experimental reduction potential change better. The effect of hydrogen bond formation by Asn_114_ in the F114N mutant was previously reported,18b and found to switch between the backbone amide of His_117_ and either the amide of Gly_45_ or carbonyl of Val_43_. The formation of the latter interactions was found to be a key determinant of the reduction potential.

Solvation effects are important for reproducing the experimental reduction potential change of the F114N and F114P mutants as seen in Table 1. Clearly, the gas phase does not capture the charge redistribution due to the addition/removal of a hydrogen bond well enough and a solvation model corrects for this as also concluded in previous work.9d, 17a Solvation effects are less important for the N47P and N47S mutants, which is reasonable when considering that a residue in position 47 is less solvent exposed than in position 114.

Subsequently, we investigated reduction potentials of axial ligand mutants, in which the Met_121_ group was replaced by either Gln, Asn, Gly, Val or Leu. Table 2 gives the DFT‐calculated reduction potentials of these five axial ligand mutants as a function of the permittivity of the implicit solvent used. Similar to the results presented in Table 1, an implicit solvent model corrects the energy difference of the charged state and brings the reduction potential change closer to the experimentally obtained value(s). However, only the reduction potential of the M121L mutant is within the experimental range, whereas all others are outside these windows by more than 25 mV. As such, DFT is not an accurate method for predicting the reduction potential of hydrophobic axial ligand mutants and does not improve dramatically upon continuum electrostatics calculations for non‐hydrophobic axial ligands mutants. However, the deviation may be systematic and hence an additional factor for hydrophobicity should be included, which will be described in the next section.

**Table 2:** DFT calculated reduction potential changes (ΔΔ*E*°′) with respect to WT as calculated with solvent models with varying dielectric constant (*ϵ*).^[a]^

Mutation	*ϵ*=1	*ϵ*=5.7	*ϵ*=78.4	Experiment^[b]^
M121V	225	148	89	125–145
M121L	222	171	129	86–115
M121G	222	132	88	7
M121N	207	165	88	28‐48
M121Q	−145	−117	−124	−64–91

[a] In mV. [b] Experimental range taken from refs. [7h, 20, 22e,g].

In summary, DFT computes the reduction potentials of mutants that alter hydrogen bonding interactions involving copper ligands with reasonable accuracy. However, our results suggest that DFT fails to predict the reduction potential of mutants with hydrophobic axial ligands and offers no improvement for predicting reduction potentials of non‐hydrophobic axial ligand mutations in comparison to continuum electrostatics.

### Hydrophobic axial ligand cases

**Figure 5:**
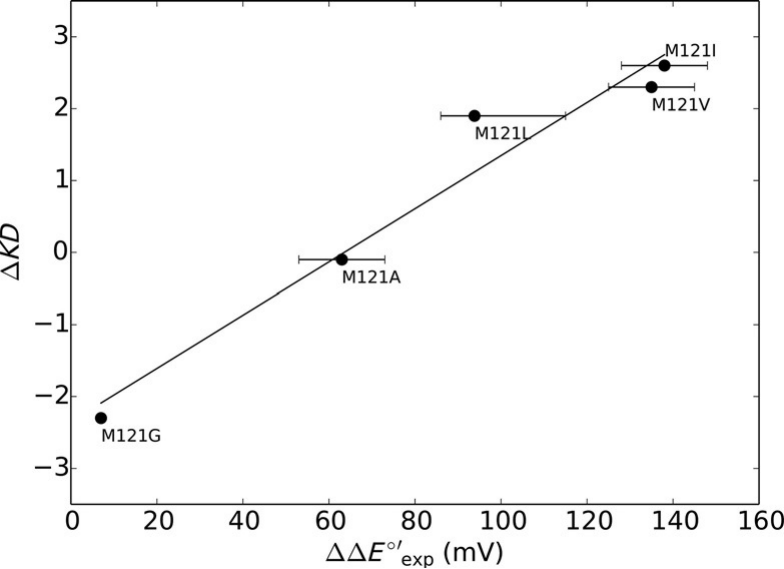
Calibration plot for the Kite–Doolittle hydrophobicity index (ΔKD) for mutants in which the axial ligand is replaced by a hydrophobic group. Obtained fit parameters from trend: *a=*25.8 mV/KD, *b=*64.7 mV. Error bars represent the experimental uncertainties of the reduction potentials.

As discussed in previous sections, both DFT and continuum electrostatics methods fail to reproduce the reduction potentials of azurin mutants with hydrophobic axial ligands. Here, we propose a simple way to approximate the reduction potential of these mutants using a previously reported correlation between reduction potential and the hydrophobicity of the axial ligand,^24^ through the Kyte–Doolittle hydrophobicity index.^32^ Thus, Equation (2) gives the hydrophobicity contribution to the calculated reduction potential (Δ*E*_hydro_) as a function of the Kyte–Doolittle hydrophobicity index (Δ*KD*) and two fit parameters (*a* and *b*) determined from a linear fit through relevant experimental data (Figure 5). Specifically, we plot the hydrophobicity index as a function of the experimental reduction potential change between WT and mutants for a series of hydrophobic axial ligand mutations (M121X, X=G, A, L, I and V) with hydrophobicity indices as taken from ref. 32. From Figure 5 the hydrophobicity correlation for azurin axial ligand mutants can be determined.

(2) $$\Delta E{}_{\mathrm{hydro}}=a\;\Delta KD+b$$

A linear fit through the data in Figure 5 gives the hydrophobic contribution to the reduction potential, which is a systematic component to improve the computational prediction for mutants that contain a hydrophobic axial ligand. Overall, the reduction potential changes for mutants that include hydrophobic axial ligands is described in Equation (3).

(3) $$\Delta \Delta E{}^{\circ }{}^{'}{}_{M/A,\mathrm{predicted}}=\Delta \Delta E{}^{\circ }{}^{'}{}_{M/A,\mathrm{comp}}-\Delta \Delta E{}^{\circ }{}^{'}{}_{A,\mathrm{comp}}+\Delta E{}_{\mathrm{hydro}}$$

Equation (3) gives the calculated reduction potential changes for a mutant with respect to WT as ΔΔ*E*°′_M/A,comp_, and is corrected with the reduction potential change of the axial ligand mutant (ΔΔ*E*°′_A_) and the hydrophobicity factor. The sum of these contributions then gives the predicted mutant reduction potential ΔΔ*E*°′_M/A,predicted_. So, for example, if we are interested in the reduction potential change of the M121L/N47S mutant, we calculate both the M121L/N47S and M121L mutants with continuum electrostatics and correct the difference between the two values with the hydrophobicity factor from Figure 5 to obtain the predicted double mutant reduction potential.

### Benchmarking of reduction potentials of azurin mutants using a combination of modeling techniques

**Figure 6:**
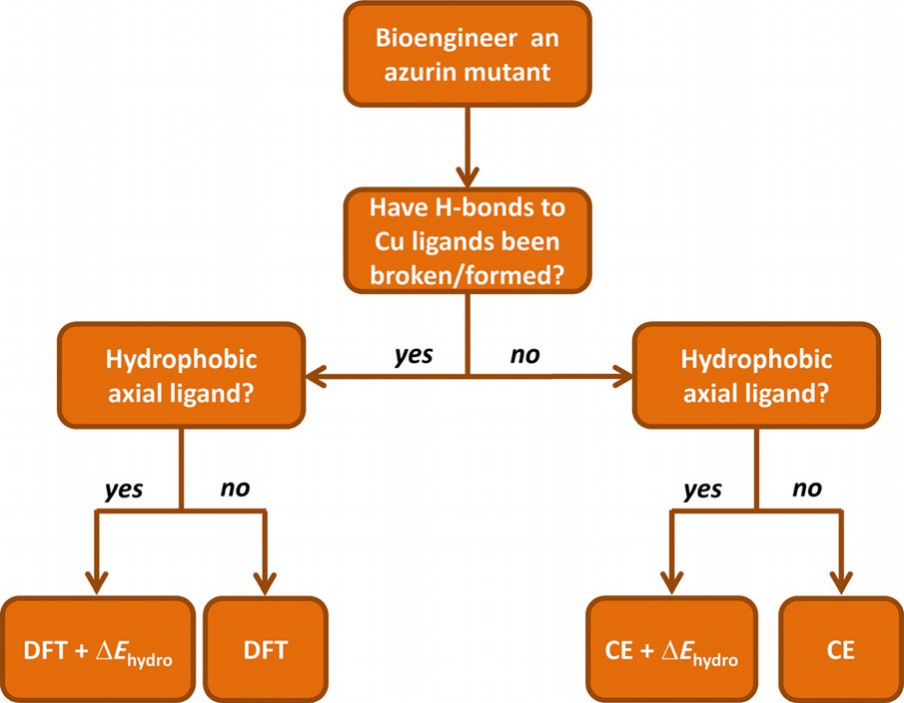
Flow diagram used to decide which computational method to use to compute the predicted reduction potential (ΔΔ*E*°′_predicted_) of an azurin mutant (CE=continuum electrostatics).

A more general scheme that describes how the reduction potential changes of azurin mutants should be predicted is given in Figure 6 as a flow diagram. Thus, if hydrogen‐bonding interactions to copper ligands are deleted or created through mutation then continuum electrostatics methods will most likely fail and a density functional theory or quantum mechanics approach will be needed. Otherwise, a continuum electrostatics calculation should give an accurate result. If the mutation results in a hydrophobic axial ligand then a factor for hydrophobicity will need to be applied to achieve an accurate prediction. If a multiple point mutant includes both a mutation in which hydrogen‐bonding interactions to copper ligands are deleted or created and another mutation in the secondary coordination sphere, then DFT should be used to calculate the reduction potential of a model representing the primary sphere, which includes the hydrogen bond creating/deleting mutation, and continuum electrostatics should be used to calculate the reduction potential of any other mutations made in the secondary sphere. Then, the reduction potential of the multiple point mutant can be approximated by summing the values computed with each method. For example, to compute the reduction potential of the triple mutant M121Q/F114N/N47S, DFT should be used to calculate the reduction potential of M121Q/F114N, continuum electrostatics should be used to calculate the reduction potential of N47S, and then the sum of the two computed values gives the reduction potential for the triple mutant. A similar approach combining DFT and continuum electrostatics calculations was reported previously, and used to successfully predict the reduction potentials of iron‐sulfur proteins.^33^

**Figure 7:**
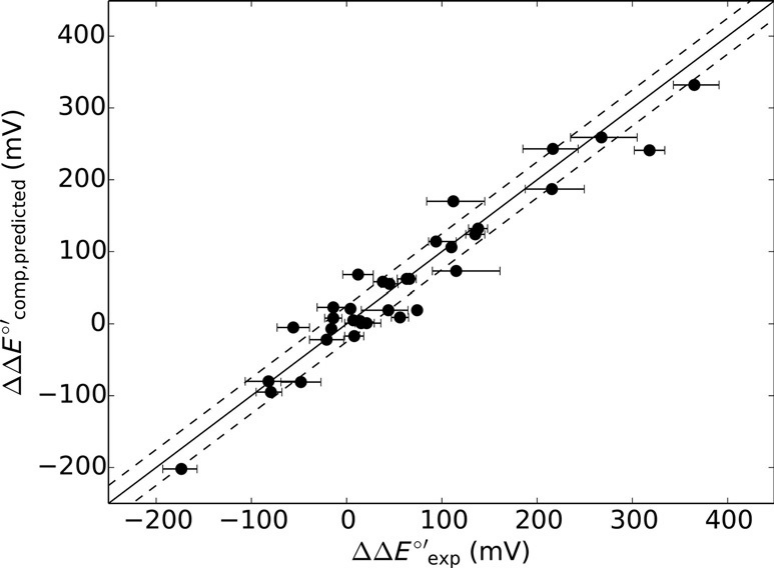
Final calibration trend of our model against experiment. Error bars represent the experimental uncertainties of the reduction potentials.

Following the flow diagram from Figure 6, we reevaluated all reduction potential changes from Figure 2 above and give our best predicted reduction potential changes in Figure 7. Thus, Figure 7 gives calculated reduction potential changes with respect to WT as compared to experimentally determined values for 34 mutants. This set of data contains those from Figure 2 using continuum electrostatics, supplemented with the successful DFT results from Table 1, as well as those with a hydrophobic axial ligand mutation through the hydrophobicity factor and finally ten multiple point mutants. As can be seen, the core of the results matches the experimentally obtained reduction potentials very well and fall within a mean absolute deviation of 24 mV.

One particular outlier from the trend in Figure 7 is the triple mutant M121L/N47S/F114S.22f Due to multiple mutations, our predictive model may incur a large systematic error. Interestingly, the triple mutant has a reduction potential (+318 mV) that is greater than the sum of the reduction potentials of the individual mutants (+254 mV). This implies major structural changes to the protein as a result of the three mutations.

Overall, the results shown in Figure 7 imply that our procedure for calculating reduction potentials of azurin reproduces experimental data with good accuracy and should be sufficient to give reasonable predictions for unknown reduction potentials of azurin mutants. In the next section, the continuum electrostatics method will be applied in a fully predictive manner on systems that have not been experimentally investigated and for which no reduction potentials are known.

### Predictive studies of azurin mutants used a combined model

Finally, to test whether the methods and procedures described in this work could predict novel azurin mutations with large reduction potential changes with respect to WT, we ran a further set of continuum electrostatics calculations on mutants that have not been reported in the literature previously.

To this end, we calculated the reduction potential changes with respect to WT for 124 azurin mutants (see Supporting Information, Table S9 for the full set of results). These mutants replace each individual Arg, Lys, Asp, Glu, Gln, Asn, Ser, Thr and Met amino acid in the secondary coordination sphere with either Lys, Asp or Ala as a single mutation. Apart from the one bound to the copper ion as a ligand there are no free Cys residues in azurin. The only other two Cys residues in the protein form a cystine group and therefore, were not considered for mutation. Aromatic amino acids were not mutated because it would disrupt the protein structure too much. Initially, continuum electrostatics calculations for all 124 mutants were performed. The finite difference/Debye–Hückel (FD/DH) method was used to estimate p*K*_a_ values of inserted charged residues and their protonation states for mutations which suggest changes in reduction potential of at least 20 mV.^34^ Most mutants (see Supporting Information) gave little change in reduction potential as compared to WT. However, in 17 cases, (see Table 3) a reduction potential shift of larger than ±20 mV was obtained.

**Table 3:** Calculated reduction potential changes (ΔΔ*E*°′) of azurin mutants with respect to WT that give a reduction potential shift by more than 20 mV as calculated with continuum electrostatics.

Mutant	ΔΔ*E*°′_M_ [mV]	Distance from Cu^II^ [Å]
N10K	+20	10.6
D11A	+85	9.3
D11K	+96	11.7
M13K	+62	5.9
N16K	+32	12.3
N18K	+59	13.8
K41D	−97	9.3
M56K	+49	12.8
D71A	+24	10.9
D71K	+48	11.9
D77K	+48	16.5
E91K	+28	12.8
K92D	−21	14.3
D93K	+26	13.7
T113A	+26	6.7
S118D	−80	7.8
K122D	−42	9.9

The results in Table 3 imply the scope of modulation of the reduction potential changes of azurin beyond that is known in the literature. Several as‐yet unreported mutations could result in a change of the reduction potential with magnitude between −100 and +100 mV. Particularly interesting is the mutation D11A, in which the removal of negative charge at a distance of about 9 Å from the copper center results in an 85 mV increase in the reduction potential. Adding additional positive charge in this position (D11K), however, does not result in a much higher reduction potential (+96 mV). Our observation for the D11K mutant is in agreement with a recent quantum mechanics/molecular mechanics study on azurin and a few mutants.18b Also interesting is that the average distance between the mutated residue and the copper for some of these mutants is relatively large yet the calculations suggest reasonable shifts in reduction potential.

An analysis of the position of the amino acid residues described in Table 3 reveals them scattered through the second coordination sphere of the protein. Hence, no clear electrostatic or structural origin to the reduction potential changes can be identified. However, it is clear that perturbations in the second coordination sphere and addition or removal of a charged residue has a major effect on the reduction potential in azurin. Similar effects are likely to be seen in other enzymes, but future studies will be needed to confirm this.

## Conclusion

In this work, we present a computational model for predicting the reduction potential of copper proteins. In general, it is shown that continuum electrostatics is a computationally inexpensive and efficient method that gives good agreement with experiment. However, two cases are identified in which continuum electrostatics fails, namely when donating or accepting hydrogen bonds to a copper ligand are perturbed or when hydrophobic axial ligands are introduced. The former can be resolved with a high‐level computation, for example, DFT, and an additional factor is proposed for the latter. Finally, an extensive predictive study is presented on 124 mutants of which 17 mutants give a reduction potential change of more than 20 mV with respect to WT. These mutants may have interesting biotechnological functions.

## Experimental Section

### Continuum electrostatics calculations

The 4AZU pdb file was used as a model of the azurin structure and the coordinates of each of the four chains were extracted.^4^ Mutations were created by replacing specific amino acid sidechains using the SCWRL 4.0 algorithm to find the optimum position of the side chain.^35^ The PDB2PQR software package was used to add hydrogen atoms to the structure, refine hydrogen bonds and assign partial charges and atomic radii to each atom using the CHARMM27 forcefield.^36^ Protonation states of titratable residues at pH 7.0 were determined by calculating their p*K*_a_ values using the in‐house finite difference/Debye–Hückel (FD/DH) method.^34^ All histidine amino acid residues were singly protonated at Nδ (His_35_, His_46_, His_83_ and His_117_). Glu and Asp residues were in the anionic form and Arg/Lys in the protonated form. The only exception to this rule was the Lys_121_ residue in the M121K mutant, which was left unprotonated. Partial charges for the copper ion and its direct ligands, namely His_46_, Cys_112_ and His_117_ in both oxidation states were determined from small gas phase DFT clusters with Gaussian09.^37^ Geometries were optimized with the B3LYP method in combination with a 6‐31G** basis set on all atoms except copper for which we utilized the effective core potential LANL2DZ basis set (basis set BS1).^38^ To improve the energetics and partial charges, single point calculations were performed using a 6‐311++G(2df,p) basis set (BS2) on all atoms and partial charges were calculated using natural bond orbital (NBO) analysis.^39^ The atomic radius of copper was set to 1.71 Å in both oxidation states in accordance with previously reported observations.^40^

Relative reduction potentials were estimated by comparing the difference in total computed electrostatic energy of mutants in each oxidation state, with that of wild type. This was done for each of the four chains (asymmetric units) of the protein and the results were averaged. Electrostatic energies were calculated using a finite‐difference Poisson–Boltzmann method. The linearized finite difference Poisson–Boltzmann equation was solved using adaptive Poisson–Boltzmann solver (APBS).^41^ Calculations were performed on a 57.8 Å^3^ cubic grid with 0.2 Å grid spacing. Charges were mapped onto the grid using cubic B‐spline discretization. The dielectric constant was 4 for regions of the protein and 78 for regions of the solvent. The protein region was defined by a molecular surface determined by a solvent probe with radius 1.4 Å. The value for the dielectric constant was smoothed at the protein‐solvent boundary using 9‐point harmonic averaging. The ionic strength was 0.15 M.

### DFT calculations

Active site models of azurin were constructed in GaussView 5,^42^ based on chain A of the 4AZU pdb crystal structure coordinates.^4^ The model included the copper ion with its three main ligands (His_46_, Cys_112_ and His_117_), the two axial ligands (Gly_45_ and Met_121_), two hydrogen bonds between backbone nitrogen atoms and the Cys_112_ sulfur as well as the two hydrogen bonds that form an interaction between Asn_47_ and Thr_113_. Following a procedure reported previously,17a the model for the F114N mutant also included the additional environment surrounding Asn_114_ (namely Pro_115_, Gly_116_) to aid with its orientation of the polar sidechain. A WT model including this additional environment was also constructed to calculate relative reduction potential of the F114N mutant.

Geometries of both oxidation states were partially optimized using the B3LYP/BS1 method.^38^, ^43^ To mimic the rigidity of the protein backbone and imposition of the axial ligand some atoms were fixed in the models (see Supporting Information for details). As shown before,^44^ differences between zero‐point energies, thermal energies, and entropies of models only make minor contributions to the relative reduction potential, due to the similarity of each of the models.1d Single point energies were calculated using the basis set 6‐311++G(2df,p) for all atoms: Basis set BS2. Optimizations and single point calculations were performed in the gas phase as well in implicit solvent using the integral equation formalism polarization continuum model (IEFPCM) method. Chlorobenzene was chosen to mimic “protein‐like” conditions (*ϵ*=5.6968) and calculations were also performed in water (*ϵ*=78.3553). In general, the value of the dielectric constant appeared to be less critical for the results as long as a value larger than one is used.^45^ As shown previously, for these types of calculations, zero‐point and entropic corrections cancel out,^46^ and hence have not been considered.

## Conflict of interest

The authors declare no conflict of interest.

## Supporting information

As a service to our authors and readers, this journal provides supporting information supplied by the authors. Such materials are peer reviewed and may be re‐organized for online delivery, but are not copy‐edited or typeset. Technical support issues arising from supporting information (other than missing files) should be addressed to the authors.

Supplementary
