# Supplementary material for: Prediction of Reduction Potentials of Copper Proteins with Continuum Electrostatics and Density Functional Theory
Source: Chemistry. 2017 Sep 21;23(61):15436–45. doi: 10.1002/chem.201702901 (PMC5698706; doi:10.1002/chem.201702901)
Supplement: Supplementary file 1 — Supplementary [file CHEM-23-15436-s001.pdf]

CHEMISTRY
A European Journal

Supporting Information

Prediction of Reduction Potentials of Copper Proteins with Continuum Electrostatics and Density Functional Theory

Nicholas J. Fowler,[a] Christopher F. Blanford,[b] Jim Warwicker,*[a] and Sam P. de Visser*[c]

chem_201702901_sm_miscellaneous_information.pdf

Supporting Information

Prediction of reduction potentials of copper proteins with continuum electrostatics and density functional theory

Nicholas J. Fowler,[a] Christopher F. Blanford,[b] Jim Warwicker,*[a] and Sam P. de Visser*[c]

[a] Mr N.J. Fowler, Dr J. Warwicker
Manchester Institute of Biotechnology and School of Chemistry
The University of Manchester
131 Princess Street, Manchester M1 7DN (UK)
E-mail: j.warwicker@manchester.ac.uk

[b] Dr C.F. Blanford
Manchester Institute of Biotechnology and School of Materials
The University of Manchester
131 Princess Street, Manchester M1 7DN (UK)

[c] Dr S.P. de Visser
Manchester Institute of Biotechnology and School of Chemical Engineering and Analytical Science
The University of Manchester
131 Princess Street, Manchester M1 7DN (UK)
E-mail: sam.devisser@manchester.ac.uk

Contents

Table S1 Experimental reduction potentials 3

Table S2 Overlay of the crystal structures of azurin mutants in the PDB 5

Table S3 Optimised geometries and partial charges of the copper site used for continuum electrostatics calculations 6

Table S4 Reduction potentials from continuum electrostatics calculations 8

Table S5 DFT optimised geometries 9

Table S6 Reduction potentials from DFT calculations 92

Table S7 DFT Charge and spin densities 93

Table S8 Calculated reduction potentials from model 99

Table S9 Computed reduction potentials for 124 prospective mutants 100

Table S1 Experimental reduction potentials

Experimental reduction potentials are determined relative to the wild type value obtained by the same research group with the same experimental conditions (e.g. experimental method, buffer, pH = 7.0).

Mutant	WT ΔE (mV)	mutant ΔE (mV)	ΔΔE (mV)	reference
**M44K**	304	370	66	[1]
**M44F**	310 ± 3	384 ±	74 ± 3	[2]
**M44P**	286 ± 8	342 ± 3	56 ± 9	[3]
**N47S**	286 ± 8	385 ± 3	99 ± 9	[4]
	265 ± 16	396 ± 25	131 ± 30	[5]
**N47L***	286	396	110	[6]
**N47P**	286 ± 8	238 ± 19	-48 ± 21	[3]
**W48M**	310 ± 3	323 ± 3	13 ± 4	[2]
	308 ± 3	340 ± 3	32 ± 4	[2]
	294 ± 3	315 ± 3	21 ± 4	[2]
	294	312	18	[7]
**W48L**	310 ± 3	312 ± 3	2 ± 4	[2]
	308 ± 3	345 ± 3	3 ± 4	[2]
	294 ± 3	321 ± 3	27 ± 4	[2]
	294	323	29	[7]
**E91N**	310 ± 3	314 ± 3	4 ± 4	[2]
**S89G**	310 ± 3	294 ± 3	-16 ± 4	[2]
**F114A**	310 ± 3	358 ± 3	48 ± 4	[2]
	294	358	64	[7]
	308 ± 3	341 ± 3	33 ± 4	[2]
	341 ± 3	360 ± 3	19 ± 4	[2]
	294 ± 3	347 ± 3	53 ± 4	[2]
**F114V**	310 ± 3	324 ± 3	14 ± 4	[2]
**F114I**	286 ± 8	272 ± 4	-14 ± 9	[3]
**F114P**	286 ± 8	219 ± 8	-67 ± 11	[4]
	265 ± 16	171 ± 7	-94 ± 17	[5]
**F114N**	265 ± 16	394 ± 4	129 ± 16	[5]
	286 ± 8	381 ± 8	95 ± 11	[8]
**F114S**	286 ± 8	331 ± 4	45 ± 9	[3]
**M121Q**	265 ± 16	190 ± 4	-75 ± 16	[5]
	304	220	-84	[9]
**M121G**	304	311	7	[9]
**M121A**	310 ± 3	373 ± 10	63 ± 10	[2]
**M121L**	265 ± 16	358 ± 4	93 ± 16	[5]
	310 ± 3	412 ± 10	102 ± 10	[2]
	308	375	67	[10]
	297	412	115	[7]
	304	390	86	[9]
	180	280	100	[11]
**M121V**	310 ± 3	445 ± 10	135 ± 10	[2]

Mutant	WT ΔE (mV)	mutant ΔE (mV)	ΔΔE (mV)	reference
**M121I**	310 ± 3	448 ± 10	138 ± 10	[2]
**M121N**	310 ± 3	348 ± 10	38 ± 10	[2]
**M121K**	310 ± 3	318 ± 10	8 ± 10	[2]
**N47S/M121Q**	265 ± 16	244 ± 9	-21 ± 18	[5]
**N47S/M121L**	265 ± 16	496 ± 13	231 ± 21	[5]
	286 ± 8	509 ± 3	223 ± 9	[12]
	310 ± 3	510 ± 10	200 ± 10	[2]
**N47S/F114N**	265 ± 16	494 ± 11	229 ± 19	[5]
	286 ± 8	490 ± 12	204 ± 14	[12]
**F114P/M121Q**	265 ± 16	90 ± 8	-175 ± 18	[5]
	286 ± 8	114 ± 11	-172 ± 14	[12]
**F114P/M121L**	265 ± 16	251 ± 5	-14 ± 17	[5]
**F114N/M121Q**	265 ± 16	209 ± 6	-56 ± 17	[5]
**F114N/M121L**	265 ± 16	551 ± 11	286 ± 19	[5]
	286 ± 8	535 ± 11	249 ± 14	[12]
**N47S/F114N/M121Q**	265 ± 16	277 ± 2	12 ± 16	[5]
**N47S/F114N/M121L**	265 ± 16	640 ± 1	375 ± 16	[5]
	286 ± 8	641 ± 9	355 ± 12	[12]
**N47S/F114S/M121L**	286 ± 8	604 ± 14	318 ± 16	[8]

* Species is *A. denitrificans*

[1] O. Farver, L. K. Skov, M. Vandekamp, G. W. Canters and I. Pecht, *Eur. J. Biochem.* **1992**, *210*, 399-403.
[2] T. Pascher, B. G. Karlsson, M. Nordling, B. G. Malmstrom and T. Vanngard, *Eur. J. Biochem.* **1993**, *212*, 289-296.
[3] N. Marshall in *Fine tuning the reduction potential of cupredoxin proteins by altering secondary coordination sphere interactions (PhD thesis)*, University of Illinois, **2011** URL: https://www.ideals.illinois.edu/handle/2142/26396
[4] S. Y. New, N. M. Marshall, T. S. Hor, F. Xue and Y. Lu, *Chem. Commun.* **2012**, *48*, 4217-4219.
[5] N. M. Marshall, D. K. Garner, T. D. Wilson, Y. G. Gao, H. Robinson, M. J. Nilges and Y. Lu, *Nature* **2009**, *462*, 113-116.
[6] a) C. W. G. Hoitink and G. W. Canters, *Journal of Biological Chemistry* **1992**, *267*, 13836-13842; b) M. C. Machczynski, H. B. Gray and J. H. Richards, *J. Inorg. Biochem.* **2002**, *88*, 375-380.
[7] O. Farver, L. K. Skov, T. Pascher, B. G. Karlsson, M. Nordling, L. G. Lundberg, T. Vanngard and I. Pecht, *Biochemistry* **1993**, *32*, 7317-7322.
[8] O. Farver, P. Hosseinzadeh, N. M. Marshall, S. Wherland, Y. Lu and I. Pecht, *J Phys Chem Lett* **2015**, *6*, 100-105.
[9] A. J. Dibilio, T. K. Chang, B. G. Malmstrom, H. B. Gray, B. G. Karlsson, M. Nordling, T. Pascher and L. G. Lundberg, *Inorg. Chimi. Acta.* **1992**, *198*, 145-148.
[10] B. G. Karlsson, R. Aasa, B. G. Malmstrom and L. G. Lundberg, *Febs Letters* **1989**, *253*, 99-102.
[11] K. M. Lancaster, K. Yokoyama, J. H. Richards, J. R. Winkler and H. B. Gray, *Inorg. Chem.* **2009**, *48*, 1278-1280.
[12] O. Farver, N. M. Marshall, S. Wherland, Y. Lu and I. Pecht, *Proc. Natl. Acad. Sc. USA.* **2013**, *110*, 10536-10540.

Table S2 Overlay of the crystal structures of azurin mutants in the PDB

Crystal structures suggest azurin mutants have similar folds.

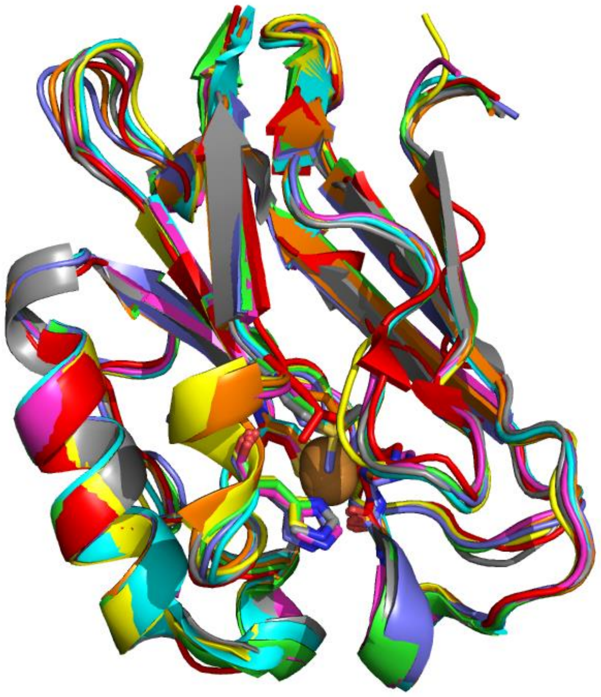

Mutant	Color	PDB ID
WT	Green	4AZU
N47S/F114N	Blue	3JTB
N47S/M121L	Red	3IN2
F114P	Cyan	2GHZ
F114A	Grey	1AZN
F114P/M121Q	Orange	3IN0
M121A	Pink	2TSA
M121G	Yellow	4MFH

Table S3 Optimised geometries and partial charges of the copper site used for continuum electrostatics calculations

Geometries were fully optimised using B3LYP/6-31G** method in the gas phase.

**Atom number**	**Atomic number**	OX			RED		
		X	Y	Z	X	Y	Z
1	29	12.46913	52.26644	31.52891	12.59483	51.88963	32.04328
2	7	11.37938	53.07155	32.90025	11.27017	52.88257	32.99416
3	6	10.63124	54.22784	32.74976	10.79805	54.02687	32.36784
4	6	9.934399	54.45882	33.90205	9.80462	54.59171	33.11668
5	7	10.26719	53.42627	34.75324	9.661703	53.77662	34.22134
6	6	11.13467	52.61232	34.12517	10.56354	52.76019	34.10789
7	1	10.64743	54.79913	31.8352	11.19792	54.33717	31.4147
8	1	9.251703	55.24194	34.18832	9.194992	55.46859	32.96769
9	1	9.92032	53.29553	35.69403	8.995388	53.89481	34.96853
10	1	11.55727	51.72354	34.56624	10.66255	51.97111	34.83721
11	7	13.92935	51.58887	32.59229	14.35082	51.45317	32.72811
12	6	14.57049	52.21439	33.57646	15.10374	51.98233	33.67988
13	7	15.57984	51.44612	34.02493	16.32018	51.37625	33.72564
14	6	15.59466	50.27128	33.30367	16.34253	50.40183	32.74588
15	6	14.56333	50.37046	32.41284	15.11912	50.46226	32.13937
16	1	14.33412	53.19055	33.96967	14.81452	52.78644	34.33927
17	1	16.22011	51.69805	34.76601	17.07396	51.60827	34.35394
18	1	16.31774	49.4935	33.48671	17.20655	49.77982	32.57487
19	1	14.2381	49.66445	31.66521	14.73042	49.89208	31.30902
20	16	12.1387	52.22837	29.4016	12.44485	51.43059	29.89827
21	6	11.09114	50.73678	29.11996	10.67791	51.42799	29.37015
22	1	11.62218	49.83208	29.42048	10.20096	50.47089	29.60218
23	1	10.86018	50.67687	28.05542	10.62344	51.58199	28.28782
24	1	10.15942	50.81041	29.68333	10.10174	52.22144	29.85762

NBO partial charges obtained following a single point energy calculation using B3LYP/6-311++G(2df,p) method. NBO charges are listed for the atoms in the DFT model, namely Cu and the sidechains of His46, His117 and Cys112. CHARMM27 charges were applied on all other methods.

**Residue**	**PDB atom code**	**Partial charges**	
		**OX**	**RED**
Cu	Cu	0.64	0.38
His46	ND1	-0.59	-0.55
His46	CE1	0.25	0.24
His46	NE2	-0.51	-0.54
His46	CD2	-0.04	-0.07
His46	CG	-0.03	-0.03
His46	HE1	0.22	0.2
His46	HE2	0.43	0.41
His46	HD2	0.24	0.22
Cys112	SG	0	-0.44
Cys112	CB	-0.7	-0.69
Cys112	HB3	0.21	0.18
Cys112	HB2	0.22	0.17
His117	ND1	-0.59	-0.55
His117	CG	-0.03	-0.04
His117	CD2	-0.04	-0.07
His117	NE2	-0.51	-0.54
His117	CE1	0.25	0.23
His117	HD2	0.24	0.22
His117	HE2	0.43	0.41
His117	HE1	0.22	0.2

Table S4 Reduction potentials from continuum electrostatics calculations

Reduction potentials of 24 azurin single point mutants calculated using continuum electrostatics.

Mutant	ΔΔE	SD	Experimental range	Mutant type
M44F	19	1.7	71 to 77	Secondary sphere
M44K	62	5.4	66	Secondary sphere
M44P	9	3.0	47 to 65	Secondary sphere
N47P	68	9.8	-68 to -27	Deletes H-bond to S(Cys_{112})
N47S	73	11.0	90 to 161	Secondary sphere
N47L	106	9.0	110	Secondary sphere
W48M	1	1.4	9 to 36	Secondary sphere
W48L	1	0.0	-2 to 29	Secondary sphere
S89G	-7	0.5	-20 to -12	Secondary sphere
E91N	21	1.7	0 - 8	Secondary sphere
F114A	19	0.5	15 to 64	Secondary sphere
F114I	8	0.5	-23 to -5	Secondary sphere
F114N	44	9.0	84 to 145	H-bond donor introduced near to Gly_{46} and His_{117}
F114P	25	32.9	-111 to -56	Deletes H-bond to S(Cys_{112})
F114S	55	19.4	36 to 54	Secondary sphere
F114V	4	0.5	10 to 18	Secondary sphere
M121A	45	5.3	53 to 73	Hydrophobic axial ligand
M121G	58	5.2	7	Hydrophobic axial ligand
M121I	29	3.4	128 to 138	Hydrophobic axial ligand
M121K	-17	11.7	2 to 18	Non-hydrophobic axial ligand
M121L	31	2.8	86 to 115	Hydrophobic axial ligand
M121N	58	4.6	28 to 48	Non-hydrophobic axial ligand
M121Q	-95	34.8	-91 to -64	Non-hydrophobic axial ligand
M121V	30	3.5	125 to 145	Hydrophobic axial ligand

Table S5 DFT optimised geometries

Geometries were partially optimised using B3LYP/6-31G** method in gas phase (ε = 1), chlorobenzene (ε = 5.7) and water (ε = 78.4). Cartesian coordinates of optimised geometries are given below. Atoms with fixed coordinates during optimisation are highlighted with an asterisk. OX – oxidised Cu(II) state, RED – reduced Cu(I) state.

WT - OX

Atom number	Atomic number	ε = 1			ε = 5.7			ε = 78.4		
		X	Y	Z	X	Y	Z	X	Y	Z
1	29	12.53	52.31	31.52	12.55	52.37	31.50	12.58	52.42	31.49
2	16	12.25	52.13	29.39	12.25	52.17	29.36	12.27	52.19	29.34
3	6	11.13	50.75	28.86	11.17	50.75	28.86	11.18	50.76	28.86
4*	6	11.21	50.54	27.34	11.21	50.54	27.34	11.21	50.54	27.34
5	6	12.64	50.15	26.95	12.63	50.14	26.90	12.62	50.15	26.89
6	8	13.11	49.04	27.22	13.06	49.00	27.10	13.06	49.00	27.09
7	7	13.38	51.13	26.35	13.38	51.13	26.33	13.37	51.13	26.32
8*	6	14.73	50.89	25.85	14.73	50.89	25.85	14.73	50.89	25.85
9	6	15.83	51.53	26.70	15.83	51.49	26.75	15.83	51.49	26.75
10	8	16.97	51.67	26.23	17.00	51.53	26.34	17.00	51.51	26.35
11	7	15.50	51.96	27.93	15.46	52.04	27.93	15.46	52.04	27.93
12*	6	16.49	52.55	28.82	16.49	52.55	28.81	16.48	52.55	28.81
13	6	17.45	51.51	29.45	17.37	51.40	29.35	17.39	51.41	29.34
14	8	17.38	51.24	30.65	16.90	50.31	29.64	16.96	50.26	29.50
15	7	18.36	50.97	28.60	18.67	51.74	29.54	18.64	51.79	29.67
16	6	19.32	49.97	29.02	19.66	50.80	30.06	19.62	50.89	30.25
17*	6	14.76	47.95	31.60	14.76	47.95	31.60	14.76	47.95	31.60
18	6	13.85	46.97	30.85	13.88	46.94	30.85	13.88	46.94	30.86
19	6	14.09	49.33	31.52	14.10	49.34	31.48	14.10	49.34	31.48
20	8	12.85	46.51	31.40	12.92	46.40	31.43	12.94	46.38	31.43
21	7	14.16	46.72	29.55	14.15	46.74	29.54	14.15	46.74	29.54
22	6	14.68	50.39	32.39	14.67	50.39	32.39	14.68	50.40	32.37
23*	6	13.32	45.88	28.69	13.32	45.88	28.69	13.32	45.88	28.69
24	6	15.83	50.39	33.13	15.76	50.36	33.21	15.78	50.37	33.19
25	7	14.12	51.67	32.48	14.10	51.67	32.48	14.13	51.68	32.44
26	7	15.96	51.66	33.65	15.86	51.62	33.78	15.89	51.63	33.74
27	6	14.92	52.41	33.23	14.85	52.38	33.32	14.89	52.39	33.27
28	7	11.42	53.18	32.89	11.45	53.22	32.90	11.49	53.23	32.90
29	6	10.47	54.19	32.69	10.49	54.21	32.72	10.51	54.21	32.76
30	6	11.47	52.95	34.19	11.55	52.99	34.20	11.62	53.00	34.21
31	6	10.08	54.72	31.34	10.06	54.74	31.39	10.05	54.75	31.44
32	6	9.96	54.54	33.92	10.01	54.56	33.96	10.06	54.55	34.01
33	7	10.61	53.75	34.85	10.69	53.78	34.87	10.77	53.78	34.90
34	6	11.27	55.28	30.53	11.23	55.31	30.55	11.20	55.32	30.57

35	7	12.04	56.27	31.26	12.03	56.28	31.27	12.01	56.29	31.28
36	6	10.77	55.93	29.22	10.71	55.96	29.26	10.65	55.98	29.30
37	6	13.20	55.94	31.86	13.21	55.94	31.83	13.21	55.95	31.81
38	8	10.50	57.13	29.18	10.37	57.15	29.25	10.24	57.14	29.31
39	7	10.66	55.07	28.19	10.65	55.14	28.20	10.67	55.18	28.21
40*	6	13.96	57.07	32.51	13.96	57.07	32.51	13.96	57.07	32.51
41	8	13.62	54.77	31.91	13.65	54.78	31.81	13.66	54.79	31.75
42	6	10.01	55.41	26.94	10.08	55.52	26.92	10.11	55.57	26.93
43	6	9.07	54.24	26.57	9.11	54.40	26.47	9.11	54.48	26.46
44	6	11.04	55.79	25.85	11.17	55.88	25.89	11.21	55.91	25.91
45	8	8.96	53.26	27.29	8.90	53.40	27.16	8.86	53.49	27.15
46	7	8.35	54.42	25.43	8.48	54.62	25.29	8.52	54.71	25.27
47	6	12.03	54.67	25.54	12.13	54.72	25.61	12.15	54.74	25.63
48	6	7.39	53.44	24.94	7.50	53.70	24.74	7.51	53.81	24.73
49	7	13.15	55.01	24.88	13.30	55.04	25.02	13.32	55.05	25.03
50	8	11.78	53.50	25.87	11.83	53.56	25.91	11.84	53.58	25.93
51*	6	9.90	47.88	31.09	9.90	47.88	31.09	9.90	47.88	31.09
52*	6	9.41	46.46	30.92	9.41	46.46	30.92	9.41	46.46	30.92
53*	6	10.16	48.22	32.54	10.16	48.22	32.54	10.16	48.22	32.54
54	16	10.57	49.99	32.85	10.57	49.99	32.86	10.58	49.98	32.87
55	6	8.92	50.73	32.57	8.94	50.74	32.57	8.94	50.75	32.60
56	1	11.42	49.85	29.39	11.51	49.85	29.38	11.52	49.87	29.39
57	1	10.12	51.03	29.15	10.16	50.98	29.19	10.17	50.99	29.19
58*	1	10.85	51.40	26.82	10.85	51.40	26.82	10.85	51.40	26.82
59*	1	10.60	49.70	27.10	10.60	49.70	27.10	10.60	49.70	27.10
60	1	12.93	52.02	26.13	12.94	52.04	26.16	12.94	52.04	26.16
61*	1	14.85	49.83	25.80	14.85	49.83	25.80	14.85	49.83	25.80
62	1	14.54	51.84	28.26	14.51	51.94	28.26	14.50	51.97	28.25
63*	1	17.09	53.28	28.31	17.09	53.28	28.31	17.09	53.28	28.31
64*	1	16.04	52.98	29.69	16.04	52.98	29.69	16.04	52.98	29.69
65	1	18.26	51.20	27.61	19.01	52.61	29.16	18.93	52.73	29.45
66	1	19.05	48.97	28.66	20.32	50.43	29.27	20.34	50.53	29.50
67	1	20.32	50.22	28.64	20.27	51.28	30.83	20.17	51.39	31.05
68*	1	14.78	47.62	32.62	14.78	47.62	32.62	14.78	47.62	32.62
69	1	13.03	49.21	31.78	13.03	49.25	31.68	13.03	49.25	31.70
70	1	14.10	49.69	30.48	14.20	49.70	30.45	14.18	49.69	30.44
71	1	14.95	47.20	29.13	14.91	47.25	29.12	14.92	47.24	29.12
72*	1	13.80	44.94	28.50	13.80	44.94	28.50	13.80	44.94	28.50
73*	1	12.41	45.72	29.23	12.41	45.72	29.23	12.41	45.72	29.23
74*	1	13.11	46.37	27.76	13.11	46.37	27.76	13.11	46.37	27.76
75	1	16.58	49.63	33.28	16.47	49.58	33.43	16.48	49.58	33.41
76	1	16.75	52.00	34.18	16.57	51.92	34.43	16.60	51.93	34.39
77	1	14.79	53.46	33.43	14.70	53.42	33.55	14.75	53.44	33.51
78	1	12.10	52.21	34.66	12.20	52.27	34.67	12.29	52.29	34.66
79	1	9.33	55.50	31.48	9.31	55.52	31.55	9.30	55.53	31.62

80	1	9.61	53.93	30.74	9.58	53.95	30.80	9.56	53.96	30.85
81	1	9.22	55.27	34.20	9.26	55.28	34.26	9.32	55.26	34.33
82	1	10.45	53.75	35.84	10.56	53.78	35.88	10.67	53.78	35.91
83	1	11.93	54.45	30.31	11.89	54.48	30.31	11.86	54.50	30.32
84	1	11.78	57.23	31.10	11.73	57.24	31.22	11.69	57.25	31.29
85	1	10.87	54.08	28.32	10.93	54.16	28.30	11.00	54.22	28.29
86	1	13.51	58.05	32.32	13.51	58.05	32.32	13.51	58.05	32.34
87	1	14.99	57.07	32.14	14.99	57.07	32.16	14.99	57.08	32.15
88	1	14.00	56.90	33.59	13.97	56.89	33.59	13.98	56.87	33.58
89	1	9.40	56.30	27.13	9.49	56.42	27.10	9.53	56.49	27.11
90	1	11.59	56.68	26.19	11.73	56.74	26.27	11.79	56.76	26.29
91	1	10.54	56.08	24.92	10.72	56.19	24.94	10.78	56.24	24.96
92	1	8.46	55.29	24.91	8.60	55.51	24.83	8.66	55.59	24.80
93	1	7.39	52.61	25.64	7.75	52.69	25.08	7.85	52.77	24.84
94	1	6.39	53.88	24.88	6.48	53.94	25.08	6.55	53.92	25.24
95	1	7.68	53.08	23.94	7.53	53.73	23.65	7.37	54.03	23.67
96	1	13.82	54.28	24.63	13.95	54.30	24.77	13.96	54.29	24.78
97	1	13.34	55.96	24.61	13.52	56.00	24.77	13.56	56.00	24.79
98*	1	10.85	47.93	30.60	10.85	47.93	30.60	10.85	47.93	30.60
99*	1	9.10	48.52	30.77	9.10	48.52	30.77	9.10	48.52	30.77
100*	1	8.88	46.16	31.80	8.88	46.16	31.80	8.88	46.16	31.80
101*	1	10.24	45.81	30.77	10.24	45.81	30.77	10.24	45.81	30.77
102*	1	8.75	46.41	30.08	8.75	46.41	30.08	8.75	46.41	30.08
103*	1	9.24	48.04	33.05	9.24	48.04	33.05	9.24	48.04	33.05
104*	1	11.04	47.66	32.81	11.04	47.66	32.81	11.04	47.66	32.81
105	1	9.00	51.80	32.77	9.02	51.81	32.79	9.04	51.82	32.82
106	1	8.19	50.30	33.25	8.19	50.31	33.25	8.20	50.32	33.27
107	1	8.59	50.60	31.54	8.61	50.62	31.54	8.61	50.64	31.56
108	1	19.35	49.95	30.11	19.13	49.96	30.49	19.09	50.03	30.66
109*	1	15.76	47.98	31.20	15.76	47.98	31.20	15.76	47.98	31.20
110	6	14.92	51.41	24.39	14.97	51.42	24.41	14.97	51.41	24.41
111	8	15.09	52.83	24.36	15.18	52.84	24.39	15.18	52.84	24.38
112	6	13.77	51.05	23.47	13.83	51.11	23.45	13.84	51.09	23.45
113	1	13.60	49.97	23.47	13.62	50.03	23.44	13.64	50.01	23.44
114	1	12.84	51.54	23.78	12.91	51.62	23.75	12.92	51.60	23.74
115	1	14.01	51.36	22.45	14.10	51.42	22.44	14.11	51.40	22.44
116	1	15.85	50.95	24.03	15.89	50.93	24.06	15.90	50.93	24.07
117	1	15.97	53.00	24.72	16.05	52.99	24.80	16.04	53.00	24.79

WT – RED

Atom number	Atomic number	ε = 1			ε = 5.7			ε = 78.4		
		X	Y	Z	X	Y	Z	X	Y	Z
1	29	12.62	52.49	31.51	12.59	52.53	31.53	12.63	52.61	31.52
2	16	12.42	52.15	29.29	12.38	52.17	29.30	12.37	52.20	29.30
3	6	11.20	50.83	28.85	11.20	50.82	28.86	11.22	50.82	28.86
4*	6	11.21	50.54	27.34	11.21	50.54	27.34	11.21	50.54	27.34
5	6	12.63	50.14	26.91	12.62	50.14	26.90	12.62	50.14	26.89
6	8	13.09	49.01	27.14	13.06	49.00	27.10	13.06	49.00	27.09
7	7	13.37	51.11	26.30	13.37	51.11	26.30	13.37	51.12	26.30
8*	6	14.73	50.89	25.85	14.73	50.89	25.85	14.73	50.89	25.85
9	6	15.82	51.50	26.73	15.81	51.50	26.76	15.82	51.50	26.76
10	8	16.97	51.62	26.27	16.98	51.57	26.34	16.99	51.54	26.35
11	7	15.49	51.94	27.96	15.45	52.02	27.95	15.45	52.03	27.94
12*	6	16.49	52.55	28.82	16.49	52.55	28.82	16.49	52.55	28.82
13	6	17.54	51.60	29.46	17.41	51.42	29.33	17.41	51.42	29.33
14	8	17.85	51.76	30.64	17.03	50.27	29.47	17.06	50.24	29.38
15	7	18.11	50.68	28.65	18.66	51.85	29.68	18.62	51.85	29.77
16	6	19.19	49.83	29.13	19.68	50.96	30.20	19.58	50.94	30.37
17*	6	14.76	47.95	31.60	14.76	47.95	31.60	14.76	47.95	31.60
18	6	13.90	46.92	30.86	13.90	46.92	30.86	13.89	46.93	30.86
19	6	14.10	49.34	31.49	14.12	49.35	31.47	14.12	49.35	31.49
20	8	13.01	46.30	31.45	13.00	46.30	31.45	12.96	46.36	31.44
21	7	14.16	46.75	29.53	14.15	46.75	29.53	14.15	46.74	29.54
22	6	14.84	50.43	32.21	14.78	50.41	32.29	14.79	50.43	32.29
23*	6	13.32	45.88	28.69	13.32	45.88	28.69	13.32	45.88	28.69
24	6	16.06	50.38	32.83	15.90	50.34	33.09	15.89	50.34	33.11
25	7	14.35	51.73	32.26	14.29	51.72	32.32	14.32	51.74	32.29
26	7	16.31	51.67	33.27	16.09	51.61	33.59	16.10	51.62	33.60
27	6	15.27	52.44	32.89	15.11	52.40	33.11	15.14	52.42	33.09
28	7	11.42	53.20	32.90	11.43	53.24	32.94	11.44	53.28	32.94
29	6	10.44	54.18	32.79	10.44	54.22	32.86	10.44	54.25	32.86
30	6	11.53	52.92	34.18	11.57	52.97	34.23	11.60	53.02	34.23
31	6	10.02	54.73	31.46	9.99	54.77	31.54	9.98	54.81	31.55
32	6	9.97	54.48	34.04	10.00	54.53	34.12	10.01	54.55	34.13
33	7	10.67	53.67	34.91	10.73	53.73	34.98	10.75	53.76	34.98
34	6	11.24	55.20	30.63	11.19	55.23	30.68	11.16	55.27	30.67
35	7	12.03	56.20	31.32	12.01	56.22	31.35	12.00	56.25	31.34
36	6	10.79	55.78	29.29	10.71	55.82	29.35	10.66	55.87	29.36
37	6	13.23	55.91	31.87	13.22	55.92	31.86	13.22	55.94	31.83
38	8	10.47	56.97	29.21	10.32	57.00	29.30	10.22	57.03	29.34
39	7	10.75	54.90	28.27	10.72	54.97	28.31	10.75	55.06	28.28
40*	6	13.96	57.07	32.51	13.96	57.07	32.51	13.96	57.07	32.51
41	8	13.70	54.77	31.89	13.71	54.78	31.82	13.70	54.79	31.75

42	6	10.18	55.28	27.00	10.20	55.37	27.01	10.24	55.48	26.99
43	6	9.26	54.14	26.50	9.25	54.27	26.48	9.25	54.43	26.44
44	6	11.27	55.74	26.00	11.32	55.80	26.05	11.38	55.87	26.03
45	8	8.95	53.21	27.24	8.92	53.30	27.17	8.91	53.44	27.09
46	7	8.72	54.32	25.26	8.71	54.51	25.26	8.73	54.70	25.22
47	6	12.21	54.62	25.57	12.24	54.65	25.63	12.29	54.71	25.65
48	6	7.91	53.27	24.65	7.84	53.54	24.61	7.73	53.84	24.61
49	7	13.47	54.96	25.25	13.51	54.98	25.32	13.55	55.02	25.30
50	8	11.79	53.45	25.48	11.82	53.49	25.55	11.87	53.54	25.66
51*	6	9.90	47.88	31.09	9.90	47.88	31.09	9.90	47.88	31.09
52*	6	9.41	46.46	30.92	9.41	46.46	30.92	9.41	46.46	30.92
53*	6	10.16	48.22	32.54	10.16	48.22	32.54	10.16	48.22	32.54
54	16	10.59	49.97	32.87	10.60	49.97	32.87	10.59	49.97	32.88
55	6	9.03	50.78	32.37	9.03	50.78	32.41	8.97	50.76	32.59
56	1	11.44	49.92	29.41	11.47	49.91	29.40	11.52	49.91	29.39
57	1	10.20	51.16	29.15	10.19	51.11	29.16	10.20	51.07	29.18
58*	1	10.85	51.40	26.82	10.85	51.40	26.82	10.85	51.40	26.82
59*	1	10.60	49.70	27.10	10.60	49.70	27.10	10.60	49.70	27.10
60	1	12.92	52.00	26.12	12.93	52.02	26.13	12.93	52.03	26.14
61*	1	14.85	49.83	25.80	14.85	49.83	25.80	14.85	49.83	25.80
62	1	14.52	51.85	28.31	14.49	51.93	28.31	14.49	51.95	28.29
63*	1	17.09	53.28	28.31	17.09	53.28	28.31	17.09	53.28	28.31
64*	1	16.04	52.98	29.69	16.04	52.98	29.69	16.04	52.98	29.69
65	1	17.96	50.79	27.65	18.93	52.79	29.42	18.87	52.82	29.67
66	1	19.24	48.92	28.53	20.39	50.65	29.43	19.96	50.22	29.64
67	1	20.17	50.33	29.09	20.23	51.45	31.01	20.42	51.52	30.76
68*	1	14.78	47.62	32.62	14.78	47.62	32.62	14.78	47.62	32.62
69	1	13.08	49.28	31.89	13.06	49.29	31.76	13.07	49.29	31.78
70	1	13.99	49.63	30.44	14.12	49.67	30.42	14.11	49.66	30.43
71	1	14.75	47.42	29.07	14.82	47.35	29.09	14.90	47.27	29.11
72*	1	13.80	44.94	28.50	13.80	44.94	28.50	13.80	44.94	28.50
73*	1	12.41	45.72	29.23	12.41	45.72	29.23	12.41	45.72	29.23
74*	1	13.11	46.37	27.76	13.11	46.37	27.76	13.11	46.37	27.76
75	1	16.76	49.58	32.98	16.56	49.52	33.32	16.53	49.52	33.37
76	1	17.20	52.00	33.60	16.84	51.90	34.21	16.84	51.90	34.23
77	1	15.21	53.51	33.05	15.03	53.46	33.31	15.07	53.48	33.28
78	1	12.20	52.20	34.61	12.25	52.25	34.65	12.30	52.31	34.65
79	1	9.32	55.56	31.60	9.30	55.61	31.71	9.29	55.64	31.73
80	1	9.51	53.96	30.87	9.45	54.01	30.97	9.43	54.04	30.98
81	1	9.21	55.17	34.37	9.26	55.22	34.47	9.27	55.24	34.48
82	1	10.55	53.63	35.91	10.64	53.70	35.98	10.68	53.75	35.99
83	1	11.87	54.32	30.51	11.81	54.34	30.54	11.78	54.39	30.52
84	1	11.74	57.16	31.18	11.69	57.18	31.31	11.66	57.20	31.37
85	1	11.10	53.95	28.43	11.10	54.02	28.45	11.14	54.12	28.41
86	1	13.49	58.04	32.31	13.48	58.04	32.34	13.48	58.04	32.36

87	1	14.99	57.09	32.14	14.98	57.10	32.13	14.98	57.11	32.13
88	1	14.00	56.91	33.59	14.01	56.89	33.59	14.01	56.86	33.58
89	1	9.55	56.15	27.19	9.59	56.26	27.20	9.66	56.39	27.18
90	1	11.82	56.56	26.47	11.90	56.60	26.53	11.97	56.67	26.49
91	1	10.82	56.17	25.10	10.90	56.24	25.13	10.98	56.29	25.10
92	1	9.15	54.98	24.64	9.08	55.26	24.69	8.96	55.57	24.76
93	1	7.38	52.74	25.44	7.16	53.11	25.35	8.05	52.80	24.65
94	1	7.20	53.71	23.95	7.25	54.05	23.84	6.76	53.92	25.11
95	1	8.53	52.54	24.11	8.41	52.72	24.15	7.61	54.14	23.56
96	1	14.12	54.24	24.92	14.15	54.25	24.99	14.18	54.27	24.97
97	1	13.80	55.90	25.38	13.85	55.92	25.42	13.88	55.97	25.30
98*	1	10.85	47.93	30.60	10.85	47.93	30.60	10.85	47.93	30.60
99*	1	9.10	48.52	30.77	9.10	48.52	30.77	9.10	48.52	30.77
100*	1	8.88	46.16	31.80	8.88	46.16	31.80	8.88	46.16	31.80
101*	1	10.24	45.81	30.77	10.24	45.81	30.77	10.24	45.81	30.77
102*	1	8.75	46.41	30.08	8.75	46.41	30.08	8.75	46.41	30.08
103*	1	9.24	48.04	33.05	9.24	48.04	33.05	9.24	48.04	33.05
104*	1	11.04	47.66	32.81	11.04	47.66	32.81	11.04	47.66	32.81
105	1	9.08	51.81	32.70	9.09	51.82	32.74	9.09	51.82	32.81
106	1	8.17	50.29	32.85	8.18	50.30	32.91	8.21	50.34	33.25
107	1	8.90	50.76	31.28	8.87	50.77	31.33	8.65	50.65	31.55
108	1	19.00	49.57	30.18	19.18	50.07	30.58	19.12	50.38	31.19
109*	1	15.76	47.98	31.20	15.76	47.98	31.20	15.76	47.98	31.20
110	6	14.98	51.41	24.40	15.00	51.41	24.41	15.00	51.41	24.41
111	8	15.29	52.81	24.37	15.31	52.82	24.39	15.31	52.81	24.38
112	6	13.81	51.17	23.46	13.84	51.17	23.45	13.85	51.16	23.45
113	1	13.53	50.11	23.46	13.56	50.12	23.44	13.57	50.11	23.44
114	1	12.94	51.75	23.76	12.97	51.76	23.74	12.97	51.75	23.74
115	1	14.09	51.46	22.44	14.13	51.47	22.44	14.14	51.45	22.43
116	1	15.86	50.87	24.03	15.88	50.87	24.05	15.89	50.87	24.05
117	1	16.16	52.87	24.81	16.16	52.89	24.85	16.16	52.90	24.83

WT (including Pro115, Gly116) – OX

Atom number	Atomic number	ε = 1			ε = 5.7			ε = 78.4		
		X	Y	Z	X	Y	Z	X	Y	Z
1	29	12.26	52.09	31.67	12.31	52.20	31.64	12.34	52.27	31.62
2	16	12.18	52.04	29.51	12.22	52.08	29.47	12.24	52.10	29.44
3	6	11.10	50.69	28.86	11.13	50.71	28.86	11.15	50.73	28.87
4*	6	11.21	50.54	27.34	11.21	50.54	27.34	11.21	50.54	27.34
5	6	12.64	50.15	26.95	12.63	50.14	26.91	12.63	50.15	26.91
6	8	13.10	49.04	27.21	13.07	49.01	27.12	13.07	49.01	27.12
7	7	13.38	51.13	26.34	13.38	51.13	26.34	13.37	51.13	26.33
8*	6	14.73	50.89	25.85	14.73	50.89	25.85	14.73	50.89	25.85

9	6	15.82	51.52	26.73	15.82	51.50	26.74	15.82	51.49	26.74
10	8	16.99	51.57	26.33	17.00	51.52	26.34	17.00	51.48	26.36
11	7	15.45	52.08	27.91	15.45	52.07	27.90	15.45	52.07	27.90
12*	6	16.49	52.55	28.82	16.49	52.55	28.81	16.49	52.55	28.82
13	6	17.35	51.33	29.20	17.35	51.33	29.21	17.35	51.33	29.21
14	8	16.78	50.28	29.53	16.78	50.27	29.53	16.78	50.28	29.54
15*	6	14.76	47.95	31.60	14.76	47.95	31.60	14.76	47.95	31.60
16	6	13.82	47.00	30.83	13.84	46.97	30.84	13.86	46.95	30.84
17	6	14.13	49.35	31.48	14.12	49.34	31.48	14.11	49.34	31.48
18	8	12.76	46.65	31.36	12.83	46.54	31.40	12.86	46.49	31.42
19	7	14.16	46.70	29.56	14.16	46.72	29.55	14.16	46.72	29.55
20	6	14.62	50.39	32.44	14.57	50.37	32.48	14.53	50.35	32.50
21*	6	13.32	45.88	28.69	13.32	45.88	28.69	13.32	45.88	28.69
22	6	15.78	50.48	33.16	15.67	50.39	33.31	15.56	50.33	33.41
23	7	13.87	51.55	32.66	13.88	51.57	32.64	13.87	51.58	32.63
24	7	15.72	51.69	33.82	15.62	51.60	33.97	15.52	51.54	34.08
25	6	14.58	52.31	33.49	14.55	52.29	33.54	14.50	52.26	33.58
26	7	11.22	53.15	32.97	11.26	53.18	32.99	11.26	53.21	32.97
27	6	10.39	54.23	32.71	10.38	54.24	32.78	10.37	54.26	32.77
28	6	11.25	52.97	34.28	11.32	52.97	34.30	11.32	52.99	34.28
29	6	10.05	54.74	31.34	10.01	54.77	31.43	9.99	54.80	31.42
30	6	9.92	54.70	33.92	9.93	54.66	34.00	9.91	54.67	33.99
31	7	10.48	53.90	34.89	10.52	53.84	34.94	10.51	53.85	34.93
32	6	11.28	55.26	30.54	11.21	55.28	30.60	11.20	55.31	30.59
33	7	12.04	56.26	31.25	12.01	56.27	31.30	12.01	56.28	31.29
34	6	10.84	55.87	29.20	10.73	55.91	29.28	10.72	55.94	29.27
35	6	13.16	55.92	31.94	13.17	55.93	31.91	13.17	55.94	31.90
36	8	10.75	57.09	29.07	10.51	57.12	29.20	10.48	57.15	29.20
37	7	10.57	54.98	28.23	10.58	55.04	28.26	10.57	55.07	28.25
38*	6	13.96	57.07	32.51	13.96	57.07	32.51	13.96	57.07	32.51
39	8	13.51	54.75	32.11	13.55	54.75	32.00	13.55	54.76	31.96
40	6	9.99	55.35	26.96	10.01	55.41	26.98	10.03	55.46	26.96
41	6	9.08	54.17	26.52	9.08	54.26	26.52	9.09	54.33	26.48
42	6	11.06	55.77	25.93	11.10	55.82	25.97	11.14	55.85	25.96
43	8	8.96	53.17	27.22	8.91	53.26	27.21	8.88	53.32	27.15
44	7	8.41	54.35	25.35	8.46	54.46	25.33	8.48	54.56	25.29
45	6	12.05	54.65	25.60	12.08	54.70	25.65	12.11	54.71	25.66
46	6	7.51	53.35	24.79	7.52	53.50	24.77	7.52	53.63	24.73
47	7	13.24	55.03	25.09	13.25	55.05	25.09	13.30	55.05	25.14
48	8	11.75	53.47	25.80	11.80	53.52	25.90	11.80	53.53	25.88
49*	6	9.90	47.88	31.09	9.90	47.88	31.09	9.90	47.88	31.09
50*	6	9.41	46.46	30.92	9.41	46.46	30.92	9.41	46.46	30.92
51*	6	10.16	48.22	32.54	10.16	48.22	32.54	10.16	48.22	32.54
52	16	10.53	50.00	32.86	10.55	49.99	32.86	10.55	49.99	32.86
53	6	8.87	50.70	32.58	8.89	50.72	32.57	8.89	50.72	32.60
54	1	11.38	49.76	29.35	11.43	49.79	29.36	11.45	49.81	29.37
55	1	10.07	50.94	29.14	10.11	50.95	29.16	10.13	50.96	29.17
56*	1	10.85	51.40	26.82	10.85	51.40	26.82	10.85	51.40	26.82
57*	1	10.60	49.70	27.10	10.60	49.70	27.10	10.60	49.70	27.10

58	1	12.92	52.01	26.11	12.93	52.03	26.15	12.93	52.03	26.14
59*	1	14.85	49.83	25.80	14.85	49.83	25.80	14.85	49.83	25.80
60	1	14.51	51.92	28.26	14.50	51.95	28.25	14.50	51.98	28.24
61*	1	17.09	53.28	28.31	17.09	53.28	28.31	17.09	53.28	28.31
62	1	19.51	49.59	28.61	19.52	49.61	28.59	19.48	49.60	28.53
63*	1	14.78	47.62	32.62	14.78	47.62	32.62	14.78	47.62	32.62
64	1	13.06	49.23	31.62	13.04	49.21	31.59	13.02	49.20	31.57
65	1	14.28	49.72	30.45	14.28	49.73	30.47	14.29	49.74	30.48
66	1	15.05	47.04	29.22	15.02	47.10	29.19	15.01	47.12	29.18
67*	1	13.80	44.94	28.50	13.80	44.94	28.50	13.80	44.94	28.50
68*	1	12.41	45.72	29.23	12.41	45.72	29.23	12.41	45.72	29.23
69*	1	13.11	46.37	27.76	13.11	46.37	27.76	13.11	46.37	27.76
70	1	16.64	49.84	33.22	16.47	49.69	33.45	16.31	49.59	33.62
71	1	16.46	52.09	34.38	16.31	51.94	34.63	16.16	51.84	34.80
72	1	14.32	53.32	33.77	14.31	53.29	33.83	14.27	53.27	33.88
73	1	11.79	52.19	34.80	11.90	52.21	34.79	11.90	52.23	34.77
74	1	9.32	55.54	31.44	9.28	55.58	31.57	9.28	55.62	31.57
75	1	9.58	53.94	30.76	9.50	53.99	30.85	9.48	54.03	30.84
76	1	9.26	55.52	34.15	9.23	55.44	34.27	9.21	55.44	34.27
77	1	10.32	53.96	35.88	10.38	53.88	35.94	10.37	53.88	35.93
78	1	11.93	54.40	30.37	11.85	54.43	30.40	11.84	54.45	30.39
79	1	11.85	57.21	30.98	11.76	57.24	31.14	11.75	57.25	31.17
80	1	10.64	53.98	28.41	10.76	54.05	28.40	10.78	54.08	28.39
81	1	13.62	58.05	32.16	13.59	58.05	32.21	13.57	58.05	32.24
82	1	15.01	56.93	32.25	15.00	56.96	32.21	15.00	56.99	32.18
83	1	13.89	57.05	33.60	13.92	57.00	33.60	13.95	56.97	33.60
84	1	9.35	56.22	27.12	9.38	56.30	27.15	9.43	56.35	27.13
85	1	11.59	56.64	26.34	11.63	56.68	26.37	11.69	56.70	26.37
86	1	10.60	56.11	25.00	10.65	56.16	25.03	10.71	56.19	25.02
87	1	8.54	55.21	24.85	8.56	55.36	24.87	8.59	55.46	24.83
88	1	7.45	52.53	25.51	7.78	52.51	25.15	7.89	52.61	24.84
89	1	6.51	53.77	24.64	6.49	53.73	25.06	6.54	53.70	25.23
90	1	7.89	52.98	23.84	7.59	53.51	23.68	7.39	53.84	23.67
91	1	13.90	54.30	24.80	13.90	54.32	24.81	13.95	54.32	24.86
92	1	13.46	56.00	24.92	13.45	56.02	24.87	13.54	56.02	24.95
93*	1	10.85	47.93	30.60	10.85	47.93	30.60	10.85	47.93	30.60
94*	1	9.10	48.52	30.77	9.10	48.52	30.77	9.10	48.52	30.77
95*	1	8.88	46.16	31.80	8.88	46.16	31.80	8.88	46.16	31.80
96*	1	10.24	45.81	30.77	10.24	45.81	30.77	10.24	45.81	30.77
97*	1	8.75	46.41	30.08	8.75	46.41	30.08	8.75	46.41	30.08
98*	1	9.24	48.04	33.05	9.24	48.04	33.05	9.24	48.04	33.05
99*	1	11.04	47.66	32.81	11.04	47.66	32.81	11.04	47.66	32.81
100	1	8.91	51.77	32.79	8.96	51.79	32.78	8.96	51.78	32.81
101	1	8.14	50.24	33.25	8.16	50.27	33.25	8.17	50.27	33.29
102	1	8.55	50.56	31.55	8.57	50.58	31.54	8.55	50.58	31.57
103	6	14.95	51.43	24.41	14.95	51.42	24.41	14.97	51.42	24.41
104	8	15.15	52.86	24.41	15.14	52.85	24.39	15.15	52.85	24.39
105	6	13.81	51.11	23.46	13.81	51.09	23.46	13.83	51.08	23.45
106	1	13.61	50.04	23.43	13.62	50.02	23.45	13.64	50.01	23.44

107	1	12.89	51.62	23.76	12.89	51.60	23.76	12.91	51.59	23.74
108	1	14.07	51.44	22.45	14.07	51.41	22.44	14.10	51.40	22.44
109	1	15.87	50.96	24.05	15.88	50.96	24.05	15.89	50.95	24.06
110	1	16.03	53.00	24.78	16.01	53.02	24.77	16.03	53.02	24.77
111	7	18.69	51.44	29.17	18.69	51.45	29.19	18.69	51.44	29.17
112	6	19.54	50.27	29.47	19.55	50.28	29.46	19.54	50.25	29.41
113	6	19.09	49.52	30.73	19.11	49.49	30.71	19.14	49.46	30.66
114	8	18.95	50.07	31.82	19.01	50.01	31.82	19.10	49.97	31.78
115	6	20.93	50.90	29.61	20.94	50.90	29.62	20.95	50.86	29.52
116	6	20.91	52.02	28.56	20.91	52.05	28.59	20.90	52.02	28.52
117	6	19.48	52.57	28.63	19.48	52.60	28.69	19.48	52.59	28.68
118	7	18.94	48.18	30.54	18.91	48.16	30.48	18.90	48.15	30.42
119	6	18.53	47.29	31.60	18.52	47.24	31.52	18.51	47.22	31.46
120	6	17.02	47.11	31.84	17.01	47.07	31.76	17.01	47.05	31.72
121	8	16.65	46.27	32.66	16.64	46.18	32.52	16.63	46.13	32.45
122	7	16.16	47.93	31.17	16.16	47.94	31.17	16.16	47.96	31.16
123	6	15.88	53.21	30.07	15.86	53.22	30.06	15.86	53.22	30.06
124	6	16.89	54.04	30.84	16.88	54.02	30.85	16.89	54.00	30.87
125	6	17.82	53.45	31.71	17.74	53.41	31.78	17.68	53.36	31.83
126	6	16.94	55.43	30.65	17.00	55.40	30.64	17.08	55.37	30.64
127	6	18.76	54.23	32.38	18.69	54.16	32.48	18.64	54.08	32.55
128	6	17.89	56.22	31.32	17.95	56.16	31.34	18.04	56.09	31.36
129	6	18.80	55.62	32.19	18.79	55.54	32.26	18.82	55.44	32.32
130	1	16.24	55.90	29.97	16.35	55.89	29.92	16.48	55.87	29.89
131	1	17.91	57.29	31.15	18.03	57.23	31.16	18.17	57.15	31.17
132	1	19.48	53.76	33.04	19.34	53.67	33.19	19.24	53.57	33.30
133	1	17.83	52.37	31.85	17.68	52.34	31.94	17.56	52.29	32.01
134	1	19.53	56.22	32.71	19.53	56.12	32.81	19.56	56.00	32.88
135	1	15.05	53.86	29.75	15.06	53.88	29.72	15.08	53.91	29.71
136	1	15.44	52.44	30.70	15.40	52.45	30.68	15.37	52.46	30.67
137	1	21.66	52.79	28.75	21.66	52.82	28.80	21.66	52.78	28.72
138	1	19.41	53.43	29.30	19.41	53.43	29.40	19.44	53.41	29.40
139	1	21.73	50.17	29.46	21.74	50.18	29.44	21.73	50.13	29.30
140	1	21.03	51.31	30.62	21.04	51.29	30.63	21.09	51.23	30.54
141	1	18.95	46.29	31.43	18.93	46.25	31.31	18.92	46.23	31.25
142	1	18.95	47.66	32.54	18.95	47.58	32.46	18.95	47.56	32.41
143	1	18.96	47.83	29.60	18.92	47.83	29.52	18.87	47.82	29.47
144	1	16.52	48.70	30.59	16.52	48.72	30.61	16.51	48.74	30.61
145	1	19.10	52.85	27.64	19.10	52.92	27.72	19.07	52.94	27.72
146	1	21.11	51.60	27.56	21.10	51.66	27.59	21.05	51.65	27.50

WT (including Pro115, Gly116) – RED

Atom number	Atomic number	ε = 1			ε = 5.7			ε = 78.4		
		X	Y	Z	X	Y	Z	X	Y	Z
1	29	12.37	52.32	31.61	12.37	52.39	31.63	12.37	52.43	31.64
2	16	12.36	52.11	29.37	12.35	52.12	29.38	12.32	52.14	29.38
3	6	11.18	50.78	28.86	11.19	50.78	28.86	11.18	50.78	28.86
4*	6	11.21	50.54	27.34	11.21	50.54	27.34	11.21	50.54	27.34
5	6	12.62	50.13	26.90	12.62	50.14	26.89	12.62	50.15	26.90
6	8	13.07	49.00	27.09	13.06	49.00	27.08	13.07	49.01	27.11
7	7	13.37	51.11	26.30	13.37	51.12	26.30	13.36	51.12	26.30
8*	6	14.73	50.89	25.85	14.73	50.89	25.85	14.73	50.89	25.85
9	6	15.81	51.51	26.75	15.81	51.50	26.76	15.81	51.49	26.76
10	8	16.98	51.57	26.35	16.99	51.53	26.36	16.99	51.49	26.38
11	7	15.43	52.06	27.93	15.44	52.06	27.92	15.44	52.07	27.92
12*	6	16.49	52.55	28.82	16.49	52.55	28.82	16.48	52.55	28.82
13	6	17.37	51.34	29.18	17.36	51.34	29.20	17.35	51.33	29.20
14	8	16.84	50.28	29.53	16.81	50.28	29.53	16.79	50.28	29.54
15*	6	14.76	47.95	31.60	14.76	47.95	31.60	14.76	47.95	31.60
16	6	13.84	46.97	30.84	13.86	46.95	30.85	13.86	46.95	30.85
17	6	14.12	49.35	31.49	14.12	49.34	31.49	14.12	49.35	31.49
18	8	12.84	46.53	31.40	12.87	46.47	31.42	12.87	46.47	31.42
19	7	14.16	46.71	29.55	14.16	46.72	29.55	14.15	46.72	29.55
20	6	14.62	50.36	32.48	14.59	50.35	32.50	14.57	50.35	32.51
21*	6	13.32	45.88	28.69	13.32	45.88	28.69	13.32	45.88	28.69
22	6	15.68	50.31	33.34	15.60	50.26	33.43	15.56	50.25	33.47
23	7	14.00	51.60	32.59	14.00	51.60	32.59	13.98	51.60	32.60
24	7	15.70	51.53	33.99	15.61	51.48	34.09	15.55	51.46	34.14
25	6	14.68	52.27	33.50	14.64	52.25	33.55	14.60	52.24	33.58
26	7	11.21	53.19	32.95	11.22	53.22	33.00	11.20	53.26	33.00
27	6	10.31	54.24	32.81	10.31	54.27	32.87	10.29	54.31	32.88
28	6	11.28	52.94	34.25	11.32	52.97	34.29	11.30	53.01	34.30
29	6	9.96	54.80	31.46	9.93	54.83	31.54	9.90	54.87	31.55
30	6	9.85	54.61	34.04	9.88	54.64	34.12	9.86	54.67	34.13
31	7	10.47	53.77	34.95	10.52	53.80	35.01	10.50	53.84	35.01
32	6	11.23	55.19	30.66	11.17	55.23	30.71	11.14	55.26	30.71
33	7	12.02	56.20	31.34	11.99	56.22	31.37	11.98	56.24	31.37
34	6	10.83	55.76	29.30	10.74	55.80	29.36	10.70	55.85	29.37
35	6	13.19	55.89	31.96	13.18	55.90	31.95	13.16	55.92	31.94
36	8	10.59	56.97	29.18	10.44	57.00	29.26	10.35	57.04	29.30
37	7	10.72	54.86	28.31	10.69	54.92	28.34	10.71	55.00	28.32
38*	6	13.96	57.07	32.51	13.96	57.07	32.51	13.96	57.07	32.51
39	8	13.60	54.74	32.11	13.60	54.74	32.04	13.57	54.75	32.01
40	6	10.17	55.25	27.02	10.17	55.32	27.04	10.22	55.42	27.02
41	6	9.26	54.12	26.50	9.23	54.22	26.50	9.25	54.36	26.46
42	6	11.28	55.73	26.05	11.30	55.77	26.08	11.37	55.83	26.07
43	8	8.94	53.17	27.22	8.92	53.24	27.18	8.92	53.36	27.10
44	7	8.73	54.32	25.26	8.69	54.46	25.28	8.72	54.64	25.24
45	6	12.22	54.61	25.61	12.23	54.65	25.66	12.29	54.68	25.67

46	6	7.94	53.28	24.62	7.84	53.49	24.62	7.75	53.76	24.61
47	7	13.49	54.96	25.31	13.50	54.99	25.36	13.55	55.00	25.37
48	8	11.80	53.46	25.49	11.82	53.48	25.57	11.86	53.52	25.61
49*	6	9.90	47.88	31.09	9.90	47.88	31.09	9.90	47.88	31.09
50*	6	9.41	46.46	30.92	9.41	46.46	30.92	9.41	46.46	30.92
51*	6	10.16	48.22	32.54	10.16	48.22	32.54	10.16	48.22	32.54
52	16	10.57	49.98	32.84	10.58	49.98	32.85	10.57	49.98	32.86
53	6	8.97	50.74	32.38	8.97	50.75	32.45	8.92	50.72	32.60
54	1	11.43	49.86	29.38	11.45	49.86	29.38	11.47	49.86	29.37
55	1	10.17	51.08	29.15	10.17	51.06	29.16	10.16	51.02	29.17
56*	1	10.85	51.40	26.82	10.85	51.40	26.82	10.85	51.40	26.82
57*	1	10.60	49.70	27.10	10.60	49.70	27.10	10.60	49.70	27.10
58	1	12.92	52.01	26.11	12.93	52.02	26.12	12.92	52.02	26.11
59*	1	14.85	49.83	25.80	14.85	49.83	25.80	14.85	49.83	25.80
60	1	14.48	51.93	28.30	14.48	51.95	28.29	14.48	51.98	28.28
61*	1	17.09	53.28	28.31	17.09	53.28	28.31	17.09	53.28	28.31
62	1	19.47	49.60	28.49	19.47	49.61	28.51	19.47	49.61	28.50
63*	1	14.78	47.62	32.62	14.78	47.62	32.62	14.78	47.62	32.62
64	1	13.04	49.23	31.64	13.04	49.22	31.61	13.03	49.22	31.60
65	1	14.24	49.74	30.48	14.26	49.75	30.48	14.27	49.75	30.49
66	1	14.97	47.17	29.16	14.98	47.17	29.16	15.00	47.14	29.17
67*	1	13.80	44.94	28.50	13.80	44.94	28.50	13.80	44.94	28.50
68*	1	12.41	45.72	29.23	12.41	45.72	29.23	12.41	45.72	29.23
69*	1	13.11	46.37	27.76	13.11	46.37	27.76	13.11	46.37	27.76
70	1	16.42	49.55	33.54	16.31	49.48	33.66	16.24	49.46	33.72
71	1	16.40	51.84	34.64	16.26	51.75	34.81	16.17	51.72	34.89
72	1	14.50	53.30	33.77	14.45	53.27	33.84	14.41	53.26	33.88
73	1	11.87	52.16	34.70	11.93	52.21	34.74	11.92	52.25	34.75
74	1	9.30	55.66	31.58	9.27	55.69	31.68	9.25	55.74	31.69
75	1	9.43	54.04	30.87	9.37	54.08	30.96	9.34	54.13	30.97
76	1	9.15	55.37	34.35	9.18	55.39	34.44	9.16	55.43	34.45
77	1	10.34	53.76	35.95	10.42	53.80	36.01	10.41	53.84	36.02
78	1	11.82	54.28	30.59	11.76	54.31	30.61	11.73	54.35	30.61
79	1	11.79	57.15	31.11	11.73	57.18	31.22	11.71	57.21	31.27
80	1	11.02	53.89	28.48	11.01	53.96	28.49	11.06	54.05	28.46
81	1	13.59	58.04	32.15	13.55	58.04	32.22	13.54	58.05	32.26
82	1	15.01	56.95	32.24	14.99	56.99	32.17	14.98	57.01	32.14
83	1	13.90	57.06	33.60	13.97	57.00	33.60	14.00	56.97	33.60
84	1	9.53	56.12	27.21	9.55	56.21	27.22	9.63	56.33	27.20
85	1	11.83	56.54	26.55	11.85	56.57	26.58	11.94	56.63	26.55
86	1	10.84	56.18	25.15	10.88	56.22	25.17	10.96	56.26	25.15
87	1	9.17	54.99	24.66	9.05	55.22	24.72	8.97	55.50	24.77
88	1	7.40	52.74	25.39	7.17	53.04	25.36	8.16	52.75	24.50
89	1	7.23	53.73	23.92	7.24	53.99	23.85	6.83	53.69	25.20
90	1	8.57	52.56	24.07	8.42	52.68	24.14	7.51	54.15	23.63
91	1	14.13	54.24	24.98	14.14	54.27	25.02	14.18	54.27	25.03
92	1	13.82	55.90	25.46	13.82	55.94	25.45	13.88	55.95	25.42
93*	1	10.85	47.93	30.60	10.85	47.93	30.60	10.85	47.93	30.60
94*	1	9.10	48.52	30.77	9.10	48.52	30.77	9.10	48.52	30.77

95*	1	8.88	46.16	31.80	8.88	46.16	31.80	8.88	46.16	31.80
96*	1	10.24	45.81	30.77	10.24	45.81	30.77	10.24	45.81	30.77
97*	1	8.75	46.41	30.08	8.75	46.41	30.08	8.75	46.41	30.08
98*	1	9.24	48.04	33.05	9.24	48.04	33.05	9.24	48.04	33.05
99*	1	11.04	47.66	32.81	11.04	47.66	32.81	11.04	47.66	32.81
100	1	8.99	51.78	32.70	9.02	51.79	32.77	9.02	51.79	32.80
101	1	8.15	50.23	32.89	8.16	50.24	32.98	8.19	50.29	33.28
102	1	8.81	50.71	31.30	8.77	50.72	31.37	8.58	50.59	31.57
103	6	15.00	51.42	24.41	15.00	51.41	24.41	15.01	51.41	24.41
104	8	15.32	52.82	24.40	15.30	52.82	24.39	15.30	52.82	24.39
105	6	13.84	51.19	23.45	13.84	51.18	23.45	13.86	51.17	23.44
106	1	13.55	50.14	23.44	13.56	50.12	23.44	13.58	50.11	23.44
107	1	12.97	51.78	23.75	12.96	51.76	23.75	12.98	51.75	23.73
108	1	14.13	51.49	22.44	14.13	51.47	22.44	14.16	51.45	22.43
109	1	15.88	50.88	24.05	15.88	50.88	24.05	15.90	50.87	24.06
110	1	16.18	52.88	24.85	16.16	52.91	24.83	16.17	52.91	24.81
111	7	18.71	51.46	29.12	18.71	51.45	29.16	18.70	51.44	29.17
112	6	19.56	50.28	29.35	19.56	50.27	29.38	19.54	50.25	29.39
113	6	19.20	49.52	30.64	19.18	49.49	30.65	19.16	49.46	30.64
114	8	19.25	50.05	31.74	19.20	50.00	31.77	19.15	49.96	31.76
115	6	20.97	50.89	29.40	20.97	50.87	29.46	20.96	50.85	29.48
116	6	20.90	52.03	28.38	20.90	52.04	28.46	20.90	52.02	28.49
117	6	19.48	52.59	28.56	19.48	52.60	28.64	19.48	52.59	28.67
118	7	18.93	48.20	30.44	18.91	48.17	30.42	18.89	48.15	30.40
119	6	18.54	47.32	31.52	18.53	47.26	31.48	18.51	47.22	31.45
120	6	17.04	47.12	31.79	17.02	47.07	31.74	17.01	47.05	31.70
121	8	16.70	46.24	32.57	16.66	46.17	32.48	16.64	46.12	32.43
122	7	16.16	47.95	31.16	16.16	47.95	31.16	16.16	47.95	31.16
123	6	15.88	53.22	30.07	15.87	53.23	30.06	15.87	53.24	30.05
124	6	16.92	53.97	30.88	16.91	53.99	30.86	16.91	53.99	30.86
125	6	17.78	53.30	31.76	17.71	53.34	31.82	17.67	53.34	31.85
126	6	17.07	55.35	30.72	17.12	55.36	30.64	17.16	55.35	30.62
127	6	18.76	54.00	32.46	18.68	54.04	32.53	18.65	54.03	32.57
128	6	18.05	56.06	31.42	18.09	56.06	31.34	18.13	56.05	31.34
129	6	18.90	55.38	32.30	18.88	55.41	32.29	18.88	55.39	32.32
130	1	16.42	55.88	30.03	16.51	55.87	29.90	16.58	55.87	29.86
131	1	18.15	57.13	31.28	18.24	57.12	31.15	18.30	57.10	31.14
132	1	19.43	53.46	33.14	19.30	53.52	33.26	19.23	53.51	33.33
133	1	17.70	52.23	31.89	17.58	52.28	32.00	17.52	52.28	32.04
134	1	19.66	55.93	32.85	19.64	55.95	32.85	19.63	55.93	32.89
135	1	15.10	53.91	29.74	15.10	53.92	29.71	15.12	53.94	29.70
136	1	15.37	52.46	30.68	15.36	52.49	30.68	15.35	52.51	30.67
137	1	21.66	52.79	28.54	21.67	52.79	28.64	21.67	52.78	28.68
138	1	19.46	53.44	29.25	19.46	53.42	29.37	19.46	53.41	29.40
139	1	21.75	50.15	29.18	21.74	50.14	29.22	21.73	50.12	29.24
140	1	21.14	51.27	30.41	21.13	51.24	30.48	21.12	51.21	30.50
141	1	18.97	46.33	31.37	18.95	46.27	31.29	18.92	46.23	31.23
142	1	18.97	47.73	32.44	18.96	47.64	32.41	18.95	47.57	32.39
143	1	18.74	47.90	29.50	18.81	47.86	29.47	18.84	47.83	29.45

144	1	16.50	48.75	30.62	16.51	48.75	30.62	16.51	48.74	30.62
145	1	19.04	52.90	27.60	19.06	52.95	27.70	19.07	52.95	27.73
146	1	21.02	51.63	27.37	21.03	51.66	27.44	21.03	51.66	27.47

N47P – OX

Atom number	Atomic number	ε = 1			ε = 5.7			ε = 78.4		
		X	Y	Z	X	Y	Z	X	Y	Z
1	29	12.60	52.34	31.51	12.62	52.38	31.50	12.64	52.42	31.50
2	16	12.25	52.14	29.39	12.28	52.15	29.37	12.29	52.16	29.36
3	6	11.12	50.78	28.86	11.16	50.77	28.86	11.17	50.77	28.87
4*	6	11.21	50.54	27.34	11.21	50.54	27.34	11.21	50.54	27.34
5	6	12.64	50.15	26.98	12.63	50.14	26.92	12.63	50.14	26.92
6	8	13.12	49.05	27.28	13.07	49.01	27.15	13.07	49.02	27.16
7	7	13.39	51.12	26.36	13.38	51.11	26.33	13.38	51.11	26.33
8*	6	14.73	50.89	25.85	14.73	50.89	25.85	14.73	50.89	25.85
9	6	15.83	51.52	26.70	15.83	51.47	26.76	15.83	51.46	26.76
10	8	16.98	51.63	26.23	17.01	51.44	26.39	17.01	51.40	26.40
11	7	15.50	51.96	27.93	15.46	52.06	27.92	15.46	52.06	27.92
12*	6	16.48	52.55	28.81	16.48	52.55	28.81	16.48	52.55	28.82
13	6	17.49	51.55	29.46	17.37	51.39	29.33	17.38	51.40	29.33
14	8	17.55	51.46	30.69	16.91	50.29	29.59	16.95	50.26	29.50
15	7	18.29	50.88	28.60	18.67	51.74	29.54	18.64	51.78	29.66
16	6	19.32	49.95	29.05	19.66	50.79	30.03	19.62	50.87	30.23
17*	6	14.76	47.95	31.60	14.76	47.95	31.60	14.76	47.95	31.60
18	6	13.85	46.97	30.85	13.88	46.94	30.85	13.88	46.94	30.86
19	6	14.10	49.33	31.51	14.11	49.34	31.48	14.11	49.34	31.48
20	8	12.85	46.51	31.40	12.92	46.40	31.43	12.94	46.38	31.44
21	7	14.16	46.72	29.55	14.15	46.73	29.54	14.15	46.74	29.54
22	6	14.78	50.42	32.27	14.80	50.43	32.24	14.81	50.43	32.23
23*	6	13.32	45.88	28.69	13.32	45.88	28.69	13.32	45.88	28.69
24	6	15.98	50.42	32.94	15.99	50.42	32.93	16.00	50.43	32.92
25	7	14.27	51.71	32.31	14.29	51.72	32.28	14.31	51.73	32.26
26	7	16.18	51.71	33.37	16.19	51.72	33.37	16.20	51.72	33.35
27	6	15.15	52.47	32.96	15.15	52.47	32.96	15.17	52.48	32.93
28	7	11.70	53.15	33.06	11.76	53.17	33.08	11.79	53.18	33.09
29	6	10.76	54.17	33.07	10.81	54.19	33.13	10.84	54.20	33.15
30	6	11.97	52.85	34.32	12.08	52.88	34.34	12.14	52.90	34.34
31	6	10.11	54.71	31.84	10.11	54.73	31.92	10.12	54.74	31.96
32	6	10.48	54.48	34.37	10.58	54.50	34.44	10.64	54.52	34.47
33	7	11.25	53.64	35.15	11.39	53.67	35.19	11.46	53.69	35.20
34	6	11.05	55.28	30.74	11.01	55.29	30.80	11.00	55.29	30.82
35	7	11.95	56.31	31.26	11.93	56.32	31.29	11.93	56.33	31.29
36	6	10.12	55.74	29.58	10.06	55.73	29.64	10.04	55.70	29.66
37	6	13.18	55.98	31.78	13.17	55.99	31.76	13.17	56.00	31.75
38	8	8.98	56.13	29.83	8.91	56.07	29.90	8.87	56.00	29.91
39	7	10.57	55.57	28.30	10.51	55.59	28.37	10.50	55.60	28.38

40*	6	13.96	57.07	32.51	13.96	57.07	32.51	13.96	57.07	32.51
41	8	13.63	54.83	31.68	13.64	54.85	31.62	13.64	54.86	31.59
42	6	9.65	55.70	27.16	9.59	55.71	27.23	9.58	55.71	27.24
43	6	9.00	54.33	26.80	8.91	54.34	26.90	8.88	54.35	26.92
44	8	9.49	53.26	27.16	9.27	53.29	27.42	9.22	53.30	27.47
45	7	7.90	54.41	26.00	7.91	54.42	25.97	7.91	54.42	25.97
46	6	7.20	53.25	25.43	7.20	53.25	25.43	7.20	53.25	25.43
47*	6	9.90	47.88	31.09	9.90	47.88	31.09	9.90	47.88	31.09
48*	6	9.41	46.46	30.92	9.41	46.46	30.92	9.41	46.46	30.92
49*	6	10.16	48.22	32.54	10.16	48.22	32.54	10.16	48.22	32.54
50	16	10.59	49.98	32.87	10.60	49.98	32.88	10.60	49.98	32.88
51	6	8.96	50.77	32.60	8.98	50.78	32.60	8.98	50.78	32.63
52	1	11.37	49.87	29.41	11.45	49.87	29.40	11.47	49.87	29.40
53	1	10.10	51.08	29.12	10.14	51.04	29.16	10.15	51.04	29.17
54*	1	10.85	51.40	26.82	10.85	51.40	26.82	10.85	51.40	26.82
55*	1	10.60	49.70	27.10	10.60	49.70	27.10	10.60	49.70	27.10
56	1	12.95	51.99	26.09	12.96	52.01	26.12	12.96	52.01	26.11
57*	1	14.85	49.83	25.80	14.85	49.83	25.80	14.85	49.83	25.80
58	1	14.54	51.85	28.26	14.50	51.98	28.25	14.49	52.02	28.23
59*	1	17.09	53.28	28.31	17.09	53.28	28.31	17.09	53.28	28.31
60*	1	16.04	52.98	29.69	16.04	52.98	29.69	16.04	52.98	29.69
61	1	18.17	51.07	27.61	19.00	52.62	29.17	18.94	52.72	29.43
62	1	19.18	48.97	28.59	20.32	50.44	29.22	20.32	50.51	29.48
63	1	20.32	50.32	28.80	20.28	51.25	30.81	20.18	51.38	31.02
64*	1	14.78	47.62	32.62	14.78	47.62	32.62	14.78	47.62	32.62
65	1	13.06	49.24	31.86	13.07	49.28	31.80	13.07	49.29	31.81
66	1	14.03	49.64	30.46	14.09	49.63	30.42	14.08	49.63	30.42
67	1	14.95	47.19	29.14	14.91	47.25	29.12	14.92	47.24	29.12
68*	1	13.80	44.94	28.50	13.80	44.94	28.50	13.80	44.94	28.50
69*	1	12.41	45.72	29.23	12.41	45.72	29.23	12.41	45.72	29.23
70*	1	13.11	46.37	27.76	13.11	46.37	27.76	13.11	46.37	27.76
71	1	16.70	49.64	33.11	16.70	49.64	33.13	16.70	49.64	33.12
72	1	17.04	52.07	33.77	16.98	52.05	33.90	16.99	52.05	33.88
73	1	15.06	53.54	33.09	15.05	53.54	33.12	15.08	53.54	33.10
74	1	12.65	52.08	34.66	12.77	52.12	34.66	12.85	52.15	34.65
75	1	9.39	55.49	32.11	9.41	55.50	32.24	9.42	55.51	32.28
76	1	9.52	53.92	31.36	9.50	53.94	31.47	9.50	53.94	31.52
77	1	9.81	55.20	34.80	9.92	55.22	34.89	9.99	55.24	34.93
78	1	11.26	53.59	36.16	11.44	53.63	36.19	11.55	53.67	36.21
79	1	11.69	54.48	30.40	11.65	54.49	30.44	11.64	54.49	30.46
80	1	11.58	57.23	31.45	11.56	57.23	31.53	11.55	57.23	31.55
81	1	13.55	58.07	32.36	13.55	58.07	32.37	13.55	58.07	32.38
82	1	14.99	57.05	32.16	15.00	57.05	32.17	15.00	57.05	32.17
83	1	13.95	56.84	33.58	13.94	56.83	33.57	13.94	56.82	33.57
84	1	8.87	56.43	27.40	8.82	56.45	27.45	8.81	56.45	27.46
85	1	7.53	55.33	25.79	7.66	55.32	25.61	7.70	55.32	25.57
86	1	7.72	52.36	25.77	7.55	52.37	25.96	7.49	52.38	26.02
87	1	6.16	53.21	25.77	6.12	53.36	25.58	6.12	53.40	25.51
88	1	7.22	53.29	24.34	7.41	53.13	24.36	7.47	53.09	24.38

89*	1	10.85	47.93	30.60	10.85	47.93	30.60	10.85	47.93	30.60
90*	1	9.10	48.52	30.77	9.10	48.52	30.77	9.10	48.52	30.77
91*	1	8.88	46.16	31.80	8.88	46.16	31.80	8.88	46.16	31.80
92*	1	10.24	45.81	30.77	10.24	45.81	30.77	10.24	45.81	30.77
93*	1	8.75	46.41	30.08	8.75	46.41	30.08	8.75	46.41	30.08
94*	1	9.24	48.04	33.05	9.24	48.04	33.05	9.24	48.04	33.05
95*	1	11.04	47.66	32.81	11.04	47.66	32.81	11.04	47.66	32.81
96	1	9.07	51.83	32.84	9.09	51.83	32.88	9.10	51.83	32.90
97	1	8.21	50.33	33.26	8.22	50.33	33.25	8.22	50.33	33.27
98	1	8.63	50.67	31.56	8.66	50.71	31.56	8.66	50.72	31.58
99	1	19.23	49.85	30.13	19.14	49.93	30.44	19.09	50.02	30.65
100*	1	15.76	47.98	31.21	15.76	47.98	31.21	15.76	47.98	31.21
101	6	14.83	51.46	24.41	14.88	51.48	24.43	14.88	51.49	24.43
102	8	14.49	52.86	24.44	14.48	52.86	24.44	14.46	52.85	24.43
103	6	13.91	50.77	23.43	14.02	50.77	23.40	14.05	50.75	23.39
104	1	14.13	49.71	23.36	14.29	49.71	23.33	14.34	49.70	23.33
105	1	12.86	50.89	23.72	12.96	50.84	23.65	12.98	50.80	23.64
106	1	14.03	51.22	22.44	14.16	51.23	22.42	14.19	51.22	22.41
107	1	15.88	51.34	24.10	15.94	51.39	24.16	15.94	51.42	24.17
108	1	15.28	53.34	24.71	15.21	53.38	24.81	15.15	53.39	24.85
109	6	10.59	56.16	26.03	10.53	56.15	26.08	10.52	56.16	26.10
110	6	11.91	55.42	26.35	11.84	55.43	26.41	11.84	55.45	26.44
111	6	11.98	55.45	27.89	11.92	55.49	27.95	11.92	55.53	27.97
112	1	10.73	57.24	26.09	10.67	57.24	26.13	10.64	57.24	26.14
113	1	10.20	55.92	25.03	10.14	55.91	25.09	10.13	55.90	25.11
114	1	12.78	55.89	25.89	12.71	55.88	25.94	12.71	55.90	25.96
115	1	11.85	54.39	25.99	11.78	54.38	26.09	11.79	54.40	26.12
116	1	12.54	56.32	28.24	12.47	56.38	28.28	12.44	56.44	28.29
117	1	12.43	54.54	28.29	12.39	54.61	28.37	12.41	54.66	28.40

N47P – RED

Atom number	Atomic number	ε = 1			ε = 5.7			ε = 78.4		
		X	Y	Z	X	Y	Z	X	Y	Z
1	29	12.66	52.42	31.52	12.65	52.44	31.55	12.66	52.50	31.55
2	16	12.56	51.94	29.37	12.51	51.98	29.38	12.48	52.02	29.38
3	6	11.20	50.81	28.86	11.20	50.79	28.86	11.19	50.80	28.86
4*	6	11.21	50.54	27.34	11.21	50.54	27.34	11.21	50.54	27.34
5	6	12.60	50.11	26.86	12.59	50.11	26.84	12.61	50.13	26.89
6	8	13.00	48.95	26.94	12.97	48.94	26.87	13.03	48.98	27.05
7	7	13.38	51.11	26.34	13.38	51.11	26.34	13.37	51.10	26.32
8*	6	14.73	50.89	25.85	14.73	50.89	25.85	14.73	50.89	25.85
9	6	15.82	51.52	26.72	15.82	51.52	26.74	15.83	51.49	26.75
10	8	16.97	51.65	26.24	16.98	51.62	26.30	17.00	51.51	26.35
11	7	15.50	51.93	27.95	15.46	52.02	27.94	15.46	52.04	27.93
12*	6	16.49	52.55	28.82	16.48	52.55	28.81	16.48	52.55	28.82
13	6	17.56	51.62	29.44	17.42	51.43	29.32	17.41	51.42	29.32

14	8	17.92	51.79	30.61	17.09	50.25	29.37	17.07	50.24	29.35
15	7	18.11	50.68	28.63	18.63	51.88	29.77	18.61	51.84	29.78
16	6	19.22	49.86	29.08	19.65	51.00	30.30	19.58	50.93	30.37
17*	6	14.76	47.95	31.60	14.76	47.95	31.60	14.76	47.95	31.60
18	6	13.88	46.94	30.86	13.88	46.94	30.86	13.88	46.95	30.86
19	6	14.12	49.35	31.52	14.15	49.36	31.49	14.14	49.36	31.50
20	8	12.94	46.38	31.43	12.95	46.37	31.44	12.93	46.39	31.43
21	7	14.16	46.74	29.54	14.15	46.74	29.54	14.15	46.74	29.54
22	6	14.92	50.43	32.19	14.90	50.42	32.23	14.90	50.43	32.23
23*	6	13.32	45.88	28.69	13.32	45.88	28.69	13.32	45.88	28.69
24	6	16.16	50.38	32.76	16.08	50.35	32.94	16.06	50.35	32.95
25	7	14.45	51.74	32.23	14.46	51.74	32.24	14.47	51.76	32.21
26	7	16.44	51.67	33.17	16.35	51.63	33.37	16.34	51.64	33.37
27	6	15.40	52.45	32.80	15.35	52.44	32.93	15.37	52.45	32.90
28	7	11.71	53.19	33.02	11.77	53.20	33.09	11.80	53.22	33.13
29	6	10.79	54.22	33.03	10.86	54.24	33.15	10.89	54.26	33.23
30	6	12.00	52.93	34.28	12.12	52.94	34.34	12.20	52.95	34.37
31	6	10.15	54.73	31.77	10.15	54.76	31.94	10.14	54.79	32.05
32	6	10.54	54.59	34.32	10.68	54.61	34.47	10.76	54.62	34.55
33	7	11.31	53.76	35.11	11.49	53.77	35.21	11.59	53.78	35.26
34	6	11.14	55.20	30.68	11.09	55.19	30.79	11.04	55.21	30.87
35	7	12.04	56.25	31.18	12.01	56.26	31.22	11.97	56.28	31.26
36	6	10.28	55.65	29.47	10.18	55.58	29.59	10.10	55.56	29.68
37	6	13.26	55.96	31.72	13.26	55.97	31.70	13.22	55.98	31.72
38	8	9.17	56.16	29.67	9.02	55.95	29.79	8.91	55.84	29.89
39	7	10.74	55.40	28.22	10.66	55.41	28.33	10.58	55.46	28.41
40*	6	13.96	57.07	32.51	13.96	57.07	32.51	13.96	57.07	32.51
41	8	13.79	54.85	31.61	13.80	54.87	31.54	13.74	54.87	31.55
42	6	9.85	55.55	27.06	9.77	55.56	27.17	9.68	55.60	27.25
43	6	8.99	54.26	26.85	8.97	54.25	26.89	8.92	54.27	26.94
44	8	9.21	53.22	27.45	9.23	53.19	27.44	9.18	53.21	27.50
45	7	8.03	54.37	25.88	7.99	54.38	25.94	7.96	54.39	25.97
46	6	7.20	53.25	25.43	7.20	53.25	25.43	7.20	53.25	25.43
47*	6	9.90	47.88	31.09	9.90	47.88	31.09	9.90	47.88	31.09
48*	6	9.41	46.46	30.92	9.41	46.46	30.92	9.41	46.46	30.92
49*	6	10.16	48.22	32.54	10.16	48.22	32.54	10.16	48.22	32.54
50	16	10.62	49.96	32.88	10.62	49.96	32.88	10.62	49.96	32.90
51	6	9.08	50.82	32.39	9.08	50.82	32.43	9.03	50.81	32.56
52	1	11.30	49.87	29.40	11.34	49.85	29.39	11.35	49.86	29.39
53	1	10.24	51.26	29.12	10.22	51.19	29.13	10.20	51.18	29.14
54*	1	10.85	51.40	26.82	10.85	51.40	26.82	10.85	51.40	26.82
55*	1	10.60	49.70	27.10	10.60	49.70	27.10	10.60	49.70	27.10
56	1	13.06	52.06	26.40	13.06	52.06	26.42	13.00	52.04	26.23
57*	1	14.85	49.83	25.80	14.85	49.83	25.80	14.85	49.83	25.80
58	1	14.55	51.78	28.34	14.52	51.86	28.33	14.50	51.93	28.30
59*	1	17.09	53.28	28.31	17.09	53.28	28.31	17.09	53.28	28.31
60*	1	16.04	52.98	29.69	16.04	52.98	29.69	16.04	52.98	29.69
61	1	17.94	50.79	27.63	18.87	52.85	29.61	18.86	52.82	29.70
62	1	19.28	48.96	28.46	20.44	50.80	29.57	19.93	50.20	29.63

63	1	20.18	50.39	29.04	20.11	51.44	31.19	20.43	51.50	30.73
64*	1	14.78	47.62	32.62	14.78	47.62	32.62	14.78	47.62	32.62
65	1	13.12	49.31	31.96	13.11	49.34	31.85	13.12	49.33	31.88
66	1	13.97	49.64	30.47	14.08	49.66	30.44	14.06	49.66	30.45
67	1	14.83	47.34	29.09	14.87	47.30	29.11	14.92	47.24	29.12
68*	1	13.80	44.94	28.50	13.80	44.94	28.50	13.80	44.94	28.50
69*	1	12.41	45.72	29.23	12.41	45.72	29.23	12.41	45.72	29.23
70*	1	13.11	46.37	27.76	13.11	46.37	27.76	13.11	46.37	27.76
71	1	16.86	49.57	32.91	16.74	49.52	33.16	16.71	49.52	33.20
72	1	17.36	52.00	33.41	17.15	51.93	33.91	17.14	51.92	33.93
73	1	15.37	53.53	32.93	15.31	53.50	33.09	15.33	53.51	33.07
74	1	12.69	52.17	34.63	12.82	52.17	34.65	12.91	52.19	34.65
75	1	9.45	55.54	32.00	9.50	55.59	32.22	9.51	55.63	32.36
76	1	9.56	53.92	31.32	9.50	53.98	31.54	9.45	54.02	31.68
77	1	9.88	55.33	34.75	10.06	55.36	34.93	10.16	55.37	35.04
78	1	11.34	53.75	36.12	11.58	53.76	36.21	11.73	53.77	36.26
79	1	11.78	54.34	30.44	11.72	54.33	30.55	11.66	54.35	30.62
80	1	11.62	57.14	31.40	11.59	57.14	31.50	11.57	57.16	31.54
81	1	13.54	58.06	32.32	13.56	58.07	32.32	13.55	58.07	32.34
82	1	15.02	57.07	32.25	15.02	57.05	32.27	15.01	57.05	32.24
83	1	13.87	56.85	33.57	13.84	56.85	33.57	13.87	56.84	33.57
84	1	9.18	56.40	27.20	9.07	56.38	27.34	8.94	56.38	27.45
85	1	7.89	55.27	25.45	7.82	55.29	25.55	7.80	55.29	25.56
86	1	7.51	52.38	26.00	7.51	52.36	25.98	7.45	52.37	26.02
87	1	6.14	53.45	25.61	6.13	53.42	25.59	6.13	53.45	25.49
88	1	7.35	53.06	24.36	7.39	53.10	24.36	7.47	53.07	24.38
89*	1	10.85	47.93	30.60	10.85	47.93	30.60	10.85	47.93	30.60
90*	1	9.10	48.52	30.77	9.10	48.52	30.77	9.10	48.52	30.77
91*	1	8.88	46.16	31.80	8.88	46.16	31.80	8.88	46.16	31.80
92*	1	10.24	45.81	30.77	10.24	45.81	30.77	10.24	45.81	30.77
93*	1	8.75	46.41	30.08	8.75	46.41	30.08	8.75	46.41	30.08
94*	1	9.24	48.04	33.05	9.24	48.04	33.05	9.24	48.04	33.05
95*	1	11.04	47.66	32.81	11.04	47.66	32.81	11.04	47.66	32.81
96	1	9.16	51.85	32.75	9.17	51.85	32.78	9.16	51.85	32.86
97	1	8.21	50.35	32.87	8.22	50.35	32.91	8.22	50.36	33.14
98	1	8.95	50.83	31.31	8.93	50.84	31.34	8.78	50.78	31.50
99	1	19.05	49.57	30.12	19.18	50.06	30.57	19.13	50.38	31.21
100*	1	15.76	47.98	31.21	15.76	47.98	31.21	15.76	47.98	31.21
101	6	14.91	51.45	24.41	14.91	51.45	24.42	14.89	51.47	24.43
102	8	14.87	52.88	24.43	14.82	52.88	24.42	14.57	52.87	24.44
103	6	13.83	50.97	23.45	13.88	50.92	23.44	13.99	50.80	23.40
104	1	13.80	49.87	23.42	13.90	49.83	23.40	14.21	49.73	23.34
105	1	12.85	51.33	23.76	12.87	51.23	23.73	12.94	50.93	23.67
106	1	14.04	51.34	22.45	14.08	51.31	22.43	14.15	51.25	22.42
107	1	15.89	51.11	24.07	15.92	51.14	24.09	15.94	51.33	24.15
108	1	15.73	53.16	24.78	15.65	53.20	24.81	15.30	53.34	24.85
109	6	10.85	55.74	25.89	10.77	55.86	26.02	10.65	55.98	26.12
110	6	12.03	54.86	26.32	12.00	55.04	26.42	11.94	55.24	26.49
111	6	12.13	55.09	27.83	12.07	55.22	27.95	12.00	55.35	28.02

112	1	11.15	56.79	25.84	11.00	56.93	26.01	10.82	57.07	26.14
113	1	10.42	55.46	24.92	10.37	55.59	25.04	10.28	55.72	25.13
114	1	12.96	55.08	25.79	12.92	55.37	25.92	12.83	55.65	26.02
115	1	11.78	53.81	26.12	11.84	53.99	26.18	11.86	54.18	26.20
116	1	12.78	55.94	28.06	12.66	56.10	28.22	12.54	56.26	28.34
117	1	12.48	54.20	28.36	12.48	54.33	28.45	12.45	54.47	28.49

N47S – OX

Atom number	Atomic number	ε = 1			ε = 5.7			ε = 78.4		
		X	Y	Z	X	Y	Z	X	Y	Z
1	29	12.54	52.34	31.52	12.55	52.40	31.51	12.57	52.44	31.49
2	16	12.21	52.17	29.38	12.23	52.19	29.36	12.25	52.21	29.34
3	6	11.12	50.77	28.86	11.16	50.77	28.86	11.17	50.78	28.86
4*	6	11.21	50.54	27.34	11.21	50.54	27.34	11.21	50.54	27.34
5	6	12.64	50.14	26.96	12.62	50.13	26.92	12.62	50.13	26.92
6	8	13.09	49.02	27.22	13.05	48.99	27.12	13.06	49.00	27.12
7	7	13.39	51.12	26.38	13.38	51.11	26.36	13.38	51.11	26.35
8*	6	14.73	50.89	25.85	14.73	50.89	25.85	14.73	50.89	25.85
9	6	15.84	51.52	26.70	15.83	51.47	26.75	15.84	51.46	26.76
10	8	16.98	51.64	26.23	17.01	51.43	26.38	17.01	51.40	26.39
11	7	15.50	51.96	27.93	15.46	52.06	27.91	15.46	52.06	27.92
12*	6	16.49	52.55	28.81	16.48	52.55	28.81	16.48	52.55	28.81
13	6	17.46	51.52	29.45	17.36	51.39	29.34	17.37	51.40	29.34
14	8	17.41	51.29	30.66	16.87	50.30	29.65	16.93	50.27	29.53
15	7	18.35	50.95	28.60	18.67	51.71	29.53	18.64	51.77	29.65
16	6	19.32	49.97	29.04	19.64	50.76	30.04	19.61	50.85	30.23
17*	6	14.76	47.95	31.60	14.76	47.95	31.60	14.76	47.95	31.60
18	6	13.85	46.97	30.85	13.88	46.94	30.85	13.88	46.93	30.86
19	6	14.08	49.33	31.51	14.10	49.34	31.48	14.10	49.34	31.47
20	8	12.85	46.51	31.40	12.92	46.40	31.43	12.94	46.38	31.44
21	7	14.16	46.72	29.55	14.15	46.74	29.54	14.15	46.74	29.54
22	6	14.70	50.40	32.36	14.68	50.40	32.36	14.69	50.41	32.34
23*	6	13.32	45.88	28.69	13.32	45.88	28.69	13.32	45.88	28.69
24	6	15.86	50.40	33.08	15.79	50.38	33.16	15.80	50.39	33.14
25	7	14.14	51.68	32.43	14.12	51.68	32.44	14.14	51.70	32.40
26	7	16.01	51.68	33.58	15.90	51.64	33.71	15.92	51.65	33.68
27	6	14.97	52.43	33.16	14.89	52.40	33.25	14.92	52.41	33.21
28	7	11.46	53.15	32.97	11.49	53.18	32.97	11.52	53.21	32.97
29	6	10.50	54.15	32.86	10.51	54.18	32.88	10.54	54.19	32.90
30	6	11.59	52.87	34.25	11.64	52.90	34.26	11.70	52.93	34.26
31	6	10.02	54.72	31.56	10.01	54.75	31.59	10.01	54.76	31.62
32	6	10.06	54.45	34.12	10.09	54.48	34.15	10.15	54.50	34.18
33	7	10.76	53.63	34.99	10.81	53.66	35.00	10.89	53.69	35.02
34	6	11.16	55.29	30.67	11.14	55.31	30.68	11.12	55.32	30.69
35	7	11.97	56.27	31.36	11.97	56.28	31.35	11.97	56.29	31.34
36	6	10.59	55.93	29.40	10.55	55.94	29.41	10.52	55.94	29.42

37	6	13.17	55.94	31.89	13.18	55.95	31.86	13.18	55.96	31.83
38	8	10.30	57.12	29.38	10.22	57.12	29.39	10.15	57.11	29.40
39*	6	13.96	57.07	32.51	13.96	57.07	32.51	13.96	57.07	32.51
40	8	13.59	54.77	31.90	13.62	54.78	31.82	13.63	54.79	31.76
41	7	7.83	54.62	25.89	7.85	54.67	25.84	7.84	54.69	25.85
42	6	6.80	53.70	25.43	6.81	53.78	25.36	6.78	53.81	25.37
43*	6	9.90	47.88	31.09	9.90	47.88	31.09	9.90	47.88	31.09
44*	6	9.41	46.46	30.92	9.41	46.46	30.92	9.41	46.46	30.92
45*	6	10.16	48.22	32.54	10.16	48.22	32.54	10.16	48.22	32.54
46	16	10.57	49.99	32.86	10.57	49.98	32.86	10.58	49.98	32.87
47	6	8.92	50.74	32.58	8.93	50.74	32.60	8.94	50.75	32.61
48	7	10.43	55.06	28.38	10.42	55.07	28.39	10.41	55.07	28.39
49*	6	9.80	55.39	27.12	9.80	55.39	27.12	9.80	55.39	27.12
50	6	8.76	54.29	26.82	8.74	54.32	26.80	8.73	54.33	26.80
51	8	8.81	53.20	27.38	8.74	53.23	27.38	8.73	53.23	27.35
52	6	10.84	55.48	25.99	10.86	55.46	26.00	10.86	55.46	26.00
53	8	11.45	54.21	25.84	11.45	54.18	25.85	11.44	54.17	25.85
54	1	11.41	49.87	29.40	11.48	49.88	29.40	11.49	49.89	29.40
55	1	10.10	51.04	29.13	10.14	51.00	29.17	10.15	51.02	29.17
56*	1	10.85	51.40	26.82	10.85	51.40	26.82	10.85	51.40	26.82
57*	1	10.60	49.70	27.10	10.60	49.70	27.10	10.60	49.70	27.10
58	1	12.97	52.02	26.18	12.97	52.02	26.18	12.97	52.02	26.17
59*	1	14.85	49.83	25.80	14.85	49.83	25.80	14.85	49.83	25.80
60	1	14.54	51.85	28.25	14.50	51.98	28.24	14.50	52.01	28.23
61*	1	17.09	53.28	28.31	17.09	53.28	28.31	17.09	53.28	28.31
62*	1	16.04	52.98	29.69	16.04	52.98	29.69	16.04	52.98	29.69
63	1	18.24	51.17	27.62	19.01	52.57	29.14	18.95	52.70	29.41
64	1	19.07	48.96	28.68	20.26	50.35	29.23	20.32	50.49	29.48
65	1	20.32	50.23	28.66	20.29	51.23	30.77	20.17	51.35	31.03
66*	1	14.78	47.62	32.62	14.78	47.62	32.62	14.78	47.62	32.62
67	1	13.03	49.21	31.80	13.03	49.25	31.69	13.03	49.25	31.71
68	1	14.08	49.67	30.47	14.19	49.68	30.44	14.16	49.67	30.43
69	1	14.96	47.19	29.14	14.91	47.25	29.12	14.92	47.24	29.12
70*	1	13.80	44.94	28.50	13.80	44.94	28.50	13.80	44.94	28.50
71*	1	12.41	45.72	29.23	12.41	45.72	29.23	12.41	45.72	29.23
72*	1	13.11	46.37	27.76	13.11	46.37	27.76	13.11	46.37	27.76
73	1	16.60	49.64	33.23	16.50	49.60	33.38	16.50	49.60	33.37
74	1	16.82	52.03	34.07	16.63	51.96	34.34	16.65	51.97	34.31
75	1	14.85	53.49	33.34	14.75	53.45	33.47	14.78	53.46	33.44
76	1	12.25	52.12	34.67	12.31	52.16	34.67	12.39	52.21	34.66
77	1	9.29	55.51	31.76	9.29	55.54	31.82	9.30	55.56	31.86
78	1	9.51	53.94	30.98	9.47	53.98	31.02	9.46	54.00	31.06
79	1	9.32	55.16	34.47	9.36	55.18	34.51	9.42	55.20	34.55
80	1	10.65	53.59	35.99	10.73	53.63	36.01	10.84	53.67	36.02
81	1	11.82	54.46	30.42	11.78	54.48	30.42	11.76	54.49	30.42
82	1	11.70	57.24	31.23	11.67	57.25	31.31	11.65	57.25	31.36
83	1	13.51	58.05	32.34	13.51	58.05	32.34	13.52	58.05	32.34
84	1	14.97	57.06	32.10	14.98	57.07	32.12	14.99	57.07	32.14
85	1	14.04	56.90	33.59	14.02	56.88	33.58	13.99	56.87	33.58

86	1	7.83	55.56	25.51	7.90	55.60	25.45	7.86	55.64	25.49
87	1	6.94	52.76	25.96	6.91	52.83	25.88	7.02	52.80	25.68
88	1	5.80	54.09	25.64	5.81	54.19	25.55	5.81	54.09	25.79
89	1	6.89	53.51	24.35	6.92	53.60	24.28	6.72	53.86	24.28
90*	1	10.85	47.93	30.60	10.85	47.93	30.60	10.85	47.93	30.60
91*	1	9.10	48.52	30.77	9.10	48.52	30.77	9.10	48.52	30.77
92*	1	8.88	46.16	31.80	8.88	46.16	31.80	8.88	46.16	31.80
93*	1	10.24	45.81	30.77	10.24	45.81	30.77	10.24	45.81	30.77
94*	1	8.75	46.41	30.08	8.75	46.41	30.08	8.75	46.41	30.08
95*	1	9.24	48.04	33.05	9.24	48.04	33.05	9.24	48.04	33.05
96*	1	11.04	47.66	32.81	11.04	47.66	32.81	11.04	47.66	32.81
97	1	9.00	51.80	32.80	9.02	51.81	32.83	9.04	51.81	32.84
98	1	8.19	50.29	33.26	8.19	50.30	33.27	8.20	50.31	33.28
99	1	8.59	50.61	31.55	8.60	50.64	31.56	8.61	50.65	31.57
100	1	19.35	49.96	30.13	19.10	49.94	30.51	19.08	50.00	30.64
101*	1	15.76	47.98	31.20	15.76	47.98	31.20	15.76	47.98	31.20
102	1	10.71	54.09	28.49	10.72	54.11	28.51	10.75	54.12	28.51
103	1	9.32	56.37	27.23	9.34	56.38	27.22	9.34	56.38	27.21
104	1	11.57	56.26	26.26	11.60	56.22	26.27	11.61	56.21	26.28
105	1	10.34	55.80	25.06	10.37	55.79	25.06	10.38	55.79	25.07
106	1	12.16	54.27	25.18	12.16	54.23	25.20	12.16	54.23	25.20
107	6	14.81	51.49	24.42	14.85	51.52	24.44	14.85	51.52	24.44
108	8	14.45	52.89	24.49	14.40	52.89	24.49	14.38	52.88	24.48
109	6	13.87	50.82	23.44	13.99	50.81	23.40	14.02	50.79	23.40
110	1	14.10	49.76	23.34	14.30	49.76	23.30	14.35	49.75	23.30
111	1	12.83	50.92	23.75	12.93	50.84	23.68	12.96	50.80	23.66
112	1	13.97	51.29	22.45	14.11	51.30	22.43	14.13	51.28	22.42
113	1	15.84	51.39	24.09	15.90	51.48	24.15	15.91	51.49	24.16
114	1	15.24	53.37	24.76	15.12	53.42	24.87	15.07	53.43	24.89

N47S – RED

Atom number	Atomic number	ε = 1			ε = 5.7			ε = 78.4		
		X	Y	Z	X	Y	Z	X	Y	Z
1	29	12.64	52.55	31.46	12.62	52.59	31.47	12.64	52.65	31.48
2	16	12.38	52.23	29.24	12.34	52.26	29.25	12.33	52.26	29.26
3	6	11.21	50.85	28.85	11.21	50.85	28.85	11.21	50.84	28.85
4*	6	11.21	50.54	27.34	11.21	50.54	27.34	11.21	50.54	27.34
5	6	12.61	50.12	26.89	12.61	50.12	26.88	12.61	50.13	26.89
6	8	13.03	48.96	27.02	13.01	48.96	27.01	13.03	48.98	27.05
7	7	13.38	51.11	26.35	13.38	51.10	26.33	13.37	51.10	26.33
8*	6	14.73	50.89	25.85	14.73	50.89	25.85	14.73	50.89	25.85
9	6	15.83	51.51	26.72	15.83	51.48	26.76	15.83	51.47	26.77
10	8	16.98	51.60	26.26	17.00	51.49	26.37	17.01	51.43	26.39
11	7	15.49	51.94	27.95	15.45	52.04	27.94	15.45	52.05	27.93
12*	6	16.48	52.55	28.82	16.48	52.55	28.81	16.48	52.55	28.81
13	6	17.54	51.60	29.46	17.40	51.41	29.33	17.39	51.41	29.33

14	8	17.86	51.76	30.64	16.99	50.27	29.51	16.99	50.25	29.46
15	7	18.11	50.68	28.65	18.66	51.82	29.64	18.63	51.80	29.70
16	6	19.19	49.83	29.13	19.67	50.91	30.16	19.60	50.88	30.27
17*	6	14.76	47.95	31.60	14.76	47.95	31.60	14.76	47.95	31.60
18	6	13.89	46.93	30.86	13.90	46.92	30.86	13.89	46.93	30.86
19	6	14.10	49.34	31.49	14.12	49.35	31.47	14.12	49.35	31.47
20	8	12.97	46.34	31.44	12.99	46.32	31.45	12.95	46.36	31.44
21	7	14.16	46.74	29.54	14.15	46.75	29.54	14.15	46.74	29.54
22	6	14.85	50.44	32.18	14.78	50.43	32.27	14.79	50.44	32.27
23*	6	13.32	45.88	28.69	13.32	45.88	28.69	13.32	45.88	28.69
24	6	16.06	50.40	32.82	15.89	50.36	33.08	15.89	50.36	33.09
25	7	14.37	51.75	32.20	14.32	51.74	32.25	14.33	51.75	32.24
26	7	16.32	51.69	33.24	16.10	51.64	33.55	16.10	51.64	33.56
27	6	15.29	52.47	32.82	15.13	52.43	33.03	15.15	52.44	33.03
28	7	11.49	53.20	32.90	11.48	53.26	32.93	11.49	53.29	32.95
29	6	10.50	54.19	32.86	10.50	54.24	32.90	10.50	54.27	32.92
30	6	11.66	52.90	34.18	11.67	52.97	34.21	11.69	53.01	34.23
31	6	10.01	54.76	31.57	9.99	54.81	31.61	9.97	54.84	31.64
32	6	10.09	54.47	34.14	10.11	54.53	34.18	10.13	54.57	34.21
33	7	10.83	53.64	34.96	10.86	53.71	35.00	10.89	53.75	35.02
34	6	11.17	55.25	30.66	11.13	55.29	30.68	11.11	55.31	30.70
35	7	12.00	56.25	31.32	11.99	56.27	31.32	11.98	56.29	31.32
36	6	10.59	55.87	29.39	10.55	55.89	29.41	10.51	55.90	29.42
37	6	13.21	55.93	31.84	13.21	55.95	31.82	13.21	55.96	31.80
38	8	10.25	57.06	29.37	10.17	57.07	29.38	10.11	57.07	29.40
39*	6	13.96	57.07	32.51	13.96	57.07	32.51	13.96	57.07	32.51
40	8	13.68	54.79	31.82	13.68	54.80	31.75	13.68	54.81	31.70
41	7	7.78	54.83	25.86	7.78	54.85	25.86	7.80	54.81	25.85
42	6	6.68	54.01	25.38	6.66	54.06	25.38	6.68	54.01	25.39
43*	6	9.90	47.88	31.09	9.90	47.88	31.09	9.90	47.88	31.09
44*	6	9.41	46.46	30.92	9.41	46.46	30.92	9.41	46.46	30.92
45*	6	10.16	48.22	32.54	10.16	48.22	32.54	10.16	48.22	32.54
46	16	10.60	49.97	32.87	10.60	49.97	32.87	10.60	49.97	32.88
47	6	9.05	50.79	32.37	9.04	50.80	32.41	8.99	50.78	32.56
48	7	10.46	55.01	28.35	10.44	55.03	28.37	10.44	55.04	28.38
49*	6	9.80	55.39	27.12	9.80	55.39	27.12	9.80	55.39	27.12
50	6	8.66	54.40	26.81	8.65	54.41	26.81	8.66	54.40	26.80
51	8	8.56	53.32	27.37	8.55	53.32	27.36	8.57	53.30	27.36
52	6	10.81	55.45	25.96	10.82	55.43	25.97	10.83	55.45	25.97
53	8	11.31	54.15	25.72	11.32	54.12	25.75	11.35	54.14	25.76
54	1	11.49	49.96	29.42	11.52	49.96	29.41	11.54	49.95	29.40
55	1	10.20	51.15	29.15	10.19	51.11	29.16	10.19	51.07	29.17
56*	1	10.85	51.40	26.82	10.85	51.40	26.82	10.85	51.40	26.82
57*	1	10.60	49.70	27.10	10.60	49.70	27.10	10.60	49.70	27.10
58	1	13.02	52.05	26.35	13.00	52.04	26.30	12.99	52.04	26.24
59*	1	14.85	49.83	25.80	14.85	49.83	25.80	14.85	49.83	25.80
60	1	14.53	51.87	28.30	14.49	51.96	28.29	14.48	52.00	28.27
61*	1	17.09	53.28	28.31	17.09	53.28	28.31	17.09	53.28	28.31
62*	1	16.04	52.98	29.69	16.04	52.98	29.69	16.04	52.98	29.69

63	1	17.96	50.79	27.65	18.96	52.74	29.35	18.93	52.75	29.50
64	1	19.26	48.94	28.51	20.38	50.60	29.38	19.99	50.18	29.52
65	1	20.16	50.35	29.10	20.23	51.39	30.97	20.43	51.45	30.69
66*	1	14.78	47.62	32.62	14.78	47.62	32.62	14.78	47.62	32.62
67	1	13.08	49.29	31.89	13.06	49.29	31.76	13.07	49.29	31.77
68	1	13.99	49.62	30.43	14.12	49.65	30.41	14.11	49.65	30.42
69	1	14.81	47.36	29.08	14.84	47.33	29.09	14.90	47.26	29.11
70*	1	13.80	44.94	28.50	13.80	44.94	28.50	13.80	44.94	28.50
71*	1	12.41	45.72	29.23	12.41	45.72	29.23	12.41	45.72	29.23
72*	1	13.11	46.37	27.76	13.11	46.37	27.76	13.11	46.37	27.76
73	1	16.75	49.60	32.99	16.54	49.54	33.34	16.52	49.53	33.37
74	1	17.21	52.03	33.55	16.84	51.93	34.17	16.84	51.93	34.19
75	1	15.24	53.54	32.96	15.06	53.50	33.21	15.09	53.50	33.21
76	1	12.34	52.16	34.56	12.36	52.24	34.59	12.40	52.29	34.61
77	1	9.31	55.58	31.77	9.30	55.64	31.82	9.30	55.68	31.86
78	1	9.47	53.99	31.00	9.42	54.05	31.06	9.39	54.09	31.10
79	1	9.36	55.16	34.52	9.38	55.23	34.57	9.40	55.26	34.60
80	1	10.76	53.58	35.97	10.81	53.67	36.01	10.86	53.72	36.03
81	1	11.79	54.38	30.47	11.75	54.41	30.49	11.72	54.43	30.50
82	1	11.69	57.21	31.24	11.67	57.23	31.33	11.65	57.24	31.39
83	1	13.51	58.05	32.33	13.51	58.05	32.34	13.51	58.06	32.35
84	1	14.99	57.07	32.15	14.99	57.08	32.16	14.99	57.08	32.16
85	1	13.98	56.89	33.59	13.97	56.87	33.58	13.97	56.86	33.58
86	1	7.91	55.75	25.46	7.91	55.78	25.48	7.88	55.76	25.50
87	1	6.72	53.07	25.93	6.72	53.09	25.85	6.93	52.95	25.53
88	1	5.71	54.49	25.58	5.71	54.53	25.64	5.76	54.22	25.96
89	1	6.76	53.81	24.31	6.71	53.94	24.29	6.49	54.20	24.34
90*	1	10.85	47.93	30.60	10.85	47.93	30.60	10.85	47.93	30.60
91*	1	9.10	48.52	30.77	9.10	48.52	30.77	9.10	48.52	30.77
92*	1	8.88	46.16	31.80	8.88	46.16	31.80	8.88	46.16	31.80
93*	1	10.24	45.81	30.77	10.24	45.81	30.77	10.24	45.81	30.77
94*	1	8.75	46.41	30.08	8.75	46.41	30.08	8.75	46.41	30.08
95*	1	9.24	48.04	33.05	9.24	48.04	33.05	9.24	48.04	33.05
96*	1	11.04	47.66	32.81	11.04	47.66	32.81	11.04	47.66	32.81
97	1	9.11	51.83	32.69	9.13	51.84	32.72	9.12	51.84	32.78
98	1	8.19	50.32	32.85	8.19	50.34	32.93	8.21	50.37	33.22
99	1	8.91	50.77	31.28	8.88	50.76	31.33	8.68	50.67	31.52
100	1	18.99	49.55	30.16	19.16	50.03	30.54	19.13	50.30	31.07
101*	1	15.76	47.98	31.21	15.76	47.98	31.21	15.76	47.98	31.21
102	1	10.86	54.07	28.44	10.85	54.09	28.47	10.86	54.11	28.48
103	1	9.40	56.40	27.27	9.40	56.40	27.25	9.39	56.40	27.24
104	1	11.61	56.15	26.24	11.63	56.13	26.25	11.62	56.15	26.25
105	1	10.32	55.86	25.06	10.34	55.83	25.06	10.34	55.83	25.06
106	1	12.11	54.20	25.17	12.08	54.16	25.16	12.09	54.19	25.14
107	6	14.86	51.49	24.43	14.87	51.51	24.44	14.87	51.51	24.44
108	8	14.57	52.91	24.49	14.48	52.90	24.48	14.43	52.88	24.47
109	6	13.89	50.88	23.43	14.00	50.83	23.40	14.03	50.80	23.39
110	1	14.06	49.80	23.34	14.27	49.77	23.30	14.34	49.75	23.30
111	1	12.85	51.03	23.74	12.94	50.89	23.68	12.97	50.82	23.65

112	1	14.03	51.34	22.45	14.13	51.31	22.43	14.16	51.28	22.42
113	1	15.89	51.34	24.10	15.92	51.43	24.16	15.93	51.47	24.17
114	1	15.36	53.33	24.86	15.18	53.39	24.93	15.11	53.41	24.90

F114N – OX

Atom number	Atomic number	ε = 1			ε = 5.7			ε = 78.4		
		X	Y	Z	X	Y	Z	X	Y	Z
1	29	12.25	52.00	31.71	12.29	52.11	31.69	12.30	52.18	31.66
2	16	12.17	52.02	29.54	12.21	52.04	29.51	12.24	52.06	29.48
3	6	11.09	50.69	28.86	11.12	50.70	28.87	11.13	50.71	28.87
4*	6	11.21	50.54	27.34	11.21	50.54	27.34	11.21	50.54	27.34
5	6	12.64	50.14	26.94	12.63	50.14	26.91	12.63	50.15	26.91
6	8	13.08	49.02	27.18	13.05	49.00	27.09	13.06	49.01	27.11
7	7	13.39	51.13	26.36	13.39	51.13	26.36	13.38	51.13	26.34
8*	6	14.73	50.89	25.85	14.73	50.89	25.85	14.73	50.89	25.85
9	6	15.83	51.53	26.71	15.84	51.50	26.72	15.83	51.49	26.73
10	8	16.99	51.62	26.29	17.00	51.55	26.31	17.01	51.49	26.34
11	7	15.46	52.06	27.90	15.47	52.05	27.90	15.47	52.06	27.90
12*	6	16.49	52.55	28.81	16.48	52.55	28.82	16.48	52.55	28.81
13	6	17.36	51.35	29.25	17.35	51.35	29.27	17.35	51.34	29.25
14	8	16.81	50.26	29.47	16.80	50.26	29.49	16.78	50.27	29.52
15*	6	14.76	47.95	31.60	14.76	47.95	31.60	14.76	47.95	31.60
16	6	13.81	47.00	30.83	13.84	46.97	30.84	13.85	46.96	30.84
17	6	14.14	49.34	31.47	14.13	49.34	31.46	14.12	49.34	31.47
18	8	12.75	46.66	31.36	12.82	46.54	31.40	12.85	46.51	31.41
19	7	14.16	46.70	29.56	14.15	46.71	29.56	14.15	46.72	29.55
20	6	14.69	50.42	32.35	14.67	50.41	32.37	14.63	50.39	32.41
21*	6	13.32	45.88	28.69	13.32	45.88	28.69	13.32	45.88	28.69
22	6	15.94	50.60	32.89	15.88	50.54	33.00	15.78	50.47	33.14
23	7	13.93	51.55	32.63	13.93	51.57	32.61	13.91	51.57	32.61
24	7	15.91	51.83	33.51	15.85	51.76	33.63	15.75	51.69	33.78
25	6	14.70	52.38	33.32	14.68	52.36	33.37	14.63	52.33	33.44
26	7	11.31	53.12	33.03	11.33	53.15	33.06	11.32	53.17	33.04
27	6	10.45	54.19	32.80	10.44	54.20	32.89	10.43	54.23	32.88
28	6	11.40	52.96	34.34	11.46	52.95	34.36	11.44	52.96	34.35
29	6	10.05	54.68	31.44	10.00	54.73	31.55	9.99	54.76	31.55
30	6	10.03	54.67	34.02	10.05	54.63	34.13	10.03	54.64	34.12
31	7	10.64	53.88	34.97	10.70	53.83	35.04	10.68	53.83	35.03
32	6	11.24	55.24	30.60	11.17	55.26	30.68	11.16	55.27	30.67
33	7	12.00	56.24	31.31	11.98	56.24	31.36	11.99	56.25	31.35
34	6	10.73	55.87	29.30	10.63	55.88	29.39	10.64	55.89	29.37
35	6	13.20	55.93	31.88	13.19	55.93	31.89	13.18	55.93	31.89
36	8	10.48	57.08	29.24	10.27	57.06	29.37	10.30	57.08	29.34
37	7	10.59	55.01	28.27	10.58	55.05	28.33	10.57	55.05	28.32
38*	6	13.96	57.07	32.51	13.96	57.07	32.51	13.96	57.07	32.51
39	8	13.64	54.78	31.90	13.64	54.78	31.88	13.62	54.77	31.89

40	6	9.96	55.37	27.02	9.98	55.42	27.06	10.01	55.44	27.04
41	6	9.04	54.20	26.60	9.05	54.27	26.60	9.07	54.31	26.55
42	6	11.00	55.79	25.95	11.05	55.83	26.02	11.11	55.85	26.03
43	8	8.93	53.19	27.30	8.87	53.27	27.30	8.85	53.31	27.22
44	7	8.34	54.39	25.46	8.44	54.46	25.41	8.48	54.53	25.35
45	6	12.00	54.68	25.62	12.02	54.71	25.68	12.08	54.71	25.71
46	6	7.39	53.42	24.94	7.49	53.50	24.86	7.52	53.60	24.79
47	7	13.09	55.03	24.91	13.05	55.03	24.88	13.23	55.06	25.09
48	8	11.80	53.52	26.00	11.85	53.57	26.12	11.81	53.54	25.99
49*	6	9.90	47.88	31.09	9.90	47.88	31.09	9.90	47.88	31.09
50*	6	9.41	46.46	30.92	9.41	46.46	30.92	9.41	46.46	30.92
51*	6	10.16	48.22	32.54	10.16	48.22	32.54	10.16	48.22	32.54
52	16	10.53	50.00	32.88	10.55	49.99	32.87	10.55	49.99	32.87
53	6	8.88	50.72	32.57	8.90	50.73	32.59	8.90	50.73	32.61
54	1	11.35	49.75	29.35	11.40	49.77	29.35	11.43	49.78	29.36
55	1	10.05	50.94	29.12	10.09	50.94	29.15	10.11	50.95	29.16
56*	1	10.85	51.40	26.82	10.85	51.40	26.82	10.85	51.40	26.82
57*	1	10.60	49.70	27.10	10.60	49.70	27.10	10.60	49.70	27.10
58	1	12.94	52.03	26.17	12.96	52.05	26.21	12.94	52.04	26.17
59*	1	14.85	49.83	25.80	14.85	49.83	25.80	14.85	49.83	25.80
60	1	14.54	51.85	28.27	14.53	51.89	28.25	14.51	51.94	28.23
61*	1	17.09	53.28	28.31	17.09	53.28	28.31	17.09	53.28	28.31
62	1	19.60	49.73	28.70	19.58	49.72	28.69	19.53	49.67	28.62
63*	1	14.78	47.62	32.62	14.78	47.62	32.62	14.78	47.62	32.62
64	1	13.07	49.23	31.68	13.06	49.23	31.65	13.05	49.22	31.63
65	1	14.22	49.67	30.42	14.23	49.68	30.42	14.25	49.70	30.44
66	1	15.06	47.03	29.23	15.02	47.09	29.20	15.02	47.10	29.19
67*	1	13.80	44.94	28.50	13.80	44.94	28.50	13.80	44.94	28.50
68*	1	12.41	45.72	29.23	12.41	45.72	29.23	12.41	45.72	29.23
69*	1	13.11	46.37	27.76	13.11	46.37	27.76	13.11	46.37	27.76
70	1	16.84	50.02	32.84	16.76	49.91	33.01	16.63	49.80	33.22
71	1	16.73	52.34	33.80	16.64	52.20	34.08	16.49	52.07	34.36
72	1	14.44	53.39	33.59	14.43	53.37	33.66	14.38	53.34	33.74
73	1	11.97	52.20	34.84	12.07	52.19	34.83	12.05	52.21	34.82
74	1	9.30	55.46	31.56	9.28	55.53	31.72	9.28	55.57	31.71
75	1	9.59	53.87	30.86	9.49	53.94	30.99	9.47	53.98	30.98
76	1	9.37	55.48	34.27	9.37	55.41	34.43	9.35	55.42	34.42
77	1	10.53	53.96	35.97	10.61	53.87	36.05	10.59	53.88	36.04
78	1	11.91	54.41	30.39	11.81	54.41	30.46	11.80	54.42	30.46
79	1	11.72	57.20	31.16	11.67	57.20	31.30	11.69	57.22	31.28
80	1	10.79	54.02	28.40	10.87	54.08	28.43	10.86	54.09	28.43
81	1	13.52	58.05	32.31	13.51	58.05	32.33	13.54	58.05	32.27
82	1	14.99	57.04	32.14	14.98	57.06	32.11	14.99	57.02	32.17
83	1	14.00	56.92	33.59	14.02	56.91	33.59	13.96	56.94	33.59
84	1	9.33	56.25	27.22	9.36	56.30	27.25	9.40	56.33	27.22
85	1	11.53	56.67	26.32	11.61	56.68	26.43	11.66	56.69	26.45
86	1	10.50	56.10	25.03	10.58	56.20	25.10	10.67	56.21	25.10
87	1	8.41	55.29	24.99	8.53	55.35	24.94	8.60	55.43	24.89
88	1	7.50	52.51	25.52	7.75	52.51	25.22	7.89	52.58	24.90

89	1	6.36	53.78	25.03	6.46	53.73	25.17	6.54	53.67	25.29
90	1	7.60	53.20	23.89	7.54	53.52	23.77	7.39	53.82	23.73
91	1	13.76	54.30	24.65	13.72	54.30	24.62	13.88	54.33	24.82
92	1	13.22	55.97	24.57	13.17	55.96	24.50	13.43	56.02	24.86
93*	1	10.85	47.93	30.60	10.85	47.93	30.60	10.85	47.93	30.60
94*	1	9.10	48.52	30.77	9.10	48.52	30.77	9.10	48.52	30.77
95*	1	8.88	46.16	31.80	8.88	46.16	31.80	8.88	46.16	31.80
96*	1	10.24	45.81	30.77	10.24	45.81	30.77	10.24	45.81	30.77
97*	1	8.75	46.41	30.08	8.75	46.41	30.08	8.75	46.41	30.08
98*	1	9.24	48.04	33.05	9.24	48.04	33.05	9.24	48.04	33.05
99*	1	11.04	47.66	32.81	11.04	47.66	32.81	11.04	47.66	32.81
100	1	8.93	51.78	32.83	8.97	51.79	32.83	8.97	51.79	32.84
101	1	8.14	50.23	33.21	8.16	50.26	33.25	8.17	50.26	33.28
102	1	8.59	50.62	31.52	8.59	50.62	31.55	8.57	50.61	31.58
103	6	14.92	51.42	24.40	14.92	51.42	24.40	14.95	51.42	24.41
104	8	15.06	52.86	24.39	15.02	52.85	24.38	15.10	52.85	24.39
105	6	13.78	51.05	23.47	13.81	51.01	23.46	13.82	51.06	23.46
106	1	13.64	49.97	23.45	13.69	49.92	23.45	13.66	49.98	23.45
107	1	12.84	51.52	23.78	12.85	51.45	23.76	12.88	51.54	23.75
108	1	14.01	51.39	22.45	14.04	51.34	22.44	14.07	51.38	22.44
109	1	15.86	50.99	24.04	15.87	51.00	24.05	15.88	50.97	24.06
110	1	15.94	53.04	24.75	15.89	53.08	24.74	15.97	53.05	24.75
111	7	18.69	51.51	29.36	18.69	51.50	29.36	18.69	51.47	29.29
112	6	19.56	50.34	29.62	19.56	50.33	29.61	19.54	50.28	29.52
113	6	19.08	49.48	30.79	19.09	49.46	30.78	19.10	49.44	30.72
114	8	18.94	49.93	31.92	18.97	49.90	31.92	19.01	49.90	31.86
115	6	20.93	50.99	29.90	20.93	50.96	29.87	20.94	50.90	29.73
116	6	20.93	52.22	29.00	20.92	52.21	28.97	20.92	52.12	28.80
117	6	19.48	52.73	29.08	19.48	52.72	29.09	19.49	52.67	28.95
118	7	18.91	48.16	30.48	18.90	48.15	30.46	18.89	48.13	30.43
119	6	18.51	47.18	31.47	18.50	47.16	31.43	18.50	47.16	31.42
120	6	17.00	47.03	31.75	17.00	47.02	31.71	16.99	47.01	31.69
121	8	16.63	46.16	32.51	16.61	46.11	32.44	16.60	46.09	32.41
122	7	16.15	47.92	31.14	16.15	47.93	31.15	16.16	47.94	31.15
123	6	15.80	53.24	30.02	15.79	53.25	30.00	15.80	53.25	30.01
124	1	14.97	53.84	29.64	14.99	53.88	29.61	15.04	53.93	29.61
125	1	15.38	52.49	30.69	15.34	52.51	30.66	15.30	52.52	30.63
126	1	21.64	52.99	29.31	21.65	52.96	29.28	21.66	52.87	29.07
127	1	19.35	53.44	29.90	19.37	53.42	29.92	19.41	53.40	29.76
128	1	21.76	50.30	29.70	21.75	50.27	29.65	21.73	50.19	29.50
129	1	20.96	51.27	30.96	20.99	51.24	30.92	21.03	51.20	30.78
130	1	18.89	46.20	31.19	18.87	46.18	31.14	18.88	46.17	31.15
131	1	18.97	47.45	32.43	18.96	47.41	32.39	18.95	47.43	32.38
132	1	18.92	47.89	29.51	18.92	47.88	29.49	18.90	47.84	29.46
133	1	16.51	48.69	30.58	16.52	48.70	30.59	16.52	48.71	30.60
134	1	19.15	53.19	28.14	19.14	53.20	28.17	19.12	53.12	28.03
135	1	21.17	51.94	27.97	21.14	51.93	27.93	21.10	51.82	27.77
136	6	16.76	54.13	30.81	16.77	54.10	30.82	16.81	54.01	30.87
137	7	16.92	55.39	30.31	16.88	55.39	30.42	16.90	55.34	30.63

138	8	17.37	53.74	31.80	17.44	53.63	31.73	17.52	53.43	31.69
139	1	17.58	56.00	30.77	17.53	55.99	30.91	17.57	55.88	31.15
140	1	16.40	55.73	29.52	16.29	55.80	29.71	16.30	55.82	29.98

F114N – RED

Atom number	Atomic number	ε = 1			ε = 5.7			ε = 78.4		
		X	Y	Z	X	Y	Z	X	Y	Z
1	29	12.24	52.14	31.73	12.28	52.27	31.69	12.29	52.32	31.69
2	16	12.32	52.05	29.46	12.33	52.09	29.43	12.31	52.10	29.42
3	6	11.16	50.75	28.87	11.17	50.77	28.86	11.17	50.77	28.86
4*	6	11.21	50.54	27.34	11.21	50.54	27.34	11.21	50.54	27.34
5	6	12.62	50.13	26.91	12.62	50.14	26.90	12.62	50.15	26.90
6	8	13.07	49.00	27.10	13.06	48.99	27.08	13.07	49.01	27.11
7	7	13.37	51.12	26.32	13.37	51.12	26.31	13.37	51.12	26.31
8*	6	14.73	50.89	25.85	14.73	50.89	25.85	14.73	50.89	25.85
9	6	15.81	51.51	26.75	15.81	51.51	26.74	15.81	51.52	26.74
10	8	16.98	51.58	26.33	16.98	51.56	26.32	16.98	51.55	26.32
11	7	15.44	52.05	27.93	15.44	52.05	27.92	15.44	52.06	27.91
12*	6	16.49	52.55	28.82	16.49	52.55	28.82	16.48	52.55	28.82
13	6	17.36	51.33	29.21	17.36	51.33	29.20	17.35	51.32	29.19
14	8	16.79	50.31	29.62	16.78	50.33	29.66	16.77	50.33	29.68
15*	6	14.76	47.95	31.60	14.76	47.95	31.60	14.76	47.95	31.60
16	6	13.83	46.97	30.84	13.85	46.95	30.85	13.86	46.95	30.85
17	6	14.15	49.35	31.47	14.12	49.34	31.49	14.12	49.34	31.49
18	8	12.81	46.55	31.40	12.87	46.48	31.42	12.87	46.47	31.42
19	7	14.16	46.71	29.56	14.15	46.72	29.55	14.15	46.72	29.55
20	6	14.67	50.37	32.42	14.58	50.33	32.52	14.57	50.33	32.53
21*	6	13.32	45.88	28.69	13.32	45.88	28.69	13.32	45.88	28.69
22	6	15.86	50.44	33.10	15.61	50.26	33.42	15.57	50.24	33.46
23	7	13.91	51.50	32.71	13.93	51.55	32.67	13.93	51.56	32.66
24	7	15.81	51.61	33.83	15.58	51.44	34.13	15.54	51.43	34.16
25	6	14.63	52.22	33.57	14.56	52.19	33.65	14.55	52.19	33.65
26	7	11.16	53.10	33.08	11.18	53.17	33.05	11.18	53.21	33.05
27	6	10.28	54.17	32.95	10.28	54.23	32.93	10.28	54.27	32.94
28	6	11.25	52.85	34.37	11.27	52.92	34.35	11.28	52.96	34.35
29	6	9.92	54.73	31.61	9.90	54.80	31.60	9.88	54.84	31.61
30	6	9.86	54.56	34.20	9.85	54.61	34.18	9.86	54.64	34.19
31	7	10.48	53.71	35.09	10.49	53.76	35.07	10.50	53.80	35.07
32	6	11.18	55.12	30.81	11.15	55.18	30.77	11.12	55.22	30.77
33	7	11.98	56.14	31.47	11.97	56.18	31.44	11.95	56.21	31.43
34	6	10.78	55.70	29.45	10.73	55.77	29.43	10.69	55.82	29.43
35	6	13.15	55.88	32.07	13.15	55.90	32.00	13.15	55.92	31.97
36	8	10.53	56.91	29.35	10.42	56.97	29.34	10.32	57.00	29.37
37	7	10.70	54.82	28.43	10.69	54.90	28.39	10.70	54.97	28.38
38*	6	13.96	57.07	32.51	13.96	57.07	32.51	13.96	57.07	32.51
39	8	13.58	54.73	32.26	13.59	54.74	32.12	13.59	54.75	32.03

40	6	10.17	55.22	27.15	10.17	55.32	27.10	10.20	55.39	27.08
41	6	9.25	54.10	26.61	9.23	54.22	26.55	9.23	54.33	26.52
42	6	11.27	55.71	26.19	11.30	55.77	26.15	11.34	55.82	26.13
43	8	8.93	53.15	27.31	8.92	53.24	27.22	8.90	53.34	27.16
44	7	8.72	54.32	25.37	8.70	54.48	25.33	8.70	54.62	25.30
45	6	12.20	54.60	25.71	12.23	54.64	25.71	12.26	54.67	25.71
46	6	7.93	53.28	24.71	7.83	53.51	24.66	7.74	53.73	24.67
47	7	13.47	54.95	25.42	13.49	54.98	25.39	13.52	55.00	25.37
48	8	11.78	53.45	25.56	11.81	53.48	25.62	11.84	53.50	25.66
49*	6	9.90	47.88	31.09	9.90	47.88	31.09	9.90	47.88	31.09
50*	6	9.41	46.46	30.92	9.41	46.46	30.92	9.41	46.46	30.92
51*	6	10.16	48.22	32.54	10.16	48.22	32.54	10.16	48.22	32.54
52	16	10.55	49.99	32.84	10.57	49.99	32.84	10.56	49.99	32.85
53	6	8.92	50.71	32.43	8.93	50.72	32.50	8.90	50.71	32.61
54	1	11.40	49.81	29.36	11.43	49.83	29.37	11.44	49.83	29.37
55	1	10.14	51.03	29.14	10.15	51.04	29.15	10.14	51.01	29.16
56*	1	10.85	51.40	26.82	10.85	51.40	26.82	10.85	51.40	26.82
57*	1	10.60	49.70	27.10	10.60	49.70	27.10	10.60	49.70	27.10
58	1	12.92	52.01	26.12	12.93	52.02	26.13	12.92	52.02	26.12
59*	1	14.85	49.83	25.80	14.85	49.83	25.80	14.85	49.83	25.80
60	1	14.50	51.89	28.30	14.49	51.92	28.29	14.49	51.94	28.28
61*	1	17.09	53.28	28.31	17.09	53.28	28.31	17.09	53.28	28.31
62	1	19.36	49.51	28.42	19.18	49.43	28.24	19.13	49.39	28.17
63*	1	14.78	47.62	32.62	14.78	47.62	32.62	14.78	47.62	32.62
64	1	13.07	49.26	31.63	13.04	49.22	31.60	13.03	49.21	31.60
65	1	14.26	49.71	30.44	14.28	49.75	30.49	14.28	49.76	30.49
66	1	14.99	47.14	29.18	14.98	47.16	29.17	14.99	47.14	29.18
67*	1	13.80	44.94	28.50	13.80	44.94	28.50	13.80	44.94	28.50
68*	1	12.41	45.72	29.23	12.41	45.72	29.23	12.41	45.72	29.23
69*	1	13.11	46.37	27.76	13.11	46.37	27.76	13.11	46.37	27.76
70	1	16.74	49.82	33.09	16.36	49.50	33.61	16.29	49.47	33.68
71	1	16.56	51.99	34.38	16.23	51.72	34.85	16.16	51.70	34.91
72	1	14.34	53.18	33.97	14.32	53.18	34.00	14.31	53.19	34.00
73	1	11.83	52.06	34.82	11.87	52.15	34.80	11.89	52.19	34.80
74	1	9.26	55.60	31.73	9.25	55.66	31.74	9.23	55.70	31.76
75	1	9.38	53.98	31.03	9.35	54.05	31.02	9.32	54.09	31.03
76	1	9.18	55.34	34.52	9.17	55.37	34.50	9.17	55.41	34.52
77	1	10.37	53.70	36.09	10.39	53.76	36.07	10.41	53.81	36.08
78	1	11.77	54.20	30.73	11.74	54.27	30.69	11.71	54.31	30.67
79	1	11.72	57.09	31.22	11.69	57.15	31.29	11.66	57.18	31.34
80	1	11.01	53.84	28.59	11.01	53.94	28.55	11.05	54.02	28.52
81	1	13.45	58.03	32.36	13.45	58.03	32.38	13.45	58.03	32.41
82	1	14.90	57.08	31.95	14.91	57.10	31.97	14.91	57.11	31.97
83	1	14.21	56.97	33.57	14.18	56.92	33.57	14.18	56.89	33.56
84	1	9.53	56.09	27.35	9.56	56.20	27.29	9.61	56.30	27.27
85	1	11.83	56.51	26.71	11.87	56.56	26.65	11.92	56.61	26.61
86	1	10.83	56.19	25.31	10.88	56.23	25.25	10.93	56.26	25.21
87	1	9.17	54.99	24.78	9.05	55.24	24.78	8.96	55.47	24.83
88	1	7.37	52.75	25.48	7.15	53.08	25.39	8.15	52.73	24.55

89	1	7.23	53.75	24.01	7.25	54.03	23.89	6.82	53.65	25.27
90	1	8.56	52.57	24.18	8.40	52.70	24.20	7.48	54.13	23.69
91	1	14.11	54.24	25.05	14.12	54.26	25.03	14.14	54.27	25.02
92	1	13.81	55.88	25.59	13.81	55.93	25.47	13.84	55.95	25.41
93*	1	10.85	47.93	30.60	10.85	47.93	30.60	10.85	47.93	30.60
94*	1	9.10	48.52	30.77	9.10	48.52	30.77	9.10	48.52	30.77
95*	1	8.88	46.16	31.80	8.88	46.16	31.80	8.88	46.16	31.80
96*	1	10.24	45.81	30.77	10.24	45.81	30.77	10.24	45.81	30.77
97*	1	8.75	46.41	30.08	8.75	46.41	30.08	8.75	46.41	30.08
98*	1	9.24	48.04	33.05	9.24	48.04	33.05	9.24	48.04	33.05
99*	1	11.04	47.66	32.81	11.04	47.66	32.81	11.04	47.66	32.81
100	1	8.93	51.75	32.75	8.98	51.77	32.77	8.98	51.78	32.81
101	1	8.13	50.17	32.95	8.16	50.23	33.11	8.19	50.26	33.31
102	1	8.73	50.68	31.35	8.66	50.64	31.45	8.55	50.57	31.59
103	6	14.98	51.43	24.41	14.98	51.41	24.40	14.98	51.40	24.40
104	8	15.29	52.84	24.41	15.27	52.82	24.38	15.26	52.81	24.36
105	6	13.82	51.20	23.46	13.81	51.16	23.46	13.82	51.13	23.45
106	1	13.54	50.14	23.44	13.54	50.10	23.46	13.56	50.07	23.46
107	1	12.94	51.77	23.77	12.94	51.74	23.76	12.94	51.71	23.74
108	1	14.10	51.51	22.45	14.09	51.45	22.44	14.10	51.41	22.43
109	1	15.87	50.90	24.04	15.87	50.88	24.04	15.87	50.87	24.04
110	1	16.15	52.90	24.85	16.13	52.91	24.81	16.13	52.92	24.78
111	7	18.70	51.39	29.07	18.68	51.33	28.96	18.66	51.30	28.91
112	6	19.51	50.18	29.28	19.46	50.09	29.08	19.43	50.04	29.00
113	6	19.17	49.42	30.57	19.23	49.35	30.40	19.21	49.30	30.33
114	8	19.23	49.93	31.68	19.46	49.85	31.49	19.45	49.82	31.42
115	6	20.94	50.73	29.28	20.91	50.56	28.92	20.88	50.50	28.82
116	6	20.87	51.85	28.23	20.78	51.71	27.90	20.75	51.66	27.82
117	6	19.48	52.46	28.41	19.46	52.40	28.29	19.45	52.36	28.24
118	7	18.88	48.10	30.36	18.83	48.05	30.24	18.81	48.01	30.19
119	6	18.51	47.21	31.44	18.50	47.19	31.35	18.49	47.16	31.31
120	6	17.01	47.03	31.72	17.01	47.02	31.67	17.00	47.01	31.64
121	8	16.65	46.13	32.47	16.65	46.10	32.40	16.63	46.07	32.35
122	7	16.16	47.93	31.14	16.15	47.95	31.15	16.15	47.95	31.15
123	6	15.86	53.23	30.04	15.87	53.21	30.05	15.86	53.21	30.05
124	1	15.02	53.85	29.71	15.07	53.89	29.73	15.14	53.97	29.73
125	1	15.43	52.49	30.72	15.38	52.48	30.69	15.29	52.48	30.63
126	1	21.67	52.59	28.34	21.63	52.40	27.93	21.60	52.34	27.84
127	1	19.48	53.35	29.05	19.60	53.23	28.98	19.62	53.17	28.95
128	1	21.68	49.96	29.06	21.57	49.76	28.59	21.52	49.69	28.46
129	1	21.14	51.14	30.28	21.26	50.93	29.88	21.26	50.85	29.78
130	1	18.92	46.21	31.27	18.91	46.18	31.20	18.89	46.15	31.15
131	1	18.97	47.60	32.35	18.98	47.61	32.24	18.98	47.57	32.19
132	1	18.69	47.80	29.42	18.57	47.74	29.31	18.56	47.69	29.26
133	1	16.51	48.72	30.62	16.51	48.75	30.63	16.51	48.75	30.62
134	1	19.01	52.71	27.45	18.92	52.75	27.41	18.90	52.75	27.38
135	1	20.97	51.41	27.22	20.71	51.31	26.89	20.65	51.27	26.80
136	6	16.87	54.13	30.76	16.90	54.02	30.84	16.91	53.86	30.94
137	7	16.44	54.61	31.97	16.43	54.57	31.98	16.42	54.53	32.00

138	8	17.97	54.41	30.30	18.07	54.15	30.46	18.12	53.79	30.70
139	1	17.04	55.30	32.39	17.06	55.17	32.50	17.07	54.98	32.63
140	1	15.45	54.58	32.22	15.43	54.59	32.18	15.41	54.61	32.16

F114P – OX

Atom number	Atomic number	ε = 1			ε = 5.7			ε = 78.4		
		X	Y	Z	X	Y	Z	X	Y	Z
1	29	12.23	52.14	31.69	12.23	52.14	31.69	12.23	52.14	31.69
2	16	11.89	52.12	29.55	11.89	52.12	29.55	11.89	52.12	29.55
3	6	11.02	50.66	28.86	11.02	50.66	28.86	11.02	50.66	28.86
4*	6	11.21	50.54	27.34	11.21	50.54	27.34	11.21	50.54	27.34
5	6	12.66	50.19	26.99	12.66	50.19	26.99	12.66	50.19	26.99
6	8	13.18	49.14	27.38	13.18	49.14	27.38	13.18	49.14	27.38
7	7	13.34	51.11	26.25	13.34	51.11	26.25	13.34	51.11	26.25
8*	6	14.73	50.89	25.85	14.73	50.89	25.85	14.73	50.89	25.85
9	6	15.81	51.33	26.85	15.81	51.33	26.85	15.81	51.33	26.85
10	8	16.90	50.75	26.80	16.90	50.75	26.80	16.90	50.75	26.80
11	7	15.54	52.29	27.77	15.54	52.29	27.77	15.54	52.29	27.77
12*	6	16.52	52.53	28.81	16.52	52.53	28.81	16.52	52.53	28.81
13	6	16.96	51.24	29.53	16.96	51.24	29.53	16.96	51.24	29.53
14	8	16.20	50.29	29.73	16.20	50.29	29.73	16.20	50.29	29.73
15	6	15.08	54.42	28.71	15.08	54.42	28.71	15.08	54.42	28.71
16	6	14.56	53.40	27.68	14.56	53.40	27.68	14.56	53.40	27.68
17	7	18.23	51.27	29.97	18.23	51.27	29.97	18.23	51.27	29.97
18	6	18.82	50.14	30.69	18.82	50.14	30.69	18.82	50.14	30.69
19*	6	14.76	47.95	31.60	14.76	47.95	31.60	14.76	47.95	31.60
20	6	13.92	46.90	30.86	13.92	46.90	30.86	13.92	46.90	30.86
21	6	14.03	49.30	31.53	14.03	49.30	31.53	14.03	49.30	31.53
22	8	13.03	46.27	31.46	13.03	46.27	31.46	13.03	46.27	31.46
23	7	14.15	46.75	29.53	14.15	46.75	29.53	14.15	46.75	29.53
24	6	14.52	50.34	32.48	14.52	50.34	32.48	14.52	50.34	32.48
25*	6	13.32	45.88	28.69	13.32	45.88	28.69	13.32	45.88	28.69
26	6	15.60	50.35	33.32	15.60	50.35	33.32	15.60	50.35	33.32
27	7	13.87	51.58	32.60	13.87	51.58	32.60	13.87	51.58	32.60
28	7	15.62	51.60	33.92	15.62	51.60	33.92	15.62	51.60	33.92
29	6	14.57	52.31	33.46	14.57	52.31	33.46	14.57	52.31	33.46
30	7	11.28	53.17	33.07	11.28	53.17	33.07	11.28	53.17	33.07
31	6	10.42	54.25	32.89	10.42	54.25	32.89	10.42	54.25	32.89
32	6	11.41	52.99	34.38	11.41	52.99	34.38	11.41	52.99	34.38
33	6	9.98	54.78	31.56	9.98	54.78	31.56	9.98	54.78	31.56
34	6	10.05	54.71	34.13	10.05	54.71	34.13	10.05	54.71	34.13
35	7	10.68	53.90	35.05	10.68	53.90	35.05	10.68	53.90	35.05
36	6	11.14	55.31	30.67	11.14	55.31	30.67	11.14	55.31	30.67
37	7	11.97	56.29	31.35	11.97	56.29	31.35	11.97	56.29	31.35
38	6	10.57	55.94	29.39	10.57	55.94	29.39	10.57	55.94	29.39
39	6	13.16	55.95	31.90	13.16	55.95	31.90	13.16	55.95	31.90

40	8	10.22	57.12	29.38	10.22	57.12	29.38	10.22	57.12	29.38
41*	6	13.96	57.07	32.51	13.96	57.07	32.51	13.96	57.07	32.51
42	8	13.57	54.78	31.91	13.57	54.78	31.91	13.57	54.78	31.91
43	7	8.14	54.49	25.57	8.14	54.49	25.57	8.14	54.49	25.57
44	6	7.13	53.54	25.12	7.13	53.54	25.12	7.13	53.54	25.12
45*	6	9.90	47.88	31.09	9.90	47.88	31.09	9.90	47.88	31.09
46*	6	9.41	46.46	30.92	9.41	46.46	30.92	9.41	46.46	30.92
47*	6	10.16	48.22	32.54	10.16	48.22	32.54	10.16	48.22	32.54
48	16	10.54	49.99	32.88	10.54	49.99	32.88	10.54	49.99	32.88
49	6	8.88	50.71	32.63	8.88	50.71	32.63	8.88	50.71	32.63
50	7	10.46	55.10	28.34	10.46	55.10	28.34	10.46	55.10	28.34
51*	6	9.79	55.45	27.11	9.79	55.45	27.11	9.79	55.45	27.11
52	6	8.80	54.32	26.73	8.80	54.32	26.73	8.80	54.32	26.73
53	6	10.79	55.82	25.99	10.79	55.82	25.99	10.79	55.82	25.99
54	6	11.70	54.67	25.56	11.70	54.67	25.56	11.70	54.67	25.56
55	8	8.62	53.35	27.47	8.62	53.35	27.47	8.62	53.35	27.47
56	7	12.66	54.98	24.66	12.66	54.98	24.66	12.66	54.98	24.66
57	8	11.56	53.53	26.01	11.56	53.53	26.01	11.56	53.53	26.01
58	6	15.82	53.55	29.72	15.82	53.55	29.72	15.82	53.55	29.72
59	1	13.34	54.26	24.40	13.34	54.26	24.40	13.34	54.26	24.40
60	1	12.80	55.93	24.34	12.80	55.93	24.34	12.80	55.93	24.34
61	1	14.26	54.98	29.16	14.26	54.98	29.16	14.26	54.98	29.16
62	1	15.76	55.13	28.24	15.76	55.13	28.24	15.76	55.13	28.24
63	1	13.57	53.06	27.96	13.57	53.06	27.96	13.57	53.06	27.96
64	1	14.53	53.81	26.67	14.53	53.81	26.67	14.53	53.81	26.67
65	1	11.40	49.76	29.35	11.40	49.76	29.35	11.40	49.76	29.35
66	1	9.96	50.76	29.09	9.96	50.76	29.09	9.96	50.76	29.09
67*	1	10.85	51.40	26.82	10.85	51.40	26.82	10.85	51.40	26.82
68*	1	10.60	49.70	27.10	10.60	49.70	27.10	10.60	49.70	27.10
69	1	12.87	51.98	26.00	12.87	51.98	26.00	12.87	51.98	26.00
70*	1	14.85	49.83	25.80	14.85	49.83	25.80	14.85	49.83	25.80
71	1	18.83	52.03	29.71	18.83	52.03	29.71	18.83	52.03	29.71
72	1	18.93	49.27	30.04	18.93	49.27	30.04	18.93	49.27	30.04
73	1	19.81	50.44	31.05	19.81	50.44	31.05	19.81	50.44	31.05
74*	1	14.78	47.62	32.62	14.78	47.62	32.62	14.78	47.62	32.62
75	1	12.96	49.12	31.73	12.96	49.12	31.73	12.96	49.12	31.73
76	1	14.10	49.71	30.51	14.10	49.71	30.51	14.10	49.71	30.51
77	1	14.81	47.37	29.09	14.81	47.37	29.09	14.81	47.37	29.09
78*	1	13.80	44.94	28.50	13.80	44.94	28.50	13.80	44.94	28.50
79*	1	12.41	45.72	29.23	12.41	45.72	29.23	12.41	45.72	29.23
80*	1	13.11	46.37	27.76	13.11	46.37	27.76	13.11	46.37	27.76
81	1	16.35	49.61	33.53	16.35	49.61	33.53	16.35	49.61	33.53
82	1	16.31	51.93	34.58	16.31	51.93	34.58	16.31	51.93	34.58
83	1	14.37	53.33	33.72	14.37	53.33	33.72	14.37	53.33	33.72
84	1	12.00	52.22	34.86	12.00	52.22	34.86	12.00	52.22	34.86
85	1	9.26	55.58	31.73	9.26	55.58	31.73	9.26	55.58	31.73
86	1	9.46	53.99	31.00	9.46	53.99	31.00	9.46	53.99	31.00
87	1	9.40	55.52	34.43	9.40	55.52	34.43	9.40	55.52	34.43
88	1	10.60	53.96	36.06	10.60	53.96	36.06	10.60	53.96	36.06

89	1	11.78	54.47	30.43	11.78	54.47	30.43	11.78	54.47	30.43
90	1	11.68	57.25	31.28	11.68	57.25	31.28	11.68	57.25	31.28
91	1	13.52	58.06	32.33	13.52	58.06	32.33	13.52	58.06	32.33
92	1	14.97	57.05	32.10	14.97	57.05	32.10	14.97	57.05	32.10
93	1	14.04	56.90	33.59	14.04	56.90	33.59	14.04	56.90	33.59
94	1	8.24	55.36	25.05	8.24	55.36	25.05	8.24	55.36	25.05
95	1	7.50	52.53	25.24	7.50	52.53	25.24	7.50	52.53	25.24
96	1	6.20	53.64	25.70	6.20	53.64	25.70	6.20	53.64	25.70
97	1	6.91	53.73	24.07	6.91	53.73	24.07	6.91	53.73	24.07
98*	1	10.85	47.93	30.60	10.85	47.93	30.60	10.85	47.93	30.60
99*	1	9.10	48.52	30.77	9.10	48.52	30.77	9.10	48.52	30.77
100*	1	8.88	46.16	31.80	8.88	46.16	31.80	8.88	46.16	31.80
101*	1	10.24	45.81	30.77	10.24	45.81	30.77	10.24	45.81	30.77
102*	1	8.75	46.41	30.08	8.75	46.41	30.08	8.75	46.41	30.08
103*	1	9.24	48.04	33.05	9.24	48.04	33.05	9.24	48.04	33.05
104*	1	11.04	47.66	32.81	11.04	47.66	32.81	11.04	47.66	32.81
105	1	8.95	51.77	32.86	8.95	51.77	32.86	8.95	51.77	32.86
106	1	8.16	50.24	33.30	8.16	50.24	33.30	8.16	50.24	33.30
107	1	8.55	50.60	31.59	8.55	50.60	31.59	8.55	50.60	31.59
108	1	18.20	49.86	31.54	18.20	49.86	31.54	18.20	49.86	31.54
109*	1	15.76	47.98	31.21	15.76	47.98	31.21	15.76	47.98	31.21
110	1	16.54	54.10	30.34	16.54	54.10	30.34	16.54	54.10	30.34
111	1	15.12	53.04	30.38	15.12	53.04	30.38	15.12	53.04	30.38
112	1	10.27	56.21	25.11	10.27	56.21	25.11	10.27	56.21	25.11
113	1	11.42	56.65	26.35	11.42	56.65	26.35	11.42	56.65	26.35
114	1	9.21	56.35	27.32	9.21	56.35	27.32	9.21	56.35	27.32
115	1	10.76	54.13	28.45	10.76	54.13	28.45	10.76	54.13	28.45
116	1	17.42	52.99	28.38	17.42	52.99	28.38	17.42	52.99	28.38
117	6	15.01	51.44	24.43	15.01	51.44	24.43	15.01	51.44	24.43
118	8	14.84	52.87	24.38	14.84	52.87	24.38	14.84	52.87	24.38
119	6	14.11	50.83	23.37	14.11	50.83	23.37	14.11	50.83	23.37
120	1	16.06	51.20	24.21	16.06	51.20	24.21	16.06	51.20	24.21
121	1	14.21	49.74	23.37	14.21	49.74	23.37	14.21	49.74	23.37
122	1	13.06	51.08	23.56	13.06	51.08	23.56	13.06	51.08	23.56
123	1	14.39	51.21	22.39	14.39	51.21	22.39	14.39	51.21	22.39
124	1	15.67	53.30	24.62	15.67	53.30	24.62	15.67	53.30	24.62

F114P – RED

Atom number	Atomic number	ε = 1			ε = 5.7			ε = 78.4		
		X	Y	Z	X	Y	Z	X	Y	Z
1	29	12.16	52.20	31.71	12.15	52.26	31.73	12.09	52.25	31.78
2	16	11.39	52.24	29.61	11.45	52.25	29.60	11.52	52.22	29.59
3	6	10.99	50.63	28.87	11.01	50.63	28.87	10.99	50.64	28.86
4*	6	11.21	50.54	27.34	11.22	50.54	27.34	11.22	50.54	27.34
5	6	12.66	50.19	27.00	12.66	50.19	26.99	12.67	50.21	27.01
6	8	13.20	49.16	27.40	13.19	49.14	27.37	13.22	49.19	27.45
7	7	13.33	51.10	26.22	13.33	51.10	26.23	13.33	51.11	26.23
8*	6	14.73	50.89	25.86	14.73	50.89	25.86	14.73	50.89	25.86
9	6	15.79	51.34	26.86	15.80	51.33	26.86	15.80	51.33	26.86
10	8	16.93	50.86	26.75	16.92	50.81	26.76	16.91	50.79	26.78
11	7	15.49	52.23	27.83	15.51	52.25	27.80	15.52	52.26	27.79
12*	6	16.52	52.53	28.81	16.52	52.53	28.81	16.52	52.53	28.81
13	6	17.12	51.26	29.43	17.07	51.26	29.48	16.98	51.25	29.53
14	8	16.44	50.43	30.02	16.37	50.31	29.80	16.25	50.28	29.71
15	6	14.85	54.23	28.94	14.89	54.27	28.87	15.00	54.37	28.75
16	6	14.35	53.17	27.94	14.39	53.22	27.88	14.45	53.30	27.80
17	7	18.49	51.18	29.37	18.40	51.32	29.78	18.25	51.31	30.01
18	6	19.16	49.97	29.79	19.10	50.21	30.40	18.85	50.21	30.75
19*	6	14.76	47.95	31.60	14.76	47.95	31.60	14.76	47.95	31.60
20	6	13.94	46.88	30.86	13.96	46.86	30.86	13.96	46.86	30.86
21	6	14.03	49.30	31.49	14.03	49.30	31.48	14.01	49.29	31.50
22	8	13.11	46.19	31.46	13.19	46.12	31.47	13.16	46.14	31.47
23	7	14.16	46.76	29.52	14.15	46.77	29.52	14.15	46.77	29.52
24	6	14.49	50.34	32.45	14.48	50.34	32.45	14.47	50.34	32.47
25*	6	13.32	45.88	28.69	13.32	45.88	28.69	13.32	45.88	28.69
26	6	15.56	50.34	33.31	15.53	50.33	33.33	15.52	50.33	33.35
27	7	13.85	51.58	32.53	13.84	51.58	32.52	13.82	51.57	32.54
28	7	15.57	51.58	33.91	15.54	51.57	33.94	15.51	51.57	33.95
29	6	14.53	52.30	33.41	14.51	52.29	33.43	14.48	52.28	33.44
30	7	11.14	53.28	33.05	11.14	53.33	33.08	11.13	53.36	33.11
31	6	10.28	54.36	32.88	10.28	54.41	32.92	10.29	54.45	32.95
32	6	11.21	53.07	34.36	11.25	53.14	34.39	11.26	53.18	34.42
33	6	9.91	54.88	31.52	9.89	54.93	31.57	9.87	54.98	31.61
34	6	9.84	54.79	34.10	9.88	54.86	34.15	9.93	54.93	34.18
35	7	10.44	53.97	35.04	10.50	54.04	35.07	10.55	54.11	35.10
36	6	11.14	55.29	30.68	11.10	55.32	30.69	11.08	55.35	30.71
37	7	11.98	56.28	31.34	11.97	56.30	31.34	11.97	56.31	31.34
38	6	10.71	55.89	29.32	10.63	55.92	29.35	10.59	55.93	29.37
39	6	13.13	55.93	31.95	13.14	55.95	31.92	13.16	55.96	31.87
40	8	10.69	57.12	29.17	10.50	57.14	29.24	10.38	57.15	29.27
41*	6	13.96	57.07	32.51	13.96	57.07	32.51	13.96	57.07	32.51
42	8	13.52	54.77	32.09	13.54	54.78	32.00	13.59	54.80	31.87
43	7	8.31	54.63	25.33	8.26	54.64	25.38	8.24	54.61	25.41
44	6	7.42	53.69	24.66	7.26	53.78	24.77	7.21	53.73	24.86
45*	6	9.90	47.88	31.09	9.90	47.88	31.09	9.90	47.88	31.09

46*	6	9.41	46.46	30.92	9.41	46.46	30.92	9.41	46.46	30.92
47*	6	10.16	48.22	32.54	10.16	48.22	32.54	10.16	48.22	32.54
48	16	10.56	49.99	32.87	10.56	49.99	32.87	10.55	49.99	32.87
49	6	8.99	50.76	32.36	8.92	50.72	32.57	8.89	50.71	32.64
50	7	10.33	55.01	28.38	10.35	55.03	28.38	10.37	55.05	28.38
51*	6	9.79	55.45	27.11	9.79	55.45	27.11	9.79	55.45	27.11
52	6	8.83	54.37	26.57	8.80	54.38	26.60	8.78	54.39	26.63
53	6	10.91	55.88	26.13	10.89	55.86	26.09	10.87	55.85	26.07
54	6	11.80	54.74	25.62	11.78	54.72	25.60	11.74	54.70	25.56
55	8	8.49	53.41	27.24	8.49	53.41	27.28	8.46	53.43	27.33
56	7	12.94	55.13	25.00	12.89	55.10	24.93	12.83	55.07	24.86
57	8	11.49	53.56	25.74	11.49	53.53	25.78	11.46	53.52	25.76
58	6	15.80	53.42	29.83	15.79	53.45	29.80	15.84	53.55	29.74
59	1	13.59	54.41	24.68	13.53	54.38	24.60	13.48	54.35	24.54
60	1	13.24	56.09	24.99	13.14	56.07	24.83	13.09	56.04	24.75
61	1	14.02	54.66	29.50	14.06	54.74	29.40	14.19	54.91	29.24
62	1	15.39	55.04	28.43	15.47	55.05	28.36	15.63	55.08	28.21
63	1	13.46	52.68	28.34	13.49	52.74	28.28	13.52	52.88	28.19
64	1	14.11	53.60	26.96	14.17	53.63	26.89	14.29	53.67	26.79
65	1	11.56	49.83	29.34	11.58	49.83	29.34	11.51	49.81	29.34
66	1	9.93	50.42	29.05	9.95	50.44	29.07	9.92	50.50	29.06
67*	1	10.85	51.40	26.82	10.85	51.40	26.82	10.85	51.40	26.82
68*	1	10.60	49.70	27.10	10.60	49.70	27.10	10.60	49.70	27.10
69	1	12.85	51.96	25.96	12.86	51.96	25.98	12.85	51.95	25.95
70*	1	14.85	49.83	25.80	14.85	49.83	25.80	14.85	49.83	25.80
71	1	18.90	51.66	28.58	18.96	52.04	29.33	18.82	52.11	29.78
72	1	19.03	49.15	29.07	19.31	49.41	29.68	18.96	49.32	30.12
73	1	20.23	50.17	29.91	20.04	50.56	30.82	19.83	50.52	31.10
74*	1	14.78	47.62	32.62	14.78	47.62	32.62	14.78	47.62	32.62
75	1	12.96	49.13	31.66	12.95	49.13	31.63	12.95	49.11	31.67
76	1	14.13	49.70	30.48	14.14	49.71	30.47	14.11	49.69	30.49
77	1	14.66	47.50	29.05	14.64	47.52	29.05	14.69	47.48	29.06
78*	1	13.80	44.94	28.50	13.80	44.94	28.50	13.80	44.94	28.50
79*	1	12.41	45.72	29.23	12.41	45.72	29.23	12.41	45.72	29.23
80*	1	13.11	46.37	27.76	13.11	46.37	27.76	13.11	46.37	27.76
81	1	16.31	49.59	33.53	16.26	49.57	33.57	16.26	49.58	33.59
82	1	16.25	51.92	34.58	16.19	51.89	34.63	16.17	51.90	34.65
83	1	14.32	53.33	33.65	14.30	53.31	33.68	14.27	53.31	33.70
84	1	11.78	52.29	34.84	11.83	52.37	34.87	11.84	52.41	34.90
85	1	9.23	55.73	31.64	9.22	55.79	31.69	9.22	55.85	31.74
86	1	9.39	54.10	30.96	9.34	54.15	31.02	9.29	54.21	31.07
87	1	9.18	55.59	34.38	9.22	55.66	34.44	9.30	55.75	34.48
88	1	10.31	54.00	36.03	10.40	54.09	36.08	10.49	54.18	36.11
89	1	11.74	54.38	30.57	11.69	54.41	30.58	11.65	54.42	30.59
90	1	11.79	57.24	31.07	11.74	57.27	31.18	11.70	57.28	31.27
91	1	13.57	58.06	32.27	13.56	58.06	32.28	13.56	58.07	32.30
92	1	14.98	56.99	32.12	14.98	57.00	32.13	14.99	57.02	32.15
93	1	14.01	56.97	33.60	14.00	56.95	33.59	13.98	56.92	33.59
94	1	8.69	55.39	24.79	8.50	55.50	24.90	8.46	55.46	24.90

95	1	7.04	53.01	25.42	7.28	52.83	25.29	7.54	52.69	24.92
96	1	6.59	54.22	24.19	6.26	54.21	24.84	6.27	53.83	25.41
97	1	7.95	53.11	23.89	7.50	53.62	23.71	7.05	54.00	23.82
98*	1	10.85	47.93	30.60	10.85	47.93	30.60	10.85	47.93	30.60
99*	1	9.10	48.52	30.77	9.10	48.52	30.77	9.10	48.52	30.77
100*	1	8.88	46.16	31.80	8.88	46.16	31.80	8.88	46.16	31.80
101*	1	10.24	45.81	30.77	10.24	45.81	30.77	10.24	45.81	30.77
102*	1	8.75	46.41	30.08	8.75	46.41	30.08	8.75	46.41	30.08
103*	1	9.24	48.04	33.05	9.24	48.04	33.05	9.24	48.04	33.05
104*	1	11.04	47.66	32.81	11.04	47.66	32.81	11.04	47.66	32.81
105	1	8.96	51.75	32.79	8.99	51.78	32.81	8.96	51.77	32.87
106	1	8.14	50.18	32.74	8.17	50.26	33.22	8.17	50.24	33.31
107	1	8.93	50.86	31.27	8.62	50.62	31.53	8.55	50.60	31.60
108	1	18.74	49.65	30.75	18.47	49.80	31.19	18.23	49.94	31.61
109*	1	15.76	47.98	31.21	15.76	47.98	31.21	15.76	47.98	31.21
110	1	16.50	54.04	30.40	16.49	54.06	30.39	16.57	54.15	30.29
111	1	15.22	52.80	30.52	15.18	52.86	30.48	15.19	53.04	30.45
112	1	10.49	56.40	25.25	10.44	56.36	25.22	10.41	56.34	25.20
113	1	11.52	56.63	26.64	11.51	56.63	26.57	11.50	56.61	26.53
114	1	9.20	56.36	27.30	9.21	56.36	27.30	9.22	56.37	27.31
115	1	10.43	54.00	28.57	10.53	54.03	28.56	10.58	54.05	28.55
116	1	17.34	53.09	28.33	17.36	53.07	28.35	17.40	52.99	28.34
117	6	15.06	51.50	24.48	15.05	51.48	24.46	15.03	51.46	24.45
118	8	15.04	52.95	24.53	14.97	52.93	24.48	14.91	52.90	24.43
119	6	14.11	51.08	23.37	14.11	51.00	23.37	14.12	50.91	23.37
120	1	16.08	51.18	24.24	16.08	51.19	24.23	16.07	51.19	24.23
121	1	14.10	49.99	23.27	14.13	49.91	23.30	14.17	49.82	23.34
122	1	13.09	51.42	23.58	13.08	51.31	23.56	13.08	51.20	23.55
123	1	14.43	51.51	22.42	14.42	51.41	22.40	14.42	51.31	22.39
124	1	15.78	53.23	25.08	15.75	53.27	24.94	15.71	53.29	24.80

M121G – OX

Atom number	Atomic number	ε = 1			ε = 5.7			ε = 78.4		
		X	Y	Z	X	Y	Z	X	Y	Z
1	29	12.74	52.44	31.40	12.66	52.44	31.42	12.69	52.47	31.41
2	16	12.28	52.20	29.30	12.26	52.22	29.30	12.27	52.24	29.28
3	6	11.17	50.78	28.86	11.19	50.77	28.86	11.20	50.78	28.86
4*	6	11.21	50.54	27.34	11.21	50.54	27.34	11.21	50.54	27.34
5	6	12.64	50.15	26.94	12.63	50.14	26.90	12.62	50.15	26.89
6	8	13.10	49.03	27.19	13.07	49.01	27.11	13.06	49.01	27.09
7	7	13.38	51.13	26.35	13.37	51.13	26.32	13.37	51.13	26.32
8*	6	14.73	50.89	25.85	14.73	50.89	25.85	14.73	50.89	25.85
9	6	15.83	51.53	26.70	15.83	51.49	26.75	15.83	51.49	26.75
10	8	16.97	51.67	26.22	17.00	51.52	26.35	17.00	51.50	26.35
11	7	15.50	51.95	27.93	15.46	52.04	27.93	15.46	52.04	27.93
12*	6	16.49	52.55	28.82	16.48	52.55	28.81	16.49	52.55	28.82

13	6	17.45	51.51	29.46	17.37	51.40	29.35	17.38	51.41	29.34
14	8	17.40	51.28	30.66	16.88	50.31	29.67	16.95	50.26	29.51
15	7	18.34	50.95	28.61	18.67	51.72	29.52	18.64	51.79	29.67
16	6	19.31	49.95	29.04	19.65	50.77	30.04	19.62	50.88	30.25
17*	6	14.76	47.95	31.60	14.76	47.95	31.60	14.76	47.95	31.60
18	6	13.88	46.93	30.86	13.93	46.89	30.86	13.93	46.89	30.86
19	6	14.07	49.33	31.53	14.08	49.33	31.49	14.08	49.33	31.49
20	8	12.95	46.37	31.44	13.10	46.21	31.47	13.08	46.22	31.47
21	7	14.15	46.74	29.54	14.14	46.77	29.53	14.14	46.76	29.53
22	6	14.72	50.39	32.37	14.66	50.38	32.39	14.68	50.39	32.38
23*	6	13.32	45.88	28.69	13.32	45.88	28.69	13.32	45.88	28.69
24	6	15.86	50.33	33.12	15.73	50.31	33.24	15.75	50.32	33.22
25	7	14.24	51.70	32.41	14.14	51.67	32.46	14.17	51.69	32.43
26	7	16.06	51.61	33.62	15.86	51.56	33.82	15.89	51.58	33.78
27	6	15.08	52.40	33.16	14.89	52.36	33.33	14.93	52.37	33.28
28	7	11.40	52.96	32.74	11.36	52.99	32.77	11.40	53.00	32.78
29	6	10.38	53.91	32.62	10.36	53.96	32.67	10.39	53.96	32.70
30	6	11.36	52.51	33.99	11.33	52.55	34.03	11.41	52.55	34.04
31	6	10.04	54.61	31.34	10.00	54.66	31.39	9.99	54.67	31.44
32	6	9.74	54.01	33.82	9.76	54.08	33.89	9.82	54.07	33.94
33	7	10.37	53.12	34.67	10.38	53.18	34.73	10.47	53.17	34.76
34	6	11.23	55.25	30.59	11.19	55.28	30.62	11.16	55.29	30.65
35	7	11.98	56.22	31.38	11.96	56.23	31.40	11.95	56.24	31.41
36	6	10.74	55.95	29.32	10.71	55.97	29.34	10.65	55.98	29.38
37	6	13.18	55.91	31.91	13.17	55.92	31.91	13.17	55.92	31.90
38	8	10.35	57.12	29.36	10.35	57.16	29.36	10.23	57.14	29.43
39	7	10.76	55.17	28.21	10.70	55.18	28.25	10.71	55.23	28.27
40*	6	13.96	57.07	32.51	13.96	57.07	32.51	13.96	57.07	32.51
41	8	13.63	54.75	31.91	13.61	54.75	31.89	13.63	54.76	31.84
42	6	10.07	55.51	26.99	10.13	55.58	26.98	10.18	55.65	26.99
43	6	9.07	54.36	26.68	9.13	54.48	26.52	9.15	54.60	26.49
44	6	11.06	55.84	25.85	11.23	55.92	25.94	11.31	55.98	25.99
45	8	8.96	53.40	27.44	8.91	53.49	27.21	8.83	53.64	27.17
46	7	8.33	54.53	25.56	8.52	54.72	25.34	8.62	54.85	25.27
47	6	12.02	54.69	25.53	12.17	54.75	25.65	12.21	54.78	25.67
48	6	7.31	53.58	25.14	7.51	53.82	24.80	7.59	53.99	24.70
49	7	13.10	54.99	24.78	13.34	55.05	25.06	13.38	55.06	25.07
50	8	11.80	53.55	25.95	11.84	53.59	25.93	11.86	53.62	25.96
51	1	11.49	49.88	29.40	11.55	49.88	29.39	11.57	49.90	29.39
52	1	10.16	51.05	29.17	10.18	51.00	29.20	10.19	51.00	29.21
53*	1	10.85	51.40	26.82	10.85	51.40	26.82	10.85	51.40	26.82
54*	1	10.60	49.70	27.10	10.60	49.70	27.10	10.60	49.70	27.10
55	1	12.94	52.03	26.15	12.94	52.04	26.15	12.94	52.05	26.16
56*	1	14.85	49.83	25.80	14.85	49.83	25.80	14.85	49.83	25.80
57	1	14.54	51.84	28.26	14.50	51.95	28.26	14.50	51.97	28.25
58*	1	17.09	53.28	28.31	17.09	53.28	28.31	17.09	53.28	28.31
59*	1	16.04	52.98	29.69	16.04	52.98	29.69	16.04	52.98	29.69
60	1	18.25	51.17	27.62	19.01	52.58	29.13	18.94	52.72	29.44
61	1	19.06	48.96	28.66	20.29	50.38	29.25	20.34	50.53	29.50

62	1	20.31	50.21	28.67	20.28	51.25	30.80	20.17	51.38	31.05
63*	1	14.78	47.62	32.62	14.78	47.62	32.62	14.78	47.62	32.62
64	1	13.03	49.19	31.85	13.01	49.22	31.71	13.02	49.22	31.74
65	1	14.03	49.67	30.49	14.16	49.69	30.45	14.13	49.68	30.45
66	1	14.86	47.30	29.09	14.78	47.40	29.07	14.81	47.37	29.08
67*	1	13.80	44.94	28.50	13.80	44.94	28.50	13.80	44.94	28.50
68*	1	12.41	45.72	29.23	12.41	45.72	29.23	12.41	45.72	29.23
69*	1	13.11	46.37	27.76	13.11	46.37	27.76	13.11	46.37	27.76
70	1	16.54	49.53	33.32	16.41	49.51	33.48	16.42	49.51	33.47
71	1	16.86	51.91	34.15	16.56	51.85	34.48	16.60	51.86	34.44
72	1	15.00	53.47	33.35	14.76	53.40	33.57	14.81	53.42	33.52
73	1	12.02	51.77	34.41	11.98	51.79	34.44	12.08	51.80	34.43
74	1	9.29	55.39	31.55	9.27	55.44	31.62	9.27	55.45	31.70
75	1	9.57	53.91	30.64	9.50	53.96	30.71	9.48	53.97	30.77
76	1	8.91	54.62	34.14	8.95	54.72	34.22	9.01	54.69	34.30
77	1	10.12	52.94	35.64	10.16	53.02	35.70	10.28	53.01	35.74
78	1	11.93	54.46	30.33	11.87	54.47	30.37	11.84	54.48	30.38
79	1	11.68	57.18	31.28	11.67	57.20	31.34	11.64	57.20	31.41
80	1	11.04	54.20	28.31	10.98	54.21	28.34	11.05	54.27	28.33
81	1	13.41	58.01	32.48	13.35	57.97	32.63	13.40	58.01	32.51
82	1	14.89	57.19	31.95	14.80	57.30	31.85	14.88	57.20	31.94
83	1	14.22	56.84	33.54	14.37	56.77	33.47	14.23	56.81	33.53
84	1	9.49	56.41	27.19	9.56	56.50	27.16	9.63	56.58	27.17
85	1	11.63	56.73	26.13	11.81	56.77	26.32	11.92	56.79	26.40
86	1	10.52	56.10	24.93	10.78	56.25	25.00	10.90	56.36	25.05
87	1	8.43	55.38	25.03	8.65	55.61	24.88	8.83	55.72	24.80
88	1	7.31	52.75	25.85	7.73	52.81	25.14	7.88	52.95	24.81
89	1	6.31	54.04	25.14	6.50	54.09	25.13	6.62	54.14	25.20
90	1	7.52	53.19	24.14	7.55	53.86	23.70	7.48	54.23	23.64
91	1	13.76	54.25	24.55	13.98	54.30	24.80	14.00	54.29	24.81
92	1	13.26	55.93	24.45	13.58	56.00	24.83	13.64	56.01	24.85
93	1	19.32	49.94	30.13	19.11	49.94	30.50	19.09	50.02	30.66
94*	1	15.76	47.98	31.20	15.76	47.98	31.20	15.76	47.98	31.20
95	6	14.92	51.40	24.39	14.97	51.42	24.41	14.98	51.41	24.41
96	8	15.06	52.83	24.35	15.19	52.84	24.39	15.19	52.83	24.38
97	6	13.78	51.01	23.46	13.83	51.11	23.45	13.84	51.09	23.45
98	1	13.63	49.93	23.47	13.62	50.04	23.44	13.64	50.02	23.44
99	1	12.84	51.49	23.77	12.92	51.64	23.75	12.93	51.62	23.73
100	1	14.01	51.33	22.44	14.10	51.43	22.44	14.12	51.40	22.44
101	1	15.85	50.96	24.03	15.89	50.93	24.06	15.90	50.92	24.07
102	1	15.94	53.02	24.71	16.06	52.99	24.79	16.06	52.99	24.79

Atom number	Atomic number	ε = 1			ε = 5.7			ε = 78.4		
		X	Y	Z	X	Y	Z	X	Y	Z
1	29	12.61	52.45	31.52	12.57	52.47	31.54	12.58	52.51	31.55
2	16	12.44	52.12	29.30	12.40	52.14	29.32	12.39	52.15	29.33
3	6	11.20	50.82	28.86	11.20	50.80	28.86	11.20	50.80	28.86
4*	6	11.21	50.54	27.34	11.21	50.54	27.34	11.21	50.54	27.34
5	6	12.64	50.14	26.93	12.63	50.14	26.90	12.63	50.15	26.90
6	8	13.11	49.04	27.21	13.08	49.01	27.13	13.07	49.01	27.13
7	7	13.37	51.11	26.30	13.36	51.11	26.30	13.36	51.12	26.30
8*	6	14.73	50.89	25.85	14.73	50.89	25.85	14.73	50.89	25.85
9	6	15.82	51.50	26.73	15.81	51.50	26.76	15.82	51.49	26.76
10	8	16.97	51.62	26.26	16.98	51.56	26.34	16.99	51.54	26.35
11	7	15.49	51.93	27.96	15.45	52.02	27.95	15.45	52.03	27.94
12*	6	16.49	52.55	28.82	16.49	52.55	28.82	16.49	52.55	28.82
13	6	17.54	51.60	29.47	17.41	51.42	29.34	17.41	51.42	29.33
14	8	17.82	51.74	30.66	17.01	50.27	29.50	17.05	50.25	29.40
15	7	18.13	50.70	28.65	18.66	51.84	29.66	18.62	51.84	29.76
16	6	19.19	49.83	29.13	19.67	50.94	30.19	19.58	50.93	30.36
17*	6	14.76	47.95	31.60	14.76	47.95	31.60	14.76	47.95	31.60
18	6	14.03	46.81	30.86	13.98	46.85	30.86	13.95	46.87	30.86
19	6	14.06	49.31	31.49	14.08	49.33	31.48	14.08	49.33	31.50
20	8	13.42	45.94	31.47	13.26	46.05	31.48	13.15	46.15	31.47
21	7	14.14	46.81	29.49	14.14	46.79	29.51	14.14	46.77	29.52
22	6	14.80	50.40	32.20	14.74	50.38	32.32	14.74	50.39	32.33
23*	6	13.32	45.88	28.69	13.32	45.88	28.69	13.32	45.88	28.69
24	6	16.02	50.35	32.82	15.85	50.29	33.11	15.84	50.30	33.14
25	7	14.31	51.70	32.28	14.24	51.68	32.38	14.26	51.70	32.37
26	7	16.28	51.62	33.28	16.04	51.55	33.65	16.04	51.56	33.67
27	6	15.24	52.41	32.92	15.05	52.35	33.19	15.07	52.37	33.18
28	7	11.24	52.91	32.84	11.24	52.94	32.89	11.27	52.97	32.92
29	6	10.26	53.89	32.77	10.26	53.93	32.85	10.29	53.96	32.90
30	6	11.21	52.45	34.08	11.24	52.47	34.14	11.31	52.53	34.17
31	6	9.94	54.61	31.49	9.91	54.65	31.59	9.90	54.68	31.64
32	6	9.65	54.01	33.99	9.69	54.05	34.09	9.76	54.10	34.16
33	7	10.26	53.08	34.81	10.32	53.12	34.89	10.41	53.18	34.95
34	6	11.20	55.14	30.76	11.15	55.16	30.81	11.11	55.18	30.83
35	7	11.94	56.14	31.50	11.92	56.15	31.54	11.91	56.16	31.55
36	6	10.80	55.76	29.42	10.71	55.79	29.48	10.65	55.82	29.52
37	6	13.17	55.87	32.01	13.17	55.88	32.00	13.17	55.89	31.98
38	8	10.49	56.95	29.35	10.34	56.97	29.45	10.21	56.97	29.51
39	7	10.79	54.91	28.37	10.75	54.97	28.42	10.75	55.03	28.43
40*	6	13.96	57.07	32.51	13.96	57.07	32.51	13.96	57.07	32.51
41	8	13.63	54.73	32.07	13.64	54.73	32.00	13.64	54.74	31.93
42	6	10.24	55.31	27.09	10.24	55.39	27.12	10.26	55.47	27.13
43	6	9.30	54.19	26.59	9.28	54.31	26.57	9.27	54.43	26.56
44	6	11.34	55.76	26.11	11.38	55.82	26.17	11.41	55.87	26.19
45	8	8.98	53.25	27.30	8.95	53.33	27.23	8.91	53.44	27.20

46	7	8.75	54.40	25.35	8.74	54.59	25.35	8.75	54.73	25.34
47	6	12.25	54.63	25.64	12.28	54.67	25.72	12.30	54.71	25.76
48	6	7.94	53.38	24.71	7.86	53.65	24.68	7.77	53.87	24.70
49	7	13.52	54.95	25.33	13.55	54.98	25.42	13.57	55.01	25.42
50	8	11.81	53.48	25.53	11.84	53.52	25.62	11.86	53.55	25.72
51	1	11.43	49.90	29.40	11.47	49.89	29.39	11.48	49.88	29.39
52	1	10.20	51.15	29.15	10.20	51.10	29.17	10.19	51.08	29.18
53*	1	10.85	51.40	26.82	10.85	51.40	26.82	10.85	51.40	26.82
54*	1	10.60	49.70	27.10	10.60	49.70	27.10	10.60	49.70	27.10
55	1	12.91	52.00	26.10	12.92	52.01	26.12	12.93	52.02	26.13
56*	1	14.85	49.83	25.80	14.85	49.83	25.80	14.85	49.83	25.80
57	1	14.52	51.84	28.32	14.49	51.92	28.31	14.49	51.94	28.30
58*	1	17.09	53.28	28.31	17.09	53.28	28.31	17.09	53.28	28.31
59*	1	16.04	52.98	29.69	16.04	52.98	29.69	16.04	52.98	29.69
60	1	17.97	50.80	27.65	18.95	52.77	29.39	18.88	52.81	29.65
61	1	19.15	48.87	28.61	20.39	50.63	29.42	19.97	50.21	29.63
62	1	20.19	50.27	28.98	20.22	51.42	31.00	20.42	51.51	30.75
63*	1	14.78	47.62	32.62	14.78	47.62	32.62	14.78	47.62	32.62
64	1	13.04	49.22	31.89	13.03	49.24	31.77	13.03	49.23	31.80
65	1	13.93	49.61	30.44	14.08	49.66	30.43	14.06	49.67	30.45
66	1	14.42	47.66	29.02	14.61	47.54	29.04	14.73	47.45	29.06
67*	1	13.80	44.94	28.50	13.80	44.94	28.50	13.80	44.94	28.50
68*	1	12.41	45.72	29.23	12.41	45.72	29.23	12.41	45.72	29.23
69*	1	13.11	46.37	27.76	13.11	46.37	27.76	13.11	46.37	27.76
70	1	16.73	49.54	32.94	16.52	49.48	33.33	16.50	49.48	33.38
71	1	17.17	51.95	33.61	16.79	51.83	34.27	16.77	51.83	34.30
72	1	15.18	53.47	33.11	14.96	53.40	33.42	14.98	53.42	33.41
73	1	11.86	51.68	34.48	11.89	51.70	34.52	11.97	51.76	34.55
74	1	9.25	55.44	31.69	9.24	55.49	31.82	9.23	55.51	31.89
75	1	9.44	53.93	30.80	9.36	53.98	30.92	9.33	54.00	30.99
76	1	8.85	54.64	34.33	8.91	54.69	34.46	8.99	54.74	34.54
77	1	10.04	52.90	35.78	10.13	52.94	35.87	10.26	53.02	35.93
78	1	11.86	54.27	30.63	11.79	54.29	30.66	11.75	54.31	30.66
79	1	11.66	57.10	31.32	11.62	57.11	31.44	11.59	57.12	31.51
80	1	11.14	53.95	28.51	11.12	54.01	28.54	11.14	54.08	28.54
81	1	13.39	58.00	32.49	13.38	58.00	32.52	13.37	57.99	32.57
82	1	14.85	57.18	31.88	14.83	57.21	31.87	14.81	57.24	31.85
83	1	14.30	56.88	33.53	14.32	56.86	33.52	14.35	56.82	33.50
84	1	9.62	56.19	27.29	9.65	56.29	27.32	9.69	56.38	27.33
85	1	11.91	56.56	26.60	11.97	56.60	26.67	12.00	56.64	26.68
86	1	10.89	56.22	25.22	10.97	56.29	25.27	11.01	56.33	25.27
87	1	9.19	55.08	24.75	9.11	55.36	24.81	9.03	55.58	24.87
88	1	7.39	52.84	25.49	7.17	53.22	25.41	8.17	52.86	24.57
89	1	7.22	53.84	24.03	7.29	54.17	23.91	6.85	53.80	25.30
90	1	8.55	52.65	24.16	8.42	52.82	24.22	7.53	54.28	23.72
91	1	14.15	54.23	24.97	14.17	54.25	25.06	14.18	54.28	25.06
92	1	13.87	55.89	25.47	13.90	55.92	25.52	13.91	55.96	25.46
93	1	19.06	49.67	30.20	19.17	50.05	30.57	19.12	50.37	31.18
94*	1	15.76	47.98	31.20	15.76	47.98	31.20	15.76	47.98	31.20

95	6	14.98	51.41	24.40	15.00	51.42	24.41	15.00	51.41	24.41
96	8	15.30	52.81	24.37	15.31	52.82	24.40	15.29	52.82	24.39
97	6	13.81	51.17	23.46	13.84	51.19	23.45	13.85	51.17	23.45
98	1	13.52	50.12	23.46	13.55	50.13	23.44	13.57	50.11	23.44
99	1	12.94	51.77	23.76	12.97	51.78	23.75	12.97	51.75	23.74
100	1	14.09	51.46	22.44	14.13	51.49	22.44	14.14	51.46	22.44
101	1	15.86	50.86	24.03	15.88	50.87	24.05	15.89	50.88	24.06
102	1	16.17	52.87	24.81	16.17	52.89	24.85	16.15	52.91	24.83

M121L – OX

Atom number	Atomic number	ε = 1			ε = 5.7			ε = 78.4		
		X	Y	Z	X	Y	Z	X	Y	Z
1	29	12.83	52.53	31.37	12.84	52.58	31.35	12.86	52.61	31.33
2	16	12.28	52.20	29.30	12.29	52.23	29.27	12.28	52.26	29.25
3	6	11.17	50.79	28.86	11.21	50.78	28.86	11.21	50.80	28.86
4*	6	11.21	50.54	27.34	11.21	50.54	27.34	11.21	50.54	27.34
5	6	12.64	50.15	26.94	12.62	50.14	26.89	12.62	50.14	26.89
6	8	13.11	49.04	27.21	13.06	49.00	27.09	13.06	49.01	27.09
7	7	13.38	51.13	26.34	13.37	51.13	26.32	13.37	51.13	26.31
8*	6	14.73	50.89	25.85	14.73	50.89	25.85	14.73	50.89	25.85
9	6	15.83	51.52	26.70	15.83	51.50	26.74	15.83	51.49	26.75
10	8	16.97	51.67	26.22	17.00	51.53	26.34	17.00	51.51	26.34
11	7	15.51	51.94	27.94	15.46	52.04	27.92	15.46	52.04	27.92
12*	6	16.49	52.55	28.82	16.48	52.55	28.82	16.48	52.55	28.82
13	6	17.49	51.55	29.46	17.38	51.40	29.34	17.39	51.40	29.33
14	8	17.54	51.43	30.68	16.92	50.29	29.59	16.97	50.25	29.46
15	7	18.31	50.90	28.59	18.67	51.75	29.56	18.63	51.79	29.69
16	6	19.33	49.97	29.03	19.67	50.82	30.07	19.61	50.88	30.27
17*	6	14.76	47.95	31.60	14.76	47.95	31.60	14.76	47.95	31.60
18	6	13.86	46.96	30.85	13.89	46.93	30.86	13.89	46.93	30.86
19	6	14.16	49.36	31.43	14.19	49.37	31.39	14.19	49.37	31.39
20	8	12.89	46.46	31.42	12.97	46.35	31.45	12.98	46.33	31.45
21	7	14.16	46.73	29.54	14.15	46.75	29.54	14.15	46.75	29.54
22	6	14.84	50.43	32.24	14.84	50.44	32.23	14.84	50.44	32.22
23*	6	13.32	45.88	28.69	13.32	45.88	28.69	13.32	45.88	28.69
24	6	15.98	50.36	32.99	15.95	50.36	33.03	15.95	50.36	33.03
25	7	14.41	51.77	32.24	14.41	51.77	32.23	14.42	51.78	32.21
26	7	16.24	51.64	33.43	16.19	51.63	33.51	16.19	51.64	33.49
27	6	15.30	52.46	32.95	15.25	52.45	33.01	15.26	52.46	32.98
28	7	11.72	53.44	32.75	11.77	53.46	32.76	11.80	53.47	32.77
29	6	10.68	54.33	32.49	10.71	54.35	32.53	10.74	54.34	32.58
30	6	11.88	53.40	34.07	11.96	53.43	34.07	12.03	53.42	34.08
31	6	10.25	54.73	31.11	10.24	54.74	31.17	10.22	54.75	31.23
32	6	10.22	54.81	33.69	10.28	54.82	33.74	10.34	54.81	33.80
33	7	10.98	54.21	34.67	11.08	54.23	34.70	11.16	54.21	34.73

34	6	11.41	55.36	30.28	11.37	55.38	30.31	11.34	55.39	30.35
35	7	12.17	56.32	31.04	12.16	56.34	31.06	12.14	56.35	31.08
36	6	10.86	56.03	29.02	10.80	56.05	29.05	10.74	56.05	29.10
37	6	13.32	55.99	31.67	13.32	56.00	31.64	13.32	56.01	31.63
38	8	10.50	57.21	29.04	10.44	57.23	29.07	10.34	57.22	29.12
39	7	10.79	55.20	27.96	10.73	55.23	27.99	10.70	55.23	28.02
40*	6	13.96	57.07	32.51	13.96	57.07	32.51	13.96	57.07	32.51
41	8	13.81	54.84	31.61	13.82	54.86	31.53	13.83	54.88	31.48
42	6	10.08	55.52	26.74	10.11	55.58	26.73	10.09	55.59	26.76
43	6	9.07	54.37	26.47	9.09	54.47	26.36	9.07	54.50	26.37
44	6	11.06	55.81	25.57	11.16	55.87	25.64	11.16	55.89	25.69
45	8	8.99	53.40	27.22	8.91	53.50	27.09	8.86	53.52	27.08
46	7	8.28	54.55	25.38	8.40	54.68	25.21	8.41	54.71	25.20
47	6	12.04	54.66	25.33	12.13	54.71	25.42	12.12	54.72	25.45
48	6	7.25	53.60	24.99	7.37	53.76	24.74	7.38	53.80	24.73
49	7	13.13	54.95	24.58	13.28	55.01	24.79	13.29	55.02	24.87
50	8	11.83	53.54	25.79	11.84	53.57	25.79	11.81	53.57	25.78
51*	6	9.90	47.88	31.09	9.90	47.88	31.09	9.90	47.88	31.09
52*	6	9.41	46.46	30.92	9.41	46.46	30.92	9.41	46.46	30.92
53*	6	10.16	48.22	32.54	10.16	48.22	32.54	10.16	48.22	32.54
54	6	9.52	50.74	32.29	9.48	50.72	32.30	9.47	50.72	32.31
55	1	11.47	49.90	29.41	11.56	49.90	29.39	11.57	49.92	29.40
56	1	10.16	51.07	29.16	10.20	51.02	29.19	10.20	51.02	29.20
57*	1	10.85	51.40	26.82	10.85	51.40	26.82	10.85	51.40	26.82
58*	1	10.60	49.70	27.10	10.60	49.70	27.10	10.60	49.70	27.10
59	1	12.93	52.02	26.11	12.94	52.03	26.14	12.93	52.04	26.13
60*	1	14.85	49.83	25.80	14.85	49.83	25.80	14.85	49.83	25.80
61	1	14.55	51.84	28.26	14.50	51.95	28.25	14.50	51.98	28.25
62*	1	17.09	53.28	28.31	17.09	53.28	28.31	17.09	53.28	28.31
63*	1	16.04	52.98	29.69	16.04	52.98	29.69	16.04	52.98	29.69
64	1	18.19	51.10	27.60	19.00	52.64	29.21	18.92	52.74	29.49
65	1	19.19	48.99	28.56	20.34	50.47	29.27	20.31	50.52	29.51
66	1	20.33	50.33	28.78	20.26	51.29	30.85	20.18	51.39	31.05
67*	1	14.78	47.62	32.62	14.78	47.62	32.62	14.78	47.62	32.62
68	1	13.10	49.32	31.68	13.12	49.37	31.57	13.11	49.37	31.57
69	1	14.21	49.64	30.37	14.34	49.64	30.34	14.32	49.64	30.33
70	1	14.92	47.23	29.12	14.88	47.29	29.10	14.89	47.28	29.10
71*	1	13.80	44.94	28.50	13.80	44.94	28.50	13.80	44.94	28.50
72*	1	12.41	45.72	29.23	12.41	45.72	29.23	12.41	45.72	29.23
73*	1	13.11	46.37	27.76	13.11	46.37	27.76	13.11	46.37	27.76
74	1	16.63	49.53	33.21	16.58	49.53	33.29	16.58	49.53	33.29
75	1	17.08	51.93	33.91	16.94	51.91	34.12	16.94	51.92	34.11
76	1	15.27	53.53	33.08	15.20	53.52	33.19	15.22	53.53	33.16
77	1	12.60	52.81	34.60	12.71	52.85	34.59	12.80	52.85	34.57
78	1	9.43	55.45	31.20	9.42	55.45	31.28	9.41	55.47	31.37
79	1	9.87	53.86	30.55	9.85	53.88	30.61	9.82	53.89	30.68
80	1	9.43	55.50	33.92	9.50	55.52	33.99	9.56	55.50	34.08
81	1	10.87	54.33	35.67	11.01	54.36	35.70	11.13	54.34	35.73
82	1	12.10	54.57	30.01	12.05	54.59	30.02	12.02	54.60	30.05

83	1	11.85	57.28	31.01	11.80	57.29	31.11	11.76	57.29	31.20
84	1	11.05	54.23	28.07	11.02	54.26	28.08	11.02	54.28	28.11
85	1	13.55	58.06	32.31	13.51	58.06	32.36	13.54	58.06	32.35
86	1	15.04	57.08	32.33	15.03	57.12	32.30	15.03	57.09	32.32
87	1	13.79	56.83	33.56	13.84	56.78	33.56	13.81	56.79	33.55
88	1	9.51	56.44	26.93	9.55	56.51	26.90	9.54	56.52	26.93
89	1	11.61	56.72	25.80	11.72	56.77	25.92	11.73	56.77	25.99
90	1	10.51	56.00	24.64	10.67	56.11	24.68	10.70	56.14	24.73
91	1	8.35	55.41	24.86	8.51	55.56	24.73	8.52	55.59	24.72
92	1	7.34	52.73	25.65	7.61	52.76	25.11	7.72	52.77	24.86
93	1	6.25	54.03	25.11	6.38	54.04	25.11	6.44	53.92	25.29
94	1	7.38	53.28	23.95	7.36	53.75	23.65	7.19	53.99	23.68
95	1	13.81	54.21	24.40	13.95	54.26	24.60	13.95	54.27	24.67
96	1	13.28	55.88	24.22	13.49	55.95	24.49	13.52	55.97	24.61
97*	1	10.85	47.93	30.60	10.85	47.93	30.60	10.85	47.93	30.60
98*	1	9.10	48.52	30.77	9.10	48.52	30.77	9.10	48.52	30.77
99*	1	8.88	46.16	31.80	8.88	46.16	31.80	8.88	46.16	31.80
100*	1	10.24	45.81	30.77	10.24	45.81	30.77	10.24	45.81	30.77
101*	1	8.75	46.41	30.08	8.75	46.41	30.08	8.75	46.41	30.08
102*	1	9.24	48.04	33.05	9.24	48.04	33.05	9.24	48.04	33.05
103*	1	11.04	47.66	32.81	11.04	47.66	32.81	11.04	47.66	32.81
104	1	9.41	51.68	32.86	9.41	51.67	32.85	9.41	51.67	32.85
105	1	8.51	50.40	32.04	8.45	50.38	32.12	8.44	50.37	32.14
106	1	10.03	50.98	31.36	9.93	50.93	31.33	9.92	50.93	31.33
107	1	19.25	49.86	30.11	19.14	49.95	30.47	19.08	50.03	30.69
108*	1	15.76	47.98	31.20	15.76	47.98	31.21	15.76	47.98	31.21
109	6	14.93	51.38	24.38	14.98	51.40	24.40	14.98	51.40	24.40
110	8	15.10	52.81	24.33	15.20	52.82	24.36	15.19	52.82	24.36
111	6	13.78	51.01	23.46	13.84	51.08	23.45	13.85	51.07	23.44
112	1	13.61	49.93	23.48	13.63	50.00	23.45	13.65	49.99	23.44
113	1	12.85	51.51	23.76	12.92	51.60	23.73	12.93	51.59	23.72
114	1	14.02	51.30	22.44	14.11	51.38	22.43	14.12	51.37	22.43
115	1	15.85	50.91	24.03	15.89	50.90	24.06	15.90	50.91	24.07
116	1	15.98	52.98	24.69	16.06	52.97	24.78	16.05	52.99	24.78
117	6	10.28	49.69	33.10	10.28	49.69	33.10	10.28	49.69	33.10
118	6	11.68	50.16	33.52	11.69	50.18	33.45	11.69	50.19	33.44
119	1	12.17	50.76	32.75	12.14	50.76	32.65	12.14	50.76	32.63
120	1	11.61	50.79	34.41	11.65	50.84	34.32	11.64	50.85	34.31
121	1	12.33	49.32	33.78	12.36	49.35	33.70	12.36	49.36	33.70
122	1	9.74	49.62	34.06	9.78	49.61	34.07	9.79	49.61	34.07

M121L – RED

Atom number	Atomic number	ε = 1			ε = 5.7			ε = 78.4		
		X	Y	Z	X	Y	Z	X	Y	Z
1	29	12.77	52.60	31.47	12.77	52.64	31.45	12.77	52.66	31.46
2	16	12.41	52.18	29.25	12.37	52.22	29.25	12.35	52.24	29.26
3	6	11.21	50.83	28.85	11.22	50.83	28.85	11.22	50.83	28.85
4*	6	11.21	50.54	27.34	11.21	50.54	27.34	11.21	50.54	27.34
5	6	12.63	50.14	26.92	12.62	50.14	26.90	12.63	50.15	26.90
6	8	13.10	49.03	27.18	13.07	49.01	27.12	13.08	49.02	27.13
7	7	13.37	51.11	26.29	13.36	51.11	26.29	13.36	51.12	26.29
8*	6	14.73	50.89	25.85	14.73	50.89	25.85	14.73	50.89	25.85
9	6	15.81	51.50	26.73	15.81	51.50	26.76	15.82	51.49	26.76
10	8	16.97	51.61	26.27	16.98	51.57	26.34	16.99	51.53	26.35
11	7	15.49	51.94	27.96	15.45	52.02	27.95	15.45	52.03	27.94
12*	6	16.49	52.55	28.82	16.49	52.55	28.81	16.48	52.55	28.81
13	6	17.54	51.60	29.46	17.41	51.42	29.32	17.40	51.42	29.33
14	8	17.85	51.76	30.64	17.03	50.26	29.47	17.04	50.24	29.40
15	7	18.11	50.67	28.66	18.66	51.84	29.67	18.62	51.83	29.75
16	6	19.18	49.82	29.14	19.68	50.94	30.18	19.59	50.92	30.34
17*	6	14.76	47.95	31.60	14.76	47.95	31.60	14.76	47.95	31.60
18	6	13.93	46.88	30.86	13.94	46.88	30.86	13.92	46.90	30.86
19	6	14.16	49.35	31.41	14.19	49.37	31.38	14.19	49.37	31.39
20	8	13.12	46.18	31.47	13.13	46.18	31.47	13.07	46.23	31.46
21	7	14.15	46.76	29.52	14.14	46.77	29.52	14.14	46.76	29.53
22	6	14.90	50.43	32.15	14.87	50.43	32.20	14.87	50.43	32.20
23*	6	13.32	45.88	28.69	13.32	45.88	28.69	13.32	45.88	28.69
24	6	16.09	50.34	32.83	15.97	50.31	33.02	15.96	50.32	33.03
25	7	14.47	51.76	32.17	14.46	51.76	32.18	14.46	51.77	32.17
26	7	16.38	51.61	33.26	16.24	51.58	33.48	16.23	51.60	33.49
27	6	15.40	52.44	32.82	15.31	52.42	32.96	15.31	52.43	32.95
28	7	11.60	53.47	32.82	11.64	53.50	32.86	11.66	53.51	32.89
29	6	10.56	54.37	32.61	10.59	54.39	32.69	10.60	54.41	32.77
30	6	11.78	53.43	34.13	11.86	53.43	34.16	11.93	53.44	34.19
31	6	10.14	54.76	31.22	10.11	54.81	31.34	10.08	54.84	31.43
32	6	10.11	54.84	33.81	10.18	54.85	33.92	10.24	54.86	34.01
33	7	10.89	54.23	34.77	11.00	54.23	34.84	11.09	54.23	34.90
34	6	11.37	55.27	30.41	11.30	55.30	30.47	11.24	55.32	30.53
35	7	12.14	56.25	31.12	12.11	56.27	31.16	12.08	56.29	31.19
36	6	10.91	55.86	29.08	10.80	55.91	29.16	10.71	55.91	29.22
37	6	13.32	55.95	31.72	13.31	55.96	31.70	13.29	55.98	31.70
38	8	10.65	57.06	28.98	10.45	57.09	29.11	10.27	57.07	29.19
39	7	10.79	54.96	28.08	10.75	55.05	28.12	10.75	55.09	28.16
40*	6	13.96	57.07	32.51	13.96	57.07	32.51	13.96	57.07	32.51
41	8	13.82	54.82	31.69	13.83	54.84	31.61	13.81	54.86	31.56
42	6	10.20	55.33	26.81	10.21	55.45	26.83	10.22	55.48	26.87
43	6	9.28	54.18	26.34	9.22	54.38	26.32	9.23	54.41	26.36
44	6	11.29	55.77	25.80	11.34	55.84	25.85	11.35	55.86	25.89
45	8	8.99	53.25	27.07	8.92	53.41	27.01	8.90	53.44	27.04

46	7	8.72	54.35	25.10	8.66	54.65	25.12	8.68	54.65	25.14
47	6	12.24	54.63	25.43	12.26	54.68	25.50	12.27	54.69	25.54
48	6	7.91	53.31	24.50	7.69	53.77	24.48	7.71	53.75	24.55
49	7	13.50	54.97	25.09	13.53	54.99	25.16	13.53	55.01	25.20
50	8	11.83	53.46	25.40	11.85	53.51	25.52	11.86	53.52	25.57
51*	6	9.90	47.88	31.09	9.90	47.88	31.09	9.90	47.88	31.09
52*	6	9.41	46.46	30.92	9.41	46.46	30.92	9.41	46.46	30.92
53*	6	10.16	48.22	32.54	10.16	48.22	32.54	10.16	48.22	32.54
54	6	9.50	50.73	32.27	9.48	50.72	32.29	9.46	50.72	32.31
55	1	11.48	49.93	29.40	11.53	49.93	29.39	11.53	49.93	29.40
56	1	10.20	51.13	29.15	10.21	51.08	29.17	10.20	51.07	29.17
57*	1	10.85	51.40	26.82	10.85	51.40	26.82	10.85	51.40	26.82
58*	1	10.60	49.70	27.10	10.60	49.70	27.10	10.60	49.70	27.10
59	1	12.92	52.00	26.09	12.93	52.02	26.12	12.92	52.02	26.11
60*	1	14.85	49.83	25.80	14.85	49.83	25.80	14.85	49.83	25.80
61	1	14.52	51.87	28.30	14.49	51.94	28.30	14.48	51.97	28.28
62*	1	17.09	53.28	28.31	17.09	53.28	28.31	17.09	53.28	28.31
63*	1	16.04	52.98	29.69	16.04	52.98	29.69	16.04	52.98	29.69
64	1	17.95	50.78	27.66	18.94	52.77	29.41	18.89	52.80	29.63
65	1	19.24	48.92	28.53	20.38	50.62	29.40	19.95	50.19	29.60
66	1	20.16	50.32	29.12	20.24	51.42	30.99	20.43	51.49	30.72
67*	1	14.78	47.62	32.62	14.78	47.62	32.62	14.78	47.62	32.62
68	1	13.11	49.34	31.70	13.12	49.38	31.58	13.12	49.38	31.59
69	1	14.16	49.61	30.34	14.30	49.64	30.33	14.30	49.63	30.33
70	1	14.65	47.50	29.04	14.72	47.45	29.06	14.81	47.37	29.07
71*	1	13.80	44.94	28.50	13.80	44.94	28.50	13.80	44.94	28.50
72*	1	12.41	45.72	29.23	12.41	45.72	29.23	12.41	45.72	29.23
73*	1	13.11	46.37	27.76	13.11	46.37	27.76	13.11	46.37	27.76
74	1	16.74	49.51	33.02	16.58	49.47	33.29	16.57	49.48	33.31
75	1	17.27	51.92	33.62	16.99	51.85	34.10	16.97	51.86	34.12
76	1	15.38	53.51	32.97	15.28	53.49	33.13	15.28	53.49	33.13
77	1	12.52	52.83	34.65	12.62	52.84	34.64	12.70	52.85	34.64
78	1	9.37	55.54	31.28	9.35	55.59	31.44	9.34	55.64	31.57
79	1	9.73	53.90	30.69	9.64	53.96	30.81	9.57	54.01	30.92
80	1	9.33	55.54	34.06	9.41	55.54	34.21	9.49	55.56	34.33
81	1	10.81	54.35	35.77	10.96	54.33	35.84	11.10	54.34	35.90
82	1	12.00	54.39	30.27	11.92	54.42	30.30	11.86	54.44	30.35
83	1	11.83	57.21	31.04	11.76	57.22	31.20	11.72	57.23	31.30
84	1	11.07	53.99	28.25	11.08	54.09	28.26	11.12	54.14	28.29
85	1	13.56	58.06	32.26	13.55	58.06	32.28	13.53	58.06	32.32
86	1	15.04	57.06	32.34	15.03	57.07	32.32	15.03	57.09	32.29
87	1	13.79	56.89	33.57	13.81	56.86	33.57	13.84	56.84	33.57
88	1	9.57	56.21	26.99	9.63	56.36	27.02	9.64	56.39	27.05
89	1	11.84	56.61	26.25	11.91	56.66	26.29	11.92	56.68	26.32
90	1	10.84	56.16	24.88	10.92	56.22	24.91	10.93	56.24	24.95
91	1	9.12	55.04	24.48	8.95	55.48	24.61	8.96	55.47	24.62
92	1	7.42	52.75	25.30	7.45	52.98	25.19	8.17	52.79	24.27
93	1	7.15	53.75	23.85	6.78	54.32	24.22	6.90	53.55	25.25
94	1	8.52	52.61	23.91	8.10	53.32	23.57	7.30	54.22	23.65

95	1	14.15	54.23	24.81	14.17	54.24	24.88	14.17	54.26	24.90
96	1	13.82	55.92	25.17	13.85	55.95	25.18	13.85	55.97	25.18
97*	1	10.85	47.93	30.60	10.85	47.93	30.60	10.85	47.93	30.60
98*	1	9.10	48.52	30.77	9.10	48.52	30.77	9.10	48.52	30.77
99*	1	8.88	46.16	31.80	8.88	46.16	31.80	8.88	46.16	31.80
100*	1	10.24	45.81	30.77	10.24	45.81	30.77	10.24	45.81	30.77
101*	1	8.75	46.41	30.08	8.75	46.41	30.08	8.75	46.41	30.08
102*	1	9.24	48.04	33.05	9.24	48.04	33.05	9.24	48.04	33.05
103*	1	11.04	47.66	32.81	11.04	47.66	32.81	11.04	47.66	32.81
104	1	9.40	51.67	32.82	9.41	51.67	32.83	9.40	51.67	32.85
105	1	8.49	50.38	32.04	8.46	50.37	32.11	8.43	50.36	32.15
106	1	10.01	50.97	31.33	9.94	50.94	31.32	9.90	50.92	31.33
107	1	18.98	49.54	30.18	19.17	50.05	30.58	19.13	50.36	31.16
108*	1	15.76	47.98	31.20	15.76	47.98	31.21	15.76	47.98	31.21
109	6	14.99	51.39	24.39	15.01	51.40	24.40	15.01	51.40	24.40
110	8	15.32	52.79	24.35	15.33	52.80	24.37	15.31	52.81	24.37
111	6	13.82	51.15	23.45	13.85	51.16	23.44	13.86	51.15	23.44
112	1	13.53	50.10	23.46	13.57	50.11	23.44	13.58	50.09	23.44
113	1	12.95	51.75	23.74	12.98	51.76	23.73	12.98	51.74	23.72
114	1	14.11	51.43	22.43	14.15	51.45	22.43	14.16	51.43	22.43
115	1	15.87	50.84	24.04	15.89	50.84	24.06	15.90	50.86	24.06
116	1	16.18	52.85	24.80	16.18	52.87	24.84	16.17	52.90	24.82
117	6	10.28	49.70	33.09	10.29	49.69	33.09	10.29	49.69	33.09
118	6	11.68	50.19	33.49	11.70	50.20	33.44	11.69	50.21	33.43
119	1	12.13	50.86	32.75	12.12	50.87	32.69	12.11	50.87	32.67
120	1	11.61	50.77	34.42	11.66	50.78	34.37	11.65	50.80	34.35
121	1	12.36	49.36	33.69	12.39	49.37	33.62	12.39	49.39	33.62
122	1	9.75	49.63	34.05	9.79	49.62	34.06	9.80	49.61	34.07

M121V – OX

Atom number	Atomic number	ε = 1			ε = 5.7			ε = 78.4		
		X	Y	Z	X	Y	Z	X	Y	Z
1	29	12.71	52.44	31.40	12.65	52.44	31.42	12.67	52.46	31.41
2	16	12.28	52.22	29.29	12.26	52.23	29.30	12.27	52.25	29.28
3	6	11.16	50.79	28.86	11.20	50.78	28.86	11.20	50.79	28.86
4*	6	11.21	50.54	27.34	11.21	50.54	27.34	11.21	50.54	27.34
5	6	12.65	50.15	26.95	12.63	50.14	26.90	12.63	50.15	26.90
6	8	13.12	49.05	27.25	13.07	49.01	27.13	13.07	49.01	27.12
7	7	13.38	51.13	26.34	13.37	51.13	26.32	13.37	51.13	26.32
8*	6	14.73	50.89	25.85	14.73	50.89	25.85	14.73	50.89	25.85
9	6	15.83	51.53	26.70	15.83	51.49	26.75	15.83	51.48	26.75
10	8	16.97	51.67	26.23	17.00	51.52	26.35	17.00	51.49	26.35
11	7	15.50	51.95	27.93	15.46	52.04	27.93	15.46	52.04	27.93
12*	6	16.49	52.55	28.82	16.49	52.55	28.81	16.49	52.55	28.82
13	6	17.45	51.51	29.46	17.36	51.40	29.35	17.38	51.40	29.34
14	8	17.39	51.27	30.66	16.87	50.31	29.66	16.94	50.26	29.51

15	7	18.34	50.95	28.61	18.67	51.71	29.53	18.64	51.78	29.66
16	6	19.31	49.95	29.04	19.65	50.77	30.04	19.61	50.87	30.25
17*	6	14.76	47.95	31.60	14.76	47.95	31.60	14.76	47.95	31.60
18	6	13.95	46.87	30.86	13.97	46.85	30.86	13.98	46.85	30.86
19	6	14.07	49.32	31.50	14.09	49.33	31.46	14.09	49.33	31.46
20	8	13.15	46.16	31.47	13.22	46.09	31.48	13.25	46.06	31.48
21	7	14.15	46.77	29.52	14.14	46.78	29.51	14.13	46.79	29.51
22	6	14.70	50.38	32.35	14.65	50.37	32.39	14.66	50.38	32.37
23*	6	13.32	45.88	28.69	13.32	45.88	28.69	13.32	45.88	28.69
24	6	15.84	50.33	33.11	15.72	50.31	33.25	15.73	50.32	33.22
25	7	14.21	51.69	32.40	14.12	51.67	32.46	14.15	51.68	32.43
26	7	16.03	51.60	33.62	15.83	51.55	33.83	15.86	51.57	33.79
27	6	15.04	52.39	33.17	14.86	52.35	33.33	14.89	52.36	33.29
28	7	11.38	52.96	32.74	11.35	52.98	32.77	11.38	52.99	32.78
29	6	10.37	53.91	32.61	10.35	53.95	32.67	10.38	53.96	32.71
30	6	11.33	52.50	33.98	11.32	52.53	34.02	11.39	52.54	34.03
31	6	10.03	54.62	31.33	9.99	54.66	31.39	9.98	54.67	31.45
32	6	9.72	54.00	33.82	9.75	54.07	33.89	9.80	54.07	33.94
33	7	10.34	53.10	34.66	10.37	53.17	34.72	10.45	53.16	34.76
34	6	11.23	55.26	30.59	11.19	55.28	30.63	11.16	55.29	30.66
35	7	11.97	56.22	31.38	11.96	56.23	31.41	11.94	56.24	31.42
36	6	10.74	55.96	29.32	10.70	55.98	29.35	10.65	55.99	29.39
37	6	13.17	55.91	31.91	13.16	55.92	31.92	13.17	55.92	31.90
38	8	10.36	57.13	29.35	10.35	57.16	29.37	10.23	57.15	29.43
39	7	10.77	55.17	28.22	10.70	55.19	28.25	10.71	55.23	28.27
40*	6	13.96	57.07	32.51	13.96	57.07	32.51	13.96	57.07	32.51
41	8	13.62	54.75	31.92	13.61	54.75	31.89	13.62	54.76	31.84
42	6	10.07	55.51	26.99	10.13	55.58	26.98	10.18	55.65	26.99
43	6	9.08	54.36	26.68	9.13	54.49	26.53	9.15	54.60	26.50
44	6	11.06	55.83	25.85	11.23	55.92	25.95	11.31	55.97	25.99
45	8	8.96	53.39	27.43	8.91	53.49	27.22	8.83	53.63	27.18
46	7	8.33	54.53	25.56	8.51	54.73	25.35	8.61	54.85	25.28
47	6	12.02	54.69	25.53	12.16	54.75	25.65	12.20	54.78	25.68
48	6	7.31	53.57	25.14	7.50	53.82	24.81	7.58	53.99	24.72
49	7	13.11	55.00	24.80	13.34	55.05	25.07	13.38	55.06	25.08
50	8	11.79	53.54	25.93	11.84	53.59	25.93	11.85	53.62	25.95
51	6	9.86	47.92	31.15	9.86	47.92	31.15	9.86	47.92	31.15
52*	6	9.41	46.46	30.92	9.41	46.46	30.92	9.41	46.46	30.92
53	6	10.55	48.06	32.52	10.57	48.06	32.50	10.57	48.06	32.50
54	1	10.89	49.09	32.70	10.91	49.10	32.67	10.90	49.09	32.67
55	6	8.68	48.89	31.04	8.66	48.88	31.06	8.66	48.88	31.07
56	1	11.48	49.91	29.41	11.56	49.90	29.39	11.57	49.91	29.39
57	1	10.15	51.07	29.16	10.18	51.00	29.20	10.19	51.00	29.20
58*	1	10.85	51.40	26.82	10.85	51.40	26.82	10.85	51.40	26.82
59*	1	10.60	49.70	27.10	10.60	49.70	27.10	10.60	49.70	27.10
60	1	12.93	52.02	26.13	12.93	52.04	26.15	12.94	52.04	26.15
61*	1	14.85	49.83	25.80	14.85	49.83	25.80	14.85	49.83	25.80
62	1	14.54	51.84	28.26	14.50	51.96	28.25	14.50	51.98	28.25
63*	1	17.09	53.28	28.31	17.09	53.28	28.31	17.09	53.28	28.31

64*	1	16.04	52.98	29.69	16.04	52.98	29.69	16.04	52.98	29.69
65	1	18.25	51.18	27.62	19.01	52.57	29.13	18.94	52.71	29.43
66	1	19.04	48.95	28.68	20.29	50.37	29.25	20.33	50.51	29.50
67	1	20.31	50.20	28.67	20.28	51.24	30.80	20.16	51.37	31.05
68*	1	14.78	47.62	32.62	14.78	47.62	32.62	14.78	47.62	32.62
69	1	13.02	49.20	31.79	13.02	49.23	31.65	13.02	49.23	31.67
70	1	14.07	49.66	30.45	14.21	49.69	30.43	14.17	49.68	30.43
71	1	14.70	47.47	29.05	14.69	47.48	29.05	14.68	47.49	29.04
72*	1	13.80	44.94	28.50	13.80	44.94	28.50	13.80	44.94	28.50
73*	1	12.41	45.72	29.23	12.41	45.72	29.23	12.41	45.72	29.23
74*	1	13.11	46.37	27.76	13.11	46.37	27.76	13.11	46.37	27.76
75	1	16.53	49.53	33.30	16.39	49.50	33.48	16.40	49.51	33.47
76	1	16.83	51.90	34.16	16.53	51.83	34.50	16.56	51.85	34.47
77	1	14.96	53.45	33.36	14.73	53.39	33.58	14.76	53.40	33.54
78	1	11.99	51.75	34.40	11.97	51.77	34.43	12.06	51.78	34.43
79	1	9.28	55.39	31.55	9.26	55.44	31.63	9.26	55.45	31.70
80	1	9.56	53.92	30.63	9.50	53.96	30.71	9.47	53.97	30.77
81	1	8.89	54.61	34.13	8.94	54.71	34.22	9.00	54.69	34.30
82	1	10.09	52.92	35.62	10.14	53.00	35.70	10.26	53.00	35.74
83	1	11.92	54.46	30.34	11.86	54.48	30.37	11.83	54.49	30.39
84	1	11.68	57.19	31.28	11.66	57.20	31.35	11.63	57.20	31.41
85	1	11.04	54.20	28.31	10.98	54.21	28.34	11.05	54.27	28.34
86	1	13.41	58.01	32.49	13.35	57.97	32.64	13.40	58.01	32.52
87	1	14.89	57.19	31.94	14.79	57.30	31.84	14.88	57.20	31.93
88	1	14.23	56.83	33.54	14.38	56.77	33.47	14.25	56.81	33.53
89	1	9.50	56.42	27.19	9.56	56.50	27.16	9.63	56.58	27.18
90	1	11.63	56.73	26.13	11.81	56.77	26.32	11.91	56.79	26.40
91	1	10.53	56.10	24.93	10.78	56.25	25.00	10.89	56.35	25.05
92	1	8.43	55.38	25.03	8.64	55.61	24.89	8.81	55.72	24.81
93	1	7.31	52.76	25.86	7.73	52.81	25.15	7.87	52.94	24.82
94	1	6.32	54.04	25.12	6.49	54.09	25.14	6.61	54.13	25.22
95	1	7.53	53.18	24.14	7.54	53.85	23.72	7.46	54.23	23.66
96	1	13.78	54.26	24.56	13.97	54.30	24.80	14.00	54.29	24.82
97	1	13.29	55.94	24.48	13.58	56.01	24.83	13.64	56.01	24.87
98	1	10.60	48.18	30.37	10.56	48.19	30.35	10.56	48.19	30.36
99*	1	8.88	46.16	31.80	8.88	46.16	31.80	8.88	46.16	31.80
100*	1	10.24	45.81	30.77	10.24	45.81	30.77	10.24	45.81	30.77
101*	1	8.75	46.41	30.08	8.75	46.41	30.08	8.75	46.41	30.08
102	1	9.85	47.80	33.33	9.90	47.80	33.32	9.90	47.80	33.32
103	1	11.42	47.39	32.60	11.45	47.41	32.55	11.45	47.41	32.56
104	1	8.99	49.93	31.22	8.96	49.92	31.24	8.96	49.91	31.25
105	1	7.90	48.64	31.78	7.90	48.62	31.82	7.90	48.62	31.83
106	1	8.22	48.85	30.05	8.17	48.83	30.08	8.17	48.83	30.09
107	1	19.32	49.94	30.13	19.10	49.94	30.50	19.08	50.01	30.65
108*	1	15.76	47.98	31.20	15.76	47.98	31.20	15.76	47.98	31.20
109	6	14.92	51.40	24.39	14.97	51.42	24.41	14.98	51.41	24.41
110	8	15.07	52.83	24.36	15.19	52.84	24.39	15.19	52.83	24.38
111	6	13.78	51.02	23.46	13.83	51.11	23.45	13.85	51.09	23.45
112	1	13.63	49.93	23.46	13.63	50.04	23.44	13.64	50.02	23.44

113	1	12.85	51.50	23.76	12.92	51.63	23.74	12.93	51.62	23.73
114	1	14.02	51.33	22.44	14.11	51.42	22.44	14.12	51.40	22.43
115	1	15.86	50.95	24.03	15.89	50.93	24.06	15.90	50.92	24.07
116	1	15.95	53.02	24.71	16.06	52.99	24.79	16.06	52.99	24.79

M121V – RED

Atom number	Atomic number	ε = 1			ε = 5.7			ε = 78.4		
		X	Y	Z	X	Y	Z	X	Y	Z
1	29	12.63	52.45	31.51	12.57	52.46	31.53	12.57	52.50	31.54
2	16	12.45	52.14	29.28	12.41	52.16	29.30	12.39	52.17	29.31
3	6	11.21	50.83	28.85	11.21	50.82	28.86	11.21	50.81	28.86
4*	6	11.21	50.54	27.34	11.21	50.54	27.34	11.21	50.54	27.34
5	6	12.64	50.14	26.93	12.63	50.14	26.90	12.63	50.15	26.90
6	8	13.11	49.03	27.19	13.07	49.01	27.13	13.08	49.02	27.14
7	7	13.37	51.11	26.30	13.36	51.11	26.29	13.36	51.11	26.29
8*	6	14.73	50.89	25.85	14.73	50.89	25.85	14.73	50.89	25.85
9	6	15.82	51.50	26.73	15.81	51.50	26.76	15.82	51.49	26.76
10	8	16.97	51.62	26.26	16.98	51.56	26.34	16.99	51.53	26.35
11	7	15.49	51.93	27.96	15.45	52.02	27.95	15.45	52.03	27.94
12*	6	16.49	52.55	28.81	16.49	52.55	28.82	16.48	52.55	28.81
13	6	17.54	51.60	29.47	17.41	51.42	29.34	17.41	51.42	29.33
14	8	17.83	51.74	30.65	17.02	50.27	29.50	17.04	50.25	29.42
15	7	18.13	50.69	28.65	18.66	51.84	29.66	18.62	51.84	29.75
16	6	19.19	49.83	29.13	19.68	50.94	30.19	19.59	50.92	30.34
17*	6	14.76	47.95	31.60	14.76	47.95	31.60	14.76	47.95	31.60
18	6	14.00	46.83	30.86	14.00	46.84	30.86	13.99	46.84	30.86
19	6	14.08	49.32	31.48	14.10	49.33	31.46	14.09	49.33	31.47
20	8	13.33	46.00	31.47	13.32	46.01	31.47	13.29	46.03	31.48
21	7	14.14	46.80	29.50	14.13	46.79	29.51	14.13	46.79	29.51
22	6	14.83	50.41	32.20	14.75	50.39	32.31	14.73	50.39	32.32
23*	6	13.32	45.88	28.69	13.32	45.88	28.69	13.32	45.88	28.69
24	6	16.04	50.35	32.82	15.86	50.30	33.10	15.83	50.30	33.14
25	7	14.34	51.71	32.27	14.25	51.69	32.37	14.25	51.69	32.36
26	7	16.30	51.62	33.29	16.04	51.55	33.65	16.01	51.55	33.68
27	6	15.26	52.41	32.92	15.06	52.35	33.18	15.04	52.36	33.19
28	7	11.26	52.89	32.83	11.25	52.92	32.89	11.26	52.96	32.92
29	6	10.27	53.86	32.76	10.27	53.91	32.85	10.28	53.95	32.90
30	6	11.23	52.41	34.06	11.25	52.46	34.13	11.30	52.50	34.16
31	6	9.95	54.59	31.49	9.91	54.64	31.59	9.89	54.67	31.64
32	6	9.65	53.96	33.98	9.70	54.03	34.09	9.75	54.07	34.16
33	7	10.26	53.02	34.80	10.33	53.09	34.89	10.40	53.15	34.94
34	6	11.20	55.13	30.76	11.14	55.16	30.82	11.11	55.18	30.84
35	7	11.94	56.13	31.52	11.92	56.14	31.55	11.91	56.16	31.56
36	6	10.80	55.76	29.43	10.71	55.79	29.49	10.65	55.82	29.53
37	6	13.17	55.87	32.01	13.17	55.88	32.00	13.16	55.89	31.98
38	8	10.48	56.95	29.38	10.32	56.97	29.46	10.21	56.98	29.52

39	7	10.80	54.92	28.38	10.76	54.97	28.42	10.75	55.03	28.43
40*	6	13.96	57.07	32.51	13.96	57.07	32.51	13.96	57.07	32.51
41	8	13.64	54.73	32.06	13.65	54.73	31.99	13.64	54.74	31.93
42	6	10.25	55.32	27.10	10.25	55.40	27.13	10.26	55.47	27.14
43	6	9.30	54.20	26.59	9.28	54.32	26.57	9.27	54.44	26.56
44	6	11.35	55.77	26.12	11.39	55.82	26.18	11.42	55.87	26.19
45	8	8.98	53.26	27.30	8.95	53.34	27.23	8.91	53.44	27.20
46	7	8.75	54.42	25.36	8.75	54.60	25.36	8.76	54.74	25.35
47	6	12.25	54.63	25.65	12.28	54.67	25.73	12.31	54.71	25.77
48	6	7.94	53.40	24.72	7.86	53.66	24.69	7.77	53.88	24.70
49	7	13.52	54.96	25.33	13.55	54.98	25.42	13.57	55.01	25.44
50	8	11.81	53.48	25.54	11.84	53.52	25.63	11.86	53.55	25.72
51	6	9.88	47.91	31.16	9.87	47.92	31.16	9.86	47.92	31.16
52*	6	9.41	46.46	30.92	9.41	46.46	30.92	9.41	46.46	30.92
53	6	10.60	48.01	32.51	10.59	48.03	32.51	10.58	48.05	32.51
54	1	10.95	49.04	32.70	10.93	49.06	32.69	10.91	49.08	32.69
55	6	8.69	48.89	31.10	8.67	48.88	31.10	8.66	48.88	31.09
56	1	11.45	49.92	29.40	11.49	49.90	29.39	11.50	49.90	29.39
57	1	10.21	51.16	29.16	10.21	51.11	29.17	10.20	51.08	29.18
58*	1	10.85	51.40	26.82	10.85	51.40	26.82	10.85	51.40	26.82
59*	1	10.60	49.70	27.10	10.60	49.70	27.10	10.60	49.70	27.10
60	1	12.92	52.00	26.10	12.92	52.02	26.13	12.93	52.02	26.13
61*	1	14.85	49.83	25.80	14.85	49.83	25.80	14.85	49.83	25.80
62	1	14.52	51.85	28.31	14.49	51.92	28.31	14.49	51.95	28.29
63*	1	17.09	53.28	28.31	17.09	53.28	28.31	17.09	53.28	28.31
64*	1	16.04	52.98	29.69	16.04	52.98	29.69	16.04	52.98	29.69
65	1	17.97	50.80	27.65	18.95	52.77	29.39	18.89	52.80	29.61
66	1	19.17	48.88	28.60	20.39	50.64	29.42	19.98	50.21	29.60
67	1	20.19	50.28	28.99	20.23	51.43	31.00	20.42	51.50	30.74
68*	1	14.78	47.62	32.62	14.78	47.62	32.62	14.78	47.62	32.62
69	1	13.06	49.25	31.87	13.04	49.26	31.73	13.03	49.25	31.74
70	1	13.98	49.62	30.42	14.12	49.66	30.42	14.11	49.66	30.43
71	1	14.49	47.62	29.02	14.58	47.57	29.03	14.63	47.53	29.04
72*	1	13.80	44.94	28.50	13.80	44.94	28.50	13.80	44.94	28.50
73*	1	12.41	45.72	29.23	12.41	45.72	29.23	12.41	45.72	29.23
74*	1	13.11	46.37	27.76	13.11	46.37	27.76	13.11	46.37	27.76
75	1	16.75	49.54	32.96	16.53	49.48	33.32	16.49	49.47	33.38
76	1	17.19	51.95	33.62	16.78	51.83	34.27	16.74	51.83	34.33
77	1	15.20	53.47	33.10	14.96	53.40	33.41	14.95	53.41	33.43
78	1	11.88	51.64	34.46	11.90	51.68	34.51	11.96	51.74	34.53
79	1	9.25	55.41	31.70	9.24	55.47	31.83	9.23	55.51	31.89
80	1	9.45	53.91	30.79	9.37	53.97	30.92	9.33	54.00	30.98
81	1	8.84	54.58	34.33	8.92	54.66	34.47	8.98	54.72	34.55
82	1	10.04	52.83	35.76	10.14	52.91	35.87	10.25	52.99	35.93
83	1	11.86	54.27	30.63	11.79	54.29	30.66	11.75	54.32	30.67
84	1	11.65	57.09	31.34	11.61	57.10	31.45	11.58	57.12	31.52
85	1	11.15	53.95	28.51	11.13	54.02	28.54	11.14	54.09	28.54
86	1	13.38	58.00	32.51	13.37	57.99	32.54	13.36	57.98	32.60
87	1	14.83	57.20	31.86	14.82	57.22	31.86	14.79	57.26	31.83

88	1	14.32	56.87	33.52	14.34	56.85	33.51	14.38	56.81	33.48
89	1	9.63	56.20	27.30	9.65	56.29	27.32	9.69	56.39	27.34
90	1	11.93	56.56	26.60	11.97	56.60	26.67	12.01	56.65	26.69
91	1	10.90	56.23	25.22	10.98	56.29	25.27	11.02	56.33	25.28
92	1	9.19	55.10	24.75	9.11	55.37	24.81	9.03	55.59	24.88
93	1	7.40	52.86	25.50	7.17	53.24	25.41	8.17	52.87	24.57
94	1	7.22	53.87	24.04	7.30	54.19	23.91	6.86	53.81	25.30
95	1	8.55	52.68	24.16	8.42	52.84	24.22	7.53	54.30	23.73
96	1	14.15	54.23	24.98	14.17	54.25	25.06	14.18	54.27	25.07
97	1	13.88	55.89	25.48	13.90	55.92	25.52	13.92	55.96	25.48
98	1	10.58	48.19	30.37	10.57	48.20	30.36	10.56	48.20	30.36
99*	1	8.88	46.16	31.80	8.88	46.16	31.80	8.88	46.16	31.80
100*	1	10.24	45.81	30.77	10.24	45.81	30.77	10.24	45.81	30.77
101*	1	8.75	46.41	30.08	8.75	46.41	30.08	8.75	46.41	30.08
102	1	9.92	47.75	33.33	9.92	47.76	33.33	9.90	47.77	33.33
103	1	11.46	47.34	32.55	11.46	47.37	32.55	11.45	47.39	32.56
104	1	9.02	49.92	31.28	8.99	49.92	31.27	8.97	49.91	31.26
105	1	7.94	48.64	31.87	7.93	48.63	31.86	7.91	48.62	31.85
106	1	8.20	48.86	30.13	8.18	48.84	30.12	8.17	48.83	30.11
107	1	19.05	49.66	30.20	19.17	50.06	30.57	19.13	50.35	31.15
108*	1	15.76	47.98	31.20	15.76	47.98	31.20	15.76	47.98	31.20
109	6	14.98	51.41	24.40	15.00	51.42	24.41	15.00	51.42	24.41
110	8	15.30	52.81	24.38	15.31	52.82	24.40	15.30	52.82	24.40
111	6	13.81	51.18	23.46	13.84	51.19	23.45	13.85	51.17	23.45
112	1	13.52	50.12	23.46	13.55	50.14	23.44	13.58	50.12	23.43
113	1	12.94	51.77	23.76	12.97	51.78	23.75	12.97	51.75	23.74
114	1	14.09	51.46	22.44	14.13	51.49	22.44	14.15	51.47	22.44
115	1	15.86	50.86	24.03	15.88	50.87	24.05	15.89	50.88	24.06
116	1	16.17	52.86	24.81	16.17	52.89	24.85	16.16	52.91	24.83

M121Q – OX

Atom number	Atomic number	ε = 1			ε = 5.7			ε = 78.4		
		X	Y	Z	X	Y	Z	X	Y	Z
1	29	12.42	52.09	31.60	12.33	52.07	31.66	12.32	52.03	31.69
2	16	12.19	52.12	29.44	12.38	52.03	29.44	12.39	52.01	29.46
3	6	11.13	50.72	28.86	11.17	50.75	28.86	11.17	50.75	28.86
4*	6	11.21	50.54	27.34	11.21	50.54	27.34	11.21	50.54	27.34
5	6	12.64	50.14	26.94	12.62	50.13	26.89	12.62	50.13	26.89
6	8	13.09	49.02	27.18	13.03	48.98	27.04	13.03	48.98	27.03
7	7	13.38	51.13	26.35	13.39	51.12	26.35	13.39	51.13	26.35
8*	6	14.73	50.89	25.85	14.73	50.89	25.85	14.73	50.89	25.85
9	6	15.84	51.51	26.72	15.84	51.51	26.72	15.84	51.52	26.71
10	8	16.99	51.61	26.27	16.99	51.62	26.27	16.99	51.64	26.25
11	7	15.48	52.00	27.92	15.49	52.00	27.93	15.50	51.99	27.92
12*	6	16.48	52.55	28.81	16.49	52.55	28.82	16.49	52.55	28.82
13	6	17.35	51.43	29.42	17.37	51.45	29.43	17.39	51.46	29.44

14	8	16.83	50.48	30.00	17.03	50.27	29.44	17.17	50.26	29.28
15*	6	14.76	47.95	31.60	14.76	47.95	31.60	14.76	47.95	31.60
16	6	13.83	46.98	30.85	13.87	46.93	30.87	13.88	46.93	30.87
17	6	14.20	49.37	31.44	14.14	49.34	31.43	14.13	49.33	31.44
18	8	12.81	46.55	31.40	12.92	46.39	31.44	12.93	46.38	31.45
19	7	14.15	46.72	29.56	14.15	46.73	29.55	14.15	46.73	29.55
20	6	14.74	50.40	32.38	14.79	50.43	32.21	14.80	50.44	32.18
21*	6	13.32	45.88	28.69	13.32	45.88	28.69	13.32	45.88	28.69
22	6	15.89	50.42	33.12	16.00	50.49	32.84	16.03	50.51	32.78
23	7	14.10	51.63	32.53	14.17	51.67	32.34	14.19	51.69	32.29
24	7	15.94	51.68	33.71	16.11	51.78	33.34	16.16	51.81	33.24
25	6	14.86	52.38	33.32	15.00	52.46	33.01	15.04	52.49	32.92
26	7	11.37	53.17	32.92	11.59	53.12	33.19	11.66	53.09	33.24
27	6	10.48	54.21	32.70	10.66	54.15	33.12	10.72	54.12	33.22
28	6	11.48	53.00	34.23	11.95	53.00	34.46	12.07	52.97	34.50
29	6	10.09	54.71	31.34	10.05	54.63	31.84	10.06	54.60	31.96
30	6	10.05	54.67	33.92	10.46	54.64	34.39	10.59	54.60	34.49
31	7	10.69	53.90	34.87	11.28	53.90	35.22	11.44	53.86	35.28
32	6	11.29	55.26	30.52	11.12	55.16	30.85	11.09	55.13	30.93
33	7	12.05	56.25	31.25	11.96	56.18	31.44	11.94	56.16	31.50
34	6	10.80	55.90	29.22	10.46	55.76	29.59	10.40	55.71	29.69
35	6	13.21	55.93	31.87	13.22	55.93	31.85	13.24	55.94	31.82
36	8	10.57	57.11	29.16	9.89	56.85	29.65	9.73	56.74	29.78
37	7	10.64	55.04	28.20	10.60	55.02	28.47	10.62	55.04	28.54
38*	6	13.96	57.07	32.51	13.96	57.07	32.51	13.96	57.07	32.51
39	8	13.63	54.77	31.95	13.75	54.81	31.73	13.81	54.86	31.58
40	6	10.03	55.40	26.94	10.00	55.40	27.21	10.06	55.45	27.27
41	6	9.09	54.24	26.52	9.06	54.26	26.73	9.09	54.37	26.74
42	6	11.08	55.80	25.88	11.06	55.84	26.18	11.16	55.88	26.28
43	8	8.94	53.25	27.24	8.82	53.28	27.43	8.76	53.40	27.42
44	7	8.41	54.43	25.36	8.50	54.45	25.51	8.59	54.59	25.50
45	6	12.06	54.67	25.56	12.02	54.71	25.79	12.07	54.74	25.85
46	6	7.47	53.46	24.83	7.53	53.51	24.95	7.60	53.70	24.91
47	7	13.22	55.02	24.98	13.05	55.04	24.99	13.08	55.05	25.02
48	8	11.78	53.50	25.83	11.83	53.56	26.19	11.87	53.58	26.25
49*	6	9.90	47.88	31.09	9.90	47.88	31.09	9.90	47.88	31.09
50*	6	9.41	46.46	30.92	9.41	46.46	30.92	9.41	46.46	30.92
51*	6	10.16	48.22	32.54	10.16	48.22	32.54	10.16	48.22	32.54
52	1	11.44	49.81	29.37	11.41	49.82	29.38	11.39	49.81	29.38
53	1	10.10	50.96	29.15	10.17	51.06	29.16	10.17	51.07	29.16
54*	1	10.85	51.40	26.82	10.85	51.40	26.82	10.85	51.40	26.82
55*	1	10.60	49.70	27.10	10.60	49.70	27.10	10.60	49.70	27.10
56	1	12.93	52.02	26.14	12.97	52.05	26.24	12.97	52.06	26.26
57*	1	14.85	49.83	25.80	14.85	49.83	25.80	14.85	49.83	25.80
58	1	14.54	51.86	28.26	14.55	51.85	28.28	14.55	51.84	28.28
59*	1	17.09	53.28	28.33	17.09	53.28	28.33	17.09	53.28	28.33
60*	1	14.80	47.66	32.63	14.80	47.66	32.63	14.80	47.66	32.63
61	1	13.12	49.32	31.58	13.08	49.29	31.69	13.08	49.28	31.76
62	1	14.39	49.72	30.42	14.17	49.62	30.37	14.10	49.60	30.38

63	1	14.95	47.19	29.16	14.91	47.24	29.13	14.93	47.22	29.14
64*	1	13.80	44.94	28.50	13.80	44.94	28.50	13.80	44.94	28.50
65*	1	12.41	45.72	29.23	12.41	45.72	29.23	12.41	45.72	29.23
66*	1	13.11	46.37	27.76	13.11	46.37	27.76	13.11	46.37	27.76
67	1	16.67	49.69	33.26	16.78	49.76	32.97	16.81	49.78	32.91
68	1	16.68	52.02	34.30	16.90	52.15	33.85	16.96	52.19	33.72
69	1	14.66	53.41	33.55	14.83	53.51	33.21	14.89	53.54	33.11
70	1	12.09	52.26	34.72	12.66	52.29	34.86	12.80	52.26	34.86
71	1	9.34	55.49	31.46	9.33	55.43	32.06	9.35	55.39	32.21
72	1	9.63	53.90	30.76	9.49	53.83	31.34	9.48	53.79	31.49
73	1	9.37	55.46	34.19	9.83	55.43	34.76	9.97	55.39	34.89
74	1	10.59	53.97	35.87	11.37	54.00	36.22	11.57	53.97	36.28
75	1	11.95	54.43	30.32	11.76	54.33	30.59	11.74	54.30	30.66
76	1	11.80	57.22	31.06	11.58	57.12	31.50	11.52	57.06	31.69
77	1	10.83	54.05	28.33	11.04	54.10	28.53	11.13	54.15	28.57
78	1	13.52	58.05	32.31	13.45	58.03	32.39	13.41	58.01	32.48
79	1	14.99	57.06	32.15	14.96	57.14	32.09	14.93	57.21	32.04
80	1	13.99	56.92	33.59	14.06	56.86	33.58	14.13	56.79	33.55
81	1	9.41	56.29	27.12	9.37	56.27	27.42	9.45	56.34	27.50
82	1	11.62	56.68	26.25	11.64	56.66	26.61	11.77	56.66	26.75
83	1	10.60	56.11	24.95	10.60	56.24	25.28	10.72	56.35	25.39
84	1	8.54	55.29	24.85	8.63	55.33	25.03	8.79	55.45	25.03
85	1	7.42	52.63	25.54	7.79	52.51	25.29	7.92	52.66	25.02
86	1	6.47	53.90	24.73	6.51	53.74	25.27	6.62	53.81	25.38
87	1	7.80	53.08	23.86	7.58	53.55	23.86	7.51	53.93	23.85
88	1	13.89	54.28	24.71	13.71	54.31	24.72	13.73	54.31	24.74
89	1	13.43	55.98	24.75	13.19	55.98	24.67	13.24	55.99	24.71
90*	1	10.85	47.93	30.60	10.85	47.93	30.60	10.85	47.93	30.60
91*	1	9.10	48.52	30.77	9.10	48.52	30.77	9.10	48.52	30.77
92*	1	8.88	46.16	31.80	8.88	46.16	31.80	8.88	46.16	31.80
93*	1	10.24	45.81	30.77	10.24	45.81	30.77	10.24	45.81	30.77
94*	1	8.75	46.41	30.08	8.75	46.41	30.08	8.75	46.41	30.08
95*	1	9.24	48.04	33.05	9.24	48.04	33.05	9.24	48.04	33.05
96*	1	11.04	47.66	32.81	11.04	47.66	32.81	11.04	47.66	32.81
97	6	14.93	51.42	24.40	14.92	51.43	24.41	14.91	51.42	24.40
98	8	15.14	52.84	24.39	15.02	52.86	24.39	15.01	52.85	24.37
99	6	13.78	51.10	23.47	13.80	51.03	23.46	13.79	51.00	23.46
100	1	13.58	50.02	23.45	13.68	49.94	23.44	13.68	49.92	23.45
101	1	12.86	51.61	23.78	12.85	51.47	23.77	12.84	51.44	23.77
102	1	14.03	51.42	22.45	14.03	51.37	22.45	14.02	51.34	22.45
103	1	15.85	50.94	24.03	15.87	51.01	24.05	15.86	51.00	24.04
104	1	16.02	52.98	24.77	15.88	53.08	24.79	15.86	53.07	24.77
105	7	18.70	51.56	29.32	18.53	51.87	30.00	18.43	51.92	30.17
106	6	19.45	52.44	28.43	19.11	53.21	29.95	18.75	53.31	30.47
107	1	19.46	53.48	28.79	18.41	53.96	30.30	17.95	53.82	31.02
108	1	19.03	52.41	27.42	19.44	53.48	28.93	18.97	53.89	29.56
109	8	11.45	50.27	32.38	11.12	50.36	31.94	11.07	50.37	31.91
110	7	9.30	50.43	33.06	9.86	50.27	33.82	9.95	50.24	33.87
111	6	10.38	49.71	32.67	10.43	49.70	32.74	10.44	49.70	32.75

112	1	9.35	51.44	33.01	9.97	51.26	33.98	10.09	51.22	34.06
113	1	8.41	50.00	33.24	9.28	49.74	34.45	9.43	49.70	34.54
114	1	15.96	53.03	29.65	15.96	53.06	29.63	15.95	53.06	29.62
115	1	15.78	47.90	31.20	15.78	47.94	31.20	15.78	47.95	31.20
116	1	20.48	52.10	28.39	19.98	53.23	30.60	19.65	53.33	31.10
117	1	19.20	50.77	29.72	19.11	51.12	30.36	18.99	51.20	30.61

M121Q – RED

Atom number	Atomic number	ε = 1			ε = 5.7			ε = 78.4		
		X	Y	Z	X	Y	Z	X	Y	Z
1	29	12.54	52.59	31.52	12.36	52.14	31.70	12.31	52.18	31.63
2	16	12.35	52.20	29.29	12.32	52.08	29.44	12.31	52.18	29.36
3	6	11.19	50.83	28.85	11.17	50.75	28.86	11.19	50.80	28.86
4*	6	11.21	50.54	27.34	11.21	50.54	27.34	11.21	50.54	27.34
5	6	12.63	50.14	26.91	12.63	50.14	26.91	12.62	50.14	26.90
6	8	13.08	49.01	27.14	13.07	49.00	27.11	13.06	49.00	27.10
7	7	13.37	51.11	26.30	13.38	51.12	26.33	13.37	51.12	26.32
8*	6	14.73	50.89	25.85	14.73	50.89	25.85	14.73	50.89	25.85
9	6	15.82	51.50	26.75	15.83	51.51	26.74	15.83	51.50	26.74
10	8	16.99	51.58	26.32	16.98	51.63	26.28	16.99	51.58	26.30
11	7	15.46	52.02	27.94	15.48	51.99	27.94	15.48	51.99	27.94
12*	6	16.48	52.55	28.81	16.48	52.55	28.81	16.49	52.55	28.82
13	6	17.37	51.41	29.38	17.39	51.46	29.42	17.38	51.46	29.43
14	8	16.87	50.40	29.86	17.16	50.26	29.30	17.16	50.26	29.29
15*	6	14.76	47.95	31.60	14.76	47.95	31.60	14.76	47.95	31.60
16	6	13.90	46.92	30.86	13.88	46.93	30.87	13.89	46.92	30.87
17	6	14.09	49.34	31.48	14.19	49.36	31.41	14.14	49.34	31.44
18	8	12.99	46.31	31.45	12.97	46.33	31.45	12.98	46.33	31.46
19	7	14.16	46.74	29.54	14.15	46.74	29.55	14.15	46.74	29.55
20	6	14.72	50.42	32.31	14.83	50.41	32.26	14.76	50.42	32.28
21*	6	13.32	45.88	28.69	13.32	45.88	28.69	13.32	45.88	28.69
22	6	15.82	50.35	33.12	16.02	50.42	32.94	15.95	50.44	32.96
23	7	14.22	51.72	32.34	14.21	51.65	32.42	14.13	51.65	32.42
24	7	16.00	51.62	33.64	16.12	51.67	33.52	16.04	51.70	33.52
25	6	15.01	52.40	33.15	15.02	52.37	33.18	14.93	52.39	33.17
26	7	11.28	53.31	32.87	11.31	53.10	33.09	11.48	53.30	33.06
27	6	10.34	54.33	32.72	10.41	54.16	33.04	10.51	54.29	33.01
28	6	11.28	52.98	34.15	11.51	52.84	34.37	11.77	53.14	34.35
29	6	9.99	54.89	31.37	9.94	54.74	31.74	9.97	54.80	31.72
30	6	9.81	54.62	33.94	10.07	54.53	34.32	10.23	54.72	34.29
31	7	10.41	53.76	34.84	10.78	53.68	35.15	11.03	53.97	35.12
32	6	11.24	55.30	30.55	11.14	55.14	30.85	11.12	55.23	30.78
33	7	12.06	56.28	31.24	11.97	56.14	31.48	11.97	56.22	31.43
34	6	10.82	55.87	29.20	10.64	55.71	29.52	10.57	55.80	29.47
35	6	13.21	55.93	31.85	13.25	55.90	31.87	13.31	56.08	31.57
36	8	10.56	57.08	29.09	10.20	56.87	29.48	10.01	56.91	29.46

37	7	10.74	54.97	28.20	10.70	54.89	28.46	10.74	55.02	28.38
38*	6	13.96	57.07	32.51	13.96	57.07	32.51	13.96	57.07	32.51
39	8	13.62	54.77	31.91	13.81	54.80	31.76	13.96	55.18	31.03
40	6	10.19	55.33	26.92	10.18	55.30	27.17	10.22	55.43	27.08
41	6	9.27	54.18	26.43	9.23	54.22	26.60	9.24	54.38	26.53
42	6	11.29	55.77	25.93	11.31	55.76	26.21	11.36	55.84	26.12
43	8	8.97	53.25	27.17	8.88	53.25	27.27	8.88	53.40	27.20
44	7	8.73	54.35	25.19	8.70	54.49	25.38	8.71	54.65	25.31
45	6	12.23	54.63	25.53	12.22	54.63	25.74	12.26	54.68	25.70
46	6	7.93	53.30	24.57	7.83	53.54	24.71	7.76	53.75	24.68
47	7	13.50	54.96	25.22	13.48	54.97	25.41	13.52	55.00	25.35
48	8	11.81	53.47	25.47	11.79	53.47	25.63	11.84	53.52	25.67
49*	6	9.90	47.88	31.09	9.90	47.88	31.09	9.90	47.88	31.09
50*	6	9.41	46.46	30.92	9.41	46.46	30.92	9.41	46.46	30.92
51*	6	10.16	48.22	32.54	10.16	48.22	32.54	10.16	48.22	32.54
52	1	11.46	49.93	29.40	11.43	49.82	29.36	11.49	49.89	29.39
53	1	10.17	51.13	29.12	10.14	51.01	29.16	10.17	51.03	29.16
54*	1	10.85	51.40	26.82	10.85	51.40	26.82	10.85	51.40	26.82
55*	1	10.60	49.70	27.10	10.60	49.70	27.10	10.60	49.70	27.10
56	1	12.92	52.01	26.11	12.93	52.02	26.14	12.93	52.03	26.14
57*	1	14.85	49.83	25.80	14.85	49.83	25.80	14.85	49.83	25.80
58	1	14.50	51.91	28.30	14.53	51.87	28.31	14.52	51.91	28.28
59*	1	17.09	53.28	28.33	17.09	53.28	28.33	17.09	53.28	28.33
60*	1	14.80	47.66	32.63	14.80	47.66	32.63	14.80	47.66	32.63
61	1	13.04	49.25	31.78	13.13	49.35	31.64	13.07	49.29	31.68
62	1	14.10	49.67	30.43	14.28	49.65	30.35	14.18	49.65	30.39
63	1	14.78	47.39	29.08	14.84	47.32	29.11	14.88	47.28	29.12
64*	1	13.80	44.94	28.50	13.80	44.94	28.50	13.80	44.94	28.50
65*	1	12.41	45.72	29.23	12.41	45.72	29.23	12.41	45.72	29.23
66*	1	13.11	46.37	27.76	13.11	46.37	27.76	13.11	46.37	27.76
67	1	16.50	49.54	33.34	16.78	49.66	33.06	16.72	49.70	33.10
68	1	16.73	51.91	34.27	16.88	52.00	34.09	16.80	52.05	34.09
69	1	14.90	53.46	33.36	14.85	53.40	33.45	14.75	53.41	33.45
70	1	11.83	52.15	34.57	12.14	52.06	34.76	12.50	52.45	34.75
71	1	9.33	55.76	31.50	9.30	55.61	31.93	9.29	55.64	31.90
72	1	9.45	54.14	30.79	9.35	54.00	31.19	9.39	54.02	31.20
73	1	9.06	55.34	34.25	9.41	55.29	34.70	9.55	55.46	34.66
74	1	10.21	53.68	35.82	10.75	53.68	36.16	11.07	54.03	36.13
75	1	11.84	54.39	30.44	11.73	54.23	30.73	11.73	54.34	30.63
76	1	11.80	57.25	31.09	11.61	57.08	31.49	11.51	57.02	31.86
77	1	11.04	54.00	28.38	11.09	53.94	28.59	11.16	54.09	28.50
78	1	13.56	58.06	32.25	13.41	58.01	32.43	13.38	57.99	32.63
79	1	15.01	57.02	32.22	14.94	57.19	32.05	14.95	57.32	32.14
80	1	13.90	56.95	33.59	14.12	56.85	33.57	14.07	56.60	33.49
81	1	9.55	56.21	27.08	9.57	56.19	27.37	9.64	56.34	27.28
82	1	11.84	56.60	26.39	11.89	56.54	26.72	11.95	56.62	26.60
83	1	10.85	56.18	25.01	10.89	56.24	25.32	10.95	56.29	25.21
84	1	9.15	55.03	24.57	9.09	55.24	24.83	9.04	55.45	24.79
85	1	7.41	52.76	25.36	7.13	53.12	25.43	6.98	53.47	25.39

86	1	7.20	53.74	23.89	7.27	54.06	23.93	7.30	54.27	23.83
87	1	8.56	52.58	24.02	8.40	52.72	24.25	8.24	52.84	24.31
88	1	14.15	54.23	24.92	14.11	54.25	25.05	14.14	54.26	25.01
89	1	13.83	55.91	25.33	13.82	55.91	25.54	13.85	55.95	25.39
90*	1	10.85	47.93	30.60	10.85	47.93	30.60	10.85	47.93	30.60
91*	1	9.10	48.52	30.77	9.10	48.52	30.77	9.10	48.52	30.77
92*	1	8.88	46.16	31.80	8.88	46.16	31.80	8.88	46.16	31.80
93*	1	10.24	45.81	30.77	10.24	45.81	30.77	10.24	45.81	30.77
94*	1	8.75	46.41	30.08	8.75	46.41	30.08	8.75	46.41	30.08
95*	1	9.24	48.04	33.05	9.24	48.04	33.05	9.24	48.04	33.05
96*	1	11.04	47.66	32.81	11.04	47.66	32.81	11.04	47.66	32.81
97	6	15.00	51.41	24.40	14.96	51.41	24.40	14.98	51.40	24.40
98	8	15.33	52.81	24.39	15.26	52.82	24.38	15.27	52.81	24.37
99	6	13.83	51.19	23.45	13.79	51.16	23.47	13.81	51.15	23.46
100	1	13.55	50.14	23.44	13.52	50.10	23.46	13.54	50.09	23.46
101	1	12.96	51.78	23.75	12.92	51.74	23.78	12.93	51.73	23.76
102	1	14.13	51.49	22.44	14.06	51.46	22.45	14.08	51.44	22.44
103	1	15.88	50.86	24.05	15.85	50.88	24.03	15.86	50.87	24.04
104	1	16.18	52.86	24.85	16.12	52.90	24.82	16.13	52.90	24.81
105	7	18.72	51.61	29.36	18.47	51.92	30.12	18.44	51.92	30.15
106	6	19.45	52.52	28.48	18.87	53.30	30.32	18.79	53.31	30.42
107	1	19.40	53.56	28.83	18.05	53.91	30.72	17.97	53.84	30.93
108	1	19.07	52.47	27.45	19.23	53.77	29.39	19.05	53.85	29.51
109	8	11.27	49.94	33.80	11.58	50.19	32.76	11.15	50.36	31.97
110	7	10.13	50.61	31.94	9.34	50.50	32.76	9.97	50.23	33.90
111	6	10.58	49.66	32.82	10.44	49.70	32.70	10.47	49.69	32.77
112	1	10.72	51.44	31.87	9.49	51.50	32.67	10.18	51.19	34.12
113	1	9.78	50.29	31.05	8.42	50.14	32.57	9.45	49.68	34.56
114	1	15.98	53.01	29.67	15.96	53.05	29.64	15.96	53.06	29.63
115	1	15.78	47.99	31.19	15.79	47.90	31.21	15.79	47.94	31.20
116	1	20.50	52.23	28.48	19.69	53.32	31.04	19.66	53.32	31.08
117	1	19.22	50.80	29.70	19.06	51.19	30.49	19.00	51.19	30.58

M121N – OX

Atom number	Atomic number	ε = 1			ε = 5.7			ε = 78.4		
		X	Y	Z	X	Y	Z	X	Y	Z
1	29	12.7	52.4	31.4	12.7	52.4	31.4	12.69	52.46	31.41
2	16	12.3	52.2	29.3	12.3	52.2	29.3	12.28	52.24	29.28
3	6	11.2	50.8	28.9	11.2	50.8	28.9	11.21	50.79	28.86
4*	6	11.2	50.5	27.3	11.2	50.5	27.3	11.21	50.54	27.34
5	6	12.6	50.2	26.9	12.6	50.1	26.9	12.62	50.14	26.88
6	8	13.1	49	27.2	13.1	49	27.1	13.05	49.00	27.06
7	7	13.4	51.1	26.3	13.4	51.1	26.3	13.37	51.13	26.32
8*	6	14.7	50.9	25.9	14.7	50.9	25.9	14.73	50.89	25.85
9	6	15.8	51.5	26.7	15.8	51.5	26.8	15.83	51.49	26.75
10	8	17	51.5	26.3	17	51.5	26.4	17.00	51.50	26.35

11	7	15.5	52	27.9	15.5	52	27.9	15.46	52.04	27.93
12*	6	16.5	52.6	28.8	16.5	52.5	28.8	16.49	52.55	28.81
13	6	17.3	51.4	29.5	17.3	51.4	29.4	17.38	51.41	29.34
14	8	16.8	50.5	30.1	16.8	50.4	30	16.95	50.27	29.51
15	7	14.8	47.9	31.6	14.8	47.9	31.6	18.64	51.79	29.67
16	6	13.9	47	30.8	13.9	46.9	30.9	19.62	50.88	30.25
17*	6	14.1	49.3	31.5	14.1	49.3	31.5	14.76	47.95	31.60
18	6	12.9	46.5	31.4	12.9	46.4	31.4	13.88	46.94	30.85
19	6	14.2	46.7	29.5	14.2	46.7	29.5	14.09	49.34	31.50
20	8	14.6	50.4	32.4	14.6	50.4	32.4	12.94	46.39	31.43
21	7	13.3	45.9	28.7	13.3	45.9	28.7	14.15	46.74	29.54
22	6	15.7	50.3	33.3	15.7	50.3	33.3	14.69	50.40	32.39
23*	6	14.1	51.7	32.5	14.1	51.7	32.5	13.32	45.88	28.69
24	6	15.8	51.6	33.8	15.8	51.6	33.9	15.77	50.34	33.23
25	7	14.9	52.4	33.3	14.8	52.4	33.4	14.18	51.69	32.44
26	7	11.3	53	32.7	11.3	53	32.8	15.91	51.60	33.78
27	6	10.3	54	32.5	10.4	54	32.6	14.94	52.39	33.28
28	7	11.2	52.6	34	11.3	52.6	34	11.41	52.99	32.79
29	6	10	54.7	31.2	10	54.7	31.3	10.40	53.95	32.71
30	6	9.67	54.2	33.7	9.75	54.1	33.8	11.43	52.54	34.04
31	6	10.2	53.3	34.6	10.4	53.3	34.7	9.99	54.65	31.45
32	6	11.3	55.3	30.5	11.2	55.3	30.6	9.83	54.07	33.95
33	7	12	56.2	31.3	12	56.2	31.4	10.49	53.17	34.77
34	6	10.8	56	29.2	10.7	56	29.3	11.16	55.28	30.66
35	7	13.2	55.9	31.9	13.2	55.9	31.9	11.95	56.23	31.42
36	6	10.5	57.2	29.2	10.4	57.2	29.3	10.65	55.98	29.39
37	6	10.8	55.2	28.1	10.8	55.2	28.2	13.17	55.92	31.90
38	8	14	57.1	32.5	14	57.1	32.5	10.21	57.13	29.45
39	7	13.6	54.7	31.9	13.6	54.7	31.9	10.71	55.23	28.27
40*	6	10.2	55.6	26.9	10.2	55.6	26.9	13.96	57.07	32.51
41	8	9.2	54.5	26.5	9.2	54.5	26.5	13.63	54.76	31.84
42	6	11.3	55.9	25.8	11.3	56	25.9	10.18	55.66	26.99
43	6	9.03	53.5	27.3	8.95	53.6	27.1	9.16	54.61	26.49
44	6	8.5	54.6	25.4	8.61	54.8	25.3	11.32	55.98	26.00
45	8	12.2	54.7	25.5	12.3	54.8	25.6	8.82	53.65	27.18
46	7	7.49	53.7	24.9	7.6	53.9	24.7	8.63	54.86	25.27
47	6	13.4	55	24.9	13.4	55.1	25.1	12.21	54.79	25.68
48	6	11.9	53.6	25.8	11.9	53.6	25.9	7.60	54.00	24.70
49	7	9.9	47.9	31.1	9.9	47.9	31.1	13.37	55.06	25.06
50	8	9.41	46.5	30.9	9.41	46.5	30.9	11.86	53.63	25.97
51	1	11.5	49.9	29.4	11.6	49.9	29.4	11.58	49.91	29.39
52	1	10.2	51	29.2	10.2	51	29.2	10.20	51.01	29.20
53*	1	10.9	51.4	26.8	10.9	51.4	26.8	10.85	51.40	26.82
54*	1	10.6	49.7	27.1	10.6	49.7	27.1	10.60	49.70	27.10
55	1	12.9	52	26.1	12.9	52	26.1	12.95	52.05	26.17
56*	1	14.8	49.8	25.8	14.8	49.8	25.8	14.85	49.83	25.80
57	1	14.5	51.9	28.3	14.5	52	28.2	14.50	51.97	28.25
58*	1	17.1	53.3	28.3	17.1	53.3	28.3	17.09	53.28	28.31
59*	1	14.8	47.6	32.6	14.8	47.6	32.6	16.04	52.98	29.69

60	1	13	49.2	31.7	13	49.2	31.7	18.94	52.73	29.44
61	1	14.2	49.7	30.5	14.2	49.7	30.5	20.34	50.53	29.50
62	1	14.9	47.2	29.1	14.9	47.3	29.1	20.17	51.39	31.05
63*	1	13.8	44.9	28.5	13.8	44.9	28.5	14.78	47.62	32.62
64	1	12.4	45.7	29.2	12.4	45.7	29.2	13.03	49.23	31.75
65	1	13.1	46.4	27.8	13.1	46.4	27.8	14.14	49.69	30.46
66	1	16.4	49.5	33.5	16.4	49.5	33.6	14.92	47.24	29.12
67*	1	16.6	51.9	34.5	16.5	51.8	34.6	13.80	44.94	28.50
68*	1	14.7	53.4	33.6	14.7	53.4	33.6	12.41	45.72	29.23
69*	1	11.9	51.9	34.4	12	51.9	34.5	13.11	46.37	27.76
70	1	9.29	55.5	31.4	9.27	55.4	31.5	16.44	49.53	33.48
71	1	9.6	54	30.5	9.54	54	30.7	16.61	51.89	34.44
72	1	8.85	54.8	34	8.95	54.8	34.2	14.81	53.43	33.52
73	1	9.98	53.2	35.6	10.1	53.1	35.7	12.10	51.80	34.44
74	1	12	54.5	30.3	11.9	54.5	30.3	9.26	55.43	31.71
75	1	11.7	57.2	31.2	11.7	57.2	31.3	9.49	53.95	30.78
76	1	11.1	54.2	28.2	11	54.2	28.3	9.03	54.69	34.31
77	1	13.4	58	32.5	13.4	58	32.6	10.30	53.01	35.75
78	1	14.9	57.2	32	14.8	57.3	31.8	11.84	54.48	30.38
79	1	14.2	56.8	33.5	14.4	56.8	33.5	11.63	57.19	31.42
80	1	9.64	56.5	27.1	9.65	56.5	27.1	11.06	54.27	28.33
81	1	11.8	56.8	26.1	11.9	56.8	26.3	13.40	58.01	32.51
82	1	10.8	56.2	24.8	10.9	56.3	25	14.88	57.20	31.93
83	1	8.66	55.5	24.8	8.79	55.7	24.8	14.24	56.81	33.53
84	1	7.45	52.9	25.6	7.68	52.9	25.2	9.64	56.59	27.18
85	1	6.51	54.2	24.8	6.59	54.3	24.8	11.93	56.79	26.42
86	1	7.76	53.3	23.9	7.77	53.8	23.6	10.91	56.37	25.06
87	1	14	54.3	24.7	14.1	54.3	24.8	8.84	55.72	24.80
88	1	13.6	56	24.6	13.7	56	24.8	7.88	52.96	24.81
89	1	10.9	47.9	30.6	10.9	47.9	30.6	6.63	54.15	25.19
90	1	8.88	46.2	31.8	8.88	46.2	31.8	7.50	54.24	23.64
91	1	10.2	45.8	30.8	10.2	45.8	30.8	13.99	54.29	24.80
92	1	8.75	46.4	30.1	8.75	46.4	30.1	13.63	56.01	24.83
93	1	15	51.4	24.4	15	51.4	24.4	19.09	50.02	30.66
94*	1	15.3	52.8	24.4	15.3	52.8	24.4	15.76	47.98	31.20
95	6	13.9	51.2	23.4	13.9	51.2	23.4	14.98	51.41	24.41
96	8	13.7	50.1	23.4	13.7	50.1	23.4	15.19	52.83	24.38
97	6	13	51.7	23.7	13	51.7	23.7	13.84	51.09	23.45
98	1	14.2	51.5	22.4	14.2	51.5	22.4	13.64	50.01	23.44
99	1	15.9	50.9	24.1	15.9	50.9	24.1	12.92	51.61	23.74
100	1	16.1	53	24.8	16.2	53	24.8	14.11	51.40	22.44
101	1	18.7	51.5	29.4	18.7	51.6	29.4	15.90	50.92	24.06
102	1	19.4	52.3	28.4	19.5	52.5	28.7	16.05	52.99	24.79
103*	6	19.5	53.3	28.8	19.4	53.5	29	9.90	47.88	31.09
104*	6	19	52.3	27.4	19.2	52.5	27.6	9.41	46.46	30.92
105*	1	8.95	49	30.7	8.95	49	30.7	10.85	47.93	30.60
106*	1	8.63	49.9	31.7	8.89	50	31.6	8.88	46.16	31.80
107*	1	8.52	49.1	29.6	8.31	49	29.7	10.24	45.81	30.77
108*	1	10.3	47.9	32.1	10.3	47.9	32.1	8.75	46.41	30.08

109	6	8.73	49.6	32.7	9.26	49.9	32.5	8.95	49.00	30.73
110	7	7.85	50.5	31.5	8.19	50.7	31.4	8.94	50.06	31.57
111	8	15.8	48	31.2	15.8	48	31.2	8.25	48.96	29.71
112*	1	20.5	51.9	28.4	20.5	52.2	28.7	10.29	47.91	32.09
113	1	16	53.1	29.6	16	53.1	29.6	9.45	50.05	32.45
114	1	19.1	50.7	29.8	19.2	50.8	29.9	8.31	50.83	31.39

M121N – RED

Atom number	Atomic number	ε = 1			ε = 5.7			ε = 78.4		
		X	Y	Z	X	Y	Z	X	Y	Z
1	29	12.6	52.5	31.5	12.6	52.5	31.5	12.60	52.48	31.55
2	16	12.5	52.1	29.3	12.4	52.2	29.3	12.41	52.16	29.32
3	6	11.2	50.8	28.9	11.2	50.8	28.9	11.22	50.81	28.86
4*	6	11.2	50.5	27.3	11.2	50.5	27.3	11.21	50.54	27.34
5	6	12.6	50.1	26.9	12.6	50.1	26.9	12.62	50.14	26.88
6	8	13.1	49	27.2	13.1	49	27.1	13.05	49.00	27.08
7	7	13.4	51.1	26.3	13.4	51.1	26.3	13.37	51.12	26.30
8*	6	14.7	50.9	25.9	14.7	50.9	25.9	14.73	50.89	25.85
9	6	15.8	51.5	26.7	15.8	51.5	26.8	15.82	51.50	26.76
10	8	17	51.6	26.3	17	51.6	26.3	16.99	51.54	26.34
11	7	15.5	51.9	28	15.5	52	27.9	15.45	52.02	27.94
12*	6	16.5	52.6	28.8	16.5	52.5	28.8	16.48	52.55	28.81
13	6	17.5	51.6	29.5	17.4	51.4	29.3	17.41	51.42	29.33
14	8	17.8	51.7	30.7	17	50.3	29.5	17.06	50.25	29.40
15	7	18.2	50.7	28.6	18.7	51.8	29.7	18.62	51.85	29.77
16	6	19.2	49.9	29.1	19.7	50.9	30.2	19.59	50.94	30.37
17*	6	14.8	47.9	31.6	14.8	47.9	31.6	14.76	47.95	31.60
18	6	13.9	46.9	30.9	13.9	46.9	30.9	13.89	46.93	30.86
19	6	14.1	49.3	31.5	14.1	49.3	31.5	14.10	49.34	31.51
20	8	13.1	46.2	31.5	13.1	46.2	31.5	12.96	46.35	31.44
21	7	14.2	46.8	29.5	14.1	46.8	29.5	14.15	46.74	29.54
22	6	14.8	50.4	32.2	14.8	50.4	32.3	14.77	50.40	32.34
23*	6	13.3	45.9	28.7	13.3	45.9	28.7	13.32	45.88	28.69
24	6	16	50.4	32.9	15.9	50.3	33.1	15.88	50.31	33.15
25	7	14.3	51.7	32.3	14.3	51.7	32.4	14.29	51.71	32.37
26	7	16.3	51.6	33.3	16.1	51.6	33.7	16.07	51.57	33.67
27	6	15.3	52.4	32.9	15.1	52.4	33.2	15.10	52.38	33.18
28	7	11.2	52.9	32.8	11.2	52.9	32.9	11.27	52.92	32.92
29	6	10.3	53.8	32.7	10.3	53.9	32.8	10.29	53.90	32.91
30	6	11.2	52.4	34.1	11.2	52.5	34.1	11.32	52.46	34.17
31	6	9.96	54.6	31.5	9.91	54.6	31.6	9.90	54.63	31.66
32	6	9.63	54	34	9.67	54	34.1	9.76	54.02	34.17
33	7	10.2	53	34.8	10.3	53.1	34.9	10.42	53.09	34.95
34	6	11.2	55.1	30.8	11.2	55.2	30.8	11.11	55.16	30.86
35	7	11.9	56.1	31.5	11.9	56.1	31.6	11.90	56.14	31.58
36	6	10.8	55.8	29.4	10.7	55.8	29.5	10.65	55.80	29.55

37	6	13.2	55.9	32	13.2	55.9	32	13.17	55.88	31.99
38	8	10.5	57	29.4	10.4	57	29.5	10.20	56.95	29.55
39	7	10.8	54.9	28.4	10.8	55	28.4	10.76	55.03	28.45
40*	6	14	57.1	32.5	14	57.1	32.5	13.96	57.07	32.51
41	8	13.6	54.7	32.1	13.6	54.7	32	13.66	54.74	31.94
42	6	10.2	55.3	27.1	10.3	55.5	27.1	10.27	55.48	27.16
43	6	9.28	54.2	26.7	9.3	54.4	26.5	9.28	54.45	26.58
44	6	11.3	55.8	26.1	11.4	55.9	26.2	11.43	55.88	26.22
45	8	8.96	53.3	27.4	8.96	53.4	27.2	8.91	53.46	27.21
46	7	8.72	54.3	25.4	8.77	54.8	25.3	8.77	54.75	25.36
47	6	12.2	54.6	25.6	12.3	54.7	25.7	12.31	54.71	25.78
48	6	7.88	53.3	24.9	7.8	53.9	24.6	7.78	53.90	24.72
49	7	13.5	55	25.3	13.6	55	25.4	13.57	55.02	25.44
50	8	11.8	53.5	25.6	11.9	53.5	25.7	11.87	53.56	25.74
51	1	11.5	49.9	29.4	11.5	49.9	29.4	11.50	49.89	29.38
52	1	10.2	51.2	29.1	10.2	51.1	29.2	10.22	51.08	29.18
53*	1	10.9	51.4	26.8	10.9	51.4	26.8	10.85	51.40	26.82
54*	1	10.6	49.7	27.1	10.6	49.7	27.1	10.60	49.70	27.10
55	1	12.9	52	26.1	12.9	52	26.1	12.94	52.03	26.15
56*	1	14.8	49.8	25.8	14.8	49.8	25.8	14.85	49.83	25.80
57	1	14.5	51.8	28.3	14.5	51.9	28.3	14.49	51.94	28.30
58*	1	17.1	53.3	28.3	17.1	53.3	28.3	17.09	53.28	28.31
59*	1	16	53	29.7	16	53	29.7	16.04	52.98	29.69
60	1	18	50.8	27.6	18.9	52.8	29.4	18.88	52.82	29.66
61	1	19.2	48.9	28.6	20.4	50.6	29.4	19.96	50.22	29.64
62	1	20.2	50.3	28.9	20.2	51.4	31	20.42	51.52	30.76
63*	1	14.8	47.6	32.6	14.8	47.6	32.6	14.78	47.62	32.62
64	1	13.1	49.2	31.9	13	49.2	31.8	13.05	49.25	31.83
65	1	13.9	49.6	30.5	14.1	49.7	30.4	14.07	49.68	30.47
66	1	14.7	47.5	29	14.8	47.4	29.1	14.89	47.28	29.11
67*	1	13.8	44.9	28.5	13.8	44.9	28.5	13.80	44.94	28.50
68*	1	12.4	45.7	29.2	12.4	45.7	29.2	12.41	45.72	29.23
69*	1	13.1	46.4	27.8	13.1	46.4	27.8	13.11	46.37	27.76
70	1	16.7	49.5	33	16.5	49.5	33.3	16.53	49.49	33.39
71	1	17.2	52	33.7	16.8	51.8	34.3	16.81	51.85	34.30
72	1	15.2	53.5	33.1	15	53.4	33.4	15.01	53.43	33.40
73	1	11.8	51.7	34.5	11.9	51.7	34.5	11.98	51.69	34.53
74	1	9.25	55.4	31.7	9.23	55.5	31.8	9.23	55.46	31.92
75	1	9.48	53.9	30.7	9.39	54	30.9	9.35	53.96	31.00
76	1	8.82	54.6	34.3	8.88	54.7	34.4	8.99	54.66	34.57
77	1	9.99	52.9	35.7	10.1	52.9	35.8	10.26	52.93	35.93
78	1	11.9	54.3	30.6	11.8	54.3	30.6	11.76	54.30	30.69
79	1	11.7	57.1	31.3	11.6	57.1	31.5	11.57	57.10	31.55
80	1	11.2	54	28.5	11.1	54.1	28.5	11.16	54.08	28.55
81	1	13.4	58	32.5	13.4	58	32.6	13.35	57.97	32.63
82	1	14.8	57.2	31.8	14.8	57.3	31.8	14.77	57.29	31.80
83	1	14.4	56.9	33.5	14.4	56.8	33.5	14.42	56.81	33.46
84	1	9.62	56.2	27.3	9.73	56.4	27.3	9.71	56.39	27.36
85	1	11.9	56.6	26.5	12	56.6	26.7	12.03	56.65	26.71

86	1	10.8	56.2	25.2	11	56.3	25.2	11.03	56.35	25.31
87	1	9.17	55	24.8	9.07	55.6	24.9	9.05	55.60	24.89
88	1	7.36	52.8	25.7	7.65	53	25.2	8.17	52.89	24.58
89	1	7.14	53.7	24.2	6.84	54.4	24.5	6.86	53.83	25.31
90	1	8.46	52.5	24.3	8.16	53.6	23.7	7.54	54.32	23.74
91	1	14.1	54.2	24.9	14.2	54.3	25	14.18	54.28	25.07
92	1	13.8	55.9	25.4	13.9	56	25.4	13.92	55.96	25.48
93	1	19.1	49.7	30.2	19.2	50.1	30.6	19.13	50.38	31.19
94*	1	15.8	48	31.2	15.8	48	31.2	15.76	47.98	31.20
95	6	15	51.4	24.4	15	51.4	24.4	15.00	51.41	24.41
96	8	15.3	52.8	24.4	15.3	52.8	24.4	15.29	52.82	24.39
97	6	13.8	51.2	23.5	13.8	51.2	23.5	13.84	51.17	23.45
98	1	13.5	50.1	23.5	13.5	50.1	23.4	13.57	50.11	23.44
99	1	12.9	51.7	23.8	13	51.8	23.8	12.96	51.75	23.74
100	1	14.1	51.4	22.4	14.1	51.5	22.4	14.13	51.47	22.44
101	1	15.9	50.9	24	15.9	50.9	24	15.89	50.88	24.05
102	1	16.1	52.9	24.8	16.2	52.9	24.8	16.15	52.91	24.83
103*	6	9.9	47.9	31.1	9.9	47.9	31.1	9.90	47.88	31.09
104*	6	9.41	46.5	30.9	9.41	46.5	30.9	9.41	46.46	30.92
105*	1	10.9	47.9	30.6	10.9	47.9	30.6	10.85	47.93	30.60
106*	1	8.88	46.2	31.8	8.88	46.2	31.8	8.88	46.16	31.80
107*	1	10.2	45.8	30.8	10.2	45.8	30.8	10.24	45.81	30.77
108*	1	8.75	46.4	30.1	8.75	46.4	30.1	8.75	46.41	30.08
109	6	8.91	49	30.8	8.93	49	30.8	8.94	49.00	30.75
110	7	9.1	50.2	31.5	9.08	50.1	31.5	8.99	50.09	31.56
111	8	7.96	48.8	30	8.03	48.9	29.9	8.15	48.92	29.80
112*	1	10.3	47.9	32.1	10.3	47.9	32.1	10.29	47.91	32.09
113	1	10	50.4	31.8	9.93	50.3	32	9.71	50.19	32.26
114	1	8.56	50.9	31.1	8.55	50.9	31.2	8.45	50.90	31.31

M121L/F114N (including Pro115, Gly116) – OX

Atom number	Atomic number	ε = 1			ε = 5.7			ε = 78.4		
		X	Y	Z	X	Y	Z	X	Y	Z
1	29	12.74	52.44	31.49	12.76	52.50	31.47	12.77	52.54	31.45
2	16	12.29	52.14	29.38	12.31	52.16	29.35	12.31	52.19	29.33
3	6	11.17	50.75	28.86	11.19	50.76	28.86	11.20	50.77	28.86
4*	6	11.21	50.54	27.34	11.21	50.54	27.34	11.21	50.54	27.34
5	6	12.63	50.14	26.91	12.62	50.14	26.89	12.62	50.14	26.88
6	8	13.06	49.00	27.10	13.05	48.99	27.06	13.05	49.00	27.07
7	7	13.39	51.13	26.36	13.38	51.13	26.35	13.38	51.13	26.33
8*	6	14.73	50.89	25.85	14.73	50.89	25.85	14.73	50.89	25.85
9	6	15.85	51.52	26.70	15.85	51.49	26.72	15.84	51.48	26.73
10	8	17.00	51.62	26.27	17.01	51.51	26.31	17.01	51.47	26.34
11	7	15.48	52.04	27.90	15.48	52.04	27.90	15.47	52.05	27.90
12*	6	16.49	52.55	28.81	16.48	52.55	28.82	16.48	52.55	28.82

13	6	17.35	51.36	29.32	17.34	51.36	29.31	17.33	51.34	29.28
14	8	16.80	50.27	29.50	16.79	50.26	29.49	16.75	50.29	29.58
15*	6	14.76	47.95	31.60	14.76	47.95	31.60	14.76	47.95	31.60
16	6	13.80	47.01	30.83	13.83	46.98	30.84	13.84	46.97	30.84
17	6	14.23	49.39	31.43	14.22	49.38	31.43	14.21	49.38	31.43
18	8	12.75	46.67	31.36	12.81	46.56	31.40	12.84	46.52	31.41
19	7	14.16	46.71	29.56	14.15	46.72	29.55	14.15	46.73	29.55
20	6	14.84	50.43	32.33	14.80	50.41	32.36	14.76	50.40	32.39
21*	6	13.32	45.88	28.69	13.32	45.88	28.69	13.32	45.88	28.69
22	6	16.01	50.40	33.05	15.89	50.34	33.19	15.79	50.30	33.30
23	7	14.29	51.71	32.47	14.28	51.71	32.47	14.27	51.71	32.46
24	7	16.15	51.64	33.62	16.01	51.57	33.80	15.90	51.53	33.91
25	6	15.11	52.41	33.24	15.04	52.37	33.33	14.98	52.36	33.38
26	7	11.64	53.42	32.83	11.65	53.44	32.82	11.65	53.45	32.82
27	6	10.62	54.32	32.55	10.64	54.35	32.54	10.63	54.36	32.55
28	6	11.77	53.38	34.14	11.80	53.42	34.14	11.80	53.43	34.14
29	6	10.21	54.71	31.16	10.21	54.73	31.16	10.20	54.75	31.17
30	6	10.14	54.81	33.74	10.18	54.86	33.73	10.17	54.86	33.74
31	7	10.88	54.20	34.73	10.92	54.25	34.72	10.92	54.26	34.73
32	6	11.37	55.34	30.34	11.37	55.36	30.32	11.36	55.37	30.33
33	7	12.13	56.31	31.09	12.15	56.32	31.07	12.14	56.33	31.08
34	6	10.84	56.01	29.07	10.84	56.01	29.05	10.81	56.03	29.06
35	6	13.28	55.97	31.72	13.29	55.98	31.71	13.28	55.98	31.70
36	8	10.52	57.20	29.07	10.60	57.22	28.99	10.52	57.23	29.04
37	7	10.75	55.18	28.01	10.66	55.14	28.03	10.68	55.18	28.02
38*	6	13.96	57.07	32.51	13.96	57.07	32.51	13.96	57.07	32.51
39	8	13.74	54.82	31.70	13.74	54.82	31.67	13.74	54.82	31.66
40	6	10.07	55.52	26.78	10.05	55.50	26.76	10.09	55.55	26.75
41	6	9.07	54.37	26.46	9.08	54.35	26.36	9.09	54.45	26.33
42	6	11.07	55.82	25.64	11.12	55.84	25.70	11.17	55.87	25.70
43	8	8.99	53.38	27.19	8.94	53.36	27.07	8.89	53.46	27.03
44	7	8.31	54.56	25.36	8.41	54.55	25.20	8.45	54.66	25.16
45	6	12.06	54.68	25.39	12.11	54.70	25.46	12.14	54.72	25.46
46	6	7.28	53.62	24.94	7.43	53.59	24.71	7.43	53.74	24.67
47	7	13.14	54.97	24.63	13.28	55.03	24.89	13.32	55.02	24.90
48	8	11.87	53.57	25.89	11.83	53.54	25.78	11.83	53.55	25.78
49*	6	9.90	47.88	31.09	9.90	47.88	31.09	9.90	47.88	31.09
50*	6	9.41	46.46	30.92	9.41	46.46	30.92	9.41	46.46	30.92
51*	6	10.16	48.22	32.54	10.16	48.22	32.54	10.16	48.22	32.54
52	6	9.50	50.73	32.31	9.48	50.72	32.31	9.44	50.71	32.33
53	1	11.46	49.84	29.39	11.50	49.86	29.39	11.53	49.88	29.39
54	1	10.16	51.03	29.17	10.18	51.02	29.19	10.19	51.02	29.20
55*	1	10.85	51.40	26.82	10.85	51.40	26.82	10.85	51.40	26.82
56*	1	10.60	49.70	27.10	10.60	49.70	27.10	10.60	49.70	27.10
57	1	12.95	52.04	26.17	12.94	52.04	26.15	12.94	52.04	26.14
58*	1	14.85	49.83	25.80	14.85	49.83	25.80	14.85	49.83	25.80
59	1	14.55	51.85	28.25	14.53	51.91	28.23	14.51	51.97	28.22
60*	1	17.09	53.28	28.31	17.09	53.28	28.31	17.09	53.28	28.31
61	1	19.58	49.77	28.79	19.54	49.76	28.74	19.43	49.62	28.56

62*	1	14.78	47.62	32.62	14.78	47.62	32.62	14.78	47.62	32.62
63	1	13.15	49.35	31.60	13.14	49.34	31.56	13.13	49.33	31.54
64	1	14.37	49.69	30.39	14.40	49.71	30.40	14.41	49.72	30.41
65	1	15.06	47.02	29.24	15.02	47.09	29.20	15.02	47.10	29.19
66*	1	13.80	44.94	28.50	13.80	44.94	28.50	13.80	44.94	28.50
67*	1	12.41	45.72	29.23	12.41	45.72	29.23	12.41	45.72	29.23
68*	1	13.11	46.37	27.76	13.11	46.37	27.76	13.11	46.37	27.76
69	1	16.77	49.65	33.15	16.60	49.55	33.37	16.45	49.48	33.54
70	1	16.98	51.99	34.07	16.76	51.86	34.40	16.59	51.79	34.60
71	1	15.01	53.46	33.46	14.93	53.41	33.58	14.88	53.40	33.63
72	1	12.48	52.77	34.68	12.51	52.82	34.69	12.52	52.84	34.69
73	1	9.39	55.44	31.24	9.39	55.46	31.24	9.38	55.48	31.26
74	1	9.83	53.85	30.61	9.83	53.87	30.61	9.81	53.89	30.62
75	1	9.36	55.52	33.95	9.41	55.58	33.95	9.40	55.58	33.96
76	1	10.76	54.33	35.72	10.82	54.40	35.72	10.83	54.40	35.72
77	1	12.05	54.53	30.08	12.05	54.55	30.06	12.03	54.57	30.06
78	1	11.82	57.27	31.03	11.83	57.28	31.04	11.82	57.29	31.08
79	1	10.99	54.20	28.11	10.85	54.16	28.16	10.94	54.21	28.13
80	1	13.53	58.06	32.32	13.51	58.05	32.35	13.61	58.07	32.24
81	1	15.02	57.08	32.26	15.02	57.10	32.25	15.04	57.01	32.36
82	1	13.87	56.85	33.58	13.89	56.82	33.57	13.76	56.90	33.57
83	1	9.50	56.43	26.97	9.46	56.40	26.93	9.52	56.47	26.92
84	1	11.62	56.74	25.90	11.66	56.73	26.02	11.73	56.76	26.03
85	1	10.54	56.04	24.70	10.65	56.09	24.74	10.72	56.14	24.74
86	1	8.37	55.45	24.87	8.49	55.43	24.73	8.55	55.55	24.69
87	1	7.39	52.72	25.55	7.70	52.60	25.08	7.79	52.71	24.79
88	1	6.28	54.03	25.08	6.42	53.83	25.05	6.49	53.85	25.21
89	1	7.41	53.36	23.88	7.45	53.58	23.62	7.26	53.94	23.61
90	1	13.82	54.23	24.44	13.94	54.29	24.66	13.98	54.28	24.68
91	1	13.26	55.88	24.21	13.47	55.98	24.60	13.54	55.97	24.64
92*	1	10.85	47.93	30.60	10.85	47.93	30.60	10.85	47.93	30.60
93*	1	9.10	48.52	30.77	9.10	48.52	30.77	9.10	48.52	30.77
94*	1	8.88	46.16	31.80	8.88	46.16	31.80	8.88	46.16	31.80
95*	1	10.24	45.81	30.77	10.24	45.81	30.77	10.24	45.81	30.77
96*	1	8.75	46.41	30.08	8.75	46.41	30.08	8.75	46.41	30.08
97*	1	9.24	48.04	33.05	9.24	48.04	33.05	9.24	48.04	33.05
98*	1	11.04	47.66	32.81	11.04	47.66	32.81	11.04	47.66	32.81
99	1	9.40	51.67	32.87	9.40	51.67	32.86	9.37	51.66	32.88
100	1	8.49	50.39	32.07	8.46	50.38	32.10	8.42	50.36	32.15
101	1	10.00	50.96	31.36	9.95	50.94	31.35	9.89	50.93	31.36
102	6	14.93	51.39	24.39	14.95	51.40	24.40	14.97	51.40	24.40
103	8	15.11	52.82	24.34	15.17	52.82	24.36	15.19	52.82	24.35
104	6	13.78	51.03	23.47	13.79	51.09	23.46	13.83	51.07	23.45
105	1	13.60	49.95	23.48	13.58	50.01	23.47	13.61	50.00	23.46
106	1	12.85	51.53	23.77	12.89	51.62	23.77	12.91	51.60	23.74
107	1	14.01	51.34	22.44	14.05	51.39	22.44	14.09	51.37	22.43
108	1	15.85	50.92	24.03	15.86	50.91	24.04	15.88	50.90	24.06
109	1	15.99	52.99	24.71	16.05	52.97	24.74	16.06	52.98	24.74
110	7	18.68	51.53	29.48	18.67	51.52	29.48	18.68	51.44	29.28

111	6	19.55	50.36	29.71	19.53	50.33	29.68	19.51	50.23	29.46
112	6	19.07	49.46	30.87	19.08	49.42	30.83	19.12	49.40	30.69
113	8	18.94	49.88	32.01	18.98	49.82	31.98	19.10	49.86	31.83
114	6	20.92	50.99	30.01	20.92	50.95	29.94	20.93	50.81	29.59
115	6	20.91	52.27	29.16	20.90	52.24	29.13	20.89	52.04	28.67
116	6	19.46	52.77	29.29	19.46	52.76	29.32	19.49	52.62	28.89
117	7	18.91	48.15	30.52	18.89	48.13	30.47	18.87	48.09	30.40
118	6	18.51	47.14	31.48	18.49	47.11	31.41	18.50	47.13	31.41
119	6	17.01	47.01	31.78	16.99	46.97	31.71	17.00	46.98	31.70
120	8	16.63	46.16	32.57	16.61	46.08	32.46	16.61	46.07	32.44
121	7	16.16	47.88	31.14	16.16	47.89	31.15	16.16	47.90	31.15
122	6	15.77	53.35	29.93	15.77	53.35	29.92	15.80	53.34	29.94
123	1	15.03	53.99	29.43	15.07	54.03	29.42	15.16	54.09	29.48
124	1	15.23	52.69	30.60	15.18	52.70	30.56	15.17	52.70	30.55
125	1	21.63	53.01	29.50	21.64	52.97	29.46	21.67	52.77	28.90
126	1	19.34	53.42	30.16	19.37	53.36	30.23	19.47	53.35	29.71
127	1	21.74	50.31	29.78	21.72	50.26	29.66	21.69	50.08	29.31
128	1	20.96	51.24	31.08	21.00	51.16	31.01	21.09	51.11	30.63
129	1	18.87	46.16	31.15	18.85	46.13	31.07	18.88	46.14	31.14
130	1	18.99	47.36	32.43	18.98	47.32	32.37	18.96	47.42	32.36
131	1	18.91	47.91	29.54	18.89	47.89	29.48	18.83	47.80	29.44
132	1	16.53	48.64	30.58	16.53	48.66	30.60	16.53	48.67	30.61
133	1	19.14	53.31	28.40	19.12	53.34	28.47	19.09	53.08	27.99
134	1	21.13	52.03	28.12	21.08	52.03	28.07	21.01	51.73	27.63
135	6	16.72	54.21	30.74	16.74	54.14	30.79	16.83	54.00	30.85
136	7	16.94	55.46	30.25	16.96	55.42	30.40	17.00	55.34	30.69
137	8	17.30	53.80	31.75	17.31	53.64	31.76	17.50	53.34	31.65
138	1	17.61	56.05	30.72	17.62	55.98	30.92	17.67	55.82	31.27
139	1	16.47	55.81	29.43	16.46	55.85	29.64	16.41	55.88	30.08
140	6	10.27	49.68	33.12	10.27	49.68	33.11	10.26	49.68	33.11
141	6	11.67	50.13	33.52	11.67	50.15	33.48	11.66	50.17	33.46
142	1	12.14	50.73	32.75	12.13	50.74	32.69	12.10	50.75	32.65
143	1	11.63	50.75	34.42	11.63	50.80	34.37	11.63	50.83	34.33
144	1	12.32	49.28	33.74	12.33	49.31	33.72	12.34	49.34	33.70
145	1	9.73	49.60	34.07	9.75	49.60	34.07	9.76	49.59	34.09

M121L/F114N (including Pro115, Gly116) – RED

Atom number	Atomic number	ε = 1			ε = 5.7			ε = 78.4		
		X	Y	Z	X	Y	Z	X	Y	Z
1	29	12.71	52.47	31.58	12.72	52.52	31.57	12.77	52.55	31.54
2	16	12.39	52.14	29.34	12.39	52.15	29.33	12.38	52.19	29.31
3	6	11.21	50.79	28.86	11.23	50.79	28.86	11.22	50.81	28.86
4*	6	11.21	50.54	27.34	11.21	50.54	27.34	11.21	50.54	27.34
5	6	12.62	50.13	26.88	12.62	50.13	26.87	12.62	50.14	26.88
6	8	13.06	48.99	27.06	13.05	48.99	27.05	13.05	49.00	27.07
7	7	13.37	51.12	26.30	13.37	51.12	26.30	13.37	51.12	26.30
8*	6	14.73	50.89	25.85	14.73	50.89	25.85	14.73	50.89	25.85
9	6	15.81	51.52	26.75	15.81	51.51	26.74	15.81	51.52	26.73
10	8	16.98	51.58	26.32	16.98	51.56	26.32	16.97	51.57	26.31
11	7	15.44	52.05	27.93	15.44	52.05	27.92	15.44	52.06	27.91
12*	6	16.48	52.55	28.82	16.48	52.55	28.81	16.48	52.55	28.82
13	6	17.35	51.33	29.22	17.34	51.33	29.22	17.33	51.31	29.20
14	8	16.79	50.33	29.69	16.77	50.35	29.73	16.75	50.34	29.72
15*	6	14.76	47.95	31.60	14.76	47.95	31.60	14.76	47.95	31.60
16	6	13.83	46.99	30.84	13.84	46.97	30.85	13.84	46.97	30.85
17	6	14.22	49.38	31.45	14.22	49.38	31.45	14.23	49.38	31.45
18	8	12.81	46.56	31.39	12.85	46.50	31.42	12.85	46.50	31.42
19	7	14.15	46.72	29.55	14.15	46.73	29.55	14.15	46.73	29.55
20	6	14.78	50.35	32.46	14.77	50.35	32.46	14.77	50.35	32.46
21*	6	13.32	45.88	28.69	13.32	45.88	28.69	13.32	45.88	28.69
22	6	15.77	50.16	33.39	15.74	50.17	33.41	15.72	50.16	33.43
23	7	14.30	51.65	32.56	14.30	51.66	32.55	14.32	51.67	32.53
24	7	15.88	51.36	34.08	15.85	51.36	34.09	15.84	51.37	34.10
25	6	14.98	52.22	33.55	14.96	52.23	33.54	14.97	52.24	33.53
26	7	11.47	53.41	32.88	11.50	53.45	32.91	11.55	53.47	32.92
27	6	10.45	54.32	32.60	10.47	54.35	32.67	10.51	54.37	32.74
28	6	11.55	53.36	34.20	11.64	53.39	34.22	11.75	53.40	34.23
29	6	10.11	54.71	31.19	10.07	54.75	31.29	10.04	54.80	31.39
30	6	9.92	54.79	33.77	10.00	54.83	33.87	10.08	54.82	33.97
31	7	10.63	54.17	34.78	10.75	54.21	34.84	10.87	54.19	34.90
32	6	11.37	55.21	30.44	11.31	55.25	30.48	11.24	55.28	30.53
33	7	12.11	56.21	31.19	12.08	56.24	31.20	12.05	56.26	31.23
34	6	10.98	55.83	29.10	10.86	55.87	29.15	10.76	55.90	29.22
35	6	13.25	55.92	31.84	13.24	55.94	31.82	13.24	55.96	31.79
36	8	10.80	57.05	29.01	10.60	57.08	29.08	10.39	57.08	29.18
37	7	10.82	54.95	28.10	10.75	55.00	28.13	10.74	55.06	28.17
38*	6	13.96	57.07	32.51	13.96	57.07	32.51	13.96	57.07	32.51
39	8	13.71	54.76	31.93	13.71	54.78	31.84	13.72	54.81	31.74
40	6	10.26	55.36	26.82	10.22	55.42	26.85	10.20	55.46	26.88
41	6	9.33	54.24	26.31	9.25	54.33	26.32	9.21	54.39	26.37
42	6	11.38	55.81	25.85	11.34	55.83	25.87	11.32	55.84	25.89
43	8	9.01	53.30	27.03	8.97	53.33	26.99	8.91	53.40	27.04
44	7	8.79	54.44	25.07	8.67	54.61	25.13	8.66	54.64	25.16

45	6	12.30	54.66	25.46	12.27	54.68	25.51	12.25	54.69	25.55
46	6	7.98	53.42	24.44	7.72	53.72	24.48	7.65	53.76	24.59
47	7	13.57	54.98	25.12	13.53	55.00	25.17	13.49	55.01	25.16
48	8	11.88	53.50	25.44	11.87	53.51	25.53	11.85	53.51	25.61
49*	6	9.90	47.88	31.09	9.90	47.88	31.09	9.90	47.88	31.09
50*	6	9.41	46.46	30.92	9.41	46.46	30.92	9.41	46.46	30.92
51*	6	10.16	48.22	32.54	10.16	48.22	32.54	10.16	48.22	32.54
52	6	9.50	50.72	32.27	9.47	50.72	32.28	9.44	50.71	32.30
53	1	11.49	49.87	29.38	11.52	49.87	29.38	11.52	49.90	29.39
54	1	10.20	51.06	29.17	10.22	51.05	29.19	10.21	51.06	29.18
55*	1	10.85	51.40	26.82	10.85	51.40	26.82	10.85	51.40	26.82
56*	1	10.60	49.70	27.10	10.60	49.70	27.10	10.60	49.70	27.10
57	1	12.93	52.02	26.12	12.94	52.03	26.13	12.93	52.03	26.13
58*	1	14.85	49.83	25.80	14.85	49.83	25.80	14.85	49.83	25.80
59	1	14.49	51.91	28.29	14.49	51.93	28.28	14.48	51.95	28.27
60*	1	17.09	53.28	28.31	17.09	53.28	28.31	17.09	53.28	28.31
61	1	19.18	49.42	28.32	19.09	49.37	28.23	19.04	49.33	28.13
62*	1	14.78	47.62	32.62	14.78	47.62	32.62	14.78	47.62	32.62
63	1	13.14	49.35	31.54	13.13	49.35	31.54	13.14	49.35	31.54
64	1	14.43	49.76	30.44	14.43	49.75	30.44	14.44	49.75	30.44
65	1	14.99	47.14	29.18	14.98	47.15	29.18	15.00	47.12	29.19
66*	1	13.80	44.94	28.50	13.80	44.94	28.50	13.80	44.94	28.50
67*	1	12.41	45.72	29.23	12.41	45.72	29.23	12.41	45.72	29.23
68*	1	13.11	46.37	27.76	13.11	46.37	27.76	13.11	46.37	27.76
69	1	16.39	49.32	33.62	16.36	49.32	33.66	16.32	49.31	33.70
70	1	16.56	51.57	34.79	16.49	51.56	34.84	16.45	51.56	34.87
71	1	14.86	53.24	33.87	14.84	53.25	33.87	14.87	53.26	33.85
72	1	12.24	52.75	34.76	12.35	52.79	34.76	12.49	52.80	34.73
73	1	9.33	55.48	31.20	9.31	55.53	31.34	9.30	55.59	31.49
74	1	9.73	53.84	30.63	9.65	53.90	30.74	9.57	53.97	30.85
75	1	9.12	55.49	33.97	9.22	55.54	34.10	9.31	55.52	34.24
76	1	10.47	54.27	35.77	10.65	54.32	35.84	10.82	54.30	35.90
77	1	12.01	54.34	30.33	11.94	54.38	30.34	11.87	54.41	30.37
78	1	11.84	57.17	31.01	11.78	57.20	31.12	11.71	57.22	31.24
79	1	11.04	53.97	28.26	11.03	54.03	28.27	11.08	54.10	28.30
80	1	13.46	58.03	32.36	13.45	58.03	32.40	13.44	58.03	32.45
81	1	14.98	57.13	32.12	14.97	57.15	32.11	14.96	57.18	32.08
82	1	14.03	56.87	33.58	14.04	56.84	33.57	14.07	56.79	33.56
83	1	9.64	56.25	27.02	9.62	56.32	27.04	9.62	56.37	27.07
84	1	11.93	56.62	26.32	11.90	56.65	26.32	11.89	56.67	26.32
85	1	10.95	56.24	24.93	10.92	56.22	24.93	10.89	56.22	24.95
86	1	9.22	55.13	24.47	8.92	55.46	24.64	8.85	55.51	24.69
87	1	7.46	52.87	25.22	7.51	52.90	25.17	7.99	52.72	24.63
88	1	7.24	53.89	23.78	6.78	54.25	24.26	6.70	53.83	25.12
89	1	8.58	52.71	23.86	8.12	53.31	23.55	7.49	54.03	23.54
90	1	14.21	54.23	24.83	14.17	54.26	24.87	14.13	54.26	24.86
91	1	13.90	55.93	25.18	13.85	55.96	25.17	13.80	55.97	25.11
92*	1	10.85	47.93	30.60	10.85	47.93	30.60	10.85	47.93	30.60
93*	1	9.10	48.52	30.77	9.10	48.52	30.77	9.10	48.52	30.77

94*	1	8.88	46.16	31.80	8.88	46.16	31.80	8.88	46.16	31.80
95*	1	10.24	45.81	30.77	10.24	45.81	30.77	10.24	45.81	30.77
96*	1	8.75	46.41	30.08	8.75	46.41	30.08	8.75	46.41	30.08
97*	1	9.24	48.04	33.05	9.24	48.04	33.05	9.24	48.04	33.05
98*	1	11.04	47.66	32.81	11.04	47.66	32.81	11.04	47.66	32.81
99	1	9.36	51.65	32.84	9.37	51.66	32.84	9.37	51.66	32.84
100	1	8.50	50.36	32.00	8.47	50.36	32.05	8.42	50.35	32.12
101	1	10.03	50.98	31.35	9.98	50.96	31.34	9.90	50.92	31.33
102	6	15.00	51.39	24.40	14.99	51.39	24.39	14.98	51.38	24.39
103	8	15.35	52.79	24.36	15.31	52.79	24.35	15.27	52.79	24.33
104	6	13.83	51.17	23.45	13.83	51.15	23.45	13.82	51.10	23.45
105	1	13.53	50.12	23.45	13.54	50.09	23.46	13.56	50.04	23.47
106	1	12.97	51.78	23.75	12.96	51.74	23.74	12.94	51.69	23.74
107	1	14.13	51.45	22.43	14.11	51.42	22.43	14.10	51.37	22.43
108	1	15.87	50.83	24.04	15.87	50.84	24.04	15.87	50.85	24.04
109	1	16.20	52.84	24.82	16.17	52.87	24.80	16.13	52.90	24.76
110	7	18.68	51.34	28.99	18.66	51.30	28.94	18.64	51.25	28.87
111	6	19.45	50.10	29.14	19.41	50.03	29.05	19.38	49.98	28.95
112	6	19.23	49.40	30.48	19.22	49.31	30.38	19.19	49.24	30.29
113	8	19.45	49.93	31.56	19.47	49.82	31.47	19.46	49.76	31.37
114	6	20.90	50.57	28.97	20.87	50.47	28.83	20.84	50.41	28.73
115	6	20.78	51.68	27.91	20.73	51.61	27.81	20.71	51.59	27.76
116	6	19.46	52.39	28.28	19.45	52.34	28.24	19.44	52.32	28.23
117	7	18.87	48.08	30.36	18.82	48.01	30.25	18.78	47.95	30.15
118	6	18.54	47.26	31.51	18.50	47.16	31.37	18.48	47.09	31.27
119	6	17.05	47.11	31.85	17.01	47.00	31.71	17.00	46.96	31.64
120	8	16.72	46.33	32.73	16.66	46.11	32.48	16.63	46.04	32.38
121	7	16.16	47.89	31.16	16.16	47.90	31.15	16.15	47.89	31.14
122	6	15.89	53.29	30.01	15.88	53.30	30.00	15.88	53.29	30.01
123	1	15.13	54.00	29.63	15.20	54.06	29.62	15.31	54.14	29.63
124	1	15.35	52.63	30.69	15.26	52.66	30.64	15.14	52.69	30.56
125	1	21.62	52.37	27.92	21.60	52.27	27.80	21.58	52.24	27.76
126	1	19.60	53.24	28.95	19.65	53.16	28.93	19.66	53.09	28.97
127	1	21.57	49.76	28.67	21.49	49.64	28.49	21.45	49.59	28.35
128	1	21.25	50.98	29.93	21.26	50.84	29.78	21.25	50.74	29.69
129	1	18.94	46.25	31.39	18.91	46.15	31.22	18.87	46.09	31.10
130	1	19.02	47.71	32.38	18.99	47.59	32.25	19.00	47.50	32.15
131	1	18.56	47.76	29.45	18.54	47.69	29.33	18.52	47.63	29.23
132	1	16.52	48.68	30.61	16.53	48.69	30.62	16.52	48.68	30.61
133	1	18.91	52.71	27.39	18.89	52.71	27.39	18.89	52.75	27.40
134	1	20.70	51.24	26.91	20.61	51.20	26.80	20.57	51.22	26.74
135	6	16.96	54.10	30.74	16.96	54.00	30.82	16.96	53.79	30.96
136	7	16.55	54.65	31.92	16.52	54.64	31.92	16.53	54.66	31.90
137	8	18.09	54.26	30.30	18.15	54.01	30.48	18.14	53.46	30.85
138	1	17.20	55.28	32.36	17.19	55.19	32.45	17.18	54.95	32.61
139	1	15.56	54.70	32.14	15.52	54.73	32.10	15.53	54.81	32.03
140	6	10.26	49.69	33.10	10.26	49.69	33.10	10.26	49.69	33.10
141	6	11.66	50.15	33.51	11.66	50.17	33.49	11.66	50.19	33.45
142	8	12.08	50.85	32.79	12.07	50.87	32.75	12.07	50.87	32.70

143	6	11.63	50.67	34.47	11.62	50.72	34.43	11.63	50.76	34.38
144	6	12.35	49.31	33.63	12.36	49.34	33.63	12.37	49.37	33.60
145	6	9.71	49.61	34.05	9.73	49.61	34.06	9.75	49.60	34.07

M121L/F114P – OX

Atom number	Atomic number	ε = 1			ε = 5.7			ε = 78.4		
		X	Y	Z	X	Y	Z	X	Y	Z
1	29	12.70	52.45	31.53	12.73	52.51	31.51	12.74	52.53	31.51
2	16	12.05	52.18	29.48	12.06	52.21	29.46	12.07	52.22	29.45
3	6	11.09	50.72	28.89	11.12	50.73	28.89	11.12	50.74	28.90
4	6	11.24	50.54	27.38	11.24	50.54	27.37	11.24	50.54	27.37
5	6	12.68	50.19	27.01	12.67	50.18	26.99	12.68	50.19	27.00
6	8	13.21	49.15	27.41	13.18	49.11	27.34	13.19	49.13	27.37
7	7	13.35	51.11	26.25	13.35	51.11	26.26	13.35	51.11	26.26
8*	6	14.73	50.89	25.85	14.73	50.89	25.85	14.73	50.89	25.85
9	6	15.81	51.32	26.85	15.82	51.33	26.83	15.83	51.34	26.82
10	8	16.90	50.74	26.82	16.91	50.75	26.79	16.91	50.74	26.78
11	7	15.54	52.29	27.76	15.56	52.31	27.74	15.57	52.31	27.73
12*	6	16.52	52.53	28.81	16.52	52.53	28.81	16.52	52.53	28.81
13	6	17.04	51.24	29.46	16.97	51.23	29.49	16.94	51.23	29.51
14	8	16.29	50.41	29.96	16.22	50.27	29.67	16.21	50.24	29.62
15	6	15.08	54.42	28.73	15.13	54.45	28.67	15.16	54.47	28.64
16	6	14.63	53.45	27.62	14.65	53.47	27.59	14.66	53.48	27.57
17	7	18.40	51.14	29.52	18.25	51.25	29.94	18.19	51.27	30.03
18	6	19.02	49.91	29.97	18.87	50.09	30.54	18.79	50.12	30.70
19*	6	14.76	47.95	31.60	14.76	47.95	31.60	14.76	47.95	31.60
20	6	13.87	46.94	30.85	13.92	46.90	30.86	13.93	46.89	30.86
21	6	14.11	49.34	31.50	14.12	49.34	31.47	14.11	49.34	31.48
22	8	12.94	46.39	31.43	13.08	46.22	31.47	13.11	46.20	31.47
23	7	14.16	46.74	29.54	14.15	46.76	29.53	14.14	46.77	29.53
24	6	14.74	50.38	32.38	14.75	50.39	32.35	14.74	50.39	32.35
25*	6	13.32	45.88	28.69	13.32	45.88	28.69	13.32	45.88	28.69
26	6	15.83	50.28	33.21	15.83	50.28	33.19	15.80	50.28	33.22
27	7	14.30	51.70	32.42	14.32	51.72	32.38	14.32	51.72	32.37
28	7	16.05	51.54	33.73	16.05	51.54	33.71	16.03	51.54	33.73
29	6	15.11	52.38	33.23	15.13	52.38	33.20	15.12	52.38	33.20
30	7	11.66	53.36	32.99	11.72	53.38	33.00	11.74	53.39	33.01
31	6	10.61	54.26	32.82	10.66	54.27	32.87	10.68	54.28	32.89
32	6	11.89	53.29	34.29	11.99	53.31	34.30	12.03	53.32	34.30
33	6	10.09	54.68	31.48	10.10	54.69	31.54	10.10	54.71	31.58
34	6	10.21	54.70	34.06	10.30	54.72	34.11	10.35	54.73	34.14
35	7	11.04	54.08	34.97	11.15	54.10	35.00	11.21	54.11	35.01
36	6	11.22	55.29	30.60	11.19	55.31	30.62	11.17	55.32	30.63
37	7	12.02	56.27	31.31	12.01	56.29	31.30	12.00	56.30	31.29
38	6	10.63	55.95	29.36	10.56	55.95	29.39	10.51	55.94	29.40
39	6	13.22	55.94	31.83	13.22	55.96	31.80	13.22	55.97	31.78

40	8	10.27	57.13	29.38	10.13	57.10	29.41	10.02	57.08	29.45
41*	6	13.96	57.07	32.51	13.96	57.07	32.51	13.96	57.07	32.51
42	8	13.68	54.79	31.80	13.70	54.81	31.72	13.69	54.82	31.68
43	7	7.98	54.47	25.77	8.05	54.47	25.69	8.11	54.44	25.63
44	6	6.95	53.50	25.40	7.02	53.51	25.30	7.09	53.48	25.24
45*	6	9.90	47.88	31.09	9.90	47.88	31.09	9.90	47.88	31.09
46*	6	9.41	46.46	30.92	9.41	46.46	30.92	9.41	46.46	30.92
47*	6	10.16	48.22	32.54	10.16	48.22	32.54	10.16	48.22	32.54
48	6	9.53	50.75	32.31	9.51	50.74	32.31	9.52	50.74	32.30
49	7	10.55	55.13	28.29	10.52	55.13	28.31	10.51	55.14	28.32
50*	6	9.79	55.45	27.11	9.79	55.45	27.11	9.79	55.45	27.11
51	6	8.81	54.28	26.83	8.80	54.30	26.80	8.80	54.31	26.79
52	6	10.71	55.80	25.91	10.74	55.80	25.94	10.75	55.81	25.95
53	6	11.68	54.68	25.52	11.69	54.67	25.54	11.67	54.66	25.53
54	8	8.79	53.29	27.55	8.71	53.32	27.53	8.63	53.36	27.56
55	7	12.63	54.99	24.62	12.64	54.98	24.63	12.60	54.95	24.60
56	8	11.58	53.55	26.01	11.58	53.54	26.02	11.56	53.53	26.03
57	6	15.78	53.49	29.74	15.79	53.54	29.71	15.80	53.56	29.69
58	1	13.31	54.28	24.35	13.31	54.26	24.36	13.28	54.23	24.34
59	1	12.70	55.91	24.22	12.72	55.91	24.25	12.71	55.88	24.23
60	1	14.24	54.96	29.16	14.30	55.03	29.09	14.34	55.06	29.05
61	1	15.79	55.15	28.34	15.87	55.15	28.26	15.91	55.15	28.23
62	1	13.60	53.14	27.78	13.62	53.17	27.78	13.63	53.20	27.76
63	1	14.71	53.89	26.63	14.71	53.88	26.59	14.74	53.89	26.56
64	1	11.44	49.83	29.41	11.49	49.84	29.41	11.48	49.86	29.42
65	1	10.05	50.90	29.15	10.07	50.88	29.17	10.07	50.90	29.17
66*	1	10.85	51.40	26.82	10.85	51.40	26.82	10.85	51.40	26.82
67*	1	10.60	49.70	27.10	10.60	49.70	27.10	10.60	49.70	27.10
68	1	12.87	51.97	26.00	12.88	51.99	26.03	12.87	51.98	26.02
69*	1	14.85	49.83	25.80	14.85	49.83	25.80	14.85	49.83	25.80
70	1	18.91	51.68	28.83	18.86	51.99	29.61	18.78	52.06	29.83
71	1	18.93	49.11	29.22	19.11	49.31	29.80	18.97	49.30	30.00
72	1	20.08	50.09	30.17	19.80	50.39	31.03	19.74	50.43	31.14
73*	1	14.78	47.62	32.62	14.78	47.62	32.62	14.78	47.62	32.62
74	1	13.05	49.24	31.75	13.05	49.26	31.72	13.05	49.26	31.72
75	1	14.17	49.70	30.47	14.19	49.69	30.44	14.18	49.69	30.44
76	1	14.83	47.34	29.09	14.74	47.44	29.07	14.74	47.44	29.07
77*	1	13.80	44.94	28.50	13.80	44.94	28.50	13.80	44.94	28.50
78*	1	12.41	45.72	29.23	12.41	45.72	29.23	12.41	45.72	29.23
79*	1	13.11	46.37	27.76	13.11	46.37	27.76	13.11	46.37	27.76
80	1	16.47	49.45	33.44	16.45	49.44	33.46	16.41	49.43	33.50
81	1	16.80	51.81	34.35	16.79	51.80	34.35	16.75	51.80	34.39
82	1	15.06	53.44	33.42	15.08	53.44	33.40	15.07	53.44	33.40
83	1	12.65	52.68	34.77	12.76	52.71	34.76	12.81	52.72	34.75
84	1	9.29	55.41	31.63	9.30	55.43	31.72	9.31	55.44	31.77
85	1	9.67	53.83	30.94	9.65	53.84	31.01	9.64	53.86	31.05
86	1	9.44	55.40	34.35	9.53	55.41	34.43	9.59	55.43	34.47
87	1	10.99	54.18	35.98	11.14	54.20	36.01	11.23	54.22	36.02
88	1	11.88	54.48	30.32	11.85	54.51	30.32	11.83	54.52	30.33

89	1	11.71	57.23	31.24	11.67	57.24	31.33	11.66	57.25	31.37
90	1	13.51	58.05	32.33	13.50	58.05	32.35	13.52	58.06	32.35
91	1	14.99	57.08	32.16	14.99	57.09	32.16	15.00	57.08	32.18
92	1	13.98	56.89	33.59	13.97	56.86	33.58	13.95	56.85	33.58
93	1	7.98	55.36	25.30	8.07	55.36	25.20	8.20	55.28	25.09
94	1	7.13	52.60	25.98	7.38	52.50	25.51	7.45	52.46	25.41
95	1	5.95	53.88	25.62	6.09	53.67	25.85	6.16	53.61	25.82
96	1	7.02	53.27	24.33	6.83	53.61	24.23	6.86	53.61	24.18
97*	1	10.85	47.93	30.60	10.85	47.93	30.60	10.85	47.93	30.60
98*	1	9.10	48.52	30.77	9.10	48.52	30.77	9.10	48.52	30.77
99*	1	8.88	46.16	31.80	8.88	46.16	31.80	8.88	46.16	31.80
100*	1	10.24	45.81	30.77	10.24	45.81	30.77	10.24	45.81	30.77
101*	1	8.75	46.41	30.08	8.75	46.41	30.08	8.75	46.41	30.08
102*	1	9.24	48.04	33.05	9.24	48.04	33.05	9.24	48.04	33.05
103*	1	11.04	47.66	32.81	11.04	47.66	32.81	11.04	47.66	32.81
104	1	9.38	51.66	32.89	9.40	51.67	32.88	9.42	51.67	32.87
105	1	8.54	50.41	31.99	8.51	50.40	32.04	8.52	50.41	32.03
106	1	10.10	51.02	31.41	10.04	50.98	31.38	10.06	50.98	31.38
107	1	18.54	49.58	30.89	18.20	49.66	31.28	18.13	49.75	31.49
108*	1	15.76	47.98	31.21	15.76	47.98	31.20	15.76	47.98	31.21
109	1	16.45	54.02	30.42	16.47	54.06	30.39	16.49	54.08	30.37
110	1	15.04	52.95	30.32	15.03	53.03	30.30	15.03	53.07	30.29
111	1	10.12	56.07	25.03	10.17	56.11	25.05	10.19	56.15	25.07
112	1	11.29	56.69	26.17	11.34	56.68	26.23	11.37	56.66	26.26
113	1	9.21	56.35	27.34	9.21	56.35	27.32	9.20	56.35	27.32
114	1	10.81	54.15	28.39	10.85	54.17	28.40	10.91	54.21	28.39
115	1	17.39	53.06	28.39	17.43	53.01	28.41	17.44	52.99	28.41
116	6	15.01	51.43	24.43	15.00	51.42	24.42	14.99	51.42	24.42
117	8	14.87	52.88	24.37	14.84	52.86	24.34	14.80	52.84	24.33
118	6	14.09	50.85	23.37	14.07	50.81	23.38	14.08	50.77	23.38
119	1	16.05	51.17	24.21	16.04	51.17	24.19	16.04	51.18	24.19
120	1	14.17	49.76	23.36	14.16	49.72	23.39	14.20	49.68	23.40
121	1	13.05	51.11	23.57	13.03	51.07	23.58	13.03	51.01	23.57
122	1	14.38	51.23	22.39	14.34	51.17	22.39	14.34	51.13	22.38
123	1	15.72	53.27	24.61	15.69	53.27	24.55	15.64	53.28	24.50
124	6	10.25	49.68	33.13	10.26	49.68	33.13	10.26	49.68	33.13
106	6	11.63	50.12	33.63	11.64	50.13	33.59	11.64	50.13	33.60
107	1	12.16	50.72	32.90	12.16	50.73	32.84	12.16	50.72	32.85
108	1	11.53	50.73	34.54	11.55	50.77	34.48	11.54	50.77	34.48
109	1	12.26	49.26	33.90	12.28	49.29	33.87	12.27	49.29	33.88
110	1	9.66	49.59	34.06	9.69	49.59	34.06	9.68	49.60	34.06

M121L/F114P – RED

Atom number	Atomic number	ε = 1			ε = 5.7			ε = 78.4		
		X	Y	Z	X	Y	Z	X	Y	Z
1	29	12.74	52.49	31.49	12.79	52.55	31.49	12.76	52.62	31.51
2	16	11.67	52.30	29.52	11.77	52.31	29.48	11.83	52.31	29.46
3	6	11.08	50.68	28.90	11.08	50.71	28.90	11.09	50.73	28.90
4	6	11.24	50.54	27.37	11.23	50.54	27.38	11.23	50.54	27.38
5	6	12.67	50.19	27.00	12.68	50.19	27.01	12.67	50.20	27.01
6	8	13.21	49.14	27.38	13.20	49.14	27.39	13.20	49.15	27.40
7	7	13.34	51.11	26.24	13.35	51.11	26.26	13.35	51.11	26.26
8*	6	14.73	50.89	25.85	14.73	50.89	25.85	14.73	50.89	25.85
9	6	15.81	51.32	26.84	15.83	51.34	26.82	15.83	51.34	26.82
10	8	16.92	50.78	26.78	16.94	50.78	26.73	16.93	50.77	26.75
11	7	15.54	52.27	27.77	15.58	52.30	27.73	15.57	52.30	27.73
12*	6	16.52	52.53	28.81	16.52	52.53	28.81	16.52	52.53	28.81
13	6	17.07	51.24	29.44	16.96	51.22	29.49	16.96	51.23	29.51
14	8	16.36	50.34	29.86	16.25	50.23	29.58	16.26	50.22	29.58
15	6	14.99	54.34	28.77	15.06	54.40	28.69	15.11	54.43	28.66
16	6	14.49	53.32	27.75	14.55	53.38	27.67	14.59	53.41	27.64
17	7	18.43	51.23	29.59	18.20	51.28	30.04	18.19	51.30	30.07
18	6	19.10	50.02	30.04	18.81	50.13	30.68	18.78	50.19	30.79
19*	6	14.76	47.95	31.60	14.76	47.95	31.60	14.76	47.95	31.60
20	6	13.94	46.88	30.86	13.96	46.87	30.86	13.95	46.87	30.86
21	6	14.12	49.34	31.47	14.13	49.34	31.46	14.13	49.35	31.46
22	8	13.13	46.17	31.46	13.19	46.11	31.47	13.17	46.14	31.47
23	7	14.15	46.77	29.51	14.14	46.78	29.51	14.14	46.78	29.52
24	6	14.74	50.37	32.36	14.76	50.37	32.36	14.74	50.38	32.37
25*	6	13.32	45.88	28.69	13.32	45.88	28.69	13.32	45.88	28.69
26	6	15.80	50.25	33.23	15.77	50.22	33.27	15.74	50.23	33.30
27	7	14.32	51.70	32.36	14.36	51.70	32.34	14.35	51.71	32.34
28	7	16.02	51.51	33.75	16.00	51.48	33.80	15.96	51.49	33.83
29	6	15.11	52.35	33.20	15.13	52.34	33.21	15.11	52.35	33.23
30	7	11.62	53.48	32.98	11.66	53.50	32.99	11.63	53.54	33.01
31	6	10.54	54.36	32.85	10.58	54.37	32.87	10.58	54.44	32.93
32	6	11.85	53.39	34.28	11.92	53.42	34.28	11.93	53.45	34.30
33	6	10.04	54.79	31.51	10.04	54.79	31.54	10.01	54.89	31.62
34	6	10.14	54.77	34.09	10.19	54.79	34.12	10.25	54.87	34.19
35	7	10.98	54.15	34.99	11.05	54.18	35.01	11.11	54.23	35.05
36	6	11.20	55.29	30.62	11.18	55.32	30.63	11.13	55.35	30.66
37	7	12.04	56.26	31.29	12.03	56.28	31.29	12.02	56.32	31.27
38	6	10.69	55.91	29.32	10.62	55.94	29.35	10.54	55.95	29.38
39	6	13.24	55.94	31.81	13.24	55.96	31.78	13.23	55.97	31.77
40	8	10.56	57.14	29.21	10.34	57.14	29.30	10.16	57.13	29.35
41*	6	13.96	57.07	32.51	13.96	57.07	32.51	13.96	57.07	32.51
42	8	13.73	54.80	31.78	13.74	54.82	31.70	13.71	54.83	31.68
43	7	8.24	54.62	25.40	8.11	54.59	25.54	8.13	54.54	25.54
44	6	7.31	53.68	24.78	7.07	53.71	25.05	7.07	53.64	25.11
45*	6	9.90	47.88	31.09	9.90	47.88	31.09	9.90	47.88	31.09

46*	6	9.41	46.46	30.92	9.41	46.46	30.92	9.41	46.46	30.92
47*	6	10.16	48.22	32.54	10.16	48.22	32.54	10.16	48.22	32.54
48	6	9.40	50.71	32.36	9.40	50.71	32.36	9.38	50.70	32.36
49	7	10.38	55.03	28.35	10.45	55.07	28.33	10.46	55.09	28.34
50*	6	9.79	55.45	27.11	9.79	55.45	27.11	9.79	55.45	27.11
51	6	8.82	54.36	26.61	8.76	54.37	26.71	8.75	54.37	26.73
52	6	10.87	55.88	26.08	10.80	55.83	25.99	10.80	55.81	25.99
53	6	11.76	54.73	25.57	11.69	54.68	25.51	11.68	54.66	25.51
54	8	8.53	53.39	27.30	8.52	53.41	27.44	8.46	53.45	27.50
55	7	12.86	55.11	24.88	12.79	55.05	24.81	12.75	55.01	24.77
56	8	11.48	53.55	25.75	11.41	53.50	25.71	11.42	53.48	25.77
57	6	15.76	53.47	29.76	15.76	53.52	29.72	15.78	53.54	29.71
58	1	13.51	54.39	24.57	13.43	54.33	24.49	13.40	54.29	24.46
59	1	13.14	56.08	24.81	13.04	56.02	24.69	12.99	55.98	24.61
60	1	14.16	54.87	29.24	14.24	54.97	29.12	14.29	55.02	29.08
61	1	15.66	55.08	28.30	15.77	55.11	28.23	15.83	55.12	28.20
62	1	13.53	52.91	28.08	13.58	52.99	27.99	13.60	53.07	27.93
63	1	14.38	53.73	26.74	14.48	53.78	26.66	14.56	53.81	26.62
64	1	11.62	49.87	29.38	11.58	49.88	29.40	11.56	49.89	29.40
65	1	10.03	50.58	29.15	10.02	50.67	29.15	10.02	50.71	29.15
66*	1	10.85	51.40	26.82	10.85	51.40	26.82	10.85	51.40	26.82
67*	1	10.60	49.70	27.10	10.60	49.70	27.10	10.60	49.70	27.10
68	1	12.86	51.97	25.99	12.86	51.96	26.00	12.86	51.97	26.00
69*	1	14.85	49.83	25.80	14.85	49.83	25.80	14.85	49.83	25.80
70	1	18.95	51.82	28.95	18.80	52.06	29.81	18.73	52.15	29.96
71	1	19.12	49.24	29.26	19.07	49.35	29.96	18.74	49.28	30.18
72	1	20.12	50.25	30.34	19.72	50.45	31.20	19.83	50.42	30.99
73*	1	14.78	47.62	32.62	14.78	47.62	32.62	14.78	47.62	32.62
74	1	13.05	49.26	31.69	13.06	49.28	31.67	13.05	49.27	31.67
75	1	14.20	49.70	30.44	14.23	49.70	30.43	14.23	49.69	30.44
76	1	14.63	47.53	29.04	14.62	47.54	29.04	14.67	47.50	29.05
77*	1	13.80	44.94	28.50	13.80	44.94	28.50	13.80	44.94	28.50
78*	1	12.41	45.72	29.23	12.41	45.72	29.23	12.41	45.72	29.23
79*	1	13.11	46.37	27.76	13.11	46.37	27.76	13.11	46.37	27.76
80	1	16.41	49.40	33.50	16.35	49.37	33.59	16.31	49.37	33.62
81	1	16.75	51.77	34.40	16.70	51.72	34.49	16.64	51.73	34.53
82	1	15.06	53.41	33.38	15.10	53.40	33.41	15.07	53.41	33.43
83	1	12.63	52.80	34.73	12.70	52.84	34.74	12.71	52.84	34.73
84	1	9.28	55.57	31.63	9.27	55.55	31.68	9.30	55.71	31.80
85	1	9.58	53.95	30.98	9.59	53.93	31.01	9.47	54.08	31.12
86	1	9.35	55.44	34.40	9.40	55.45	34.44	9.50	55.57	34.54
87	1	10.93	54.22	36.00	11.03	54.26	36.01	11.14	54.32	36.05
88	1	11.83	54.42	30.44	11.81	54.46	30.42	11.73	54.47	30.46
89	1	11.76	57.23	31.17	11.71	57.24	31.30	11.69	57.27	31.35
90	1	13.56	58.06	32.26	13.52	58.06	32.32	13.55	58.06	32.31
91	1	15.02	57.04	32.25	15.01	57.08	32.21	15.02	57.05	32.24
92	1	13.87	56.93	33.59	13.92	56.87	33.58	13.89	56.88	33.58
93	1	8.54	55.42	24.87	8.29	55.44	25.03	8.33	55.35	24.98
94	1	7.10	52.90	25.51	7.12	52.78	25.61	7.42	52.60	25.14

95	1	6.38	54.18	24.50	6.07	54.15	25.18	6.19	53.73	25.75
96	1	7.75	53.22	23.88	7.22	53.50	23.98	6.80	53.89	24.08
97*	1	10.85	47.93	30.60	10.85	47.93	30.60	10.85	47.93	30.60
98*	1	9.10	48.52	30.77	9.10	48.52	30.77	9.10	48.52	30.77
99*	1	8.88	46.16	31.80	8.88	46.16	31.80	8.88	46.16	31.80
100*	1	10.24	45.81	30.77	10.24	45.81	30.77	10.24	45.81	30.77
101*	1	8.75	46.41	30.08	8.75	46.41	30.08	8.75	46.41	30.08
102*	1	9.24	48.04	33.05	9.24	48.04	33.05	9.24	48.04	33.05
103*	1	11.04	47.66	32.81	11.04	47.66	32.81	11.04	47.66	32.81
104	1	9.29	51.63	32.95	9.30	51.63	32.94	9.29	51.63	32.92
105	1	8.40	50.33	32.16	8.39	50.32	32.17	8.37	50.31	32.20
106	1	9.86	50.99	31.41	9.84	50.98	31.40	9.81	50.94	31.38
107	1	18.56	49.62	30.90	18.11	49.70	31.41	18.27	49.99	31.73
108*	1	15.76	47.98	31.20	15.76	47.98	31.21	15.76	47.98	31.21
109	1	16.43	54.02	30.43	16.43	54.05	30.39	16.47	54.08	30.37
110	1	15.04	52.91	30.36	15.00	53.00	30.31	15.02	53.05	30.31
111	1	10.41	56.37	25.21	10.28	56.24	25.12	10.27	56.22	25.11
112	1	11.49	56.64	26.55	11.43	56.64	26.38	11.43	56.62	26.35
113	1	9.21	56.36	27.32	9.23	56.37	27.33	9.23	56.37	27.32
114	1	10.56	54.02	28.52	10.70	54.08	28.49	10.78	54.12	28.48
115	1	17.38	53.08	28.39	17.43	53.02	28.42	17.43	53.00	28.41
116	6	15.04	51.47	24.45	15.00	51.43	24.43	15.00	51.42	24.42
117	8	15.01	52.91	24.45	14.91	52.87	24.36	14.87	52.86	24.34
118	6	14.08	51.00	23.37	14.04	50.88	23.38	14.06	50.83	23.38
119	1	16.06	51.14	24.21	16.03	51.13	24.18	16.03	51.15	24.18
120	1	14.07	49.91	23.32	14.07	49.78	23.38	14.12	49.74	23.39
121	1	13.06	51.34	23.59	13.02	51.19	23.59	13.02	51.12	23.58
122	1	14.38	51.40	22.40	14.32	51.24	22.39	14.34	51.18	22.39
123	1	15.78	53.22	24.95	15.73	53.24	24.73	15.70	53.26	24.62
124	6	10.25	49.68	33.13	10.25	49.69	33.12	10.25	49.69	33.12
106	6	11.63	50.22	33.51	11.64	50.23	33.49	11.63	50.25	33.47
107	1	12.06	50.88	32.76	12.06	50.88	32.72	12.05	50.90	32.71
108	1	11.54	50.82	34.42	11.55	50.85	34.39	11.54	50.86	34.37
109	1	12.33	49.41	33.73	12.34	49.42	33.71	12.34	49.45	33.70
110	1	9.74	49.56	34.10	9.76	49.57	34.10	9.76	49.57	34.10

M121Q/F114N (including Pro115, Gly116) – OX

Atom number	Atomic number	ε = 1			ε = 5.7			ε = 78.4		
		X	Y	Z	X	Y	Z	X	Y	Z
1	29	12.39	52.08	31.63	12.26	52.09	31.69	12.24	52.09	31.71
2	16	12.23	52.11	29.45	12.35	52.03	29.47	12.35	52.03	29.47
3	6	11.13	50.73	28.86	11.16	50.74	28.87	11.16	50.74	28.87
4*	6	11.21	50.54	27.34	11.21	50.54	27.34	11.21	50.54	27.34
5	6	12.63	50.14	26.92	12.62	50.13	26.89	12.62	50.14	26.89
6	8	13.07	49.01	27.13	13.03	48.98	27.03	13.04	48.99	27.04
7	7	13.39	51.13	26.35	13.38	51.13	26.35	13.38	51.13	26.34
8*	6	14.73	50.89	25.85	14.73	50.89	25.85	14.73	50.89	25.85
9	6	15.83	51.52	26.72	15.83	51.51	26.73	15.83	51.50	26.73
10	8	16.99	51.61	26.29	17.00	51.55	26.31	17.00	51.51	26.34
11	7	15.47	52.06	27.91	15.46	52.05	27.90	15.46	52.06	27.90
12*	6	16.48	52.55	28.82	16.48	52.55	28.81	16.48	52.55	28.81
13	6	17.36	51.35	29.26	17.36	51.35	29.26	17.35	51.34	29.24
14	8	16.81	50.26	29.47	16.80	50.26	29.49	16.78	50.27	29.53
15*	6	14.76	47.95	31.60	14.76	47.95	31.60	14.76	47.95	31.60
16	6	13.80	47.01	30.83	13.83	46.97	30.84	13.84	46.96	30.85
17	6	14.18	49.36	31.46	14.15	49.35	31.45	14.15	49.35	31.45
18	8	12.74	46.68	31.36	12.81	46.55	31.40	12.84	46.52	31.41
19	7	14.16	46.70	29.57	14.15	46.71	29.56	14.15	46.72	29.55
20	6	14.77	50.43	32.33	14.72	50.42	32.32	14.70	50.41	32.36
21*	6	13.32	45.88	28.69	13.32	45.88	28.69	13.32	45.88	28.69
22	6	16.01	50.56	32.90	15.94	50.54	32.93	15.88	50.49	33.05
23	7	14.06	51.60	32.57	14.01	51.60	32.54	14.00	51.61	32.54
24	7	16.04	51.80	33.49	15.96	51.79	33.52	15.89	51.73	33.65
25	6	14.86	52.41	33.26	14.79	52.40	33.26	14.76	52.38	33.31
26	7	11.43	53.19	32.98	11.45	53.17	33.16	11.45	53.16	33.19
27	6	10.51	54.22	32.79	10.55	54.22	33.05	10.55	54.22	33.08
28	6	11.60	53.06	34.29	11.74	53.05	34.45	11.74	53.04	34.48
29	6	10.07	54.70	31.44	10.00	54.71	31.74	10.00	54.71	31.78
30	6	10.14	54.69	34.02	10.30	54.72	34.30	10.32	54.72	34.33
31	7	10.83	53.95	34.95	11.06	53.96	35.17	11.08	53.96	35.20
32	6	11.24	55.26	30.58	11.12	55.21	30.79	11.10	55.21	30.81
33	7	12.01	56.26	31.29	11.96	56.22	31.40	11.96	56.22	31.41
34	6	10.71	55.90	29.29	10.51	55.82	29.51	10.48	55.81	29.54
35	6	13.21	55.94	31.84	13.20	55.93	31.88	13.20	55.93	31.88
36	8	10.40	57.09	29.26	10.02	56.95	29.54	9.94	56.92	29.59
37	7	10.62	55.05	28.24	10.58	55.03	28.42	10.60	55.06	28.43
38*	6	13.96	57.07	32.51	13.96	57.07	32.51	13.96	57.07	32.51
39	8	13.67	54.80	31.84	13.68	54.79	31.82	13.68	54.79	31.81
40	6	9.99	55.41	26.99	9.98	55.40	27.15	10.03	55.46	27.16
41	6	9.05	54.24	26.59	9.05	54.25	26.69	9.08	54.36	26.64
42	6	11.03	55.81	25.92	11.04	55.83	26.12	11.13	55.88	26.16
43	8	8.92	53.25	27.30	8.84	53.26	27.39	8.78	53.38	27.32
44	7	8.35	54.44	25.44	8.47	54.43	25.47	8.57	54.57	25.40
45	6	12.02	54.69	25.60	12.01	54.71	25.74	12.06	54.74	25.76

46	6	7.39	53.47	24.93	7.52	53.48	24.92	7.59	53.66	24.82
47	7	13.12	55.03	24.91	13.02	55.03	24.91	13.07	55.03	24.92
48	8	11.79	53.53	25.96	11.85	53.57	26.20	11.87	53.59	26.20
49*	6	9.90	47.88	31.09	9.90	47.88	31.09	9.90	47.88	31.09
50*	6	9.41	46.46	30.92	9.41	46.46	30.92	9.41	46.46	30.92
51*	6	10.16	48.22	32.54	10.16	48.22	32.54	10.16	48.22	32.54
52	1	11.43	49.82	29.38	11.41	49.81	29.38	11.41	49.81	29.37
53	1	10.11	50.98	29.15	10.15	51.04	29.16	10.16	51.03	29.16
54*	1	10.85	51.40	26.82	10.85	51.40	26.82	10.85	51.40	26.82
55*	1	10.60	49.70	27.10	10.60	49.70	27.10	10.60	49.70	27.10
56	1	12.95	52.03	26.17	12.97	52.05	26.23	12.96	52.06	26.22
57*	1	14.85	49.83	25.80	14.85	49.83	25.80	14.85	49.83	25.80
58	1	14.53	51.87	28.26	14.52	51.90	28.27	14.51	51.93	28.26
59*	1	17.09	53.28	28.31	17.09	53.28	28.31	17.09	53.28	28.31
60	1	19.58	49.73	28.69	19.55	49.71	28.65	19.47	49.63	28.55
61*	1	14.78	47.62	32.62	14.78	47.62	32.62	14.78	47.62	32.62
62	1	13.12	49.29	31.66	13.08	49.26	31.65	13.07	49.26	31.63
63	1	14.29	49.67	30.41	14.24	49.66	30.40	14.26	49.68	30.41
64	1	15.06	47.02	29.24	15.02	47.09	29.20	15.02	47.09	29.20
65*	1	13.80	44.94	28.50	13.80	44.94	28.50	13.80	44.94	28.50
66*	1	12.41	45.72	29.23	12.41	45.72	29.23	12.41	45.72	29.23
67*	1	13.11	46.37	27.76	13.11	46.37	27.76	13.11	46.37	27.76
68	1	16.88	49.92	32.89	16.81	49.91	32.94	16.72	49.82	33.12
69	1	16.87	52.27	33.80	16.77	52.24	33.92	16.65	52.13	34.17
70	1	14.65	53.44	33.50	14.56	53.42	33.51	14.53	53.40	33.58
71	1	12.25	52.34	34.76	12.42	52.32	34.88	12.41	52.31	34.91
72	1	9.31	55.48	31.57	9.30	55.53	31.94	9.30	55.53	31.98
73	1	9.60	53.89	30.87	9.44	53.92	31.23	9.43	53.92	31.27
74	1	9.45	55.48	34.30	9.67	55.52	34.64	9.70	55.54	34.67
75	1	10.78	54.05	35.96	11.10	54.07	36.18	11.13	54.07	36.20
76	1	11.90	54.43	30.36	11.75	54.36	30.55	11.73	54.36	30.57
77	1	11.72	57.22	31.17	11.61	57.17	31.41	11.60	57.16	31.45
78	1	10.87	54.07	28.36	10.97	54.09	28.50	11.03	54.14	28.49
79	1	13.52	58.06	32.32	13.50	58.05	32.33	13.52	58.05	32.29
80	1	14.99	57.06	32.16	14.98	57.07	32.12	14.99	57.04	32.16
81	1	13.98	56.90	33.59	14.02	56.90	33.59	13.97	56.93	33.59
82	1	9.37	56.29	27.19	9.35	56.27	27.36	9.43	56.35	27.36
83	1	11.57	56.69	26.28	11.61	56.67	26.54	11.73	56.67	26.62
84	1	10.53	56.11	24.99	10.57	56.23	25.21	10.70	56.33	25.26
85	1	8.45	55.31	24.95	8.58	55.32	25.00	8.75	55.44	24.93
86	1	7.43	52.60	25.58	7.78	52.48	25.28	7.92	52.63	24.94
87	1	6.38	53.88	24.93	6.49	53.70	25.23	6.61	53.76	25.29
88	1	7.65	53.17	23.90	7.58	53.50	23.83	7.50	53.88	23.75
89	1	13.79	54.30	24.65	13.68	54.30	24.65	13.72	54.30	24.66
90	1	13.27	55.97	24.58	13.12	55.96	24.53	13.21	55.97	24.59
91*	1	10.85	47.93	30.60	10.85	47.93	30.60	10.85	47.93	30.60
92*	1	9.10	48.52	30.77	9.10	48.52	30.77	9.10	48.52	30.77
93*	1	8.88	46.16	31.80	8.88	46.16	31.80	8.88	46.16	31.80
94*	1	10.24	45.81	30.77	10.24	45.81	30.77	10.24	45.81	30.77

95*	1	8.75	46.41	30.08	8.75	46.41	30.08	8.75	46.41	30.08
96*	1	9.24	48.04	33.05	9.24	48.04	33.05	9.24	48.04	33.05
97*	1	11.04	47.66	32.81	11.04	47.66	32.81	11.04	47.66	32.81
98	6	14.93	51.42	24.40	14.93	51.42	24.41	14.94	51.42	24.41
99	8	15.08	52.85	24.39	15.00	52.86	24.39	15.02	52.85	24.38
100	6	13.79	51.06	23.46	13.82	51.00	23.45	13.85	50.99	23.44
101	1	13.63	49.98	23.45	13.73	49.91	23.44	13.76	49.90	23.43
102	1	12.85	51.54	23.78	12.86	51.43	23.75	12.88	51.41	23.73
103	1	14.03	51.39	22.45	14.06	51.34	22.44	14.09	51.33	22.43
104	1	15.86	50.98	24.04	15.89	51.02	24.06	15.91	51.02	24.08
105	1	15.96	53.03	24.76	15.86	53.10	24.76	15.87	53.11	24.75
106	7	18.69	51.50	29.37	18.69	51.49	29.35	18.69	51.44	29.25
107	6	19.56	50.33	29.61	19.55	50.31	29.57	19.53	50.25	29.45
108	6	19.09	49.45	30.79	19.10	49.44	30.75	19.12	49.41	30.67
109	8	18.98	49.89	31.93	19.02	49.88	31.90	19.08	49.88	31.81
110	6	20.93	50.96	29.88	20.94	50.93	29.80	20.94	50.84	29.60
111	6	20.92	52.21	28.99	20.92	52.18	28.91	20.91	52.06	28.67
112	6	19.48	52.72	29.11	19.49	52.71	29.06	19.49	52.63	28.88
113	7	18.91	48.14	30.47	18.89	48.13	30.43	18.88	48.11	30.39
114	6	18.51	47.15	31.45	18.50	47.15	31.41	18.50	47.15	31.40
115	6	17.01	47.01	31.74	17.00	47.00	31.70	17.00	47.00	31.68
116	8	16.63	46.14	32.51	16.62	46.10	32.44	16.61	46.08	32.40
117	7	16.15	47.89	31.13	16.15	47.92	31.14	16.16	47.92	31.15
118	6	15.80	53.26	30.01	15.81	53.26	30.01	15.82	53.26	30.01
119	1	14.98	53.86	29.61	14.99	53.88	29.62	15.05	53.94	29.62
120	1	15.38	52.53	30.69	15.37	52.53	30.68	15.32	52.54	30.65
121	1	21.65	52.96	29.31	21.66	52.92	29.21	21.68	52.80	28.91
122	1	19.37	53.42	29.94	19.40	53.41	29.90	19.47	53.36	29.69
123	1	21.75	50.27	29.66	21.74	50.23	29.56	21.72	50.11	29.33
124	1	20.98	51.23	30.94	21.02	51.21	30.86	21.09	51.14	30.64
125	1	18.88	46.17	31.15	18.87	46.16	31.11	18.88	46.16	31.13
126	1	18.98	47.41	32.40	18.97	47.40	32.36	18.96	47.44	32.35
127	1	18.89	47.89	29.49	18.88	47.87	29.45	18.85	47.81	29.42
128	1	16.52	48.66	30.58	16.52	48.69	30.58	16.52	48.70	30.59
129	1	19.15	53.21	28.19	19.14	53.19	28.15	19.11	53.09	27.96
130	1	21.15	51.94	27.96	21.11	51.90	27.87	21.04	51.75	27.63
131	6	16.77	54.16	30.78	16.79	54.13	30.78	16.85	54.03	30.83
132	7	16.92	55.42	30.27	16.88	55.42	30.36	16.91	55.36	30.60
133	8	17.40	53.79	31.76	17.50	53.69	31.68	17.61	53.46	31.62
134	1	17.58	56.04	30.71	17.53	56.04	30.83	17.58	55.92	31.11
135	1	16.38	55.75	29.48	16.25	55.81	29.68	16.25	55.84	30.01
136	8	11.36	50.28	32.20	11.08	50.36	31.93	11.07	50.36	31.91
137	7	9.43	50.39	33.37	9.85	50.26	33.82	9.88	50.25	33.84
138	6	10.39	49.70	32.70	10.42	49.69	32.74	10.42	49.70	32.74
139	1	9.48	51.40	33.39	9.95	51.25	33.98	9.99	51.24	34.01
140	1	8.62	49.93	33.74	9.29	49.72	34.47	9.35	49.71	34.50

M121Q/F114N (including Pro115, Gly116) – RED

Atom number	Atomic number	ε = 1			ε = 5.7			ε = 78.4		
		X	Y	Z	X	Y	Z	X	Y	Z
1	29	12.28	52.02	31.79	12.28	52.07	31.76	12.26	52.08	31.78
2	16	12.29	52.02	29.52	12.30	52.05	29.49	12.27	52.05	29.50
3	6	11.15	50.72	28.87	11.16	50.74	28.87	11.15	50.73	28.87
4*	6	11.21	50.54	27.34	11.21	50.54	27.34	11.21	50.54	27.34
5	6	12.62	50.13	26.91	12.62	50.13	26.89	12.62	50.14	26.91
6	8	13.06	49.00	27.10	13.05	48.99	27.07	13.07	49.01	27.12
7	7	13.37	51.12	26.32	13.37	51.12	26.32	13.37	51.12	26.31
8*	6	14.73	50.89	25.85	14.73	50.89	25.85	14.73	50.89	25.85
9	6	15.81	51.51	26.75	15.81	51.51	26.74	15.80	51.52	26.74
10	8	16.98	51.59	26.32	16.98	51.57	26.33	16.97	51.56	26.32
11	7	15.44	52.05	27.93	15.44	52.06	27.92	15.44	52.07	27.92
12*	6	16.49	52.55	28.82	16.49	52.55	28.82	16.49	52.55	28.82
13	6	17.36	51.33	29.21	17.35	51.32	29.20	17.34	51.31	29.18
14	8	16.79	50.30	29.61	16.78	50.32	29.65	16.76	50.32	29.66
15*	6	14.76	47.95	31.60	14.76	47.95	31.60	14.76	47.95	31.60
16	6	13.82	46.99	30.84	13.84	46.97	30.84	13.84	46.97	30.84
17	6	14.19	49.37	31.47	14.18	49.36	31.48	14.18	49.36	31.48
18	8	12.78	46.60	31.38	12.84	46.52	31.41	12.84	46.51	31.41
19	7	14.15	46.71	29.56	14.15	46.72	29.55	14.15	46.72	29.55
20	6	14.76	50.37	32.42	14.70	50.34	32.48	14.69	50.34	32.50
21*	6	13.32	45.88	28.69	13.32	45.88	28.69	13.32	45.88	28.69
22	6	15.94	50.40	33.12	15.78	50.28	33.33	15.74	50.27	33.37
23	7	14.04	51.53	32.71	14.04	51.55	32.68	14.03	51.56	32.67
24	7	15.93	51.57	33.85	15.77	51.46	34.05	15.73	51.45	34.08
25	6	14.77	52.22	33.57	14.71	52.19	33.62	14.69	52.19	33.63
26	7	11.12	53.03	33.10	11.14	53.07	33.10	11.15	53.09	33.12
27	6	10.31	54.16	33.01	10.31	54.18	33.03	10.31	54.20	33.05
28	6	11.25	52.78	34.39	11.29	52.81	34.39	11.31	52.84	34.41
29	6	9.92	54.73	31.69	9.90	54.77	31.72	9.88	54.79	31.75
30	6	9.96	54.56	34.27	9.97	54.58	34.30	9.99	54.61	34.32
31	7	10.56	53.68	35.14	10.60	53.69	35.15	10.63	53.73	35.17
32	6	11.18	55.09	30.85	11.13	55.14	30.85	11.10	55.16	30.87
33	7	11.98	56.12	31.49	11.96	56.15	31.49	11.94	56.17	31.50
34	6	10.76	55.66	29.49	10.68	55.71	29.51	10.63	55.75	29.53
35	6	13.15	55.87	32.10	13.16	55.88	32.05	13.16	55.90	32.00
36	8	10.50	56.87	29.38	10.35	56.91	29.43	10.23	56.92	29.48
37	7	10.67	54.77	28.49	10.65	54.85	28.47	10.67	54.92	28.47
38*	6	13.96	57.07	32.51	13.96	57.07	32.51	13.96	57.07	32.51
39	8	13.58	54.72	32.32	13.60	54.73	32.18	13.63	54.75	32.05
40	6	10.12	55.17	27.21	10.14	55.27	27.18	10.16	55.34	27.18
41	6	9.21	54.04	26.67	9.20	54.18	26.62	9.20	54.27	26.61
42	6	11.22	55.68	26.24	11.26	55.75	26.24	11.30	55.79	26.23
43	8	8.88	53.10	27.39	8.88	53.20	27.28	8.88	53.28	27.25
44	7	8.69	54.25	25.43	8.67	54.44	25.39	8.67	54.56	25.39
45	6	12.15	54.58	25.75	12.19	54.63	25.77	12.22	54.66	25.78

46	6	7.90	53.21	24.78	7.81	53.48	24.72	7.75	53.65	24.73
47	7	13.42	54.94	25.46	13.45	54.98	25.45	13.47	54.99	25.45
48	8	11.74	53.43	25.57	11.78	53.46	25.67	11.80	53.49	25.71
49*	6	9.90	47.88	31.09	9.90	47.88	31.09	9.90	47.88	31.09
50*	6	9.41	46.46	30.92	9.41	46.46	30.92	9.41	46.46	30.92
51*	6	10.16	48.22	32.54	10.16	48.22	32.54	10.16	48.22	32.54
52	1	11.40	49.77	29.34	11.43	49.79	29.35	11.41	49.78	29.35
53	1	10.12	50.98	29.15	10.14	50.98	29.16	10.12	50.96	29.15
54*	1	10.85	51.40	26.82	10.85	51.40	26.82	10.85	51.40	26.82
55*	1	10.60	49.70	27.10	10.60	49.70	27.10	10.60	49.70	27.10
56	1	12.92	52.00	26.13	12.93	52.02	26.14	12.92	52.02	26.12
57*	1	14.85	49.83	25.80	14.85	49.83	25.80	14.85	49.83	25.80
58	1	14.50	51.88	28.31	14.49	51.91	28.29	14.49	51.94	28.28
59*	1	17.09	53.28	28.31	17.09	53.28	28.31	17.09	53.28	28.31
60	1	19.35	49.51	28.40	19.18	49.43	28.25	19.11	49.38	28.18
61*	1	14.78	47.62	32.62	14.78	47.62	32.62	14.78	47.62	32.62
62	1	13.12	49.31	31.66	13.10	49.29	31.61	13.10	49.29	31.61
63	1	14.31	49.72	30.44	14.35	49.75	30.46	14.36	49.75	30.47
64	1	15.00	47.12	29.20	14.99	47.14	29.18	15.01	47.12	29.19
65*	1	13.80	44.94	28.50	13.80	44.94	28.50	13.80	44.94	28.50
66*	1	12.41	45.72	29.23	12.41	45.72	29.23	12.41	45.72	29.23
67*	1	13.11	46.37	27.76	13.11	46.37	27.76	13.11	46.37	27.76
68	1	16.79	49.74	33.12	16.55	49.54	33.46	16.50	49.52	33.53
69	1	16.69	51.92	34.41	16.45	51.74	34.74	16.38	51.72	34.80
70	1	14.51	53.19	33.97	14.47	53.17	34.00	14.45	53.18	34.00
71	1	11.80	51.94	34.80	11.86	51.99	34.80	11.89	52.03	34.83
72	1	9.29	55.62	31.82	9.27	55.65	31.88	9.26	55.67	31.92
73	1	9.35	54.00	31.11	9.31	54.04	31.14	9.28	54.07	31.18
74	1	9.34	55.38	34.62	9.35	55.38	34.66	9.37	55.41	34.68
75	1	10.50	53.68	36.15	10.55	53.70	36.16	10.59	53.74	36.18
76	1	11.76	54.18	30.77	11.71	54.22	30.76	11.68	54.25	30.76
77	1	11.73	57.07	31.23	11.68	57.10	31.33	11.63	57.13	31.42
78	1	10.99	53.80	28.65	11.00	53.89	28.62	11.03	53.97	28.61
79	1	13.44	58.02	32.35	13.44	58.02	32.39	13.43	58.02	32.43
80	1	14.89	57.08	31.93	14.89	57.11	31.94	14.89	57.14	31.94
81	1	14.23	56.98	33.56	14.22	56.94	33.56	14.22	56.89	33.55
82	1	9.48	56.03	27.41	9.51	56.15	27.38	9.56	56.24	27.37
83	1	11.77	56.47	26.76	11.83	56.53	26.75	11.88	56.57	26.74
84	1	10.76	56.15	25.36	10.84	56.22	25.35	10.89	56.26	25.33
85	1	9.16	54.90	24.82	9.04	55.21	24.85	8.99	55.38	24.89
86	1	7.32	52.69	25.55	7.13	53.04	25.44	7.01	53.29	25.44
87	1	7.22	53.66	24.05	7.24	54.00	23.95	7.24	54.19	23.93
88	1	8.53	52.47	24.27	8.39	52.68	24.25	8.27	52.79	24.30
89	1	14.07	54.24	25.09	14.08	54.27	25.07	14.10	54.28	25.07
90	1	13.76	55.87	25.65	13.77	55.93	25.55	13.80	55.95	25.51
91*	1	10.85	47.93	30.60	10.85	47.93	30.60	10.85	47.93	30.60
92*	1	9.10	48.52	30.77	9.10	48.52	30.77	9.10	48.52	30.77
93*	1	8.88	46.16	31.80	8.88	46.16	31.80	8.88	46.16	31.80
94*	1	10.24	45.81	30.77	10.24	45.81	30.77	10.24	45.81	30.77

95*	1	8.75	46.41	30.08	8.75	46.41	30.08	8.75	46.41	30.08
96*	1	9.24	48.04	33.05	9.24	48.04	33.05	9.24	48.04	33.05
97*	1	11.04	47.66	32.81	11.04	47.66	32.81	11.04	47.66	32.81
98	6	14.98	51.43	24.41	14.97	51.42	24.41	14.97	51.41	24.40
99	8	15.26	52.85	24.42	15.24	52.83	24.39	15.22	52.83	24.38
100	6	13.81	51.19	23.46	13.81	51.16	23.46	13.82	51.12	23.45
101	1	13.55	50.13	23.44	13.55	50.10	23.45	13.57	50.06	23.46
102	1	12.93	51.76	23.77	12.93	51.72	23.77	12.93	51.68	23.75
103	1	14.09	51.51	22.45	14.08	51.46	22.45	14.10	51.42	22.44
104	1	15.87	50.92	24.04	15.87	50.90	24.04	15.88	50.90	24.05
105	1	16.13	52.92	24.85	16.11	52.93	24.82	16.10	52.95	24.78
106	7	18.70	51.39	29.08	18.68	51.33	28.98	18.67	51.29	28.92
107	6	19.50	50.17	29.27	19.45	50.08	29.09	19.41	50.02	29.01
108	6	19.17	49.40	30.56	19.20	49.32	30.41	19.19	49.28	30.33
109	8	19.25	49.90	31.67	19.41	49.83	31.51	19.42	49.79	31.43
110	6	20.94	50.72	29.27	20.90	50.55	28.95	20.87	50.47	28.83
111	6	20.88	51.84	28.23	20.79	51.70	27.94	20.75	51.63	27.83
112	6	19.48	52.46	28.43	19.47	52.39	28.31	19.46	52.35	28.26
113	7	18.87	48.08	30.34	18.83	48.02	30.23	18.80	47.98	30.18
114	6	18.51	47.18	31.41	18.50	47.14	31.33	18.49	47.11	31.29
115	6	17.01	47.00	31.70	17.00	46.98	31.65	17.00	46.97	31.63
116	8	16.66	46.10	32.45	16.64	46.05	32.37	16.63	46.03	32.34
117	7	16.16	47.90	31.13	16.16	47.91	31.14	16.15	47.91	31.14
118	6	15.86	53.23	30.04	15.87	53.22	30.04	15.87	53.21	30.05
119	1	15.01	53.84	29.70	15.07	53.90	29.72	15.14	53.96	29.73
120	1	15.44	52.50	30.73	15.40	52.49	30.70	15.32	52.49	30.65
121	1	21.67	52.58	28.35	21.64	52.39	27.98	21.61	52.30	27.85
122	1	19.50	53.34	29.08	19.61	53.22	29.00	19.64	53.16	28.97
123	1	21.67	49.94	29.04	21.57	49.75	28.63	21.51	49.66	28.47
124	1	21.15	51.11	30.27	21.24	50.92	29.92	21.25	50.82	29.79
125	1	18.92	46.18	31.22	18.90	46.14	31.15	18.88	46.11	31.11
126	1	18.97	47.56	32.32	18.98	47.54	32.22	18.99	47.51	32.17
127	1	18.66	47.80	29.39	18.58	47.73	29.30	18.57	47.67	29.25
128	1	16.52	48.70	30.62	16.52	48.72	30.63	16.52	48.71	30.61
129	1	19.01	52.73	27.48	18.93	52.74	27.42	18.91	52.74	27.40
130	1	20.96	51.42	27.22	20.73	51.29	26.92	20.65	51.24	26.81
131	6	16.86	54.16	30.74	16.90	54.05	30.81	16.93	53.90	30.91
132	7	16.43	54.65	31.94	16.44	54.61	31.96	16.45	54.58	31.97
133	8	17.96	54.45	30.27	18.06	54.20	30.42	18.14	53.83	30.66
134	1	17.03	55.34	32.36	17.07	55.22	32.46	17.11	55.04	32.58
135	1	15.45	54.59	32.21	15.44	54.61	32.18	15.44	54.65	32.14
136	8	11.55	50.17	32.88	11.55	50.19	32.79	11.55	50.19	32.78
137	7	9.34	50.52	32.57	9.31	50.49	32.72	9.31	50.49	32.75
138	6	10.43	49.70	32.68	10.42	49.70	32.70	10.42	49.70	32.71
139	1	9.56	51.49	32.39	9.46	51.49	32.63	9.44	51.49	32.73
140	1	8.49	50.16	32.17	8.40	50.12	32.50	8.38	50.12	32.62

M121Q/F114P – OX

Atom number	Atomic number	ε = 1			ε = 5.7			ε = 78.4		
		X	Y	Z	X	Y	Z	X	Y	Z
1	29	12.26	51.98	31.75	12.24	51.99	31.77	12.27	52.16	31.67
2	16	11.57	52.14	29.70	11.63	52.15	29.68	11.92	52.21	29.50
3	6	10.93	50.62	28.90	10.97	50.62	28.90	11.03	50.73	28.89
4	6	11.22	50.54	27.39	11.22	50.54	27.39	11.23	50.54	27.38
5	6	12.69	50.23	27.09	12.68	50.21	27.05	12.68	50.21	27.04
6	8	13.27	49.27	27.60	13.22	49.18	27.48	13.21	49.17	27.45
7	7	13.33	51.09	26.23	13.34	51.10	26.26	13.34	51.11	26.26
8*	6	14.73	50.89	25.85	14.73	50.89	25.85	14.73	50.89	25.85
9	6	15.78	51.31	26.87	15.81	51.34	26.84	15.82	51.33	26.84
10	8	16.91	50.82	26.73	16.93	50.82	26.76	16.91	50.75	26.79
11	7	15.46	52.15	27.87	15.52	52.26	27.79	15.55	52.30	27.75
12*	6	16.52	52.53	28.81	16.52	52.53	28.81	16.52	52.53	28.81
13	6	17.01	51.50	29.86	17.02	51.25	29.49	16.95	51.23	29.51
14	8	17.02	51.79	31.06	16.30	50.29	29.73	16.21	50.27	29.67
15	6	15.13	54.44	28.41	14.98	54.35	28.78	15.10	54.43	28.69
16	6	14.43	53.23	27.80	14.45	53.29	27.80	14.59	53.43	27.64
17	7	17.53	50.35	29.36	18.33	51.30	29.88	18.22	51.27	30.00
18	6	18.25	49.40	30.19	18.98	50.16	30.50	18.81	50.13	30.69
19*	6	14.76	47.95	31.60	14.76	47.95	31.60	14.76	47.95	31.60
20	6	13.86	46.97	30.84	13.90	46.92	30.86	13.92	46.90	30.86
21	6	14.10	49.34	31.51	14.09	49.33	31.50	14.05	49.31	31.51
22	8	12.86	46.49	31.40	12.99	46.32	31.45	13.05	46.24	31.46
23	7	14.17	46.72	29.55	14.15	46.74	29.54	14.15	46.75	29.53
24	6	14.60	50.36	32.48	14.62	50.37	32.43	14.57	50.36	32.43
25*	6	13.32	45.88	28.69	13.32	45.88	28.69	13.32	45.88	28.69
26	6	15.70	50.36	33.30	15.75	50.39	33.21	15.68	50.37	33.24
27	7	13.99	51.60	32.58	13.98	51.59	32.58	13.96	51.62	32.52
28	7	15.74	51.61	33.88	15.78	51.64	33.81	15.74	51.63	33.81
29	6	14.72	52.34	33.41	14.71	52.33	33.41	14.70	52.35	33.35
30	7	11.20	53.17	33.05	11.25	53.19	33.08	11.40	53.27	33.09
31	6	10.40	54.29	32.85	10.43	54.30	32.89	10.51	54.32	32.93
32	6	11.32	53.00	34.36	11.40	53.03	34.39	11.64	53.15	34.39
33	6	10.00	54.81	31.50	9.99	54.81	31.56	10.00	54.81	31.61
34	6	10.05	54.79	34.08	10.10	54.79	34.13	10.22	54.83	34.17
35	7	10.64	53.97	35.02	10.72	53.98	35.05	10.94	54.08	35.07
36	6	11.19	55.31	30.64	11.15	55.32	30.66	11.12	55.34	30.67
37	7	12.00	56.29	31.32	11.99	56.29	31.33	11.97	56.32	31.32
38	6	10.69	55.94	29.33	10.61	55.94	29.37	10.51	55.94	29.41
39	6	13.14	55.94	31.95	13.16	55.95	31.90	13.18	55.97	31.84
40	8	10.56	57.16	29.23	10.35	57.15	29.31	10.08	57.10	29.41
41*	6	13.96	57.07	32.51	13.96	57.07	32.51	13.96	57.07	32.51
42	8	13.50	54.76	32.08	13.57	54.78	31.94	13.61	54.81	31.79
43	7	8.13	54.56	25.54	8.16	54.52	25.52	8.15	54.47	25.56
44	6	7.16	53.62	25.00	7.16	53.58	25.04	7.13	53.53	25.13
45*	6	9.90	47.88	31.09	9.90	47.88	31.09	9.90	47.88	31.09

46*	6	9.41	46.46	30.92	9.41	46.46	30.92	9.41	46.46	30.92
47*	6	10.16	48.22	32.54	10.16	48.22	32.54	10.16	48.22	32.54
48	7	10.41	55.06	28.35	10.43	55.08	28.35	10.47	55.12	28.34
49*	6	9.79	55.45	27.11	9.79	55.45	27.11	9.79	55.45	27.11
50	6	8.84	54.31	26.67	8.81	54.33	26.70	8.79	54.33	26.75
51	6	10.83	55.86	26.04	10.82	55.84	26.02	10.79	55.82	25.98
52	6	11.77	54.74	25.60	11.73	54.69	25.58	11.69	54.67	25.54
53	8	8.73	53.28	27.33	8.62	53.34	27.41	8.57	53.39	27.51
54	7	12.83	55.11	24.83	12.74	55.03	24.74	12.66	54.98	24.66
55	8	11.59	53.56	25.92	11.56	53.53	25.97	11.53	53.52	25.98
56	6	16.01	53.81	29.49	15.82	53.51	29.76	15.82	53.55	29.72
57	1	13.48	54.40	24.52	13.40	54.31	24.45	13.33	54.26	24.40
58	1	12.98	56.07	24.57	12.89	55.98	24.44	12.81	55.93	24.36
59	1	14.42	55.17	28.82	14.17	54.88	29.28	14.28	55.00	29.13
60	1	15.74	54.96	27.66	15.61	55.08	28.27	15.80	55.14	28.24
61	1	13.55	52.95	28.39	13.52	52.87	28.17	13.59	53.10	27.89
62	1	14.13	53.40	26.77	14.29	53.69	26.80	14.60	53.84	26.63
63	1	11.37	49.75	29.38	11.42	49.75	29.38	11.38	49.85	29.43
64	1	9.85	50.61	29.05	9.89	50.61	29.08	9.96	50.86	29.11
65*	1	10.85	51.40	26.82	10.85	51.40	26.82	10.85	51.40	26.82
66*	1	10.60	49.70	27.10	10.60	49.70	27.10	10.60	49.70	27.10
67	1	12.83	51.92	25.93	12.85	51.96	25.99	12.86	51.97	26.00
68*	1	14.85	49.83	25.80	14.85	49.83	25.80	14.85	49.83	25.80
69	1	17.56	50.27	28.35	18.91	52.03	29.50	18.81	52.05	29.76
70	1	17.91	48.38	30.00	19.18	49.35	29.79	18.98	49.29	30.01
71	1	19.33	49.43	30.00	19.93	50.49	30.93	19.77	50.44	31.11
72*	1	14.78	47.62	32.62	14.78	47.62	32.62	14.78	47.62	32.62
73	1	13.03	49.21	31.68	13.03	49.20	31.71	12.99	49.16	31.72
74	1	14.22	49.73	30.49	14.19	49.71	30.48	14.12	49.69	30.49
75	1	14.90	47.26	29.11	14.81	47.37	29.08	14.78	47.40	29.08
76*	1	13.80	44.95	28.50	13.80	44.94	28.50	13.80	44.94	28.50
77*	1	12.41	45.72	29.23	12.41	45.72	29.23	12.41	45.72	29.23
78*	1	13.11	46.37	27.76	13.11	46.37	27.76	13.11	46.37	27.76
79	1	16.45	49.61	33.49	16.52	49.65	33.38	16.41	49.62	33.46
80	1	16.50	51.96	34.45	16.50	51.97	34.44	16.45	51.97	34.44
81	1	14.53	53.38	33.62	14.50	53.36	33.67	14.52	53.39	33.58
82	1	11.87	52.21	34.84	11.96	52.25	34.87	12.28	52.43	34.86
83	1	9.29	55.63	31.65	9.28	55.63	31.72	9.27	55.60	31.79
84	1	9.48	54.02	30.93	9.46	54.02	31.00	9.48	54.00	31.08
85	1	9.44	55.63	34.35	9.48	55.63	34.42	9.58	55.65	34.48
86	1	10.56	54.06	36.02	10.66	54.07	36.06	10.94	54.19	36.08
87	1	11.83	54.46	30.42	11.79	54.47	30.42	11.76	54.49	30.43
88	1	11.79	57.26	31.09	11.72	57.27	31.22	11.66	57.27	31.33
89	1	13.55	58.06	32.28	13.54	58.06	32.30	13.54	58.06	32.34
90	1	14.98	57.00	32.11	14.98	57.03	32.13	14.99	57.05	32.14
91	1	14.03	56.96	33.59	14.00	56.93	33.59	13.99	56.88	33.58
92	1	8.21	55.46	25.10	8.26	55.40	25.04	8.28	55.31	25.02
93	1	7.27	52.69	25.55	7.52	52.56	25.21	7.49	52.51	25.26
94	1	6.14	53.99	25.11	6.21	53.69	25.57	6.21	53.64	25.72

95	1	7.36	53.43	23.94	7.01	53.74	23.98	6.90	53.71	24.08
96*	1	10.85	47.93	30.60	10.85	47.93	30.60	10.85	47.93	30.60
97*	1	9.10	48.52	30.77	9.10	48.52	30.77	9.10	48.52	30.77
98*	1	8.88	46.16	31.80	8.88	46.16	31.80	8.88	46.16	31.80
99*	1	10.24	45.81	30.77	10.24	45.81	30.77	10.24	45.81	30.77
100*	1	8.75	46.41	30.08	8.75	46.41	30.08	8.75	46.41	30.08
101*	1	9.24	48.04	33.05	9.24	48.04	33.05	9.24	48.04	33.05
102*	1	11.04	47.66	32.81	11.04	47.66	32.81	11.04	47.66	32.81
103	1	18.08	49.65	31.24	18.35	49.76	31.30	18.16	49.80	31.50
104*	1	15.76	47.98	31.21	15.76	47.98	31.21	15.76	47.98	31.21
105	1	16.84	54.44	29.81	16.53	54.10	30.34	16.52	54.09	30.35
106	1	15.43	53.55	30.38	15.17	52.97	30.45	15.09	53.04	30.36
107	1	10.33	56.26	25.14	10.31	56.24	25.13	10.26	56.20	25.10
108	1	11.42	56.69	26.44	11.43	56.66	26.40	11.41	56.65	26.33
109	1	9.19	56.35	27.31	9.20	56.35	27.31	9.21	56.36	27.31
110	1	10.52	54.05	28.51	10.64	54.09	28.49	10.83	54.17	28.44
111	1	17.42	52.75	28.21	17.39	53.02	28.35	17.43	52.99	28.39
112	6	15.04	51.48	24.46	15.01	51.46	24.44	15.00	51.43	24.43
113	8	14.96	52.93	24.48	14.87	52.90	24.42	14.83	52.86	24.36
114	6	14.11	50.99	23.37	14.08	50.90	23.37	14.08	50.81	23.38
115	1	16.07	51.19	24.23	16.05	51.20	24.21	16.04	51.18	24.20
116	1	14.13	49.90	23.31	14.15	49.81	23.34	14.18	49.72	23.38
117	1	13.08	51.30	23.55	13.04	51.18	23.57	13.04	51.07	23.57
118	1	14.42	51.40	22.40	14.36	51.30	22.40	14.36	51.19	22.39
119	1	15.80	53.27	24.81	15.69	53.29	24.76	15.67	53.29	24.59
120	7	9.26	50.47	32.74	9.25	50.45	32.79	9.82	50.24	33.83
121	8	11.51	50.23	32.76	11.50	50.23	32.75	11.17	50.34	32.02
122	6	10.38	49.70	32.70	10.37	49.70	32.71	10.43	49.69	32.77
123	1	9.36	51.47	32.69	9.34	51.46	32.80	9.96	51.22	34.03
124	1	8.35	50.07	32.61	8.33	50.05	32.72	9.23	49.71	34.45

M121Q/F114P – RED

Atom number	Atomic number	ε = 1			ε = 5.7			ε = 78.4		
		X	Y	Z	X	Y	Z	X	Y	Z
1	29	12.13	52.04	31.84	12.12	52.05	31.87	12.09	52.04	31.92
2	16	11.25	52.16	29.77	11.32	52.15	29.76	11.41	52.16	29.75
3	6	10.94	50.58	28.90	10.96	50.58	28.91	10.94	50.61	28.91
4	6	11.22	50.54	27.39	11.22	50.54	27.39	11.22	50.54	27.39
5	6	12.68	50.20	27.06	12.67	50.20	27.04	12.68	50.22	27.06
6	8	13.24	49.20	27.49	13.21	49.16	27.44	13.23	49.20	27.50
7	7	13.33	51.08	26.23	13.33	51.09	26.24	13.33	51.10	26.25
8*	6	14.73	50.89	25.85	14.73	50.89	25.85	14.73	50.89	25.85
9	6	15.79	51.36	26.85	15.80	51.35	26.85	15.80	51.35	26.84
10	8	16.95	50.96	26.69	16.94	50.90	26.71	16.93	50.85	26.74
11	7	15.47	52.21	27.86	15.49	52.22	27.84	15.51	52.25	27.80
12*	6	16.52	52.53	28.81	16.52	52.53	28.81	16.52	52.53	28.81

13	6	17.18	51.26	29.39	17.14	51.28	29.46	17.00	51.25	29.52
14	8	16.54	50.38	29.94	16.49	50.27	29.74	16.29	50.25	29.64
15	6	14.77	54.14	29.04	14.85	54.22	28.92	14.98	54.35	28.77
16	6	14.28	53.07	28.05	14.33	53.15	27.96	14.41	53.25	27.86
17	7	18.55	51.26	29.33	18.45	51.42	29.78	18.25	51.34	30.03
18	6	19.28	50.06	29.71	19.21	50.34	30.39	18.86	50.25	30.78
19*	6	14.76	47.95	31.60	14.76	47.95	31.60	14.76	47.95	31.60
20	6	13.92	46.90	30.86	13.95	46.87	30.86	13.94	46.88	30.86
21	6	14.07	49.32	31.49	14.08	49.32	31.47	14.06	49.31	31.50
22	8	13.05	46.25	31.45	13.15	46.15	31.47	13.13	46.17	31.47
23	7	14.16	46.75	29.52	14.15	46.77	29.52	14.15	46.76	29.52
24	6	14.58	50.36	32.42	14.60	50.36	32.41	14.58	50.35	32.43
25*	6	13.32	45.88	28.69	13.32	45.88	28.69	13.32	45.88	28.69
26	6	15.70	50.37	33.22	15.71	50.36	33.20	15.69	50.35	33.25
27	7	13.92	51.58	32.56	13.93	51.58	32.55	13.93	51.58	32.56
28	7	15.72	51.61	33.83	15.74	51.60	33.82	15.72	51.59	33.86
29	6	14.64	52.31	33.40	14.65	52.30	33.40	14.65	52.30	33.41
30	7	11.04	53.22	33.09	11.09	53.25	33.13	11.10	53.27	33.16
31	6	10.30	54.38	32.90	10.34	54.41	32.94	10.36	54.44	32.96
32	6	11.12	53.03	34.39	11.22	53.10	34.44	11.25	53.15	34.47
33	6	9.92	54.90	31.55	9.93	54.91	31.59	9.93	54.93	31.61
34	6	9.94	54.88	34.13	10.04	54.94	34.17	10.09	55.01	34.18
35	7	10.47	54.02	35.07	10.60	54.10	35.11	10.66	54.18	35.13
36	6	11.15	55.29	30.68	11.13	55.31	30.68	11.11	55.34	30.69
37	7	11.99	56.28	31.32	11.99	56.29	31.31	11.99	56.31	31.31
38	6	10.70	55.89	29.33	10.62	55.91	29.36	10.58	55.93	29.38
39	6	13.12	55.93	31.99	13.16	55.94	31.91	13.18	55.96	31.86
40	8	10.69	57.12	29.16	10.48	57.14	29.24	10.36	57.15	29.28
41*	6	13.96	57.07	32.51	13.96	57.07	32.51	13.96	57.07	32.51
42	8	13.46	54.77	32.18	13.55	54.78	32.00	13.61	54.80	31.86
43	7	8.31	54.65	25.32	8.30	54.64	25.35	8.26	54.60	25.39
44	6	7.43	53.71	24.64	7.30	53.78	24.72	7.24	53.72	24.84
45*	6	9.90	47.88	31.09	9.90	47.88	31.09	9.90	47.88	31.09
46*	6	9.41	46.46	30.92	9.41	46.46	30.92	9.41	46.46	30.92
47*	6	10.16	48.22	32.54	10.16	48.22	32.54	10.16	48.22	32.54
48	7	10.32	55.00	28.38	10.34	55.02	28.38	10.37	55.05	28.38
49*	6	9.79	55.45	27.11	9.79	55.45	27.11	9.79	55.45	27.11
50	6	8.83	54.37	26.56	8.81	54.38	26.58	8.79	54.38	26.62
51	6	10.92	55.88	26.14	10.90	55.87	26.11	10.87	55.85	26.08
52	6	11.82	54.74	25.66	11.79	54.73	25.64	11.76	54.70	25.59
53	8	8.51	53.40	27.22	8.48	53.41	27.25	8.46	53.43	27.32
54	7	13.00	55.12	25.10	12.89	55.09	24.93	12.82	55.06	24.84
55	8	11.49	53.56	25.74	11.53	53.54	25.87	11.51	53.52	25.86
56	6	15.81	53.38	29.87	15.81	53.44	29.82	15.85	53.55	29.74
57	1	13.65	54.40	24.78	13.53	54.37	24.62	13.47	54.34	24.53
58	1	13.30	56.09	25.11	13.12	56.06	24.77	13.04	56.03	24.67
59	1	13.95	54.52	29.65	14.03	54.67	29.49	14.19	54.90	29.27
60	1	15.24	54.98	28.50	15.38	55.02	28.39	15.59	55.05	28.19
61	1	13.45	52.52	28.50	13.46	52.65	28.39	13.51	52.81	28.31

62	1	13.96	53.50	27.10	14.08	53.56	26.98	14.17	53.61	26.86
63	1	11.52	49.78	29.37	11.54	49.77	29.35	11.46	49.76	29.36
64	1	9.88	50.34	29.05	9.91	50.35	29.06	9.87	50.44	29.06
65*	1	10.85	51.40	26.82	10.85	51.40	26.82	10.85	51.40	26.82
66*	1	10.60	49.70	27.10	10.60	49.70	27.10	10.60	49.70	27.10
67	1	12.84	51.93	25.96	12.85	51.95	25.99	12.85	51.95	25.97
68*	1	14.85	49.83	25.80	14.85	49.83	25.80	14.85	49.83	25.80
69	1	18.94	51.79	28.57	18.97	52.19	29.38	18.77	52.19	29.90
70	1	19.21	49.27	28.96	19.53	49.59	29.66	18.64	49.30	30.30
71	1	20.33	50.31	29.88	20.09	50.75	30.88	19.95	50.40	30.79
72*	1	14.78	47.62	32.62	14.78	47.62	32.62	14.78	47.62	32.62
73	1	13.00	49.19	31.70	13.01	49.21	31.66	13.00	49.18	31.71
74	1	14.15	49.70	30.47	14.19	49.69	30.44	14.14	49.69	30.47
75	1	14.69	47.48	29.06	14.66	47.51	29.05	14.70	47.47	29.06
76*	1	13.80	44.94	28.50	13.80	44.94	28.50	13.80	44.94	28.50
77*	1	12.41	45.72	29.23	12.41	45.72	29.23	12.41	45.72	29.23
78*	1	13.11	46.37	27.76	13.11	46.37	27.76	13.11	46.37	27.76
79	1	16.47	49.64	33.38	16.48	49.63	33.37	16.44	49.61	33.44
80	1	16.42	51.95	34.47	16.43	51.93	34.47	16.41	51.92	34.52
81	1	14.42	53.33	33.66	14.44	53.31	33.68	14.44	53.32	33.69
82	1	11.60	52.19	34.87	11.73	52.29	34.93	11.77	52.35	34.97
83	1	9.27	55.77	31.67	9.27	55.77	31.72	9.27	55.79	31.75
84	1	9.37	54.13	31.00	9.37	54.13	31.06	9.36	54.15	31.09
85	1	9.37	55.75	34.41	9.47	55.82	34.44	9.54	55.90	34.45
86	1	10.37	54.09	36.07	10.54	54.21	36.11	10.64	54.31	36.13
87	1	11.74	54.39	30.54	11.72	54.42	30.51	11.70	54.44	30.50
88	1	11.82	57.24	31.03	11.76	57.26	31.16	11.71	57.28	31.26
89	1	13.58	58.06	32.23	13.58	58.06	32.25	13.56	58.07	32.30
90	1	14.98	56.96	32.13	14.99	56.98	32.17	15.00	57.02	32.17
91	1	14.01	57.00	33.60	13.96	56.97	33.60	13.96	56.92	33.59
92	1	8.68	55.42	24.80	8.55	55.50	24.88	8.48	55.45	24.89
93	1	7.06	53.01	25.38	7.29	52.84	25.26	7.56	52.68	24.93
94	1	6.59	54.24	24.19	6.30	54.23	24.75	6.28	53.83	25.36
95	1	7.96	53.15	23.86	7.57	53.60	23.68	7.11	53.96	23.79
96*	1	10.85	47.93	30.60	10.85	47.93	30.60	10.85	47.93	30.60
97*	1	9.10	48.52	30.77	9.10	48.52	30.77	9.10	48.52	30.77
98*	1	8.88	46.16	31.80	8.88	46.16	31.80	8.88	46.16	31.80
99*	1	10.24	45.81	30.77	10.24	45.81	30.77	10.24	45.81	30.77
100*	1	8.75	46.41	30.08	8.75	46.41	30.08	8.75	46.41	30.08
101*	1	9.24	48.04	33.05	9.24	48.04	33.05	9.24	48.04	33.05
102*	1	11.04	47.66	32.81	11.04	47.66	32.81	11.04	47.66	32.81
103	1	18.85	49.67	30.64	18.58	49.84	31.13	18.50	50.22	31.81
104*	1	15.76	47.98	31.21	15.76	47.98	31.21	15.76	47.98	31.21
105	1	16.50	54.02	30.41	16.51	54.07	30.38	16.58	54.16	30.27
106	1	15.30	52.73	30.58	15.24	52.83	30.53	15.22	53.05	30.48
107	1	10.51	56.39	25.26	10.47	56.36	25.23	10.42	56.34	25.20
108	1	11.52	56.64	26.66	11.51	56.63	26.60	11.51	56.62	26.54
109	1	9.20	56.36	27.30	9.21	56.36	27.30	9.22	56.36	27.30
110	1	10.38	53.99	28.58	10.50	54.02	28.56	10.57	54.06	28.56

111	1	17.30	53.13	28.31	17.33	53.09	28.32	17.40	52.99	28.33
112	6	15.05	51.51	24.47	15.03	51.49	24.46	15.01	51.47	24.45
113	8	15.05	52.95	24.54	14.95	52.93	24.48	14.89	52.90	24.44
114	6	14.07	51.11	23.38	14.09	51.00	23.37	14.08	50.93	23.37
115	1	16.06	51.17	24.21	16.06	51.20	24.22	16.05	51.20	24.21
116	1	14.03	50.03	23.28	14.12	49.91	23.30	14.14	49.83	23.33
117	1	13.07	51.48	23.60	13.06	51.30	23.57	13.05	51.21	23.57
118	1	14.40	51.54	22.42	14.39	51.42	22.41	14.37	51.32	22.40
119	1	15.83	53.21	25.06	15.74	53.27	24.93	15.69	53.29	24.81
120	8	11.51	50.17	33.11	11.53	50.15	33.10	11.52	50.17	33.06
121	7	9.42	50.56	32.37	9.43	50.57	32.40	9.39	50.55	32.47
122	6	10.43	49.71	32.70	10.44	49.70	32.71	10.43	49.70	32.71
123	1	9.80	51.47	32.09	9.81	51.48	32.16	9.72	51.49	32.26
124	1	8.68	50.22	31.77	8.66	50.25	31.84	8.57	50.23	31.98

Table S6 Reduction potentials from DFT calculations

Single point energies calculated using B3LYP/6-311++G(2df,p) method were used to calculate relative reduction potentials of 13 azurin mutants.

	OX (Hartrees)			RED (Hartrees)			ΔE (mV)			ΔΔE (mV)		
**mutant / ε**	ε = 1	ε = 5.7	ε = 78.4	ε = 1	ε = 5.7	ε = 78.4	ε = 1	ε = 5.7	ε = 78.4	ε = 1	ε = 5.7	ε = 78.4
**WT**	-3658.79	-3658.86	-3658.88	-3658.98	-3659.02	-3659.04	599.46	-177.12	-222.23			
**WT (include Pro115 Gly116)**	-4421.63	-4421.71	-4421.73	-4421.82	-4421.87	-4421.89	563.97	-146.52	-207.42			
**N47P**	-3567.43	-3567.50	-3567.52	-3567.61	-3567.65	-3567.67	530.78	-257.77	-302.93	-68.69	-80.64	-80.70
**N47S**	-3565.26	-3565.33	-3565.35	-3565.45	-3565.49	-3565.50	687.62	-102.61	-157.70	88.15	74.51	64.53
**F114N (include Pro115 Gly116)**	-4359.28	-4359.36	-4359.38	-4359.47	-4359.52	-4359.54	800.36	55.58	-37.28	236.39	202.10	170.13
**F114P**	-3775.53	-3775.60	-3775.62	-3775.70	-3775.75	-3775.77	319.77	-348.04	-302.19	-279.70	-170.92	-79.96
**M121G**	-3102.07	-3102.15	-3102.17	-3102.27	-3102.31	-3102.33	821.76	-44.75	-134.09	222.29	132.37	88.15
**M121L**	-3339.18	-3339.25	-3339.27	-3339.38	-3339.41	-3339.43	821.19	-6.30	-93.51	221.73	170.82	128.73
**M121V**	-3260.58	-3260.65	-3260.68	-3260.78	-3260.81	-3260.83	824.54	-28.65	-133.11	225.07	148.47	89.12
**M121Q**	-3390.01	-3390.08	-3390.11	-3390.19	-3390.24	-3390.26	454.63	-293.50	-380.40	-144.84	-116.38	-158.17
**M121N**	-3350.69	-3350.76	-3350.79	-3350.88	-3350.93	-3350.95	806.73	-11.80	-134.67	207.26	165.33	87.56
**M121L/F114N (include Pro115 Gly116)**	-4039.66	-4039.74	-4039.77	-4039.86	-4039.91	-4039.93	978.95	184.91	51.79	414.98	331.43	259.20
**M121L/F114P**	-3455.91	-3455.98	-3456.01	-3456.09	-3456.14	-3456.16	605.16	-155.83	-184.67	5.70	21.29	37.56
**M121Q/F114N (include Pro115 Gly116)**	-4090.50	-4090.58	-4090.61	-4090.69	-4090.74	-4090.77	677.09	-88.89	-212.28	113.12	57.63	-4.86
**M121Q/F114P**	-3506.75	-3506.82	-3506.85	-3506.92	-3506.97	-3506.99	240.82	-450.40	-424.44	-358.65	-273.28	-202.21

Table S7 DFT Charge and spin densities

Mulliken spin and charge densities for each of the 15 DFT models.

WT

	OX						RED		
	Spin			Charge			Charge		
	1	5.7	78.4	1	5.7	78.4	1	5.7	78.4
**Cu**	0.41	0.44	0.46	0.76	0.59	0.63	0.22	0.56	0.40
**His46**	0.05	0.05	0.05	0.95	0.98	0.97	0.76	0.71	0.77
**Cys112**	0.39	0.37	0.35	-2.31	-1.76	-1.84	-1.54	-1.80	-1.96
**His117**	0.08	0.08	0.08	1.17	1.11	1.20	1.05	0.74	0.80
**NH1**	0.04	0.04	0.04	-0.31	-0.26	-0.22	-0.33	-0.25	-0.17
**NH2**	0.02	0.02	0.02	-0.36	-0.25	-0.23	-0.14	-0.24	-0.32
**Elsewhere**	0.01	0.01	0.00	1.10	0.58	0.48	-0.02	0.28	0.48
**total**	1	1	1	1	1	1	0	0	0

WT (including Pro115, Gly116)

	OX						RED		
	Spin			Charge			Charge		
	1	5.7	78.4	1	5.7	78.4	1	5.7	78.4
**Cu**	0.42	0.43	0.44	0.84	1.18	1.26	0.89	0.93	0.80
**His46**	0.04	0.05	0.05	0.78	0.76	0.78	0.45	0.54	0.62
**Cys112**	0.39	0.38	0.37	-0.88	-1.07	-1.09	-1.18	-1.35	-1.43
**His117**	0.08	0.08	0.08	0.01	0.03	0.04	-0.02	-0.08	-0.03
**NH1**	0.04	0.04	0.04	-0.18	-0.22	-0.23	-0.13	-0.18	-0.16
**NH2**	0.01	0.01	0.01	0.06	0.09	0.10	0.27	0.22	0.16
**Elsewhere**	0.02	0.01	0.00	0.38	0.23	0.14	-0.28	-0.09	0.05
**total**	1	1	1	1	1	1	0	0	0

F114P - OX

	OX						RED		
	Spin			Charge			Charge		
	1	5.7	78.4	1	5.7	78.4	1	5.7	78.4
**Cu**	0.39	0.40	0.42	1.79	1.88	1.94	1.28	1.26	1.18
**His46**	0.03	0.04	0.05	0.66	0.72	0.75	0.36	0.43	0.58
**Cys112**	0.41	0.39	0.37	-1.25	-1.30	-1.35	-1.15	-1.31	-1.39
**His117**	0.08	0.08	0.08	-0.16	-0.10	-0.10	-0.29	-0.13	-0.17
**NH1**	0.05	0.04	0.04	-0.39	-0.40	-0.40	-0.22	-0.24	-0.28
**Elsewhere**	0.05	0.05	0.04	0.34	0.20	0.17	0.02	-0.01	0.07
**total**	1	1	1	1	1	1	0	0	0

F114N (including Pro115, Gly116)

	Spin			Charge			Charge		
	1	5.7	78.4	1	5.7	78.4	1	5.7	78.4
**Cu**	0.42	0.44	0.45	1.29	1.54	1.65	1.42	1.36	1.30
**His46**	0.05	0.05	0.05	0.60	0.71	0.70	0.19	0.36	0.47
**Cys112**	0.36	0.34	0.35	-1.18	-1.22	-1.34	-1.21	-1.34	-1.48
**His117**	0.08	0.09	0.09	-0.15	-0.06	-0.06	-0.21	-0.12	-0.12
**NH1**	0.05	0.06	0.05	-0.19	-0.32	-0.22	-0.07	-0.18	-0.14
**NH2**	0.02	0.01	0.01	0.08	-0.08	-0.13	0.18	0.04	-0.16
**Elsewhere**	0.02	0.01	0.00	0.55	0.43	0.41	-0.30	-0.12	0.13
**total**	1	1	1	1	1	1	0	0	0

N47P

	OX						RED		
	Spin			Charge			Charge		
	1	5.7	78.4	1	5.7	78.4	1	5.7	78.4
**Cu**	0.40	0.43	0.45	-0.01	0.01	0.02	0.70	0.89	0.42
**His46**	0.09	0.09	0.10	0.87	0.84	0.85	0.84	0.77	0.61
**Cys112**	0.41	0.39	0.37	-1.76	-1.56	-1.61	-2.40	-2.38	-2.04
**His117**	0.09	0.09	0.10	1.33	1.16	1.23	1.01	0.72	0.66
**NH2**	0.02	0.01	0.01	-0.11	-0.11	-0.08	0.25	0.13	0.06
**Elsewhere**	-0.01	-0.01	-0.01	0.67	0.66	0.60	-0.40	-0.13	0.29
**total**	1	1	1	1	1	1	0	0	0

N47S

	OX						RED		
	Spin			Charge			Charge		
	1	5.7	78.4	1	5.7	78.4	1	5.7	78.4
**Cu**	0.45	0.47	0.49	0.18	0.39	0.41	-0.05	0.13	-0.06
**His46**	0.05	0.05	0.05	0.73	0.69	0.74	0.50	0.52	0.61
**Cys112**	0.35	0.34	0.32	-1.71	-1.61	-1.70	-1.56	-1.46	-1.54
**His117**	0.09	0.09	0.09	1.24	1.05	1.14	1.12	0.79	0.78
**NH1**	0.05	0.05	0.05	-0.07	-0.05	-0.03	0.04	0.01	-0.01
**NH2**	0.01	0.01	0.01	-0.01	0.14	0.19	0.01	0.15	0.18
**Elsewhere**	0.00	-0.01	-0.01	0.64	0.38	0.25	-0.06	-0.14	0.03
**total**	1	1	1	1	1	1	0	0	0

M121G

	OX						RED		
	Spin			Charge			Charge		
	1	5.7	78.4	1	5.7	78.4	1	5.7	78.4
**Cu**	0.36	0.40	0.42	1.59	1.64	1.62	0.38	0.64	0.64
**His46**	0.06	0.06	0.07	0.56	0.66	0.72	0.48	0.58	0.60
**Cys112**	0.46	0.43	0.40	-3.16	-2.97	-2.95	-1.74	-1.83	-1.89
**His117**	0.05	0.06	0.06	1.64	1.57	1.64	1.39	1.03	0.97
**NH1**	0.02	0.03	0.02	-0.27	-0.26	-0.21	-0.18	-0.17	-0.10
**NH2**	0.03	0.03	0.03	-0.30	-0.15	-0.17	-0.26	-0.24	-0.33
**Elsewhere**	0.01	0.00	0.00	0.95	0.50	0.36	-0.07	0.00	0.11
**total**	1	1	1	1	1	1	0	0	0

M121L

	OX						RED		
	Spin			Charge			Charge		
	1	5.7	78.4	1	5.7	78.4	1	5.7	78.4
**Cu**	0.36	0.39	0.42	0.74	1.19	1.38	0.38	0.68	0.73
**His46**	0.06	0.06	0.06	0.24	0.38	0.37	0.59	0.52	0.55
**Cys112**	0.46	0.44	0.41	-1.39	-1.31	-1.38	-1.36	-1.16	-1.35
**His117**	0.07	0.07	0.07	0.61	0.37	0.36	0.66	0.31	0.29
**NH1**	0.01	0.01	0.01	-0.06	-0.10	-0.09	-0.41	-0.28	-0.22
**NH2**	0.02	0.02	0.02	-0.42	-0.22	-0.17	-0.19	-0.18	-0.24
**Elsewhere**	0.02	0.01	0.01	1.28	0.70	0.51	0.34	0.12	0.24
**total**	1	1	1	1	1	1	0	0	0

M121V

	OX						RED		
	Spin			Charge			Charge		
	**1**	**5.7**	**78.4**	**1**	**5.7**	**78.4**	**1**	**5.7**	**78.4**
**Cu**	0.37	0.40	0.43	1.15	1.22	1.16	0.01	0.32	0.42
**His46**	0.06	0.07	0.07	0.75	0.78	0.81	0.69	0.73	0.73
**Cys112**	0.44	0.41	0.39	-3.36	-2.90	-2.76	-1.60	-1.77	-2.06
**His117**	0.06	0.06	0.07	1.81	1.61	1.61	1.35	0.90	0.91
**NH1**	0.01	0.01	0.01	-0.14	-0.19	-0.19	-0.30	-0.24	-0.13
**NH2**	0.03	0.02	0.02	-0.38	-0.25	-0.27	-0.20	-0.38	-0.45
**Elsewhere**	0.03	0.02	0.01	1.17	0.73	0.64	0.05	0.45	0.58
**total**	1	1	1	1	1	1	0	0	0

M121Q

	OX						RED		
	Spin			Charge			Charge		
	**1**	**5.7**	**78.4**	**1**	**5.7**	**78.4**	**1**	**5.7**	**78.4**
**Cu**	0.43	0.48	0.50	1.50	0.35	0.37	0.51	1.57	-0.01
**His46**	0.05	0.04	0.04	1.05	1.31	1.32	0.86	0.49	1.41
**Cys112**	0.41	0.33	0.30	-2.22	-1.89	-2.07	-1.35	-1.68	-1.76
**His117**	0.06	0.09	0.10	-0.12	0.51	0.61	0.54	0.06	0.45
**NH1**	0.00	-0.01	-0.01	-0.26	-0.07	-0.04	-0.23	-0.19	-0.29
**NH2**	0.02	0.00	0.00	-0.26	-0.08	-0.19	-0.11	0.09	-0.20
**Gln121**	0.00	0.02	0.02	0.46	1.07	1.13	0.03	0.06	0.76
**Elsewhere**	0.03	0.05	0.05	0.86	-0.20	-0.13	-0.25	-0.40	-0.36
**total**	1	1	1	1	1	1	0	0	0

M121N

	OX						RED		
	Spin			Charge			Charge		
	**1**	**5.7**	**78.4**	**1**	**5.7**	**78.4**	**1**	**5.7**	**78.4**
**Cu**	0.38	0.42	0.43	0.50	0.24	1.15	0.19	0.21	0.19
**His46**	0.06	0.06	0.07	0.57	0.65	0.68	0.30	0.44	0.62
**Cys112**	0.42	0.40	0.38	-2.40	-1.48	-2.72	-1.44	-1.25	-1.37
**His117**	0.05	0.06	0.06	1.68	1.47	1.70	1.35	1.02	0.91
**NH1**	0.00	0.01	0.01	0.05	-0.26	-0.18	-0.26	-0.23	-0.15
**NH2**	0.03	0.02	0.03	-0.38	-0.26	-0.25	-0.29	-0.35	-0.43
**Elsewhere**	0.05	0.02	0.02	0.98	0.64	0.62	0.14	0.16	0.23
**total**	1	1	1	1	1	1	0	0	0

M121L/F114N (including Pro115, Gly116)

	OX						RED		
	Spin			Charge			Charge		
	1	5.7	78.4	1	5.7	78.4	1	5.7	78.4
**Cu**	0.37	0.40	0.42	1.28	1.49	1.57	1.05	1.11	1.21
**His46**	0.05	0.05	0.06	0.48	0.59	0.65	0.33	0.42	0.50
**Cys112**	0.42	0.39	0.39	-0.78	-0.72	-0.73	-0.86	-1.04	-1.27
**His117**	0.06	0.06	0.06	0.02	-0.28	-0.22	-0.17	-0.02	-0.12
**NH1**	0.03	0.03	0.03	-0.22	-0.25	-0.30	-0.20	-0.17	-0.13
**NH2**	0.01	0.02	0.01	0.24	0.19	0.06	0.24	0.15	-0.02
**Elsewhere**	0.05	0.04	0.03	0.00	-0.03	-0.02	-0.40	-0.45	-0.17
**total**	1	1	1	1	1	1	0	0	0

M121L/F114P

	OX						RED		
	Spin			Charge			Charge		
	1	5.7	78.4	1	5.7	78.4	1	5.7	78.4
**Cu**	0.32	0.35	0.36	2.28	2.29	2.38	1.56	1.09	1.37
**His46**	0.04	0.05	0.05	0.22	0.22	0.25	0.15	0.23	0.39
**Cys112**	0.48	0.46	0.45	-1.14	-1.32	-1.47	-0.86	-1.08	-1.39
**His117**	0.07	0.07	0.07	-0.85	-0.46	-0.33	-0.63	-0.05	0.03
**NH1**	0.03	0.03	0.03	-0.38	-0.25	-0.13	-0.24	-0.10	-0.20
**Elsewhere**	0.06	0.04	0.03	0.87	0.52	0.29	0.01	-0.09	-0.19
**total**	1	1	1	1	1	1	0	0	0

M121Q/F114N (including Pro115, Gly116)

	OX						RED		
	Spin			Charge			Charge		
	1	5.7	78.4	1	5.7	78.4	1	5.7	78.4
**Cu**	0.46	0.47	0.49	0.56	0.24	0.17	1.56	1.66	1.63
**His46**	0.06	0.05	0.05	0.96	1.08	1.11	0.12	0.14	0.20
**Cys112**	0.35	0.33	0.31	-0.94	-1.16	-1.14	-0.88	-1.09	-1.17
**His117**	0.07	0.08	0.08	-0.14	0.03	0.01	-0.41	-0.66	-0.59
**NH1**	0.03	0.06	0.06	-0.41	-0.19	-0.11	-0.10	-0.12	-0.12
**NH2**	0.01	0.00	-0.01	0.03	-0.10	-0.22	0.04	-0.01	-0.22
**M121Q**	0.00	0.02	0.02	0.93	1.20	1.28	0.06	0.14	0.20
**Elsewhere**	0.02	-0.01	-0.01	0.00	-0.11	-0.09	-0.39	-0.05	0.07
**total**	1	1	1	1	1	1	0	0	0

M121Q/F114P

	OX						RED		
	Spin			Charge			Charge		
	1	5.7	78.4	1	5.7	78.4	1	5.7	78.4
**Cu**	0.46	0.46	0.46	0.75	1.06	0.85	1.18	1.55	1.37
**His46**	0.04	0.04	0.05	0.55	0.71	0.90	0.05	0.04	0.29
**Cys112**	0.36	0.35	0.35	-1.15	-0.99	-1.23	-0.72	-1.02	-1.14
**His117**	0.06	0.07	0.09	0.24	-0.09	0.12	-0.12	-0.02	-0.06
**NH1**	0.00	0.00	0.00	-0.38	-0.28	-0.30	-0.19	-0.24	-0.28
**M121Q**	0.02	0.01	0.00	0.41	0.49	1.06	0.11	0.14	0.17
**Elsewhere**	0.08	0.07	0.06	0.59	0.10	-0.40	-0.32	-0.45	-0.36
**total**	1	1	1	1	1	1	0	0	0

Table S8 Calculated reduction potentials from model

Calculated reduction potentials of 34 azurin mutants using the combined continuum electrostatics + DFT + hydrophobicity correction model.

**Mutant**	**$\Delta\Delta E_{CE}$**	**$\Delta\Delta E_{CE}$ + ΔE_{hydro}**	**$\Delta\Delta E_{DFT}$**	**$\Delta\Delta E_{DFT}$ + ΔE_{hydro}**	**$\Delta\Delta E_{model}$**	**Exp range**
M44F	19				19	71 to 77
M44K	62				62	66
M44P	9				9	47 to 65
N47P			-81		-81	-68 to -27
N47S	73				73	90 to 161
N47L	106				106	110
W48M	1				1	9 to 36
W48L	1				1	-2 to 29
S89G	-7				-7	-20 to -12
E91N	21				21	0 - 8
F114A	19				19	15 to 64
F114I	8				8	-23 to -5
F114N			170		170	84 to 145
F114P			-80		-80	-111 to -56
F114S	55				55	36 to 54
F114V	4				4	10 to 18
M121A	45	62			62	53 to 73
M121G	58	5			5	7
M121I	29	132			132	128 to 138
M121K	-17				-17	2 to 18
M121L	31	114			114	86 to 115
M121N	58				58	28 to 48
M121Q	-95				-95	-91 to -64
M121V	30	124			124	125 to 145
M121L/N47S	104	187			187	190 to 252
M121L/F114N			274	259	259	235 to 305
M121L/F114P			38	23	23	-31 to 3
M121Q/N47S	-22				-22	-39 to 3
M121Q/F114N			-5		-5	-73 to -39
M121Q/F114P			-202		-202	-193 to -157
N47S/F114N	73		170		243	190 to 248
M121L/N47S/F114N	73		274	259	332	343 to 391
M121L/N47S/F114S	158	241			241	302 to 334
M121Q/N47S/F114N	73		-5		68	-4 to 28

Table S9 Computed reduction potentials for 124 prospective mutants

pKa calculations were performed for mutants with predicted reduction potential changes of >±20 mV to check the protonation states of mutant K/D residues, initially assumed to be charged.

mutant	ΔΔE (mV)	> ± 20 mV?	FDDH pKa of mutant K/D residue		Charged?
			OX	RED	
D11A	85.20	1	n/a	n/a	n/a
D11K	96.22	1	10.25	10.27	1
D23A	0.49	0			
D23K	1.07	0			
D55A	2.62	0			
D55K	4.84	0			
D62A	3.82	0			
D62K	7.09	0			
D69A	3.29	0			
D69K	4.98	0			
D6A	4.20	0			
D6K	7.97	0			
D71A	24.09	1	n/a	n/a	n/a
D71K	48.17	1	10.13	10.15	1
D76A	1.98	0			
D76K	2.97	0			
D77A	13.67	0			
D77K	47.84	1	10.24	10.29	1
D93A	19.43	0			
D93K	26.22	1	9.9	9.97	1
D98A	0.89	0			
D98K	1.54	0			
E104A	0.31	0			
E104K	0.63	0			
E106A	1.47	0			
E106K	3.68	0			
E2A	0.50	0			
E2K	0.71	0			
E91A	13.56	0			
E91K	27.75	1	10.07	10.08	1
K101A	-2.19	0			
K101D	-3.87	0			
K103A	-0.33	0			
K103D	-0.97	0			
K122A	-11.33	0			
K122D	-41.88	1	4.25	4.34	1
K128A	-0.79	0			
K128D	-1.29	0			
K24A	-0.19	0			

mutant	ΔΔE (mV)	> ± 20 mV?	FDDH pKa of mutant K/D residue		Charged?
K24D	-0.81	0			
K27A	-0.22	0			
K27D	-0.45	0			
K41A	-10.97	0			
K41D	-97.45	1	4.09	4.16	1
K70A	-2.18	0			
K70D	-7.03	0			
K74A	-1.70	0			
K74D	-5.36	0			
K85A	-4.29	0			
K85D	-14.70	0			
K92A	-12.24	0			
K92D	-21.39	1	4.2	4.16	1
M109A	0.16	0			
M109K	9.09	0			
M13A	4.15	0			
M13K	62.06	1	10.82	10.45	1
M56A	3.03	0			
M56K	49.07	1	10.32	10.34	1
M64A	-3.16	0			
M64K	9.13	0			
N10A	7.52	0			
N10K	20.49	1	10.26	10.28	1
N16A	9.06	0			
N16K	31.73	1	10.13	10.19	1
N18A	-3.78	0			
N18K	58.87	1	10.19	10.3	1
N32A	-0.67	0			
N32K	1.64	0			
N38A	-0.14	0			
N38K	1.45	0			
N42A	0.70	0			
N42K	4.91	0			
Q107A	-0.08	0			
Q107K	2.67	0			
Q12A	-1.25	0			
Q12K	3.61	0			
Q14A	-12.84	0			
Q14K	-5.26	0			
Q28A	-0.05	0			
Q28K	0.68	0			
Q57A	1.05	0			
Q57K	4.29	0			
Q8A	-3.52	0			
Q8K	3.84	0			
R79A	-3.59	0			
R79D	-7.26	0			

mutant	ΔΔE (mV)	> ± 20 mV?	FDDH pKa of mutant K/D residue		Charged?
S100A	-0.09	0			
S100K	0.08	0			
S25A	0.00	0			
S25K	0.08	0			
S34A	-6.25	0			
S34K	0.75	0			
S4A	-0.42	0			
S4K	0.60	0			
S51A	3.98	0			
S51K	54.22	1	0.14	1.17	0
S66A	-1.99	0			
S66K	1.51	0			
S78A	-0.31	0			
S78K	0.17	0			
S89A	-6.95	0			
S89K	-2.03	0			
S94A	-1.92	0			
S94K	-0.02	0			
S118A	-19.01	0			
S118D	-79.91	1	3.82	3.9	1
S118K	10.54	0			
T113S	1.64	0			
T124A	-1.54	0			
T124K	0.78	0			
T17A	0.14	0			
T17K	4.20	0			
T21A	-0.23	0			
T21K	0.82	0			
T30A	-0.88	0			
T30K	1.37	0			
T52A	-1.65	0			
T52K	-0.22	0			
T61A	-1.50	0			
T61K	0.41	0			
T84A	8.76	0			
T84K	76.00	1	-1.23	-0.54	0
T96A	-0.20	0			
T96K	1.04	0			
